# A comparison of the content and primary literature support for online medication information provided by Lexicomp and Wikipedia

**DOI:** 10.5195/jmla.2018.256

**Published:** 2018-07-01

**Authors:** Julia Alexandra Hunter, Taehoon Lee, Navindra Persaud

**Affiliations:** Faculty of Medicine, Royal College of Surgeons in Ireland, Dublin, Ireland, and Li Ka Shing Knowledge Institute of St. Michael’s Hospital, Toronto, ON, Canada; Center for Urban Health Solution, Li Ka Shing Knowledge Institute of St. Michael’s Hospital, Toronto, ON, Canada; Li Ka Shing Knowledge Institute of St. Michael’s Hospital, Toronto, ON, Canada, and Department of Family and Community Medicine, University of Toronto, 30 Bond Street, Toronto, ON, M5B 1W8, Canada

## Abstract

**Objectives:**

The research compared the comprehensiveness and accuracy of two online resources that provide drug information: Lexicomp and Wikipedia.

**Methods:**

Medication information on five commonly prescribed medications was identified and comparisons were made between resources and the relevant literature. An initial content comparison of the following three categories of medication information was performed: dose and instructions, uses, and adverse effects or warnings. The content comparison included sixteen points of comparison for each of the five investigated medications, totaling eighty content comparisons. For each of the medications, adverse reactions that appeared in only one of the resources were identified. When primary, peer-reviewed literature was not referenced supporting the discrepant adverse reactions, a literature search was performed to determine whether or not evidence existed to support the listed claims.

**Results:**

Lexicomp consistently provided more medication information, with information provided in 95.0% (76/80) of the content, compared to Wikipedia’s 42.5% (34/80). Lexicomp and Wikipedia had information present in 91.4% (32/35) and 20.0% (7/35) of dosing and instructions content, respectively. Adverse effects or warning content was provided in 97.5% (39/40) of Lexicomp content and 55.0% (22/40) of Wikipedia content. The “uses” category was present in both Lexicomp and Wikipedia for the 5 medications considered. Of adverse reactions listed solely in Lexicomp, 191/302 (63.2%) were supported by primary, peer-reviewed literature in contrast to 7/7 (100.0%) of adverse reactions listed only in Wikipedia. A review of US Food and Drug Administration Prescribing Information and the Adverse Event Reporting System dashboard found support for a respective 17/102 (16.7%) and 92/102 (90.2%) of Lexicomp’s adverse reactions that were not supported in the literature.

**Conclusion:**

Lexicomp is a comprehensive medication information tool that contains lists of adverse reactions that are not entirely supported by primary-peer reviewed literature.

## INTRODUCTION

In 2006, 80% of American Internet users were reported to have used the Internet to search for health topics [1]. Of those who used the Internet, 53% indicated that the information they gained during their health information searches impacted their care for themselves or care for someone else. Since this time, the Internet has gained over 2 billion users globally [2]. In today’s Web 2.0 online environment, characterized by active participation and collaboration [3], clinicians and patients are faced with a wide array of information sources that include sources based on online collaboration and conventional sources generated by experts.

Wikipedia is a free online encyclopedia that is written and edited continuously by its users [4]. Studies have reported the use of Wikipedia as a medication information resource that both physicians and pharmacists access [5–7]. Another study found that Wikipedia was the first search result for approximately 80% of searches for generic name medication on Bing, Google Canada, and Yahoo [8]. As the 7th most visited site on the web [9], Wikipedia is also a common source of health information for consumers. In a study evaluating health index keywords, Wikipedia appeared in the top 10 results for 71%–85% of the keywords and search engines tested [10].

Previous studies have assessed the completeness, accuracy, and reference sources used in Wikipedia articles that provide health and medication information [11–20]. One study of ten mental health topics found the information in Wikipedia to be roughly equivalent to that provided by centrally controlled websites, *Encyclopaedia Britannica,* and a psychiatry textbook [17], and another study suggested that drug information provided on Wikipedia was sufficiently accurate and comprehensive to be used for undergraduate medical education [20]. However, others have found information on Wikipedia to be incomplete and/or inaccurate when compared with peer-reviewed sources [11, 14, 16, 21]. To determine the origin of medication information found on Wikipedia, a previous study evaluated the references used in Wikipedia entries for statin medications and reported that Wikipedia most commonly cited peer-reviewed journals [13].

The desired amount of detail provided by online medication information varies by user group. For clinicians, paid online drug information compendiums such as Lexicomp provide users with comprehensive product monographs. In a study of commonly used drug information databases, Lexicomp received the top quality and performance scores and was the most preferred database of a group of practicing pharmacists [22].

While previous studies have highlighted the incompleteness of Wikipedia articles and compared the quality and quantity of references used in drug-related Wikipedia articles to those provided in Lexicomp pages, research to date has not compared the extent to which the discrepant items in each article are supported by primary, peer-reviewed literature [12, 13, 21]. Discrepant items are important because they mean that people would get different information depending on the source that they consulted. The authors compared the medication information content that Wikipedia and Lexicomp provided for five commonly prescribed medications. The accuracy of adverse reactions that were listed in either Lexicomp or Wikipedia (but not both) was then assessed through a literature search to determine if support for the adverse reaction information existed in the primary, peer-reviewed literature.

## METHODS

We identified a list of the most frequently prescribed medications in Canada from national-level prescribing data provided by IMS Health Canada, and five of the six most frequently prescribed medications were used in this investigation. The investigated medications were levothyroxine (a thyroid hormone replacement), atorvastatin (a cholesterol-lowering agent), pantoprazole (used to reduce gastric acidity), acetylsalicylic acid (an anti-inflammatory and platelet-inhibiting agent), and metformin (an oral hypoglycemic agent used in diabetes mellitus). Rosuvastatin, the fourth most frequently prescribed medication in Canada, was excluded from the study because we included atorvastatin, a drug of the same class that was more frequently prescribed. We identified the Wikipedia article for each of the medications and the corresponding Lexicomp “Lexi-Drug” monograph for the most common route of administration of each medication. To ensure that we were comparing content at the same time, we archived Wikipedia articles and Lexicomp monographs corresponding to each of the medications that we evaluated between August 1 and August 5, 2016.

We considered three categories of medication information in the initial content comparison: (1) dose and instructions, (2) uses, and (3) and adverse effects or warnings. Within the three categories, we used sixteen subcategories to further separate and compare information from the two sources. As there were five medications that we investigated, this created a total of eighty subcategory comparisons. The dosing instructions subcategories that we compared were: adult dosing, geriatric dosing, pediatric dosing, hepatic impairment dosing, renal impairment dosing, adjustment for toxicity, and administration. The subcategories compared in the adverse effects or warning category were: safety considerations, contraindications or disease-related concerns, concerns for special populations, dosage form considerations, other, pregnancy risk factors or considerations, breast feeding consideration, and adverse reactions. “Uses” was compared as one category. We selected the categories and subcategories based on the type of information found in commonly used medication information resources.

The presence of each subcategory was assessed using a rating system that included five possible outcomes: present in neither source, present in Wikipedia but not Lexicomp, present in Lexicomp but not Wikipedia, present in both with discrepancies, and present in both without discrepancies. We use the term “discrepancy” to describe any substantive difference between the two resources, not including instances where slightly different wording was used in the two resources. Information in a subcategory was described as “present” whenever information that fit into that subcategory was present, irrespective of the quantity or quality of the content. The rating “present in both with discrepancies” was used when content that fell into a subcategory was provided in both sources, but the provided information clearly differed. For example, in the subcategory “administration,” if one source provided information about oral and intravenous administration and the other provided information about only oral administration, this was considered “present in both with discrepancies.”

Information in a subcategory was deemed “present in both without discrepancies” when the central ideas were the same, even if the language used or the level of detail describing central ideas varied. In some instances, information was compared in a different subcategory than the subheading from which it originated in a Wikipedia article or Lexicomp monograph if it was determined that the subcategory used for comparison was more accurate.

Additionally, when information was repeated under different subheadings, it was not compared in both instances in order to avoid overestimating the differences between the two databases. In cases where the information listed under a particular subheading was deemed “present in both with discrepancies,” specific discrepancy details were recorded. Subcategory presence was evaluated by one medical student reviewer. Any queries were discussed with the research team, and a group consensus was subsequently reached. For the purposes of this study, comprehensiveness was determined by the number of subcategories covered in the online medication information that Lexicomp and Wikipedia provided.

“Adverse reactions” was the only subcategory where information was “present in both with discrepancies” for each of the investigated medications. As a result, this subcategory was chosen to be evaluated by a fact-checking literature search. An information specialist performed a literature search using two online literature databases, EMBASE and Ovid MEDLINE, to retrieve primary, peer-reviewed literature support for each of the adverse reactions listed in one source but not the other. The research team discussed and reached a group consensus on any queries that the reviewer presented. All primary, peer-reviewed literature retrieved via the literature search was included in the evaluation.

The information specialist grouped search results for each adverse event investigated, and the contents of each group underwent a title and abstract screening to determine whether it might contain relevant adverse event information. If it was deemed possible that the article contained relevant adverse event information, the article was retrieved and reviewed in full. One medical student reviewer subsequently reviewed search results to determine whether or not the primary, peer-reviewed sources agreed with the informational discrepancies identified in Wikipedia or Lexicomp. The process of article review was discussed with information specialists and hospital-based researchers. Once an article was found supporting a given adverse event, the remainder of the articles in that group were not screened. Inline citations that appeared in the monograph text to support claims were also reviewed to see if they provided primary, peer-reviewed support.

Adverse events were not generalized between medications in the same class, for other routes of administration, or for animal studies.

The US Food and Drug Administration (FDA) Prescribing Information (PI) (supplemental appendixes) as well as the FDA Adverse Events Reporting Systems (FAERS) dashboard were subsequently reviewed for adverse events that were not supported via the fact-checking literature search.

The comprehensiveness of each of the sources was quantified by evaluating the number of instances in which Lexicomp and Wikipedia provided information in each of the subcategories. Nonnumerical observations about the information provided in the three categories were also recorded. The support in the primary, peer-reviewed literature for adverse reactions listed in either Lexicomp or Wikipedia (not both) was evaluated in a step-wise approach as a percentage of the adverse reactions listed only in the source considered. Following a review of FDA PIs and the FAERS dashboard, the number of adverse events that we found support for was presented as percentages.

## RESULTS

### Content

**Figure 1:**
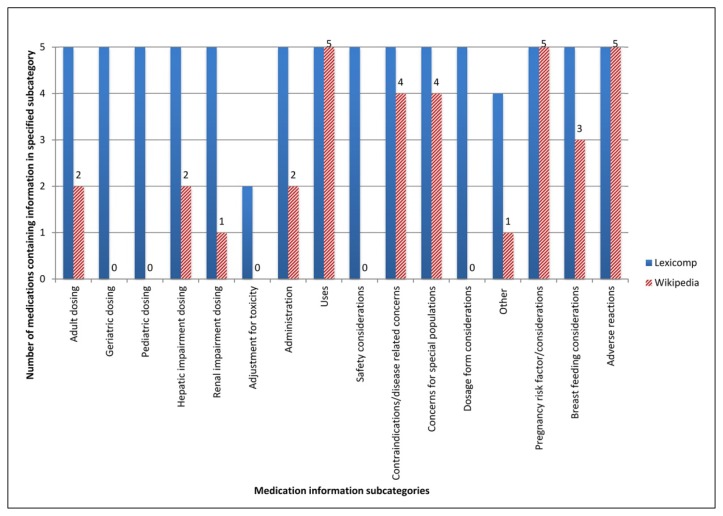
Number of medications containing information in specific medication information subcategories

Levothyroxine, atorvastatin, pantoprazole, acetylsalicylic acid, and metformin were used to compare the presence of support in the primary literature for information in Lexicomp and Wikipedia. Lexicomp monographs were found to be more comprehensive than the corresponding Wikipedia articles, as they consistently provided information in a greater number of subcategories for each of the investigated medications (Figure 1).

In 42/80 (53%) content comparisons, Lexicomp was the sole provider of information in a subcategory. The number of content comparisons with a rating of “present in both with discrepancies,” “present in both without discrepancies,” and “present in neither” was 14/80 (18%), 16/80 (20%), and 8/80 (10%), respectively. Specific ratings of each of the subcategories considered for each of the 5 medications are shown in Table 1.

**Table 1:** Subcategory content comparison for Lexicomp and Wikipedia medication information

Category	Subcategory	Levothyroxine	Atorvastatin	Pantoprazole	Acetylsalicylic acid	Metformin
Dosing and instructions	Adult dosing	L+W D*	L	L	L+W D*	L
	Geriatric dosing	L	L	L	L	L
	Pediatric dosing	L	L	L	L	L
	Hepatic impairment dosing	L	L+W ND	L	L	L+W ND
	Renal impairment dosing	L	L	L	L	L+W ND
	Adjustment for toxicity	L	L	N	N	N
	Administration	L+W D *	L+W D*	L	L	L
Uses	Uses	L+W ND	L+W ND	L+W D*	L+W D*	L+W D*
Adverse effects or warnings	Safety considerations	L	L	L	L	L
	Contraindications or disease-related concerns	L+W D*	L+W D*	L	L+W D	L+W D*
	Concerns for special populations	L+W ND	L+W D*	L+W D*	L	L+W ND
	Dosage form considerations	L	L	L	L	L
	Other	L	L	L	L+W ND	N
	Pregnancy risk factor or considerations	L+W ND	L+W ND	L+W ND	L+W ND	L+W ND
	Breast feeding considerations	L+W ND	L+W ND	L+W ND	L	L
	Adverse reactions	L+W D*	L+W D*	L+W D*	L+W D*	L+W D*

N=present in neither.

W=present in Wikipedia only.

L=present in Lexicomp only.

L+W D=present in both with discrepancies.

L+W D*=present in both with discrepancies, more information in Lexicomp.

L+W ND=present in both without discrepancies.

The subheadings considered in this evaluation never had an instance in which Wikipedia provided information on a subcategory and Lexicomp did not. Additionally, subcategories that received a rating of “present in both with discrepancies” had no instances in which Wikipedia presented a greater number of central ideas than Lexicomp did, and there was only one instance in which Lexicomp and Wikipedia presented the same number of central ideas.

### Dose and instructions

While some of the Wikipedia articles referred to dosing in a general sense, usage-specific dosing information only occurred in 2 instances, for subclinical hypothyroidism (a use of levothyroxine) and the prevention of cardiovascular events (a use of acetylsalicylic acid). Conversely, for all 5 medications, Lexicomp provided detailed usage and age-specific dosing information. Lexicomp also consistently provided information on 1 or more “special” dosing considerations (e.g., hepatic impairment, renal impairment, or toxicity). Atorvastatin and metformin were the only medications for which the corresponding Wikipedia article had dosing considerations for any of these subcategories. Wikipedia did not include any information about geriatric or pediatric dosing, while Lexicomp did in 10/10 (100%) of cases.

### Uses and adverse effects or warnings

Both Wikipedia and Lexicomp contained information on each of the five investigated medications’ uses, pregnancy risk factors or considerations, and associated adverse reactions. “Adverse reactions” was the only subcategory in which all five medications received a rating of “present in both with discrepancies.”

### Literature support for discrepant adverse reactions

For the 5 evaluated medications, Lexicomp monographs listed between 16 and 144 more adverse reactions that the corresponding Wikipedia articles (Table 2). Literature searches were performed for the 309 discrepant adverse reactions in both Embase and Ovid MEDLINE, returning a total of 62,122 articles for review. Overall, 191/302 (63%) and 7/7 (100%) of discrepant adverse reactions listed in Lexicomp and Wikipedia were supported by primary, peer-reviewed literature. Less than 3 adverse events were supported by an inline citation for each medication, despite the fact that there were between 19 and 145 adverse events listed in Lexicomp. We could not find support for adverse events listed in Lexicomp for a substantial proportion of adverse events.

**Table 2:** Literature search reporting characteristics, systematic reviews sample (n=75)

	Levothyroxine		Atorvastatin		Pantoprazole		Acetylsalicylic acid		Metformin	
Number of adverse reactions listed only in Lexicomp or only in Wikipedia										
Lexicomp	21		58		145		59		19	
Wikipedia	3		0		1		0		3	
Adverse reactions listed only in Lexicomp or only in Wikipedia supported by an inline citation										
Lexicomp	0	(—)	1	(1.7%)	2	(1.4%)	1	(1.7%)	0	(—)
Wikipedia	1	(33.3%)	0	(—)	1	(100.0%)	0	(—)		
Inline reference provided is primary literature										
Lexicomp	0	(—)	1	(1.7%)	1	(0.7%)	0	(—)	0	(—)
Wikipedia	0	(—)	0	(—)	0	(—)	3	(100.0%)		
Retrieved supporting reference from primary literature*										
Lexicomp	14	(66.7%)	46	(79.3%)	65	(44.8%)	50	(84.7%)	16	(84.2%)
Wikipedia	3	(100.0%)	0	(—)	1	(100.0%)	0	(—)	3	(100.0%)
Number of adverse events unsupported in Lexicomp following primary literature search*										
Lexicomp	7		12		80		9		3	
Number of previously unsupported adverse events in Lexicomp supported by US Food and Drug Administration (FDA) Prescribing Information (PI)										
Lexicomp	3	(42.9%)	9	(75.0%)	4	(5.0%)	—	—	1	(33.3%)
Number of previously unsupported adverse events in Lexicomp supported by FDA Adverse Events Reporting Systems (FAERS) Dashboard										
Lexicomp	7	(100.0%)	11	(91.7%)	71	(88.8%)	—	—	3	(100.0%)
Number of adverse events in Lexicomp remaining unsupported										
Lexicomp	0	(—)	1	(8.3%)	9	(11.2%)	9	(100.0%)	0	(—)

* These values included both references provided by the inline source indicated and those retrieved through a fact-checking literature search.

FDA PIs and the FAERS dashboard listed 17/102 (17%) and 92/102 (90%), respectively, of Lexicomp’s previously unsupported adverse reactions of levothyroxine, atorvastatin, pantoprazole, and metformin (Table 1). Adverse reactions for acetylsalicylic acid were not listed in the FDA PI or the FAERS dashboard, as FDA label formatting differs for over-the-counter drugs.

## DISCUSSION

Lexicomp contained more comprehensive medication information than Wikipedia for each of the five investigated medications. As the information in Lexicomp is organized under standard headings, it is not surprising that it was more comprehensive and uniform compared to that in Wikipedia. On Wikipedia, the presence of information in the subcategories varied considerably between medications. This, too, is not surprising given the ability of users to edit information freely and continuously. Evaluation of the subcategory information that was present indicated that the medication information provided on Wikipedia was highly variable; however, a larger medication sample size would be needed to draw further conclusions about the frequency with which specific subcategories of information can be found on Wikipedia.

For the five investigated medications, a literature search did not retrieve primary, peer-reviewed support for a large proportion of adverse event information that was found in Lexicomp. There are at least two possible explanations for the fact that adverse events listed in Lexicomp were less likely to be supported compared to those in Wikipedia. First, we may simply have been unable to find the primary sources to support the adverse events. This is still a problem as it means that readers will likely not be able to readily identify the underlying source of adverse event information, even if they attempt a search. Secondly, it might be a result of Lexicomp choosing to include a thorough range of potential adverse events and, thus, being a very sensitive resource. In comparison, Wikipedia provided specificity, with all adverse events retrieving support in the primary literature, but in doing so, Wikipedia did not include a large number of adverse events that have been reported and retrieved outside the primary literature.

Following our literature review, Lexicomp was contacted in order to understand their process for adding adverse drug reactions. Lexicomp responded that the source of unreferenced content is “prescribing information” (obtained from the FDA, Health Canada, and drug manufacturers) and that they are working to provide better referencing for adverse drug reactions.

Most of the adverse events that were not supported by our primary literature search were supported by the FAERS dashboard search. While the FAERS dashboard provides a platform through which the public can report adverse events while on a given medication, it does not require a strong association between the medication and the reported event. Additionally, both consumers and health care professionals can submit adverse event reports. These are significant limitations, which greatly reduce the reliability of adverse events reported using this system. Conversely, it should also be considered that a greater number of adverse reactions may be reported to FAERS due to the relative ease of reporting to FAERS compared to published literature describing an adverse reaction. It is also possible that publication bias exists, leading to adverse reactions being perceived as less interesting and less likely to be published, which may subsequently result in them only appearing in databases like the FAERS dashboard.

This study was limited by the fact that only one medical student reviewer compared online medication information and screened articles that the information specialist retrieved. A second major limitation was the use of only five medications in this evaluation. This study was also limited by the day-to-day variability of information provided on Wikipedia. In addition, only three major categories of information and one paid online drug compendium were considered. The results obtained using Lexicomp might differ from those obtained using other commonly accessed medication information sources. Additionally, we could not access six full-text articles that were returned by our search.

Future studies could compare a larger number of medications, using additional medication information sources.

## CONCLUSION

In our study of five frequently prescribed medications, we found that Lexicomp is a comprehensive source of online medication information, while Wikipedia’s comprehensiveness varies. A much higher proportion of discrepant adverse events that Wikipedia listed were supported by the primary, peer-reviewed literature compared with Lexicomp; however, following a subsequent review of FDA prescribing information and the FAERS dashboard, most discrepant adverse events from Lexicomp were supported. Medication information resources should provide references for each claim made about each medication to allow health care professionals to make informed decisions using accurate, evidence-based information.

## SUPPLEMENTAL FILES

Appendix A Prescribing Information: acetylsalicylic acid

Appendix B Prescribing Information: atorvastatin

Appendix C Prescribing Information: levothyroxine

Appendix D Prescribing Information: metformin

Appendix E Prescribing Information: pantoprazole
