# Supplementary material for: A comparison of the content and primary literature support for online medication information provided by Lexicomp and Wikipedia
Source: J Med Libr Assoc. 2018 Jul 1;106(3):352–60. doi: 10.5195/jmla.2018.256 (PMC6013145; doi:10.5195/jmla.2018.256)
Supplement: Appendix A [file jmla-106-352-s001.pdf]

ADULT LOW STRENGTH ASPIRIN- aspirin tablet, coated
Bedrock Brands, LLC

Disclaimer: Most OTC drugs are not reviewed and approved by FDA, however they may be marketed if they comply with applicable regulations and policies. FDA has not evaluated whether this product complies.

St Joseph 44-414SJ-Delisted

Active ingredient (in each tablet)

Aspirin 81 mg (NSAID)*

*nonsteroidal anti-inflammatory drug

Purpose

Pain reliever

Uses

temporarily relieves minor aches and pains

Warnings

Reye's syndrome: Children and teenagers who have or are recovering from chicken pox or flu-like symptoms should not use this product. When using this product, if changes in behavior with nausea and vomiting occur, consult a doctor because these symptoms could be an early sign of Reye's syndrome, a rare but serious illness.

Allergy alert: Aspirin may cause a severe allergic reaction, which may include:
- hives
- shock
- facial swelling
- asthma (wheezing)

Stomach bleeding warning: This product contains an NSAID, which may cause severe stomach bleeding. The chance is higher if you:
- are age 60 or older
- have had stomach ulcers or bleeding problems
- take a blood thinning (anticoagulant) or steroid drug
- take other drugs containing prescription or nonprescription) NSAIDs [aspirin, ibuprofen, naproxen, or others]
- have 3 or more alcoholic drinks every day while using this product
- take more or for a longer time than directed

Do not use

if you have ever had an allergic reaction to any pain reliever or fever reducer.

Ask a doctor before use if
- stomach bleeding warning applies to you
- you have a history of stomach problems, such as heartburn
- you have high blood pressure, heart disease, liver cirrhosis, or kidney disease

- you have asthma
- you are taking a diuretic

Ask a doctor or pharmacist before use if you are

taking a prescription drug for

- gout
- diabetes
- arthritis

Stop use and ask a doctor if

- you experience any of the following signs of stomach bleeding:
  - feel faint
  - have bloody or black stools
  - vomit blood
  - have stomach pain that does not get better
- and allergic reaction occurs
- new symptoms occur
- ringing in the ears or loss of hearing occurs
- pain gets worse or lasts more than 10 days
- redness or swelling is present

These could be signs of a serious condition.

If pregnant or breast-feeding,

ask a health professional before use. It is especially important not to use aspirin during the last 3 months of pregnancy unless definitely directed to do so by a doctor because it may cause prblems in the unborn child or complications during delivery.

Keep out of reach of children

In case of overdose, get medical help or contact a Poison Control Center (1-800-222-1222) right away.

Directions

- drink a full glass of water with each dose
- adults and children 12 years and over: take 4 to 8 tablets every 4 hours while symptoms persist. Do not exceed 48 tablets in 24 hours or as directed by a doctor.
- children under 12 years: do not use unless directed by a doctor

Other information

- store at 25°C (77°F); excursions permitted between 15°-30°F (59°-86°F)
- see end flap for expiration date and lot number

Inactive ingredients

colloidal silicon dioxide, croscarmellose sodium, corn starch, FD&C red #40 aluminum lake, FD&C yellow #6, hypromellose, methacrylic acid, microcrystalline cellulose, polydextrose, polyethylene glycol, shellac wax, simethicone, sodium, sodium bicarbonate, sodium lauryl sulfate, talc, titanium dioxide, triacetin, triethyl citrate

Questions or comments?

1-855-STJOE81 (1-855-785-6381)

Principal Display Panel

NDC 76000-414-32

Doctor Recommended 81mg Dose

ST. JOSEPH®
SAFETY COATED* ASPIRIN
PAIN RELIEVER **(NSAID)**
81mg

ASPIRIN REGIMEN

120 TABLETS
81mg each

*Coating Helps Protect Against Stomach Upset
Talk to your doctor before starting an aspirin regimen Aspirin is not right for everyone.

Dist. by: St. Josephs Health Products, LLC
Baltimore, MD 21201

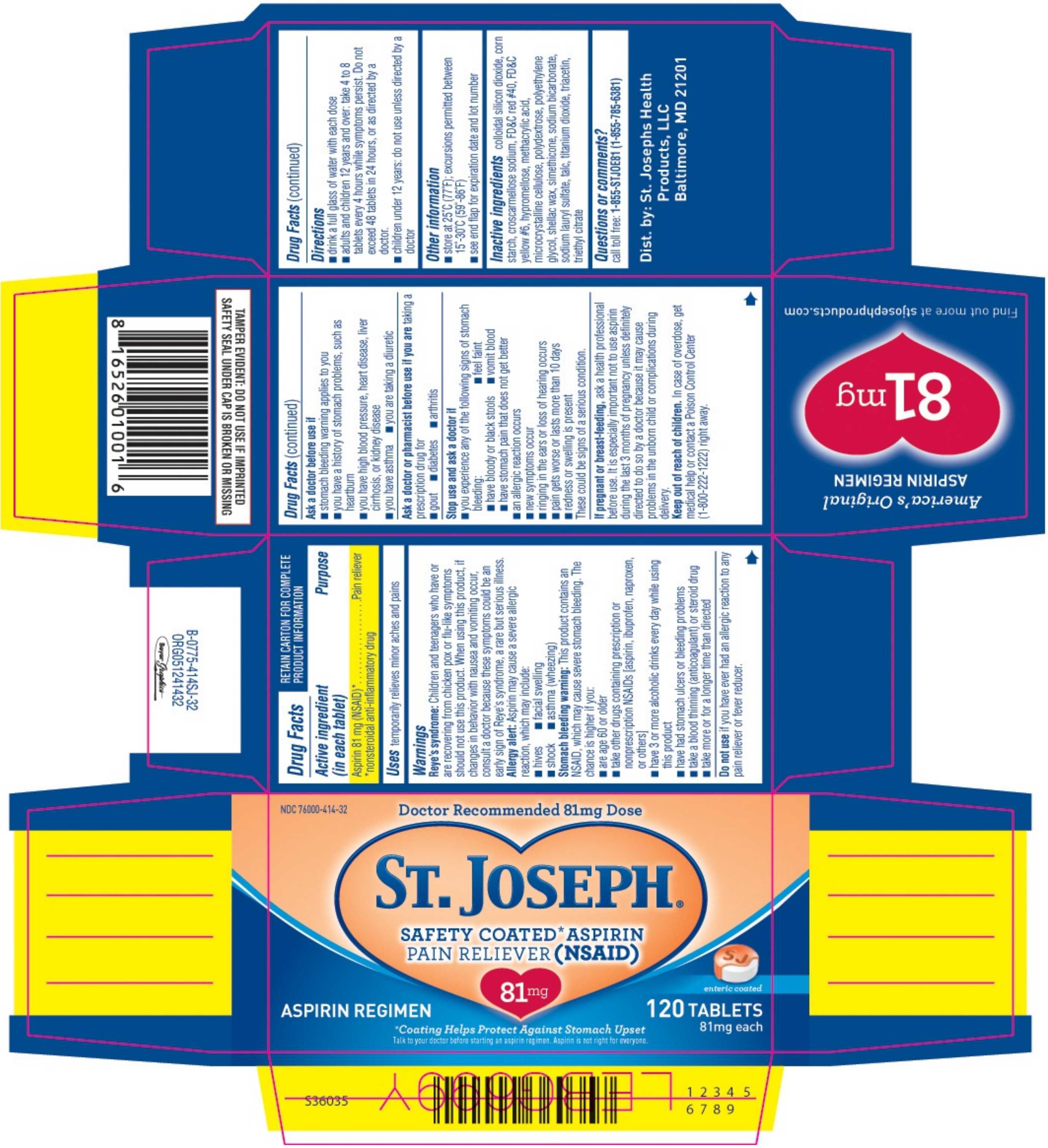

St. Joseph 44-414SJ

ADULT LOW STRENGTH ASPIRIN

aspirin tablet, coated

Product Information

**Product Type**	HUMAN OTC DRUG	**Item Code (Source)**	NDC:76000-414
**Route of Administration**	ORAL		

Active Ingredient/Active Moiety		
**Ingredient Name**	**Basis of Strength**	**Strength**
**ASPIRIN** (UNII: R16CO5Y76E) (ASPIRIN - UNII:R16CO5Y76E)	ASPIRIN	81 mg

Inactive Ingredients	
**Ingredient Name**	**Strength**
**SILICON DIOXIDE** (UNII: ETJ7Z6XBU4)	
**CROSCARMELLOSE SODIUM** (UNII: M28OL1HH48)	
**FD&C RED NO. 40** (UNII: WZB9127XOA)	
**FD&C YELLOW NO. 6** (UNII: H77VEI93A8)	
**HYPROMELLOSES** (UNII: 3NXW29V3WO)	
**POLYDEXTROSE** (UNII: VH2XOU12IE)	
**POLYETHYLENE GLYCOLS** (UNII: 3WJQ0SDW1A)	
**DIMETHICONE** (UNII: 92RU3N3Y1O)	
**SODIUM ALGINATE** (UNII: C269C4G2ZQ)	
**SODIUM BICARBONATE** (UNII: 8MDF5V39QO)	
**TALC** (UNII: 7SEV7J4R1U)	
**TITANIUM DIOXIDE** (UNII: 15FIX9V2JP)	
**TRIACETIN** (UNII: XHX3C3X673)	
**TRIETHYL CITRATE** (UNII: 8Z96QXD6UM)	

Product Characteristics			
**Color**	PINK	**Score**	no score
**Shape**	ROUND	**Size**	8mm
**Flavor**		**Imprint Code**	SJ
**Contains**			

Packaging				
**#**	**Item Code**	**Package Description**	**Marketing Start Date**	**Marketing End Date**
**1**	NDC:76000-414-32	1 in 1 CARTON		
**1**		120 in 1 BOTTLE, PLASTIC; Type 0: Not a Combination Product		

Marketing Information			
**Marketing Category**	**Application Number or Monograph Citation**	**Marketing Start Date**	**Marketing End Date**
OTC MONOGRAPH FINAL	part343	01/14/2004	05/27/2018

Labeler - Bedrock Brands, LLC (829056162)

Establishment

Name	Address	ID/FEI	Business Operations
LNK International, Inc.		038154464	PACK(76000-414)

Establishment

Name	Address	ID/FEI	Business Operations
LNK International, Inc.		832867894	MANUFACTURE(76000-414)
