# Supplementary material for: A comparison of the content and primary literature support for online medication information provided by Lexicomp and Wikipedia
Source: J Med Libr Assoc. 2018 Jul 1;106(3):352–60. doi: 10.5195/jmla.2018.256 (PMC6013145; doi:10.5195/jmla.2018.256)
Supplement: Appendix B [file jmla-106-352-s002.pdf]

LIPITOR- atorvastatin calcium tablet, film coated
Parke-Davis Div of Pfizer Inc

HIGHLIGHTS OF PRESCRIBING INFORMATION
These highlights do not include all the information needed to use LIPITOR safely and effectively. See full prescribing information for LIPITOR.

LIPITOR® (atorvastatin calcium) Tablets for oral administration
Initial U.S. Approval: 1996

INDICATIONS AND USAGE
LIPITOR is an inhibitor of HMG-CoA reductase (statin) indicated as an adjunct therapy to diet to:
- Reduce the risk of MI, stroke, revascularization procedures, and angina in patients without CHD, but with multiple risk factors (1.1).
- Reduce the risk of MI and stroke in patients with type 2 diabetes without CHD, but with multiple risk factors (1.1).
- Reduce the risk of non-fatal MI, fatal and non-fatal stroke, revascularization procedures, hospitalization for CHF, and angina in patients with CHD (1.1).
- Reduce elevated total-C, LDL-C, apo B, and TG levels and increase HDL-C in adult patients with primary hyperlipidemia (heterozygous familial and nonfamilial) and mixed dyslipidemia (1.2).
- Reduce elevated TG in patients with hypertriglyceridemia and primary dysbetalipoproteinemia (1.2).
- Reduce total-C and LDL-C in patients with homozygous familial hypercholesterolemia (HoFH) (1.2).
- Reduce elevated total-C, LDL-C, and apo B levels in boys and postmenarchal girls, 10 to 17 years of age, with heterozygous familial hypercholesterolemia after failing an adequate trial of diet therapy (1.2).

Limitations of Use
LIPITOR has not been studied in *Fredrickson* Types I and V dyslipidemias.

DOSAGE AND ADMINISTRATION
Dose range: 10 to 80 mg once daily (2.1).
Recommended start dose: 10 or 20 mg once daily (2.1).
Patients requiring large LDL-C reduction (>45%) may start at 40 mg once daily (2.1).
Pediatric starting dose: 10 mg once daily; maximum recommended dose: 20 mg once daily (2.2).

DOSAGE FORMS AND STRENGTHS
10, 20, 40, and 80 mg tablets (3).

CONTRAINDICATIONS
Active liver disease, which may include unexplained persistent elevations in hepatic transaminase levels (4.1).
Women who are pregnant or may become pregnant (4.3).
Nursing mothers (4.4).
Hypersensitivity to any component of this medication (4.2).

WARNINGS AND PRECAUTIONS
Skeletal muscle effects (e.g., myopathy and rhabdomyolysis): Risks increase when higher doses are used concomitantly with cyclosporine and strong CYP3A4 inhibitors (e.g., clarithromycin, itraconazole, HIV protease inhibitors). Predisposing factors include advanced age (> 65), uncontrolled hypothyroidism, and renal impairment. Rare cases of rhabdomyolysis with acute renal failure secondary to myoglobinuria have been reported. Advise patients to promptly report to their physician unexplained and/or persistent muscle pain, tenderness, or weakness. LIPITOR therapy should be discontinued if myopathy is diagnosed or suspected (5.1, 8.5).

Liver enzyme abnormalities: Persistent elevations in hepatic transaminases can occur. Check liver enzyme tests before initiating therapy and as clinically indicated thereafter (5.2).

A higher incidence of hemorrhagic stroke was seen in patients without CHD but with stroke or TIA within the previous 6 months in the LIPITOR 80 mg group vs. placebo (5.5).

ADVERSE REACTIONS
The most commonly reported adverse reactions (incidence ≥ 2%) in patients treated with LIPITOR in placebo-controlled trials regardless of causality were: nasopharyngitis, arthralgia, diarrhea, pain in extremity, and urinary tract infection (6.1).

To report SUSPECTED ADVERSE REACTIONS, contact Pfizer at (1-800-438-1985 and www.pfizer.com) or FDA at 1-800-FDA-1088 or *www.fda.gov/medwatch.*

DRUG INTERACTIONS

Drug Interactions Associated with Increased Risk of Myopathy/Rhabdomyolysis (2.6, 5.1, 7, 12.3)

Interacting Agents	Prescribing Recommendations
Cyclosporine, HIV protease inhibitors (tipranavir plus ritonavir), hepatitis C protease inhibitor (telaprevir)	Avoid atorvastatin
HIV protease inhibitor (lopinavir plus ritonavir)	Use with caution and lowest dose necessary
Clarithromycin, itraconazole, HIV protease inhibitors (saquinavir plus ritonavir, darunavir plus ritonavir, fosamprenavir, fosamprenavir plus ritonavir)	Do not exceed 20 mg atorvastatin daily
HIV protease inhibitor (nelfinavir)<br>Hepatitis C protease inhibitor (boceprevir)	Do not exceed 40 mg atorvastatin daily

- Other Lipid-Lowering Medications: Use with fibrate products or lipid-modifying doses (≥1 g/day) of niacin increases the risk of adverse skeletal muscle effects. Caution should be used when prescribing with LIPITOR (7).
- Digoxin: Patients should be monitored appropriately (7.8).
- Oral Contraceptives: Values for norethindrone and ethinyl estradiol may be increased (7.9).
- Rifampin should be simultaneously co-administered with LIPITOR (7.7).

USE IN SPECIFIC POPULATIONS

- Hepatic impairment: Plasma concentrations markedly increased in patients with chronic alcoholic liver disease (12.3).

See 17 for PATIENT COUNSELING INFORMATION and FDA-approved patient labeling.

Revised: 3/2015

FULL PRESCRIBING INFORMATION: CONTENTS*

1 INDICATIONS AND USAGE
1.1 Prevention of Cardiovascular Disease
1.2 Hyperlipidemia
1.3 Limitations of Use
2 DOSAGE AND ADMINISTRATION
2.1 Hyperlipidemia (Heterozygous Familial and Nonfamilial) and Mixed Dyslipidemia *(Fredrickson* Types IIa and IIb)
2.2 Heterozygous Familial Hypercholesterolemia in Pediatric Patients (10–17 years of age)
2.3 Homozygous Familial Hypercholesterolemia
2.4 Concomitant Lipid-Lowering Therapy
2.5 Dosage in Patients With Renal Impairment
2.6 Dosage in Patients Taking Cyclosporine, Clarithromycin, Itraconazole, or Certain Protease Inhibitors
3 DOSAGE FORMS AND STRENGTHS
4 CONTRAINDICATIONS
4.3 Pregnancy
4.4 Nursing mothers
5 WARNINGS AND PRECAUTIONS
5.1 Skeletal Muscle
5.2 Liver Dysfunction
5.3 Endocrine Function
5.4 CNS Toxicity
5.5 Use in Patients with Recent Stroke or TIA
6 ADVERSE REACTIONS
6.1 Clinical Trial Adverse Experiences
6.2 Postmarketing Experience
6.3 Pediatric Patients (ages 10–17 years)
7 DRUG INTERACTIONS

7.1 Strong Inhibitors of CYP 3A4
7.2 Grapefruit Juice
7.3 Cyclosporine
7.4 Gemfibrozil
7.5 Other Fibrates
7.6 Niacin
7.7 Rifampin or other Inducers of Cytochrome P450 3A4
7.8 Digoxin
7.9 Oral Contraceptives
7.10 Warfarin
7.11 Colchicine
8 USE IN SPECIFIC POPULATIONS
8.1 Pregnancy
8.3 Nursing Mothers
8.4 Pediatric Use
8.5 Geriatric Use
8.6 Hepatic Impairment
10 OVERDOSAGE
11 DESCRIPTION
12 CLINICAL PHARMACOLOGY
12.1 Mechanism of Action
12.2 Pharmacodynamics
12.3 Pharmacokinetics
13 NONCLINICAL TOXICOLOGY
13.1 Carcinogenesis, Mutagenesis, Impairment of Fertility
14 CLINICAL STUDIES
14.1 Prevention of Cardiovascular Disease
14.2 Hyperlipidemia (Heterozygous Familial and Nonfamilial) and Mixed Dyslipidemia *(Fredrickson* Types IIa and IIb)
14.3 Hypertriglyceridemia *(Fredrickson* Type IV)
14.4 Dysbetalipoproteinemia *(Fredrickson* Type III)
14.5 Homozygous Familial Hypercholesterolemia
14.6 Heterozygous Familial Hypercholesterolemia in Pediatric Patients
15 REFERENCES
16 HOW SUPPLIED/STORAGE AND HANDLING
17 PATIENT COUNSELING INFORMATION
17.1 Muscle Pain
17.2 Liver Enzymes
17.3 Pregnancy
17.4 Breast-feeding

* Sections or subsections omitted from the full prescribing information are not listed.

FULL PRESCRIBING INFORMATION

1 INDICATIONS AND USAGE

Therapy with lipid-altering agents should be only one component of multiple risk factor intervention in individuals at significantly increased risk for atherosclerotic vascular disease due to hypercholesterolemia. Drug therapy is recommended as an adjunct to diet when the response to a diet restricted in saturated fat and cholesterol and other nonpharmacologic measures alone has been

inadequate. In patients with CHD or multiple risk factors for CHD, LIPITOR can be started simultaneously with diet.

1.1 Prevention of Cardiovascular Disease

In adult patients without clinically evident coronary heart disease, but with multiple risk factors for coronary heart disease such as age, smoking, hypertension, low HDL-C, or a family history of early coronary heart disease, LIPITOR is indicated to:

- Reduce the risk of myocardial infarction
- Reduce the risk of stroke
- Reduce the risk for revascularization procedures and angina

In patients with type 2 diabetes, and without clinically evident coronary heart disease, but with multiple risk factors for coronary heart disease such as retinopathy, albuminuria, smoking, or hypertension, LIPITOR is indicated to:

- Reduce the risk of myocardial infarction
- Reduce the risk of stroke

In patients with clinically evident coronary heart disease, LIPITOR is indicated to:

- Reduce the risk of non-fatal myocardial infarction
- Reduce the risk of fatal and non-fatal stroke
- Reduce the risk for revascularization procedures
- Reduce the risk of hospitalization for CHF
- Reduce the risk of angina

1.2 Hyperlipidemia

LIPITOR is indicated:

- As an adjunct to diet to reduce elevated total-C, LDL-C, apo B, and TG levels and to increase HDL-C in patients with primary hypercholesterolemia (heterozygous familial and nonfamilial) and mixed dyslipidemia *(Fredrickson* Types IIa and IIb);
- As an adjunct to diet for the treatment of patients with elevated serum TG levels *(Fredrickson* Type IV);
- For the treatment of patients with primary dysbetalipoproteinemia *(Fredrickson* Type III) who do not respond adequately to diet;
- To reduce total-C and LDL-C in patients with homozygous familial hypercholesterolemia as an adjunct to other lipid-lowering treatments (e.g., LDL apheresis) or if such treatments are unavailable;
- As an adjunct to diet to reduce total-C, LDL-C, and apo B levels in boys and postmenarchal girls, 10 to 17 years of age, with heterozygous familial hypercholesterolemia if after an adequate trial of diet therapy the following findings are present:
  a. LDL-C remains ≥ 190 mg/dL or
  b. LDL-C remains ≥ 160 mg/dL and:
     - there is a positive family history of premature cardiovascular disease or
     - two or more other CVD risk factors are present in the pediatric patient

1.3 Limitations of Use

LIPITOR has not been studied in conditions where the major lipoprotein abnormality is elevation of chylomicrons *(Fredrickson* Types I and V).

2 DOSAGE AND ADMINISTRATION

2.1 Hyperlipidemia (Heterozygous Familial and Nonfamilial) and Mixed Dyslipidemia *(Fredrickson*

Types IIa and IIb)

The recommended starting dose of LIPITOR is 10 or 20 mg once daily. Patients who require a large reduction in LDL-C (more than 45%) may be started at 40 mg once daily. The dosage range of LIPITOR is 10 to 80 mg once daily. LIPITOR can be administered as a single dose at any time of the day, with or without food. The starting dose and maintenance doses of LIPITOR should be individualized according to patient characteristics such as goal of therapy and response (see current *NCEP Guidelines*). After initiation and/or upon titration of LIPITOR, lipid levels should be analyzed within 2 to 4 weeks and dosage adjusted accordingly.

2.2 Heterozygous Familial Hypercholesterolemia in Pediatric Patients (10–17 years of age)

The recommended starting dose of LIPITOR is 10 mg/day; the maximum recommended dose is 20 mg/day (doses greater than 20 mg have not been studied in this patient population). Doses should be individualized according to the recommended goal of therapy [see current *NCEP Pediatric Panel Guidelines, Clinical Pharmacology (12),* and *Indications and Usage (1.2)*]. Adjustments should be made at intervals of 4 weeks or more.

2.3 Homozygous Familial Hypercholesterolemia

The dosage of LIPITOR in patients with homozygous FH is 10 to 80 mg daily. LIPITOR should be used as an adjunct to other lipid-lowering treatments (e.g., LDL apheresis) in these patients or if such treatments are unavailable.

2.4 Concomitant Lipid-Lowering Therapy

LIPITOR may be used with bile acid resins. The combination of HMG-CoA reductase inhibitors (statins) and fibrates should generally be used with caution [see *Warnings and Precautions, Skeletal Muscle (5.1), Drug Interactions (7)*].

2.5 Dosage in Patients With Renal Impairment

Renal disease does not affect the plasma concentrations nor LDL-C reduction of LIPITOR; thus, dosage adjustment in patients with renal dysfunction is not necessary [see *Warnings and Precautions, Skeletal Muscle (5.1), Clinical Pharmacology, Pharmacokinetics (12.3)*].

2.6 Dosage in Patients Taking Cyclosporine, Clarithromycin, Itraconazole, or Certain Protease Inhibitors

In patients taking cyclosporine or the HIV protease inhibitors (tipranavir plus ritonavir) or the hepatitis C protease inhibitor (telaprevir), therapy with LIPITOR should be avoided. In patients with HIV taking lopinavir plus ritonavir, caution should be used when prescribing LIPITOR and the lowest dose necessary employed. In patients taking clarithromycin, itraconazole, or in patients with HIV taking a combination of saquinavir plus ritonavir, darunavir plus ritonavir, fosamprenavir, or fosamprenavir plus ritonavir, therapy with LIPITOR should be limited to 20 mg, and appropriate clinical assessment is recommended to ensure that the lowest dose necessary of LIPITOR is employed. In patients taking the HIV protease inhibitor nelfinavir or the hepatitis C protease inhibitor boceprevir, therapy with LIPITOR should be limited to 40 mg, and appropriate clinical assessment is recommended to ensure that the lowest dose necessary of LIPITOR is employed [see *Warnings and Precautions, Skeletal Muscle (5.1), Drug Interactions (7)*].

3 DOSAGE FORMS AND STRENGTHS

White, elliptical, film-coated tablets containing 10, 20, 40, and 80 mg atorvastatin calcium.

4 CONTRAINDICATIONS

4.1 Active liver disease, which may include unexplained persistent elevations in hepatic transaminase levels

4.2 Hypersensitivity to any component of this medication

4.3 Pregnancy

Women who are pregnant or may become pregnant. LIPITOR may cause fetal harm when administered to a pregnant woman. Serum cholesterol and triglycerides increase during normal pregnancy, and cholesterol or cholesterol derivatives are essential for fetal development. Atherosclerosis is a chronic process and discontinuation of lipid-lowering drugs during pregnancy should have little impact on the outcome of long-term therapy of primary hypercholesterolemia. There are no adequate and well-controlled studies of LIPITOR use during pregnancy; however in rare reports, congenital anomalies were observed following intrauterine exposure to statins. In rat and rabbit animal reproduction studies, atorvastatin revealed no evidence of teratogenicity. LIPITOR SHOULD BE ADMINISTERED TO WOMEN OF CHILDBEARING AGE ONLY WHEN SUCH PATIENTS ARE HIGHLY UNLIKELY TO CONCEIVE AND HAVE BEEN INFORMED OF THE POTENTIAL HAZARDS. If the patient becomes pregnant while taking this drug, LIPITOR should be discontinued immediately and the patient apprised of the potential hazard to the fetus [see *Use in Specific Populations (8.1)*].

4.4 Nursing mothers

It is not known whether atorvastatin is excreted into human milk; however a small amount of another drug in this class does pass into breast milk. Because statins have the potential for serious adverse reactions in nursing infants, women who require LIPITOR treatment should not breastfeed their infants [see *Use in Specific Populations (8.3)*].

5 WARNINGS AND PRECAUTIONS

5.1 Skeletal Muscle

Rare cases of rhabdomyolysis with acute renal failure secondary to myoglobinuria have been reported with LIPITOR and with other drugs in this class. A history of renal impairment may be a risk factor for the development of rhabdomyolysis. Such patients merit closer monitoring for skeletal muscle effects.

Atorvastatin, like other statins, occasionally causes myopathy, defined as muscle aches or muscle weakness in conjunction with increases in creatine phosphokinase (CPK) values >10 times ULN. The concomitant use of higher doses of atorvastatin with certain drugs such as cyclosporine and strong CYP3A4 inhibitors (e.g., clarithromycin, itraconazole, and HIV protease inhibitors) increases the risk of myopathy/rhabdomyolysis.

There have been rare reports of immune-mediated necrotizing myopathy (IMNM), an autoimmune myopathy, associated with statin use. IMNM is characterized by: proximal muscle weakness and elevated serum creatine kinase, which persist despite discontinuation of statin treatment; muscle biopsy showing necrotizing myopathy without significant inflammation; improvement with immunosuppressive agents.

Myopathy should be considered in any patient with diffuse myalgias, muscle tenderness or weakness, and/or marked elevation of CPK. Patients should be advised to report promptly unexplained muscle pain, tenderness, or weakness, particularly if accompanied by malaise or fever or if muscle signs and symptoms persist after discontinuing LIPITOR. LIPITOR therapy should be discontinued if markedly elevated CPK levels occur or myopathy is diagnosed or suspected.

The risk of myopathy during treatment with drugs in this class is increased with concurrent administration of cyclosporine, fibric acid derivatives, erythromycin, clarithromycin, the hepatitis C protease inhibitor telaprevir, combinations of HIV protease inhibitors, including saquinavir plus

ritonavir, lopinavir plus ritonavir, tipranavir plus ritonavir, darunavir plus ritonavir, fosamprenavir, and fosamprenavir plus ritonavir, niacin, or azole antifungals. Physicians considering combined therapy with LIPITOR and fibric acid derivatives, erythromycin, clarithromycin, a combination of saquinavir plus ritonavir, lopinavir plus ritonavir, darunavir plus ritonavir, fosamprenavir, or fosamprenavir plus ritonavir, azole antifungals, or lipid-modifying doses of niacin should carefully weigh the potential benefits and risks and should carefully monitor patients for any signs or symptoms of muscle pain, tenderness, or weakness, particularly during the initial months of therapy and during any periods of upward dosage titration of either drug. Lower starting and maintenance doses of atorvastatin should be considered when taken concomitantly with the aforementioned drugs (see *Drug Interactions (7)*). Periodic creatine phosphokinase (CPK) determinations may be considered in such situations, but there is no assurance that such monitoring will prevent the occurrence of severe myopathy.

Prescribing recommendations for interacting agents are summarized in Table 1 [see also *Dosage and Administration (2.6), Drug Interactions (7), Clinical Pharmacology (12.3)*].

Table 1. Drug Interactions Associated with Increased Risk of Myopathy/Rhabdomyolysis

Interacting Agents	Prescribing Recommendations
Cyclosporine, HIV protease inhibitors (tipranavir plus ritonavir), hepatitis C protease inhibitor (telaprevir)	Avoid atorvastatin
HIV protease inhibitor (lopinavir plus ritonavir)	Use with caution and lowest dose necessary
Clarithromycin, itraconazole, HIV protease inhibitors (saquinavir plus ritonavir*, darunavir plus ritonavir, fosamprenavir, fosamprenavir plus ritonavir)	Do not exceed 20 mg atorvastatin daily
HIV protease inhibitor (nelfinavir) Hepatitis C protease inhibitor (boceprevir)	Do not exceed 40 mg atorvastatin daily

* Use with caution and with the lowest dose necessary (12.3)

Cases of myopathy, including rhabdomyolysis, have been reported with atorvastatin co-administered with colchicine, and caution should be exercised when prescribing atorvastatin with colchicine [see *Drug Interactions (7.11)*].

LIPITOR therapy should be temporarily withheld or discontinued in any patient with an acute, serious condition suggestive of a myopathy or having a risk factor predisposing to the development of renal failure secondary to rhabdomyolysis (e.g., severe acute infection, hypotension, major surgery, trauma, severe metabolic, endocrine and electrolyte disorders, and uncontrolled seizures).

5.2 Liver Dysfunction

Statins, like some other lipid-lowering therapies, have been associated with biochemical abnormalities of liver function. **Persistent elevations (>3 times the upper limit of normal [ULN] occurring on 2 or more occasions) in serum transaminases occurred in 0.7% of patients who received LIPITOR in clinical trials. The incidence of these abnormalities was 0.2%, 0.2%, 0.6%, and 2.3% for 10, 20, 40, and 80 mg, respectively.**

One patient in clinical trials developed jaundice. Increases in liver function tests (LFT) in other patients were not associated with jaundice or other clinical signs or symptoms. Upon dose reduction, drug interruption, or discontinuation, transaminase levels returned to or near pretreatment levels without

sequelae. Eighteen of 30 patients with persistent LFT elevations continued treatment with a reduced dose of LIPITOR.

It is recommended that liver enzyme tests be obtained prior to initiating therapy with LIPITOR and repeated as clinically indicated. There have been rare postmarketing reports of fatal and non-fatal hepatic failure in patients taking statins, including atorvastatin. If serious liver injury with clinical symptoms and/or hyperbilirubinemia or jaundice occurs during treatment with LIPITOR, promptly interrupt therapy. If an alternate etiology is not found, do not restart LIPITOR.

LIPITOR should be used with caution in patients who consume substantial quantities of alcohol and/or have a history of liver disease. Active liver disease or unexplained persistent transaminase elevations are contraindications to the use of LIPITOR [see *Contraindications (4.1)*].

5.3 Endocrine Function

Increases in HbA1c and fasting serum glucose levels have been reported with HMG-CoA reductase inhibitors, including LIPITOR.

Statins interfere with cholesterol synthesis and theoretically might blunt adrenal and/or gonadal steroid production. Clinical studies have shown that LIPITOR does not reduce basal plasma cortisol concentration or impair adrenal reserve. The effects of statins on male fertility have not been studied in adequate numbers of patients. The effects, if any, on the pituitary-gonadal axis in premenopausal women are unknown. Caution should be exercised if a statin is administered concomitantly with drugs that may decrease the levels or activity of endogenous steroid hormones, such as ketoconazole, spironolactone, and cimetidine.

5.4 CNS Toxicity

Brain hemorrhage was seen in a female dog treated for 3 months at 120 mg/kg/day. Brain hemorrhage and optic nerve vacuolation were seen in another female dog that was sacrificed in moribund condition after 11 weeks of escalating doses up to 280 mg/kg/day. The 120 mg/kg dose resulted in a systemic exposure approximately 16 times the human plasma area-under-the-curve (AUC, 0–24 hours) based on the maximum human dose of 80 mg/day. A single tonic convulsion was seen in each of 2 male dogs (one treated at 10 mg/kg/day and one at 120 mg/kg/day) in a 2-year study. No CNS lesions have been observed in mice after chronic treatment for up to 2 years at doses up to 400 mg/kg/day or in rats at doses up to 100 mg/kg/day. These doses were 6 to 11 times (mouse) and 8 to 16 times (rat) the human AUC (0–24) based on the maximum recommended human dose of 80 mg/day.

CNS vascular lesions, characterized by perivascular hemorrhages, edema, and mononuclear cell infiltration of perivascular spaces, have been observed in dogs treated with other members of this class. A chemically similar drug in this class produced optic nerve degeneration (Wallerian degeneration of retinogeniculate fibers) in clinically normal dogs in a dose-dependent fashion at a dose that produced plasma drug levels about 30 times higher than the mean drug level in humans taking the highest recommended dose.

5.5 Use in Patients with Recent Stroke or TIA

In a post-hoc analysis of the Stroke Prevention by Aggressive Reduction in Cholesterol Levels (SPARCL) study where LIPITOR 80 mg vs. placebo was administered in 4,731 subjects without CHD who had a stroke or TIA within the preceding 6 months, a higher incidence of hemorrhagic stroke was seen in the LIPITOR 80 mg group compared to placebo (55, 2.3% atorvastatin vs. 33, 1.4% placebo; HR: 1.68, 95% CI: 1.09, 2.59; p=0.0168). The incidence of fatal hemorrhagic stroke was similar across treatment groups (17 vs. 18 for the atorvastatin and placebo groups, respectively). The incidence of nonfatal hemorrhagic stroke was significantly higher in the atorvastatin group (38, 1.6%) as compared to the placebo group (16, 0.7%). Some baseline characteristics, including hemorrhagic and lacunar stroke on study entry, were associated with a higher incidence of hemorrhagic stroke in the atorvastatin group [see *Adverse Reactions (6.1)*].

6 ADVERSE REACTIONS

The following serious adverse reactions are discussed in greater detail in other sections of the label:

Rhabdomyolysis and myopathy [see *Warnings and Precautions (5.1)*]

Liver enzyme abnormalities [see *Warnings and Precautions (5.2)*]

6.1 Clinical Trial Adverse Experiences

Because clinical trials are conducted under widely varying conditions, the adverse reaction rates observed in the clinical studies of a drug cannot be directly compared to rates in the clinical trials of another drug and may not reflect the rates observed in clinical practice.

In the LIPITOR placebo-controlled clinical trial database of 16,066 patients (8755 LIPITOR vs. 7311 placebo; age range 10–93 years, 39% women, 91% Caucasians, 3% Blacks, 2% Asians, 4% other) with a median treatment duration of 53 weeks, 9.7% of patients on LIPITOR and 9.5% of the patients on placebo discontinued due to adverse reactions regardless of causality. The five most common adverse reactions in patients treated with LIPITOR that led to treatment discontinuation and occurred at a rate greater than placebo were: myalgia (0.7%), diarrhea (0.5%), nausea (0.4%), alanine aminotransferase increase (0.4%), and hepatic enzyme increase (0.4%).

The most commonly reported adverse reactions (incidence ≥ 2% and greater than placebo) regardless of causality, in patients treated with LIPITOR in placebo controlled trials (n=8755) were: nasopharyngitis (8.3%), arthralgia (6.9%), diarrhea (6.8%), pain in extremity (6.0%), and urinary tract infection (5.7%).

Table 2 summarizes the frequency of clinical adverse reactions, regardless of causality, reported in ≥ 2% and at a rate greater than placebo in patients treated with LIPITOR (n=8755), from seventeen placebo-controlled trials.

Table 2. Clinical adverse reactions occurring in ≥ 2% in patents treated with any dose of LIPITOR and at an incidence greater than placebo regardless of causality (% of patients).

Adverse Reaction*	Any dose N=8755	10 mg N=3908	20 mg N=188	40 mg N=604	80 mg N=4055	Placebo N=7311
Nasopharyngitis	8.3	12.9	5.3	7.0	4.2	8.2
Arthralgia	6.9	8.9	11.7	10.6	4.3	6.5
Diarrhea	6.8	7.3	6.4	14.1	5.2	6.3
Pain in extremity	6.0	8.5	3.7	9.3	3.1	5.9
Urinary tract infection	5.7	6.9	6.4	8.0	4.1	5.6
Dyspepsia	4.7	5.9	3.2	6.0	3.3	4.3
Nausea	4.0	3.7	3.7	7.1	3.8	3.5
Musculoskeletal pain	3.8	5.2	3.2	5.1	2.3	3.6
Muscle Spasms	3.6	4.6	4.8	5.1	2.4	3.0
Myalgia	3.5	3.6	5.9	8.4	2.7	3.1
Insomnia	3.0	2.8	1.1	5.3	2.8	2.9
Pharyngolaryngeal pain	2.3	3.9	1.6	2.8	0.7	2.1

* Adverse Reaction ≥ 2% in any dose greater than placebo

Other adverse reactions reported in placebo-controlled studies include:

Body as a whole: malaise, pyrexia; *Digestive system:* abdominal discomfort, eructation, flatulence, hepatitis, cholestasis; *Musculoskeletal system*: musculoskeletal pain, muscle fatigue, neck pain, joint

swelling; *Metabolic and nutritional system*: transaminases increase, liver function test abnormal, blood alkaline phosphatase increase, creatine phosphokinase increase, hyperglycemia; *Nervous system*: nightmare; *Respiratory system:* epistaxis; *Skin and appendages*: urticaria; *Special senses*: vision blurred, tinnitus; *Urogenital system:* white blood cells urine positive.

Anglo-Scandinavian Cardiac Outcomes Trial (ASCOT)

In ASCOT [see *Clinical Studies (14.1)*] involving 10,305 participants (age range 40–80 years, 19% women; 94.6% Caucasians, 2.6% Africans, 1.5% South Asians, 1.3% mixed/other) treated with LIPITOR 10 mg daily (n=5,168) or placebo (n=5,137), the safety and tolerability profile of the group treated with LIPITOR was comparable to that of the group treated with placebo during a median of 3.3 years of follow-up.

Collaborative Atorvastatin Diabetes Study (CARDS)

In CARDS [see *Clinical Studies (14.1)*] involving 2,838 subjects (age range 39–77 years, 32% women; 94.3% Caucasians, 2.4% South Asians, 2.3% Afro-Caribbean, 1.0% other) with type 2 diabetes treated with LIPITOR 10 mg daily (n=1,428) or placebo (n=1,410), there was no difference in the overall frequency of adverse reactions or serious adverse reactions between the treatment groups during a median follow-up of 3.9 years. No cases of rhabdomyolysis were reported.

Treating to New Targets Study (TNT)

In TNT [see *Clinical Studies (14.1)*] involving 10,001 subjects (age range 29–78 years, 19% women; 94.1% Caucasians, 2.9% Blacks, 1.0% Asians, 2.0% other) with clinically evident CHD treated with LIPITOR 10 mg daily (n=5006) or LIPITOR 80 mg daily (n=4995), there were more serious adverse reactions and discontinuations due to adverse reactions in the high-dose atorvastatin group (92, 1.8%; 497, 9.9%, respectively) as compared to the low-dose group (69, 1.4%; 404, 8.1%, respectively) during a median follow-up of 4.9 years. Persistent transaminase elevations (≥3 × ULN twice within 4–10 days) occurred in 62 (1.3%) individuals with atorvastatin 80 mg and in nine (0.2%) individuals with atorvastatin 10 mg. Elevations of CK (≥ 10 × ULN) were low overall, but were higher in the high-dose atorvastatin treatment group (13, 0.3%) compared to the low-dose atorvastatin group (6, 0.1%).

Incremental Decrease in Endpoints through Aggressive Lipid Lowering Study (IDEAL)

In IDEAL [see *Clinical Studies (14.1)*] involving 8,888 subjects (age range 26–80 years, 19% women; 99.3% Caucasians, 0.4% Asians, 0.3% Blacks, 0.04% other) treated with LIPITOR 80 mg/day (n=4439) or simvastatin 20–40 mg daily (n=4449), there was no difference in the overall frequency of adverse reactions or serious adverse reactions between the treatment groups during a median follow-up of 4.8 years.

Stroke Prevention by Aggressive Reduction in Cholesterol Levels (SPARCL)

In SPARCL involving 4731 subjects (age range 21–92 years, 40% women; 93.3% Caucasians, 3.0% Blacks, 0.6% Asians, 3.1% other) without clinically evident CHD but with a stroke or transient ischemic attack (TIA) within the previous 6 months treated with LIPITOR 80 mg (n=2365) or placebo (n=2366) for a median follow-up of 4.9 years, there was a higher incidence of persistent hepatic transaminase elevations (≥ 3 × ULN twice within 4–10 days) in the atorvastatin group (0.9%) compared to placebo (0.1%). Elevations of CK (>10 × ULN) were rare, but were higher in the atorvastatin group (0.1%) compared to placebo (0.0%). Diabetes was reported as an adverse reaction in 144 subjects (6.1%) in the atorvastatin group and 89 subjects (3.8%) in the placebo group [see *Warnings and Precautions (5.5)*].

In a post-hoc analysis, LIPITOR 80 mg reduced the incidence of ischemic stroke (218/2365, 9.2% vs. 274/2366, 11.6%) and increased the incidence of hemorrhagic stroke (55/2365, 2.3% vs. 33/2366, 1.4%) compared to placebo. The incidence of fatal hemorrhagic stroke was similar between groups (17 LIPITOR vs. 18 placebo). The incidence of non-fatal hemorrhagic strokes was significantly greater in the atorvastatin group (38 non-fatal hemorrhagic strokes) as compared to the placebo group (16 non-fatal hemorrhagic strokes). Subjects who entered the study with a hemorrhagic stroke appeared to be at increased risk for hemorrhagic stroke [7 (16%) LIPITOR vs. 2 (4%) placebo].

There were no significant differences between the treatment groups for all-cause mortality: 216 (9.1%) in the LIPITOR 80 mg/day group vs. 211 (8.9%) in the placebo group. The proportions of subjects who experienced cardiovascular death were numerically smaller in the LIPITOR 80 mg group (3.3%) than in the placebo group (4.1%). The proportions of subjects who experienced non-cardiovascular death were numerically larger in the LIPITOR 80 mg group (5.0%) than in the placebo group (4.0%).

6.2 Postmarketing Experience

The following adverse reactions have been identified during postapproval use of LIPITOR. Because these reactions are reported voluntarily from a population of uncertain size, it is not always possible to reliably estimate their frequency or establish a causal relationship to drug exposure.

Adverse reactions associated with LIPITOR therapy reported since market introduction, that are not listed above, regardless of causality assessment, include the following: anaphylaxis, angioneurotic edema, bullous rashes (including erythema multiforme, Stevens-Johnson syndrome, and toxic epidermal necrolysis), rhabdomyolysis, myositis, fatigue, tendon rupture, fatal and non-fatal hepatic failure, dizziness, depression, peripheral neuropathy, and pancreatitis.

There have been rare reports of immune-mediated necrotizing myopathy associated with statin use [see *Warnings and Precautions (5.1)*].

There have been rare postmarketing reports of cognitive impairment (e.g., memory loss, forgetfulness, amnesia, memory impairment, confusion) associated with statin use. These cognitive issues have been reported for all statins. The reports are generally nonserious, and reversible upon statin discontinuation, with variable times to symptom onset (1 day to years) and symptom resolution (median of 3 weeks).

6.3 Pediatric Patients (ages 10–17 years)

In a 26-week controlled study in boys and postmenarchal girls (n=140, 31% female; 92% Caucasians, 1.6% Blacks, 1.6% Asians, 4.8% other), the safety and tolerability profile of LIPITOR 10 to 20 mg daily was generally similar to that of placebo [see *Clinical Studies (14.6)* and *Use in Special Populations, Pediatric Use (8.4)*].

7 DRUG INTERACTIONS

The risk of myopathy during treatment with statins is increased with concurrent administration of fibric acid derivatives, lipid-modifying doses of niacin, cyclosporine, or strong CYP 3A4 inhibitors (e.g., clarithromycin, HIV protease inhibitors, and itraconazole) [see *Warnings and Precautions, Skeletal Muscle (5.1)* and *Clinical Pharmacology (12.3)*].

7.1 Strong Inhibitors of CYP 3A4

LIPITOR is metabolized by cytochrome P450 3A4. Concomitant administration of LIPITOR with strong inhibitors of CYP 3A4 can lead to increases in plasma concentrations of atorvastatin. The extent of interaction and potentiation of effects depend on the variability of effect on CYP 3A4.

Clarithromycin: Atorvastatin AUC was significantly increased with concomitant administration of LIPITOR 80 mg with clarithromycin (500 mg twice daily) compared to that of LIPITOR alone [see *Clinical Pharmacology (12.3)*]. Therefore, in patients taking clarithromycin, caution should be used when the LIPITOR dose exceeds 20 mg [see *Warnings and Precautions, Skeletal Muscle (5.1)* and *Dosage and Administration (2.6)*].

Combination of Protease Inhibitors: Atorvastatin AUC was significantly increased with concomitant administration of LIPITOR with several combinations of HIV protease inhibitors, as well as with the hepatitis C protease inhibitor telaprevir, compared to that of LIPITOR alone [see *Clinical Pharmacology (12.3)*]. Therefore, in patients taking the HIV protease inhibitor tipranavir

plus ritonavir, or the hepatitis C protease inhibitor telaprevir, concomitant use of LIPITOR should be avoided. In patients taking the HIV protease inhibitor lopinavir plus ritonavir, caution should be used when prescribing LIPITOR and the lowest dose necessary should be used. In patients taking the HIV protease inhibitors saquinavir plus ritonavir, darunavir plus ritonavir, fosamprenavir, or fosamprenavir plus ritonavir, the dose of LIPITOR should not exceed 20 mg and should be used with caution [see *Warnings and Precautions, Skeletal Muscle (5.1)* and *Dosage and Administration (2.6)*]. In patients taking the HIV protease inhibitor nelfinavir or the hepatitis C protease inhibitor boceprevir, the dose of LIPITOR should not exceed 40 mg and close clinical monitoring is recommended.

Itraconazole: Atorvastatin AUC was significantly increased with concomitant administration of LIPITOR 40 mg and itraconazole 200 mg [see *Clinical Pharmacology (12.3)*]. Therefore, in patients taking itraconazole, caution should be used when the LIPITOR dose exceeds 20 mg [see *Warnings and Precautions, Skeletal Muscle (5.1)* and *Dosage and Administration (2.6)*].

7.2 Grapefruit Juice

Contains one or more components that inhibit CYP 3A4 and can increase plasma concentrations of atorvastatin, especially with excessive grapefruit juice consumption (>1.2 liters per day).

7.3 Cyclosporine

Atorvastatin and atorvastatin-metabolites are substrates of the OATP1B1 transporter. Inhibitors of the OATP1B1 (e.g., cyclosporine) can increase the bioavailability of atorvastatin. Atorvastatin AUC was significantly increased with concomitant administration of LIPITOR 10 mg and cyclosporine 5.2 mg/kg/day compared to that of LIPITOR alone [see *Clinical Pharmacology (12.3)*]. The co-administration of LIPITOR with cyclosporine should be avoided [see *Warnings and Precautions, Skeletal Muscle (5.1)*].

7.4 Gemfibrozil

Due to an increased risk of myopathy/rhabdomyolysis when HMG-CoA reductase inhibitors are co-administered with gemfibrozil, concomitant administration of LIPITOR with gemfibrozil should be avoided [see *Warnings and Precautions (5.1)*].

7.5 Other Fibrates

Because it is known that the risk of myopathy during treatment with HMG-CoA reductase inhibitors is increased with concurrent administration of other fibrates, LIPITOR should be administered with caution when used concomitantly with other fibrates [see *Warnings and Precautions (5.1)*].

7.6 Niacin

The risk of skeletal muscle effects may be enhanced when LIPITOR is used in combination with niacin; a reduction in LIPITOR dosage should be considered in this setting [see *Warnings and Precautions (5.1)*].

7.7 Rifampin or other Inducers of Cytochrome P450 3A4

Concomitant administration of LIPITOR with inducers of cytochrome P450 3A4 (e.g., efavirenz, rifampin) can lead to variable reductions in plasma concentrations of atorvastatin. Due to the dual interaction mechanism of rifampin, simultaneous co-administration of LIPITOR with rifampin is recommended, as delayed administration of LIPITOR after administration of rifampin has been associated with a significant reduction in atorvastatin plasma concentrations.

7.8 Digoxin

When multiple doses of LIPITOR and digoxin were co-administered, steady state plasma digoxin concentrations increased by approximately 20%. Patients taking digoxin should be monitored

appropriately.

7.9 Oral Contraceptives

Co-administration of LIPITOR and an oral contraceptive increased AUC values for norethindrone and ethinyl estradiol [see *Clinical Pharmacology (12.3)*]. These increases should be considered when selecting an oral contraceptive for a woman taking LIPITOR.

7.10 Warfarin

LIPITOR had no clinically significant effect on prothrombin time when administered to patients receiving chronic warfarin treatment.

7.11 Colchicine

Cases of myopathy, including rhabdomyolysis, have been reported with atorvastatin co-administered with colchicine, and caution should be exercised when prescribing atorvastatin with colchicine.

8 USE IN SPECIFIC POPULATIONS

8.1 Pregnancy

Pregnancy Category X

LIPITOR is contraindicated in women who are or may become pregnant. Serum cholesterol and triglycerides increase during normal pregnancy. Lipid lowering drugs offer no benefit during pregnancy because cholesterol and cholesterol derivatives are needed for normal fetal development. Atherosclerosis is a chronic process, and discontinuation of lipid-lowering drugs during pregnancy should have little impact on long-term outcomes of primary hypercholesterolemia therapy.

There are no adequate and well-controlled studies of atorvastatin use during pregnancy. There have been rare reports of congenital anomalies following intrauterine exposure to statins. In a review of about 100 prospectively followed pregnancies in women exposed to other statins, the incidences of congenital anomalies, spontaneous abortions, and fetal deaths/stillbirths did not exceed the rate expected in the general population. However, this study was only able to exclude a three-to-four-fold increased risk of congenital anomalies over background incidence. In 89% of these cases, drug treatment started before pregnancy and stopped during the first trimester when pregnancy was identified.

Atorvastatin crosses the rat placenta and reaches a level in fetal liver equivalent to that of maternal plasma. Atorvastatin was not teratogenic in rats at doses up to 300 mg/kg/day or in rabbits at doses up to 100 mg/kg/day. These doses resulted in multiples of about 30 times (rat) or 20 times (rabbit) the human exposure based on surface area (mg/m^2) [see *Contraindications, Pregnancy (4.3)*].

In a study in rats given 20, 100, or 225 mg/kg/day, from gestation day 7 through to lactation day 21 (weaning), there was decreased pup survival at birth, neonate, weaning, and maturity in pups of mothers dosed with 225 mg/kg/day. Body weight was decreased on days 4 and 21 in pups of mothers dosed at 100 mg/kg/day; pup body weight was decreased at birth and at days 4, 21, and 91 at 225 mg/kg/day. Pup development was delayed (rotorod performance at 100 mg/kg/day and acoustic startle at 225 mg/kg/day; pinnae detachment and eye-opening at 225 mg/kg/day). These doses correspond to 6 times (100 mg/kg) and 22 times (225 mg/kg) the human AUC at 80 mg/day.

Statins may cause fetal harm when administered to a pregnant woman. LIPITOR should be administered to women of childbearing potential only when such patients are highly unlikely to conceive and have been informed of the potential hazards. If the woman becomes pregnant while taking LIPITOR, it should be discontinued immediately and the patient advised again as to the potential hazards to the fetus and the lack of known clinical benefit with continued use during pregnancy.

8.3 Nursing Mothers

It is not known whether atorvastatin is excreted in human milk, but a small amount of another drug in this class does pass into breast milk. Nursing rat pups had plasma and liver drug levels of 50% and 40%, respectively, of that in their mother's milk. Animal breast milk drug levels may not accurately reflect human breast milk levels. Because another drug in this class passes into human milk and because statins have a potential to cause serious adverse reactions in nursing infants, women requiring LIPITOR treatment should be advised not to nurse their infants [see *Contraindications (4)*].

8.4 Pediatric Use

Safety and effectiveness in patients 10–17 years of age with heterozygous familial hypercholesterolemia have been evaluated in a controlled clinical trial of 6 months' duration in adolescent boys and postmenarchal girls. Patients treated with LIPITOR had an adverse experience profile generally similar to that of patients treated with placebo. The most common adverse experiences observed in both groups, regardless of causality assessment, were infections. **Doses greater than 20 mg have not been studied in this patient population.** In this limited controlled study, there was no significant effect on growth or sexual maturation in boys or on menstrual cycle length in girls [see *Clinical Studies (14.6); Adverse Reactions, Pediatric Patients (ages 10–17 years) (6.3)*; and *Dosage and Administration, Heterozygous Familial Hypercholesterolemia in Pediatric Patients (10–17 years of age) (2.2)*]. Adolescent females should be counseled on appropriate contraceptive methods while on LIPITOR therapy [see *Contraindications, Pregnancy (4.3)* and *Use in Specific Populations, Pregnancy (8.1)*]. **LIPITOR has not been studied in controlled clinical trials involving pre-pubertal patients or patients younger than 10 years of age.**

Clinical efficacy with doses up to 80 mg/day for 1 year have been evaluated in an uncontrolled study of patients with homozygous FH including 8 pediatric patients [see *Clinical Studies, Homozygous Familial Hypercholesterolemia (14.5)*].

8.5 Geriatric Use

Of the 39,828 patients who received LIPITOR in clinical studies, 15,813 (40%) were ≥65 years old and 2,800 (7%) were ≥75 years old. No overall differences in safety or effectiveness were observed between these subjects and younger subjects, and other reported clinical experience has not identified differences in responses between the elderly and younger patients, but greater sensitivity of some older adults cannot be ruled out. Since advanced age (≥65 years) is a predisposing factor for myopathy, LIPITOR should be prescribed with caution in the elderly.

8.6 Hepatic Impairment

Lipitor is contraindicated in patients with active liver disease which may include unexplained persistent elevations in hepatic transaminase levels [see *Contraindications (4)* and *Pharmacokinetics (12.3)*].

10 OVERDOSAGE

There is no specific treatment for LIPITOR overdosage. In the event of an overdose, the patient should be treated symptomatically, and supportive measures instituted as required. Due to extensive drug binding to plasma proteins, hemodialysis is not expected to significantly enhance LIPITOR clearance.

11 DESCRIPTION

LIPITOR is a synthetic lipid-lowering agent. Atorvastatin is an inhibitor of 3-hydroxy-3-methylglutaryl-coenzyme A (HMG-CoA) reductase. This enzyme catalyzes the conversion of HMG-CoA to mevalonate, an early and rate-limiting step in cholesterol biosynthesis.

Atorvastatin calcium is [R-(R*, R*)]-2-(4-fluorophenyl)-ß, δ-dihydroxy-5-(1-methylethyl)-3-phenyl-4-[(phenylamino)carbonyl]-1H-pyrrole-1-heptanoic acid, calcium salt (2:1) trihydrate. The empirical formula of atorvastatin calcium is $(C_{33}H_{34}FN_2O_5)_2Ca \cdot 3H_2O$ and its molecular weight is 1209.42. Its

structural formula is:

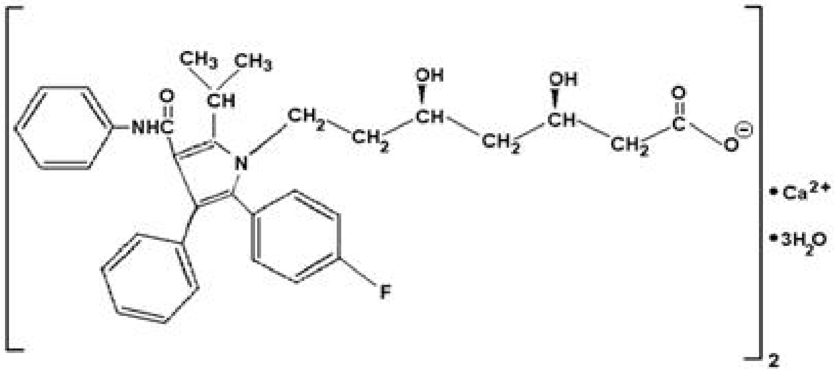

Atorvastatin calcium is a white to off-white crystalline powder that is insoluble in aqueous solutions of pH 4 and below. Atorvastatin calcium is very slightly soluble in distilled water, pH 7.4 phosphate buffer, and acetonitrile; slightly soluble in ethanol; and freely soluble in methanol.

LIPITOR Tablets for oral administration contain 10, 20, 40, or 80 mg atorvastatin and the following inactive ingredients: calcium carbonate, USP; candelilla wax, FCC; croscarmellose sodium, NF; hydroxypropyl cellulose, NF; lactose monohydrate, NF; magnesium stearate, NF; microcrystalline cellulose, NF; Opadry White YS-1-7040 (hypromellose, polyethylene glycol, talc, titanium dioxide); polysorbate 80, NF; simethicone emulsion.

12 CLINICAL PHARMACOLOGY

12.1 Mechanism of Action

LIPITOR is a selective, competitive inhibitor of HMG-CoA reductase, the rate-limiting enzyme that converts 3-hydroxy-3-methylglutaryl-coenzyme A to mevalonate, a precursor of sterols, including cholesterol. Cholesterol and triglycerides circulate in the bloodstream as part of lipoprotein complexes. With ultracentrifugation, these complexes separate into HDL (high-density lipoprotein), IDL (intermediate-density lipoprotein), LDL (low-density lipoprotein), and VLDL (very-low-density lipoprotein) fractions. Triglycerides (TG) and cholesterol in the liver are incorporated into VLDL and released into the plasma for delivery to peripheral tissues. LDL is formed from VLDL and is catabolized primarily through the high-affinity LDL receptor. Clinical and pathologic studies show that elevated plasma levels of total cholesterol (total-C), LDL-cholesterol (LDL-C), and apolipoprotein B (apo B) promote human atherosclerosis and are risk factors for developing cardiovascular disease, while increased levels of HDL-C are associated with a decreased cardiovascular risk.

In animal models, LIPITOR lowers plasma cholesterol and lipoprotein levels by inhibiting HMG-CoA reductase and cholesterol synthesis in the liver and by increasing the number of hepatic LDL receptors on the cell surface to enhance uptake and catabolism of LDL; LIPITOR also reduces LDL production and the number of LDL particles. LIPITOR reduces LDL-C in some patients with homozygous familial hypercholesterolemia (FH), a population that rarely responds to other lipid-lowering medication(s).

A variety of clinical studies have demonstrated that elevated levels of total-C, LDL-C, and apo B (a membrane complex for LDL-C) promote human atherosclerosis. Similarly, decreased levels of HDL-C (and its transport complex, apo A) are associated with the development of atherosclerosis. Epidemiologic investigations have established that cardiovascular morbidity and mortality vary directly with the level of total-C and LDL-C, and inversely with the level of HDL-C.

LIPITOR reduces total-C, LDL-C, and apo B in patients with homozygous and heterozygous FH, nonfamilial forms of hypercholesterolemia, and mixed dyslipidemia. LIPITOR also reduces VLDL-C and TG and produces variable increases in HDL-C and apolipoprotein A-1. LIPITOR reduces total-C, LDL-C, VLDL-C, apo B, TG, and non-HDL-C, and increases HDL-C in patients with isolated hypertriglyceridemia. LIPITOR reduces intermediate density lipoprotein cholesterol (IDL-C) in

patients with dysbetalipoproteinemia.

Like LDL, cholesterol-enriched triglyceride-rich lipoproteins, including VLDL, intermediate density lipoprotein (IDL), and remnants, can also promote atherosclerosis. Elevated plasma triglycerides are frequently found in a triad with low HDL-C levels and small LDL particles, as well as in association with non-lipid metabolic risk factors for coronary heart disease. As such, total plasma TG has not consistently been shown to be an independent risk factor for CHD. Furthermore, the independent effect of raising HDL or lowering TG on the risk of coronary and cardiovascular morbidity and mortality has not been determined.

12.2 Pharmacodynamics

LIPITOR, as well as some of its metabolites, are pharmacologically active in humans. The liver is the primary site of action and the principal site of cholesterol synthesis and LDL clearance. Drug dosage, rather than systemic drug concentration, correlates better with LDL-C reduction. Individualization of drug dosage should be based on therapeutic response [see *Dosage and Administration (2)*].

12.3 Pharmacokinetics

Absorption: LIPITOR is rapidly absorbed after oral administration; maximum plasma concentrations occur within 1 to 2 hours. Extent of absorption increases in proportion to LIPITOR dose. The absolute bioavailability of atorvastatin (parent drug) is approximately 14% and the systemic availability of HMG-CoA reductase inhibitory activity is approximately 30%. The low systemic availability is attributed to presystemic clearance in gastrointestinal mucosa and/or hepatic first-pass metabolism. Although food decreases the rate and extent of drug absorption by approximately 25% and 9%, respectively, as assessed by Cmax and AUC, LDL-C reduction is similar whether LIPITOR is given with or without food. Plasma LIPITOR concentrations are lower (approximately 30% for Cmax and AUC) following evening drug administration compared with morning. However, LDL-C reduction is the same regardless of the time of day of drug administration [see *Dosage and Administration (2)*].

Distribution: Mean volume of distribution of LIPITOR is approximately 381 liters. LIPITOR is ≥98% bound to plasma proteins. A blood/plasma ratio of approximately 0.25 indicates poor drug penetration into red blood cells. Based on observations in rats, LIPITOR is likely to be secreted in human milk [see *Contraindications, Nursing Mothers (4.4)* and *Use in Specific Populations, Nursing Mothers (8.3)*].

Metabolism: LIPITOR is extensively metabolized to ortho- and parahydroxylated derivatives and various beta-oxidation products. *In vitro* inhibition of HMG-CoA reductase by ortho- and parahydroxylated metabolites is equivalent to that of LIPITOR. Approximately 70% of circulating inhibitory activity for HMG-CoA reductase is attributed to active metabolites. *In vitro* studies suggest the importance of LIPITOR metabolism by cytochrome P450 3A4, consistent with increased plasma concentrations of LIPITOR in humans following co-administration with erythromycin, a known inhibitor of this isozyme [see *Drug Interactions (7.1)*]. In animals, the ortho-hydroxy metabolite undergoes further glucuronidation.

Excretion: LIPITOR and its metabolites are eliminated primarily in bile following hepatic and/or extra-hepatic metabolism; however, the drug does not appear to undergo enterohepatic recirculation. Mean plasma elimination half-life of LIPITOR in humans is approximately 14 hours, but the half-life of inhibitory activity for HMG-CoA reductase is 20 to 30 hours due to the contribution of active metabolites. Less than 2% of a dose of LIPITOR is recovered in urine following oral administration.

Specific Populations

Geriatric: Plasma concentrations of LIPITOR are higher (approximately 40% for Cmax and 30% for AUC) in healthy elderly subjects (age ≥65 years) than in young adults. Clinical data suggest a greater degree of LDL-lowering at any dose of drug in the elderly patient population compared to younger adults [see *Use in Specific Populations, Geriatric Use (8.5)*].

Pediatric: Pharmacokinetic data in the pediatric population are not available.

Gender: Plasma concentrations of LIPITOR in women differ from those in men (approximately 20% higher for Cmax and 10% lower for AUC); however, there is no clinically significant difference in LDL-C reduction with LIPITOR between men and women.

Renal Impairment: Renal disease has no influence on the plasma concentrations or LDL-C reduction of LIPITOR; thus, dose adjustment in patients with renal dysfunction is not necessary [see *Dosage and Administration, Dosage in Patients with Renal Impairment (2.5), Warnings and Precautions, Skeletal Muscle (5.1)*].

Hemodialysis: While studies have not been conducted in patients with end-stage renal disease, hemodialysis is not expected to significantly enhance clearance of LIPITOR since the drug is extensively bound to plasma proteins.

Hepatic Impairment: In patients with chronic alcoholic liver disease, plasma concentrations of LIPITOR are markedly increased. Cmax and AUC are each 4-fold greater in patients with Childs-Pugh A disease. Cmax and AUC are approximately 16-fold and 11-fold increased, respectively, in patients with Childs-Pugh B disease [see *Contraindications (4.1)*].

TABLE 3. Effect of Co-administered Drugs on the Pharmacokinetics of Atorvastatin

**Co-administered drug and dosing regimen**	**Atorvastatin**		
	**Dose (mg)**	**Change in AUC***	**Change in Cmax***
†Cyclosporine 5.2 mg/kg/day, stable dose	10 mg QD for 28 days	↑ 8.7 fold	↑ 10.7 fold
†Tipranavir 500 mg BID/ritonavir 200 mg BID, 7 days	10 mg, SD	↑ 9.4 fold	↑ 8.6 fold
†Telaprevir 750 mg q8h, 10 days	20 mg, SD	↑ 7.88 fold	↑ 10.6 fold
†, ‡Saquinavir 400 mg BID/ ritonavir 400mg BID, 15 days	40 mg QD for 4 days	↑ 3.9 fold	↑ 4.3 fold
†Clarithromycin 500 mg BID, 9 days	80 mg QD for 8 days	↑ 4.4 fold	↑ 5.4 fold
†Darunavir 300 mg BID/ritonavir 100 mg BID, 9 days	10 mg QD for 4 days	↑ 3.4 fold	↑ 2.25 fold
†Itraconazole 200 mg QD, 4 days	40 mg SD	↑ 3.3 fold	↑ 20%
†Fosamprenavir 700 mg BID/ritonavir 100 mg BID, 14 days	10 mg QD for 4 days	↑ 2.53 fold	↑ 2.84 fold
†Fosamprenavir 1400 mg BID, 14 days	10 mg QD for 4 days	↑ 2.3 fold	↑ 4.04 fold
†Nelfinavir 1250 mg BID, 14 days	10 mg QD for 28 days	↑ 74%	↑ 2.2 fold
†Grapefruit Juice, 240 mL QD §	40 mg, SD	↑ 37%	↑ 16%
Diltiazem 240 mg QD, 28 days	40 mg, SD	↑ 51%	No change
Erythromycin 500 mg QID, 7 days	10 mg, SD	↑ 33%	↑ 38%
Amlodipine 10 mg, single dose	80 mg, SD	↑ 15%	↓ 12 %
Cimetidine 300 mg QID, 2 weeks	10 mg QD for 2 weeks	↓ Less than 1%	↓ 11%
Colestipol 10 mg BID, 28 weeks	40 mg QD for 28 weeks	Not determined	↓ 26%¶
Maalox TC® 30 mL QD, 17 days	10 mg QD for 15 days	↓ 33%	↓ 34%
Efavirenz 600 mg QD, 14 days	10 mg for 3 days	↓ 41%	↓ 1%
†Rifampin 600 mg QD, 7 days (co-administered) #	40 mg SD	↑ 30%	↑ 2.7 fold
†Rifampin 600 mg QD, 5 days (doses separated) #	40 mg SD	↓ 80%	↓ 40%

†Gemfibrozil 600mg BID, 7 days	40mg SD	↑ 35%	↓ Less than 1%
†Fenofibrate 160mg QD, 7 days	40mg SD	↑ 3%	↑ 2%
Boceprevir 800 mg TID, 7 days	40 mg SD	↑2.30 fold	↑2.66 fold

* Data given as x-fold change represent a simple ratio between co-administration and atorvastatin alone (i.e., 1-fold = no change). Data given as % change represent % difference relative to atorvastatin alone (i.e., 0% = no change).

† See Sections 5.1 and 7 for clinical significance.

‡ The dose of saquinavir plus ritonavir in this study is not the clinically used dose. The increase in atorvastatin exposure when used clinically is likely to be higher than what was observed in this study. Therefore, caution should be applied and the lowest dose necessary should be used.

§ Greater increases in AUC (up to 2.5 fold) and/or Cmax (up to 71%) have been reported with excessive grapefruit consumption (≥ 750 mL – 1.2 liters per day).

¶ Single sample taken 8–16 h post dose.

# Due to the dual interaction mechanism of rifampin, simultaneous co-administration of atorvastatin with rifampin is recommended, as delayed administration of atorvastatin after administration of rifampin has been associated with a significant reduction in atorvastatin plasma concentrations.

TABLE 4. Effect of Atorvastatin on the Pharmacokinetics of Co-administered Drugs

Atorvastatin	Co-administered drug and dosing regimen		
	**Drug/Dose (mg)**	**Change in AUC**	**Change in Cmax**
80 mg QD for 15 days	Antipyrine, 600 mg SD	↑ 3%	↓ 11%
80 mg QD for 14 days	* Digoxin 0.25 mg QD, 20 days	↑ 15%	↑ 20 %
40 mg QD for 22 days	Oral contraceptive QD, 2 months<br>- norethindrone 1mg<br>- ethinyl estradiol 35µg	 <br>↑ 28%<br>↑ 19%	 <br>↑ 23%<br>↑ 30%
10 mg, SD	Tipranavir 500 mg BID/ritonavir 200 mg BID, 7 days	No change	No change
10 mg QD for 4 days	Fosamprenavir 1400 mg BID, 14 days	↓ 27%	↓ 18%
10 mg QD for 4 days	Fosamprenavir 700 mg BID/ritonavir 100 mg BID, 14 days	No change	No change

* See Section 7 for clinical significance.

13 NONCLINICAL TOXICOLOGY

13.1 Carcinogenesis, Mutagenesis, Impairment of Fertility

In a 2-year carcinogenicity study in rats at dose levels of 10, 30, and 100 mg/kg/day, 2 rare tumors were found in muscle in high-dose females: in one, there was a rhabdomyosarcoma and, in another, there was a fibrosarcoma. This dose represents a plasma AUC (0–24) value of approximately 16 times the mean human plasma drug exposure after an 80 mg oral dose.

A 2-year carcinogenicity study in mice given 100, 200, or 400 mg/kg/day resulted in a significant increase in liver adenomas in high-dose males and liver carcinomas in high-dose females. These findings occurred at plasma AUC (0–24) values of approximately 6 times the mean human plasma drug exposure after an 80 mg oral dose.

In vitro, atorvastatin was not mutagenic or clastogenic in the following tests with and without metabolic

activation: the Ames test with *Salmonella typhimurium* and *Escherichia coli,* the HGPRT forward mutation assay in Chinese hamster lung cells, and the chromosomal aberration assay in Chinese hamster lung cells. Atorvastatin was negative in the *in vivo* mouse micronucleus test.

Studies in rats performed at doses up to 175 mg/kg (15 times the human exposure) produced no changes in fertility. There was aplasia and aspermia in the epididymis of 2 of 10 rats treated with 100 mg/kg/day of atorvastatin for 3 months (16 times the human AUC at the 80 mg dose); testis weights were significantly lower at 30 and 100 mg/kg and epididymal weight was lower at 100 mg/kg. Male rats given 100 mg/kg/day for 11 weeks prior to mating had decreased sperm motility, spermatid head concentration, and increased abnormal sperm. Atorvastatin caused no adverse effects on semen parameters, or reproductive organ histopathology in dogs given doses of 10, 40, or 120 mg/kg for two years.

14 CLINICAL STUDIES

14.1 Prevention of Cardiovascular Disease

In the Anglo-Scandinavian Cardiac Outcomes Trial (ASCOT), the effect of LIPITOR on fatal and non-fatal coronary heart disease was assessed in 10,305 hypertensive patients 40–80 years of age (mean of 63 years), without a previous myocardial infarction and with TC levels ≤251 mg/dL (6.5 mmol/L). Additionally, all patients had at least 3 of the following cardiovascular risk factors: male gender (81.1%), age >55 years (84.5%), smoking (33.2%), diabetes (24.3%), history of CHD in a first-degree relative (26%), TC:HDL >6 (14.3%), peripheral vascular disease (5.1%), left ventricular hypertrophy (14.4%), prior cerebrovascular event (9.8%), specific ECG abnormality (14.3%), proteinuria/albuminuria (62.4%). In this double-blind, placebo-controlled study, patients were treated with anti-hypertensive therapy (Goal BP <140/90 mm Hg for non-diabetic patients; <130/80 mm Hg for diabetic patients) and allocated to either LIPITOR 10 mg daily (n=5168) or placebo (n=5137), using a covariate adaptive method which took into account the distribution of nine baseline characteristics of patients already enrolled and minimized the imbalance of those characteristics across the groups. Patients were followed for a median duration of 3.3 years.

The effect of 10 mg/day of LIPITOR on lipid levels was similar to that seen in previous clinical trials.

LIPITOR significantly reduced the rate of coronary events [either fatal coronary heart disease (46 events in the placebo group vs. 40 events in the LIPITOR group) or non-fatal MI (108 events in the placebo group vs. 60 events in the LIPITOR group)] with a relative risk reduction of 36% [(based on incidences of 1.9% for LIPITOR vs. 3.0% for placebo), p=0.0005 (see Figure 1)]. The risk reduction was consistent regardless of age, smoking status, obesity, or presence of renal dysfunction. The effect of LIPITOR was seen regardless of baseline LDL levels. Due to the small number of events, results for women were inconclusive.

Figure 1: Effect of LIPITOR 10 mg/day on Cumulative Incidence of Non-Fatal Myocardial Infarction or Coronary Heart Disease Death (in ASCOT-LLA)

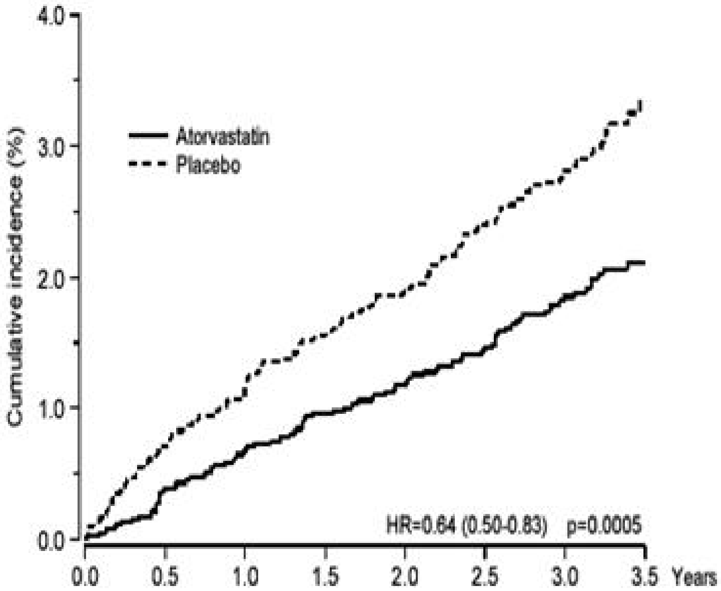

LIPITOR also significantly decreased the relative risk for revascularization procedures by 42%. Although the reduction of fatal and non-fatal strokes did not reach a pre-defined significance level (p=0.01), a favorable trend was observed with a 26% relative risk reduction (incidences of 1.7% for LIPITOR and 2.3% for placebo). There was no significant difference between the treatment groups for death due to cardiovascular causes (p=0.51) or noncardiovascular causes (p=0.17).

In the Collaborative Atorvastatin Diabetes Study (CARDS), the effect of LIPITOR on cardiovascular disease (CVD) endpoints was assessed in 2838 subjects (94% white, 68% male), ages 40–75 with type 2 diabetes based on WHO criteria, without prior history of cardiovascular disease and with LDL ≤ 160 mg/dL and TG ≤ 600 mg/dL. In addition to diabetes, subjects had 1 or more of the following risk factors: current smoking (23%), hypertension (80%), retinopathy (30%), or microalbuminuria (9%) or macroalbuminuria (3%). No subjects on hemodialysis were enrolled in the study. In this multicenter, placebo-controlled, double-blind clinical trial, subjects were randomly allocated to either LIPITOR 10 mg daily (1429) or placebo (1411) in a 1:1 ratio and were followed for a median duration of 3.9 years. The primary endpoint was the occurrence of any of the major cardiovascular events: myocardial infarction, acute CHD death, unstable angina, coronary revascularization, or stroke. The primary analysis was the time to first occurrence of the primary endpoint.

Baseline characteristics of subjects were: mean age of 62 years, mean HbA_{1c} 7.7%; median LDL-C 120 mg/dL; median TC 207 mg/dL; median TG 151 mg/dL; median HDL-C 52 mg/dL.

The effect of LIPITOR 10 mg/day on lipid levels was similar to that seen in previous clinical trials.

LIPITOR significantly reduced the rate of major cardiovascular events (primary endpoint events) (83 events in the LIPITOR group vs. 127 events in the placebo group) with a relative risk reduction of 37%, HR 0.63, 95% CI (0.48, 0.83) (p=0.001) (see Figure 2). An effect of LIPITOR was seen regardless of age, sex, or baseline lipid levels.

LIPITOR significantly reduced the risk of stroke by 48% (21 events in the LIPITOR group vs. 39 events in the placebo group), HR 0.52, 95% CI (0.31, 0.89) (p=0.016) and reduced the risk of MI by 42% (38 events in the LIPITOR group vs. 64 events in the placebo group), HR 0.58, 95.1% CI (0.39, 0.86) (p=0.007). There was no significant difference between the treatment groups for angina, revascularization procedures, and acute CHD death.

There were 61 deaths in the LIPITOR group vs. 82 deaths in the placebo group (HR 0.73, p=0.059).

Figure 2: Effect of LIPITOR 10 mg/day on Time to Occurrence of Major Cardiovascular Event (myocardial infarction, acute CHD death, unstable angina, coronary revascularization, or stroke)

in CARDS

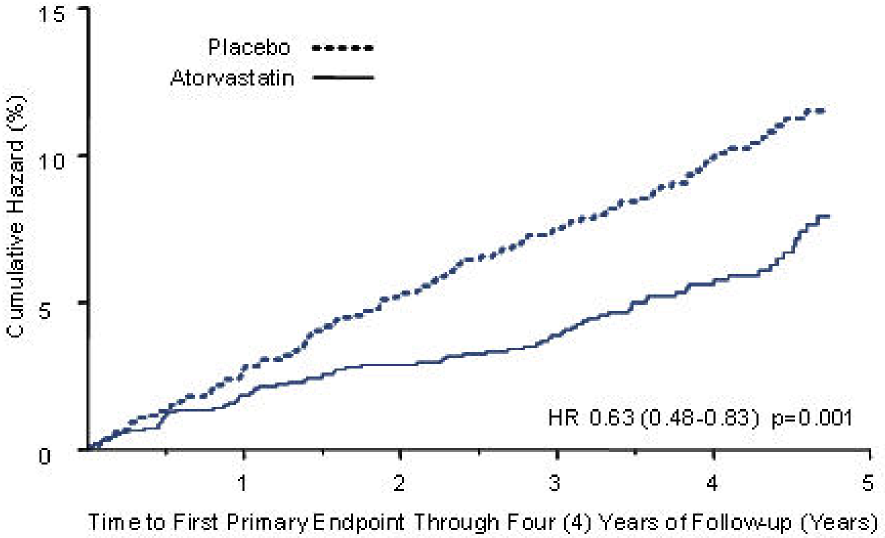

In the Treating to New Targets Study (TNT), the effect of LIPITOR 80 mg/day vs. LIPITOR 10 mg/day on the reduction in cardiovascular events was assessed in 10,001 subjects (94% white, 81% male, 38% ≥65 years) with clinically evident coronary heart disease who had achieved a target LDL-C level <130 mg/dL after completing an 8-week, open-label, run-in period with LIPITOR 10 mg/day. Subjects were randomly assigned to either 10 mg/day or 80 mg/day of LIPITOR and followed for a median duration of 4.9 years. The primary endpoint was the time-to-first occurrence of any of the following major cardiovascular events (MCVE): death due to CHD, non-fatal myocardial infarction, resuscitated cardiac arrest, and fatal and non-fatal stroke. The mean LDL-C, TC, TG, non-HDL, and HDL cholesterol levels at 12 weeks were 73, 145, 128, 98, and 47 mg/dL during treatment with 80 mg of LIPITOR and 99, 177, 152, 129, and 48 mg/dL during treatment with 10 mg of LIPITOR.

Treatment with LIPITOR 80 mg/day significantly reduced the rate of MCVE (434 events in the 80 mg/day group vs. 548 events in the 10 mg/day group) with a relative risk reduction of 22%, HR 0.78, 95% CI (0.69, 0.89), p=0.0002 (see Figure 3 and Table 5). The overall risk reduction was consistent regardless of age (<65, ≥65) or gender.

Figure 3: Effect of LIPITOR 80 mg/day vs. 10 mg/day on Time to Occurrence of Major Cardiovascular Events (TNT)

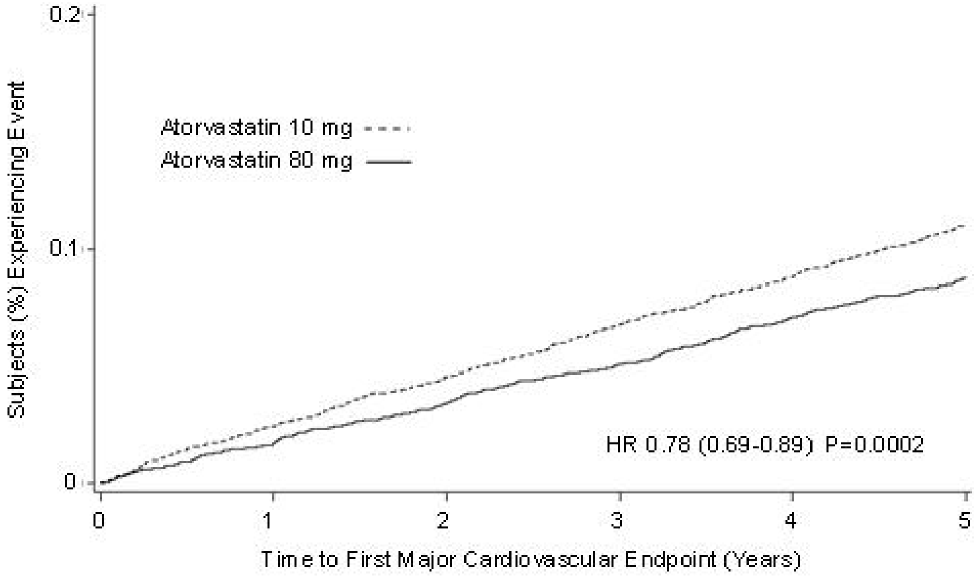

TABLE 5. Overview of Efficacy Results in TNT

Endpoint	Atorvastatin 10 mg (N=5006)		Atorvastatin 80 mg (N=4995)		HR* (95%CI)
**PRIMARY ENDPOINT**	n	(%)	n	(%)	
First major cardiovascular endpoint	548	(10.9)	434	(8.7)	0.78 (0.69, 0.89)
**Components of the Primary Endpoint**					
CHD death	127	(2.5)	101	(2.0)	0.80 (0.61, 1.03)
Non-fatal, non-procedure related MI	308	(6.2)	243	(4.9)	0.78 (0.66, 0.93)
Resuscitated cardiac arrest	26	(0.5)	25	(0.5)	0.96 (0.56, 1.67)
Stroke (fatal and non-fatal)	155	(3.1)	117	(2.3)	0.75 (0.59, 0.96)
**SECONDARY ENDPOINTS†**					
First CHF with hospitalization	164	(3.3)	122	(2.4)	0.74 (0.59, 0.94)
First PVD endpoint	282	(5.6)	275	(5.5)	0.97 (0.83, 1.15)
First CABG or other coronary revascularization procedure‡	904	(18.1)	667	(13.4)	0.72 (0.65, 0.80)
First documented angina endpoint‡	615	(12.3)	545	(10.9)	0.88 (0.79, 0.99)
All-cause mortality	282	(5.6)	284	(5.7)	1.01 (0.85, 1.19)
**Components of All-Cause Mortality**					
Cardiovascular death	155	(3.1)	126	(2.5)	0.81 (0.64, 1.03)
Noncardiovascular death	127	(2.5)	158	(3.2)	1.25 (0.99, 1.57)
Cancer death	75	(1.5)	85	(1.7)	1.13 (0.83, 1.55)
Other non-CV death	43	(0.9)	58	(1.2)	1.35 (0.91, 2.00)
Suicide, homicide, and other traumatic non-CV death	9	(0.2)	15	(0.3)	1.67 (0.73, 3.82)

HR=hazard ratio; CHD=coronary heart disease; CI=confidence interval; MI=myocardial infarction; CHF=congestive heart failure; CV=cardiovascular; PVD=peripheral vascular disease; CABG=coronary artery bypass graft

Confidence intervals for the Secondary Endpoints were not adjusted for multiple comparisons

* Atorvastatin 80 mg: atorvastatin 10 mg

† Secondary endpoints not included in primary endpoint

‡ Component of other secondary endpoints

Of the events that comprised the primary efficacy endpoint, treatment with LIPITOR 80 mg/day significantly reduced the rate of non-fatal, non-procedure related MI and fatal and non-fatal stroke, but not CHD death or resuscitated cardiac arrest (Table 5). Of the predefined secondary endpoints, treatment with LIPITOR 80 mg/day significantly reduced the rate of coronary revascularization, angina, and hospitalization for heart failure, but not peripheral vascular disease. The reduction in the rate of CHF with hospitalization was only observed in the 8% of patients with a prior history of CHF.

There was no significant difference between the treatment groups for all-cause mortality (Table 5). The proportions of subjects who experienced cardiovascular death, including the components of CHD death and fatal stroke, were numerically smaller in the LIPITOR 80 mg group than in the LIPITOR 10 mg treatment group. The proportions of subjects who experienced noncardiovascular death were numerically larger in the LIPITOR 80 mg group than in the LIPITOR 10 mg treatment group.

In the Incremental Decrease in Endpoints Through Aggressive Lipid Lowering Study (IDEAL), treatment with LIPITOR 80 mg/day was compared to treatment with simvastatin 20–40 mg/day in 8,888 subjects up to 80 years of age with a history of CHD to assess whether reduction in CV risk could be achieved. Patients were mainly male (81%), white (99%) with an average age of 61.7 years, and an

average LDL-C of 121.5 mg/dL at randomization; 76% were on statin therapy. In this prospective, randomized, open-label, blinded endpoint (PROBE) trial with no run-in period, subjects were followed for a median duration of 4.8 years. The mean LDL-C, TC, TG, HDL, and non-HDL cholesterol levels at Week 12 were 78, 145, 115, 45, and 100 mg/dL during treatment with 80 mg of LIPITOR and 105, 179, 142, 47, and 132 mg/dL during treatment with 20–40 mg of simvastatin.

There was no significant difference between the treatment groups for the primary endpoint, the rate of first major coronary event (fatal CHD, non-fatal MI, and resuscitated cardiac arrest): 411 (9.3%) in the LIPITOR 80 mg/day group vs. 463 (10.4%) in the simvastatin 20–40 mg/day group, HR 0.89, 95% CI (0.78, 1.01), p=0.07.

There were no significant differences between the treatment groups for all-cause mortality: 366 (8.2%) in the LIPITOR 80 mg/day group vs. 374 (8.4%) in the simvastatin 20–40 mg/day group. The proportions of subjects who experienced CV or non-CV death were similar for the LIPITOR 80 mg group and the simvastatin 20–40 mg group.

14.2 Hyperlipidemia (Heterozygous Familial and Nonfamilial) and Mixed Dyslipidemia *(Fredrickson* Types IIa and IIb)

LIPITOR reduces total-C, LDL-C, VLDL-C, apo B, and TG, and increases HDL-C in patients with hyperlipidemia and mixed dyslipidemia. Therapeutic response is seen within 2 weeks, and maximum response is usually achieved within 4 weeks and maintained during chronic therapy.

LIPITOR is effective in a wide variety of patient populations with hyperlipidemia, with and without hypertriglyceridemia, in men and women, and in the elderly.

In two multicenter, placebo-controlled, dose-response studies in patients with hyperlipidemia, LIPITOR given as a single dose over 6 weeks, significantly reduced total-C, LDL-C, apo B, and TG. (Pooled results are provided in Table 6.)

TABLE 6. Dose Response in Patients With Primary Hyperlipidemia (Adjusted Mean % Change From Baseline)*

Dose	N	TC	LDL-C	Apo B	TG	HDL-C	Non-HDL-C/HDL-C
Placebo	21	4	4	3	10	-3	7
10	22	-29	-39	-32	-19	6	-34
20	20	-33	-43	-35	-26	9	-41
40	21	-37	-50	-42	-29	6	-45
80	23	-45	-60	-50	-37	5	-53

* Results are pooled from 2 dose-response studies.

In patients with *Fredrickson* Types IIa and IIb hyperlipoproteinemia pooled from 24 controlled trials, the median (25th and 75th percentile) percent changes from baseline in HDL-C for LIPITOR 10, 20, 40, and 80 mg were 6.4 (-1.4, 14), 8.7 (0, 17), 7.8 (0, 16), and 5.1 (-2.7, 15), respectively. Additionally, analysis of the pooled data demonstrated consistent and significant decreases in total-C, LDL-C, TG, total-C/HDL-C, and LDL-C/HDL-C.

In three multicenter, double-blind studies in patients with hyperlipidemia, LIPITOR was compared to other statins. After randomization, patients were treated for 16 weeks with either LIPITOR 10 mg per day or a fixed dose of the comparative agent (Table 7).

TABLE 7. Mean Percentage Change From Baseline at Endpoint (Double-Blind, Randomized, Active-Controlled Trials)

Treatment (Daily Dose)	N	Total-C	LDL-C	Apo B	TG	HDL-C	Non-HDL-C/HDL-C
Study 1							
LIPITOR 10 mg	707	-27*	-36*	-28*	-17*	+7	-37*
Lovastatin 20 mg	191	-19	-27	-20	-6	+7	-28
95% CI for Diff†		-9.2, -6.5	-10.7, -7.1	-10.0, -6.5	-15.2, -7.1	-1.7, 2.0	-11.1, -7.1
Study 2							
LIPITOR 10 mg	222	-25‡	-35‡	-27‡	-17‡	+6	-36‡
Pravastatin 20 mg	77	-17	-23	-17	-9	+8	-28
95% CI for Diff†		-10.8, -6.1	-14.5, -8.2	-13.4, -7.4	-14.1, -0.7	-4.9, 1.6	-11.5, -4.1
Study 3							
LIPITOR 10 mg	132	-29§	-37§	-34§	-23§	+7	-39§
Simvastatin 10 mg	45	-24	-30	-30	-15	+7	-33
95% CI for Diff†		-8.7, -2.7	-10.1, -2.6	-8.0, -1.1	-15.1, -0.7	-4.3, 3.9	-9.6, -1.9

* Significantly different from lovastatin, ANCOVA, p ≤0.05

† A negative value for the 95% CI for the difference between treatments favors LIPITOR for all except HDL-C, for which a positive value favors LIPITOR. If the range does not include 0, this indicates a statistically significant difference.

‡ Significantly different from pravastatin, ANCOVA, p ≤0.05

§ Significantly different from simvastatin, ANCOVA, p ≤0.05

The impact on clinical outcomes of the differences in lipid-altering effects between treatments shown in Table 7 is not known. Table 7 does not contain data comparing the effects of LIPITOR 10 mg and higher doses of lovastatin, pravastatin, and simvastatin. The drugs compared in the studies summarized in the table are not necessarily interchangeable.

14.3 Hypertriglyceridemia *(Fredrickson* Type IV)

The response to LIPITOR in 64 patients with isolated hypertriglyceridemia treated across several clinical trials is shown in the table below (Table 8). For the LIPITOR-treated patients, median (min, max) baseline TG level was 565 (267–1502).

TABLE 8. Combined Patients With Isolated Elevated TG: Median (min, max) Percentage Change From Baseline

	Placebo (N=12)	LIPITOR 10 mg (N=37)	LIPITOR 20 mg (N=13)	LIPITOR 80 mg (N=14)
Triglycerides	-12.4 (-36.6, 82.7)	-41.0 (-76.2, 49.4)	-38.7 (-62.7, 29.5)	-51.8 (-82.8, 41.3)
Total-C	-2.3 (-15.5, 24.4)	-28.2 (-44.9, -6.8)	-34.9 (-49.6, -15.2)	-44.4 (-63.5, -3.8)
LDL-C	3.6 (-31.3, 31.6)	-26.5 (-57.7, 9.8)	-30.4 (-53.9, 0.3)	-40.5 (-60.6, -13.8)
HDL-C	3.8 (-18.6, 13.4)	13.8 (-9.7, 61.5)	11.0 (-3.2, 25.2)	7.5 (-10.8, 37.2)
VLDL-C	-1.0 (-31.9, 53.2)	-48.8 (-85.8, 57.3)	-44.6 (-62.2, -10.8)	-62.0 (-88.2, 37.6)
non-HDL-C	-2.8 (-17.6, 30.0)	-33.0 (-52.1, -13.3)	-42.7 (-53.7, -17.4)	-51.5 (-72.9, -4.3)

14.4 Dysbetalipoproteinemia *(Fredrickson* Type III)

The results of an open-label crossover study of 16 patients (genotypes: 14 apo E2/E2 and 2 apo E3/E2) with dysbetalipoproteinemia *(Fredrickson* Type III) are shown in the table below (Table 9).

TABLE 9. Open-Label Crossover Study of 16 Patients With Dysbetalipoproteinemia (Fredrickson Type III)

	Median (min, max) at Baseline (mg/dL)	Median % Change (min, max)	
		LIPITOR 10 mg	LIPITOR 80 mg
Total-C	442 (225, 1320)	-37 (-85, 17)	-58 (-90, -31)
Triglycerides	678 (273, 5990)	-39 (-92, -8)	-53 (-95, -30)
IDL-C + VLDL-C	215 (111, 613)	-32 (-76, 9)	-63 (-90, -8)
non-HDL-C	411 (218, 1272)	-43 (-87, -19)	-64 (-92, -36)

14.5 Homozygous Familial Hypercholesterolemia

In a study without a concurrent control group, 29 patients ages 6 to 37 years with homozygous FH received maximum daily doses of 20 to 80 mg of LIPITOR. The mean LDL-C reduction in this study was 18%. Twenty-five patients with a reduction in LDL-C had a mean response of 20% (range of 7% to 53%, median of 24%); the remaining 4 patients had 7% to 24% increases in LDL-C. Five of the 29 patients had absent LDL-receptor function. Of these, 2 patients also had a portacaval shunt and had no significant reduction in LDL-C. The remaining 3 receptor-negative patients had a mean LDL-C reduction of 22%.

14.6 Heterozygous Familial Hypercholesterolemia in Pediatric Patients

In a double-blind, placebo-controlled study followed by an open-label phase, 187 boys and postmenarchal girls 10–17 years of age (mean age 14.1 years) with heterozygous familial hypercholesterolemia (FH) or severe hypercholesterolemia, were randomized to LIPITOR (n=140) or placebo (n=47) for 26 weeks and then all received LIPITOR for 26 weeks. Inclusion in the study required 1) a baseline LDL-C level ≥ 190 mg/dL or 2) a baseline LDL-C level ≥ 160 mg/dL and positive family history of FH or documented premature cardiovascular disease in a first or second-degree relative. The mean baseline LDL-C value was 218.6 mg/dL (range: 138.5–385.0 mg/dL) in the LIPITOR group compared to 230.0 mg/dL (range: 160.0–324.5 mg/dL) in the placebo group. The dosage of LIPITOR (once daily) was 10 mg for the first 4 weeks and uptitrated to 20 mg if the LDL-C level was > 130 mg/dL. The number of LIPITOR-treated patients who required uptitration to 20 mg after Week 4 during the double-blind phase was 78 (55.7%).

LIPITOR significantly decreased plasma levels of total-C, LDL-C, triglycerides, and apolipoprotein B during the 26-week double-blind phase (see Table 10).

TABLE 10. Lipid-altering Effects of LIPITOR in Adolescent Boys and Girls with Heterozygous Familial Hypercholesterolemia or Severe Hypercholesterolemia (Mean Percentage Change From Baseline at Endpoint in Intention-to-Treat Population)

DOSAGE	N	Total-C	LDL-C	HDL-C	TG	Apolipoprotein B
Placebo	47	-1.5	-0.4	-1.9	1.0	0.7
LIPITOR	140	-31.4	-39.6	2.8	-12.0	-34.0

The mean achieved LDL-C value was 130.7 mg/dL (range: 70.0–242.0 mg/dL) in the LIPITOR group compared to 228.5 mg/dL (range: 152.0–385.0 mg/dL) in the placebo group during the 26-week double-blind phase.

The safety and efficacy of doses above 20 mg have not been studied in controlled trials in children. The

long-term efficacy of LIPITOR therapy in childhood to reduce morbidity and mortality in adulthood has not been established.

15 REFERENCES

[1] National Cholesterol Education Program (NCEP): Highlights of the Report of the Expert Panel on Blood Cholesterol Levels in Children and Adolescents, *Pediatrics*. 89(3):495–501. 1992.

16 HOW SUPPLIED/STORAGE AND HANDLING

10 mg tablets: coded "PD 155" on one side and "10" on the other.

NDC 0071-0155-23 bottles of 90
NDC 0071-0155-34 bottles of 5000
NDC 0071-0155-40 10 × 10 unit dose blisters
NDC 0071-0155-10 bottles of 1000

20 mg tablets: coded "PD 156" on one side and "20" on the other.

NDC 0071-0156-23 bottles of 90
NDC 0071-0156-40 10 × 10 unit dose blisters
NDC 0071-0156-94 bottles of 5000
NDC 0071-0156-10 bottles of 1000

40 mg tablets: coded "PD 157" on one side and "40" on the other.

NDC 0071-0157-23 bottles of 90
NDC 0071-0157-73 bottles of 500
NDC 0071-0157-88 bottles of 2500
NDC 0071-0157-40 10 × 10 unit dose blisters

80 mg tablets: coded "PD 158" on one side and "80" on the other.

NDC 0071-0158-23 bottles of 90
NDC 0071-0158-73 bottles of 500
NDC 0071-0158-88 bottles of 2500
NDC 0071-0158-92 8 × 8 unit dose blisters

Storage

Store at controlled room temperature 20 – 25°C (68 – 77°F) [see USP].

17 PATIENT COUNSELING INFORMATION

Patients taking LIPITOR should be advised that cholesterol is a chronic condition and they should adhere to their medication along with their National Cholesterol Education Program (NCEP)-recommended diet, a regular exercise program as appropriate, and periodic testing of a fasting lipid panel to determine goal attainment.

Patients should be advised about substances they should not take concomitantly with atorvastatin [see *Warnings and Precautions (5.1)*]. Patients should also be advised to inform other healthcare professionals prescribing a new medication that they are taking LIPITOR.

17.1 Muscle Pain

All patients starting therapy with LIPITOR should be advised of the risk of myopathy and told to report promptly any unexplained muscle pain, tenderness, or weakness particularly if accompanied by malaise or fever or if these muscle signs or symptoms persist after discontinuing LIPITOR. The risk of this

occurring is increased when taking certain types of medication or consuming larger quantities (>1 liter) of grapefruit juice. They should discuss all medication, both prescription and over the counter, with their healthcare professional.

17.2 Liver Enzymes

It is recommended that liver enzyme tests be performed before the initiation of LIPITOR and if signs or symptoms of liver injury occur. All patients treated with LIPITOR should be advised to report promptly any symptoms that may indicate liver injury, including fatigue, anorexia, right upper abdominal discomfort, dark urine, or jaundice.

17.3 Pregnancy

Women of childbearing age should be advised to use an effective method of birth control to prevent pregnancy while using LIPITOR. Discuss future pregnancy plans with your patients, and discuss when to stop LIPITOR if they are trying to conceive. Patients should be advised that if they become pregnant, they should stop taking LIPITOR and call their healthcare professional.

17.4 Breast-feeding

Women who are breastfeeding should be advised to not use LIPITOR. Patients who have a lipid disorder and are breast-feeding, should be advised to discuss the options with their healthcare professional.

Distributed by
Pfizer Parke-Davis
Division of Pfizer Inc, NY, NY 10017

LAB-0021-31.0

PATIENT INFORMATION

LIPITOR®
atorvastatin calcium
tablets

(LIP-ih-tore)

Read the Patient Information that comes with LIPITOR before you start taking it and each time you get a refill. There may be new information. This leaflet does not take the place of talking with your doctor about your condition or treatment.

If you have any questions about LIPITOR, ask your doctor or pharmacist.

What is LIPITOR?

LIPITOR is a prescription medicine that lowers cholesterol in your blood. It lowers the LDL-C ("bad" cholesterol) and triglycerides in your blood. It can raise your HDL-C ("good" cholesterol) as well. LIPITOR is for adults and children over 10 whose cholesterol does not come down enough with exercise and a low-fat diet alone.

LIPITOR can lower the risk for heart attack, stroke, certain types of heart surgery, and chest pain in patients who have heart disease or risk factors for heart disease such as:

- age, smoking, high blood pressure, low HDL-C, heart disease in the family.

LIPITOR can lower the risk for heart attack or stroke in patients with diabetes and risk factors such as:

- eye problems, kidney problems, smoking, or high blood pressure.

LIPITOR starts to work in about 2 weeks.

What is Cholesterol?

Cholesterol and triglycerides are fats that are made in your body. They are also found in foods. You need some cholesterol for good health, but too much is not good for you. Cholesterol and triglycerides can clog your blood vessels. It is especially important to lower your cholesterol if you have heart disease, smoke, have diabetes or high blood pressure, are older, or if heart disease starts early in your family.

Who Should Not Take LIPITOR?

Do not take LIPITOR if you:

- are pregnant or think you may be pregnant, or are planning to become pregnant. Lipitor may harm your unborn baby. If you get pregnant, stop taking LIPITOR and call your doctor right away.
- are breast feeding**.** LIPITOR can pass into your breast milk and may harm your baby.
- have liver problems.
- are allergic to LIPITOR or any of its ingredients. The active ingredient is atorvastatin. See the end of this leaflet for a complete list of ingredients in LIPITOR.

LIPITOR has not been studied in children under 10 years of age.

Before You Start LIPITOR

Tell your doctor if you:

- have muscle aches or weakness
- drink more than 2 glasses of alcohol daily
- have diabetes
- have a thyroid problem
- have kidney problems

Some medicines should not be taken with LIPITOR. Tell your doctor about all the medicines you take, including prescription and non-prescription medicines, vitamins, and herbal supplements. LIPITOR and certain other medicines can interact causing serious side effects. Especially tell your doctor if you take medicines for:

- your immune system
- cholesterol
- infections
- birth control
- heart failure
- HIV or AIDS

Know all the medicines you take. Keep a list of them with you to show your doctor and pharmacist.

How Should I Take LIPITOR?

- Take LIPITOR exactly as prescribed by your doctor. Do not change your dose or stop LIPITOR without talking to your doctor. Your doctor may do blood tests to check your cholesterol levels during your treatment with LIPITOR. Your dose of LIPITOR may be changed based on these blood test results.
- Take LIPITOR each day at any time of day at about the same time each day. LIPITOR can be taken with or without food.
  Don't break LIPITOR tablets before taking.
- Your doctor should start you on a low-fat diet before giving you LIPITOR. Stay on this low-fat diet when you take LIPITOR.
- If you miss a dose of LIPITOR, take it as soon as you remember. Do not take LIPITOR if it has been more than 12 hours since you missed your last dose. Wait and take the next dose at your regular time.

Do not take 2 doses of LIPITOR at the same time.

- If you take too much LIPITOR or overdose, call your doctor or Poison Control Center right away. Or go to the nearest emergency room.

What Should I Avoid While Taking LIPITOR?

- Talk to your doctor before you start any new medicines. This includes prescription and non-prescription medicines, vitamins, and herbal supplements. LIPITOR and certain other medicines can interact causing serious side effects.
- Do not get pregnant. If you get pregnant, stop taking LIPITOR right away and call your doctor.

What are the Possible Side Effects of LIPITOR?

LIPITOR can cause serious side effects. These side effects have happened only to a small number of people. Your doctor can monitor you for them. These side effects usually go away if your dose is lowered or LIPITOR is stopped. These serious side effects include:

- **Muscle problems.** LIPITOR can cause serious muscle problems that can lead to kidney problems, including kidney failure. You have a higher chance for muscle problems if you are taking certain other medicines with LIPITOR.
- **Liver problems.** Your doctor should do blood tests to check your liver before you start taking LIPITOR and if you have symptoms of liver problems while you take LIPITOR. Call your doctor right away if you have the following symptoms of liver problems:
  - feel tired or weak
  - loss of appetite
  - upper belly pain
  - dark amber colored urine
  - yellowing of your skin or the whites of your eyes

Call your doctor right away if you have:

- muscle problems like weakness, tenderness, or pain that happen without a good reason, especially if you also have a fever or feel more tired than usual. This may be an early sign of a rare muscle problem.
- muscle problems that do not go away even after your doctor has advised you to stop taking LIPITOR. Your doctor may do further tests to diagnose the cause of your muscle problems.
- allergic reactions including swelling of the face, lips, tongue, and/or throat that may cause difficulty in breathing or swallowing which may require treatment right away.
- nausea and vomiting.
- passing brown or dark-colored urine.
- you feel more tired than usual
- your skin and whites of your eyes get yellow.
- stomach pain.
- allergic skin reactions.

In clinical studies, patients reported the following common side effects while taking LIPITOR: diarrhea, upset stomach, muscle and joint pain, and alterations in some laboratory blood tests.

The following additional side effects have been reported with LIPITOR:

tiredness, tendon problems, memory loss, and confusion.

Talk to your doctor or pharmacist if you have side effects that bother you or that will not go away.

These are not all the side effects of LIPITOR. Ask your doctor or pharmacist for a complete list.

How do I store LIPITOR

- Store LIPITOR at room temperature, 68 to 77°F (20 to 25°C).

- Do not keep medicine that is out of date or that you no longer need.
- **Keep LIPITOR and all medicines out of the reach of children.** Be sure that if you throw medicine away, it is out of the reach of children.

General Information About LIPITOR

Medicines are sometimes prescribed for conditions that are not mentioned in patient information leaflets. Do not use LIPITOR for a condition for which it was not prescribed. Do not give LIPITOR to other people, even if they have the same problem you have. It may harm them.

This leaflet summarizes the most important information about LIPITOR. If you would like more information, talk with your doctor. You can ask your doctor or pharmacist for information about LIPITOR that is written for health professionals. Or you can go to the LIPITOR website at www.lipitor.com.

What are the Ingredients in LIPITOR?

Active Ingredient: atorvastatin calcium

Inactive Ingredients: calcium carbonate, USP; candelilla wax, FCC; croscarmellose sodium, NF; hydroxypropyl cellulose, NF; lactose monohydrate, NF; magnesium stearate, NF; microcrystalline cellulose, NF; Opadry White YS-1-7040 (hypromellose, polyethylene glycol, talc, titanium dioxide); polysorbate 80, NF; simethicone emulsion.

Pfizer

Distributed by
Parke-Davis
Division of Pfizer Inc, NY, NY 10017

LAB-0348-8.0
October 2012

PRINCIPAL DISPLAY PANEL - 10 mg Tablet Bottle Label

NDC 0071-0155-23

Pfizer

Lipitor®
(atorvastatin calcium)
tablets

10 mg*

90 Tablets
Rx only

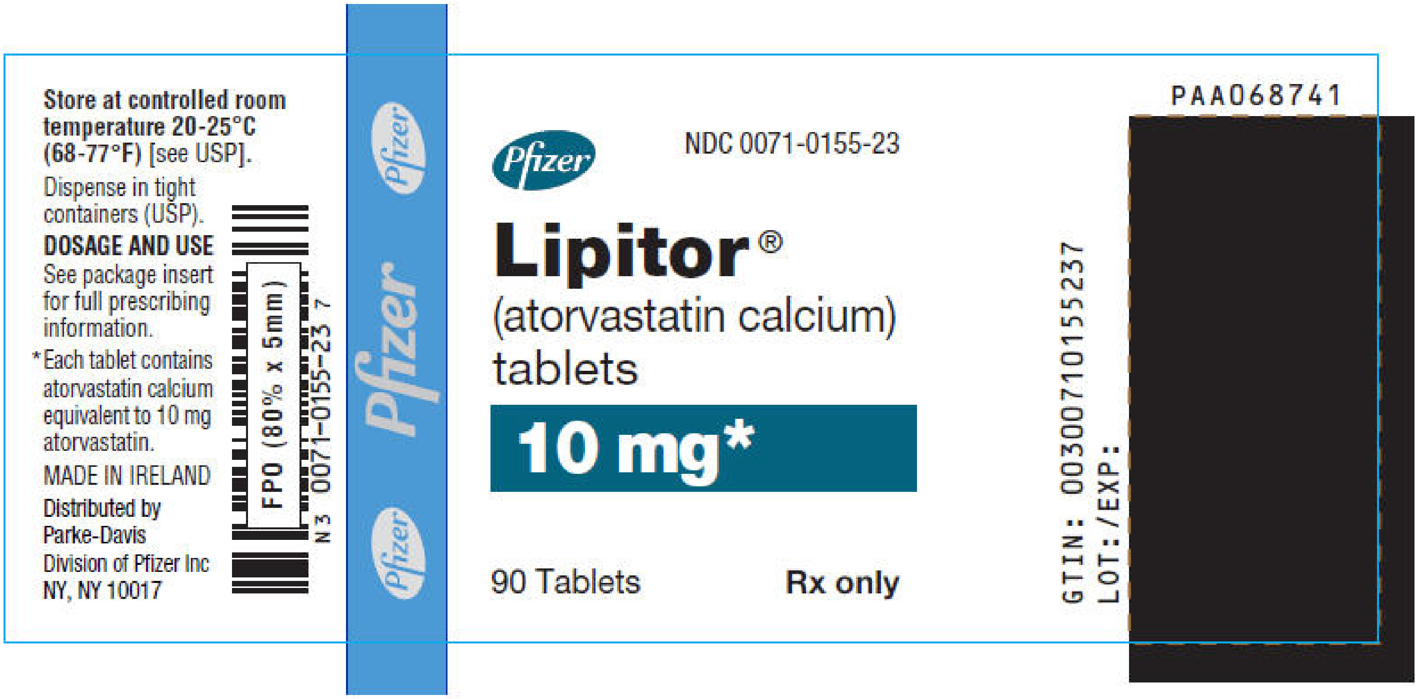

PRINCIPAL DISPLAY PANEL - 10 mg Tablet Blister Pack

Lipitor®

(Atorvastatin Calcium)
Tablet

10 mg

DISTRIBUTED BY:
PARKE-DAVIS
DIV OF PFIZER INC, NY, NY 10017
MADE IN IRELAND

EXP & LOT AREA

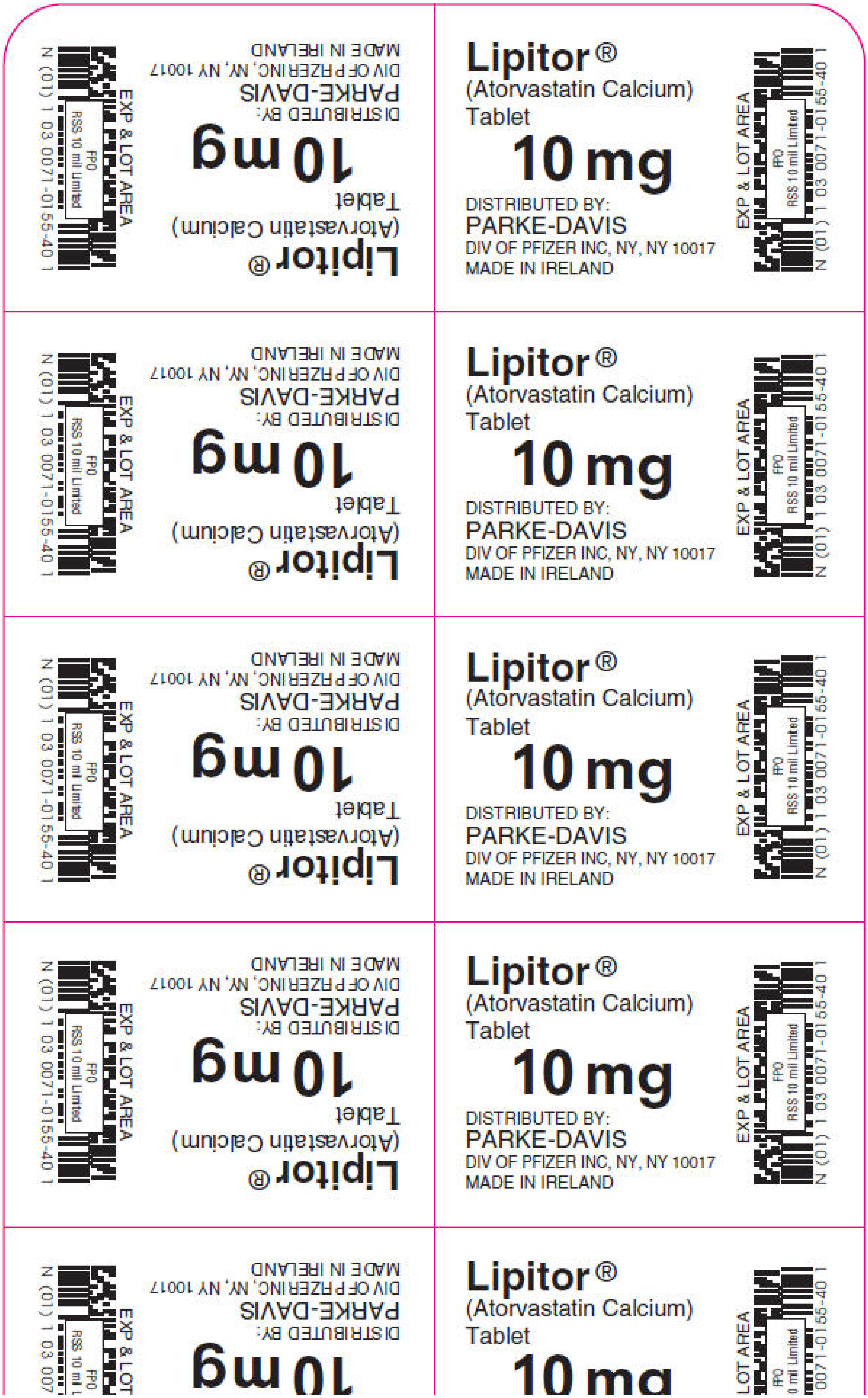
Lipitor®
(Atorvastatin Calcium)
Tablet
10 mg
DISTRIBUTED BY:
PARKE-DAVIS
DIV OF PFIZER INC, NY, NY 10017
MADE IN IRELAND
EXP & LOT AREA
FPO
RSS 10 mil Limited
N (01) 1 03 0071-0155-40 1
Lipitor®
(Atorvastatin Calcium)
Tablet
10 mg
DISTRIBUTED BY:
PARKE-DAVIS
DIV OF PFIZER INC, NY, NY 10017
MADE IN IRELAND
EXP & LOT AREA
FPO
RSS 10 mil Limited
N (01) 1 03 0071-0155-40 1
Lipitor®
(Atorvastatin Calcium)
Tablet
10 mg
DISTRIBUTED BY:
PARKE-DAVIS
DIV OF PFIZER INC, NY, NY 10017
MADE IN IRELAND
EXP & LOT AREA
FPO
RSS 10 mil Limited
N (01) 1 03 0071-0155-40 1
Lipitor®
(Atorvastatin Calcium)
Tablet
10 mg
DISTRIBUTED BY:
PARKE-DAVIS
DIV OF PFIZER INC, NY, NY 10017
MADE IN IRELAND
EXP & LOT AREA
FPO
RSS 10 mil Limited
N (01) 1 03 0071-0155-40 1
Lipitor®
(Atorvastatin Calcium)
Tablet
10 mg

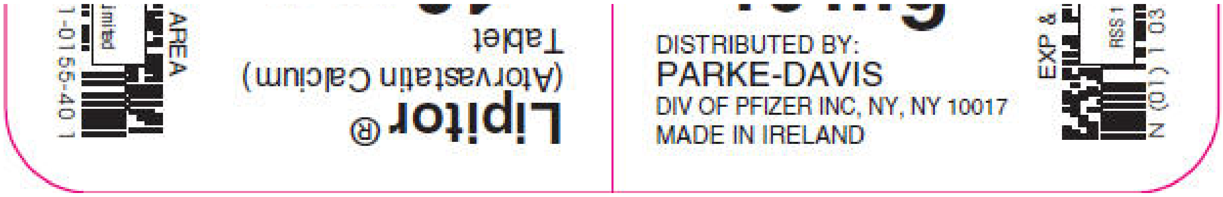

PRINCIPAL DISPLAY PANEL - 10 mg Blister Pack Carton

Pfizer

Lipitor®
(atorvastatin calcium)

10 mg*

tablets

For in-institution use only

100 Tablets

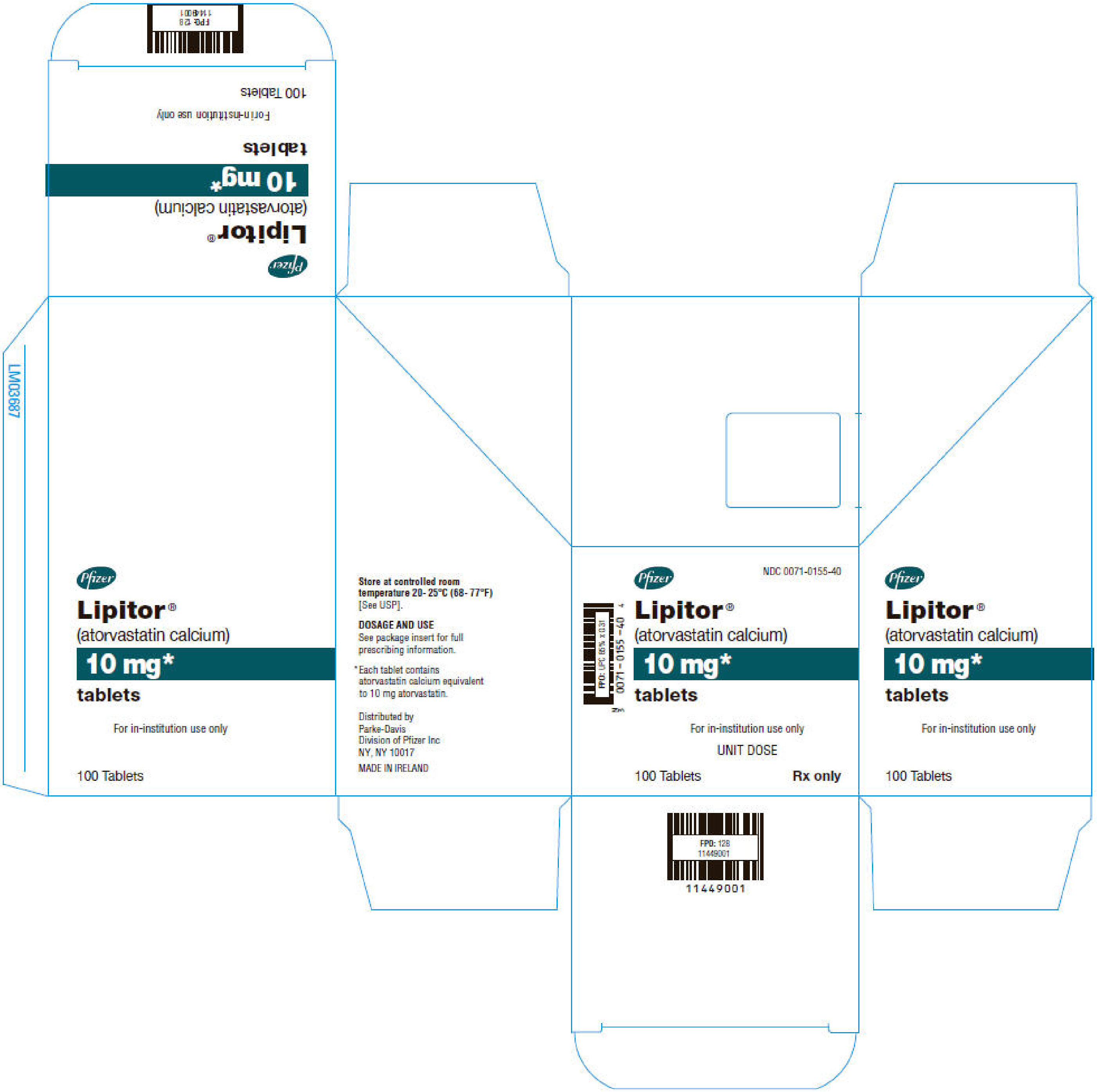

PRINCIPAL DISPLAY PANEL - 10 mg Tablet Packet Carton

NDC 0071-0155-97
PROFESSIONAL SAMPLE – NOT FOR SALE

LIPITOR®
atorvastatin calcium
tablets

10 mg*
7 TABLETS

PACKAGE NOT CHILD RESISTANT.
KEEP OUT OF REACH OF CHILDREN.

Rx only

Patient information enclosed

7 Tablets x 10 mg

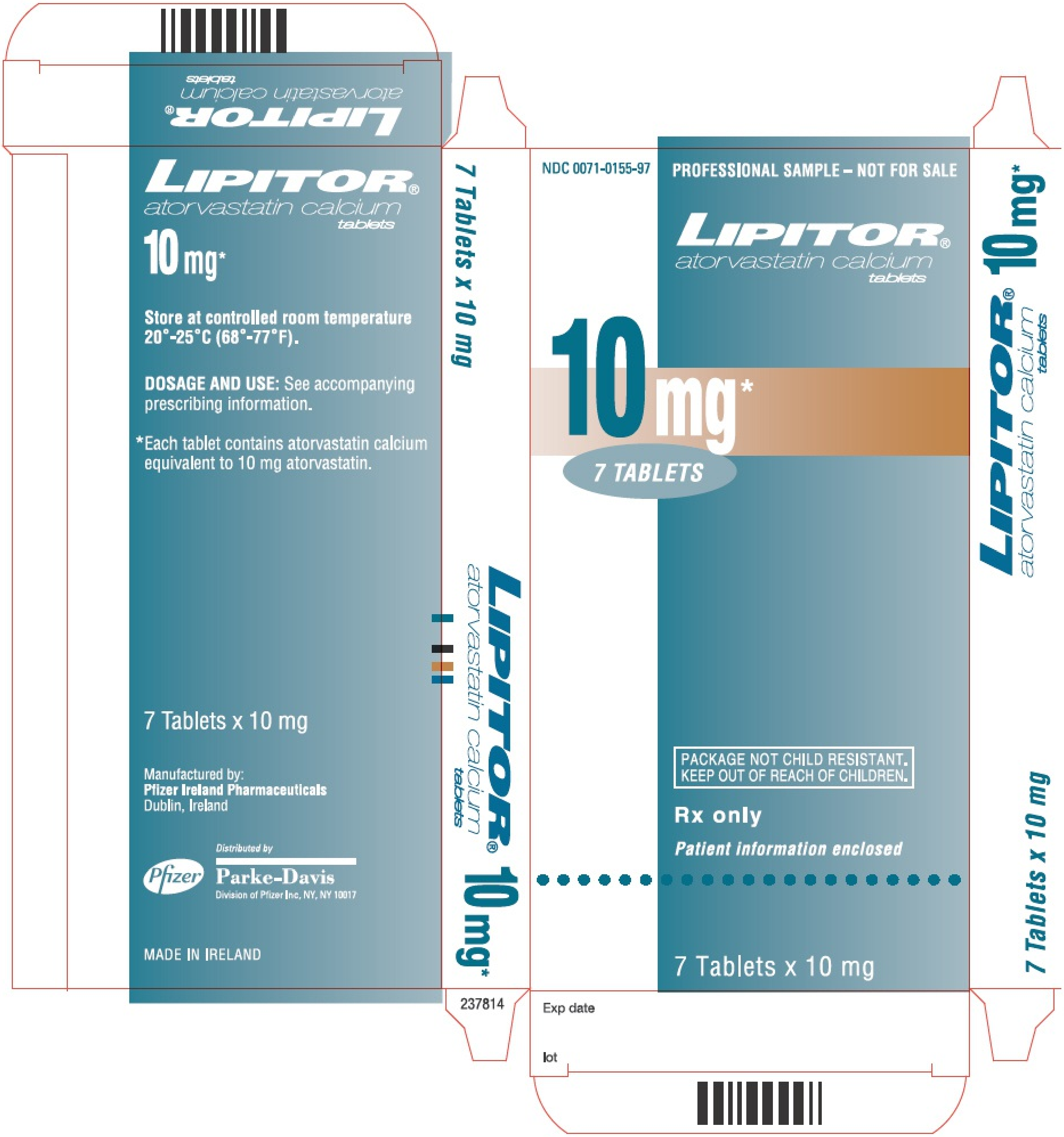
LIPITOR®
atorvastatin calcium
tablets
10 mg*
Store at controlled room temperature 20°-25°C (68°-77°F).
DOSAGE AND USE: See accompanying prescribing information.
*Each tablet contains atorvastatin calcium equivalent to 10 mg atorvastatin.
7 Tablets x 10 mg
Manufactured by:
Pfizer Ireland Pharmaceuticals
Dublin, Ireland
Distributed by
Pfizer
Parke-Davis
Division of Pfizer Inc, NY, NY 10017
MADE IN IRELAND
7 Tablets x 10 mg
LIPITOR®
atorvastatin calcium
tablets
10 mg*
237814
NDC 0071-0155-97
PROFESSIONAL SAMPLE – NOT FOR SALE
LIPITOR®
atorvastatin calcium
tablets
10 mg*
7 TABLETS
PACKAGE NOT CHILD RESISTANT.
KEEP OUT OF REACH OF CHILDREN.
Rx only
Patient information enclosed
7 Tablets x 10 mg
Exp date
lot
LIPITOR®
atorvastatin calcium
tablets
10 mg*
7 Tablets x 10 mg

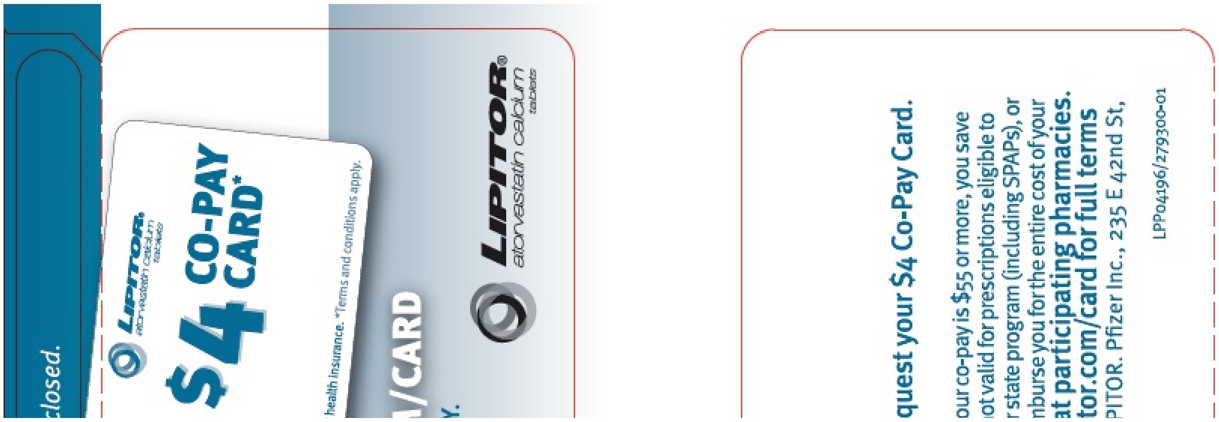
LIPITOR®
atorvastatin calcium
tablets
$4 CO-PAY CARD*
*Terms and conditions apply.
LIPITOR®
atorvastatin calcium
tablets
quest your $4 Co-Pay Card.
LPP04196/279300-01

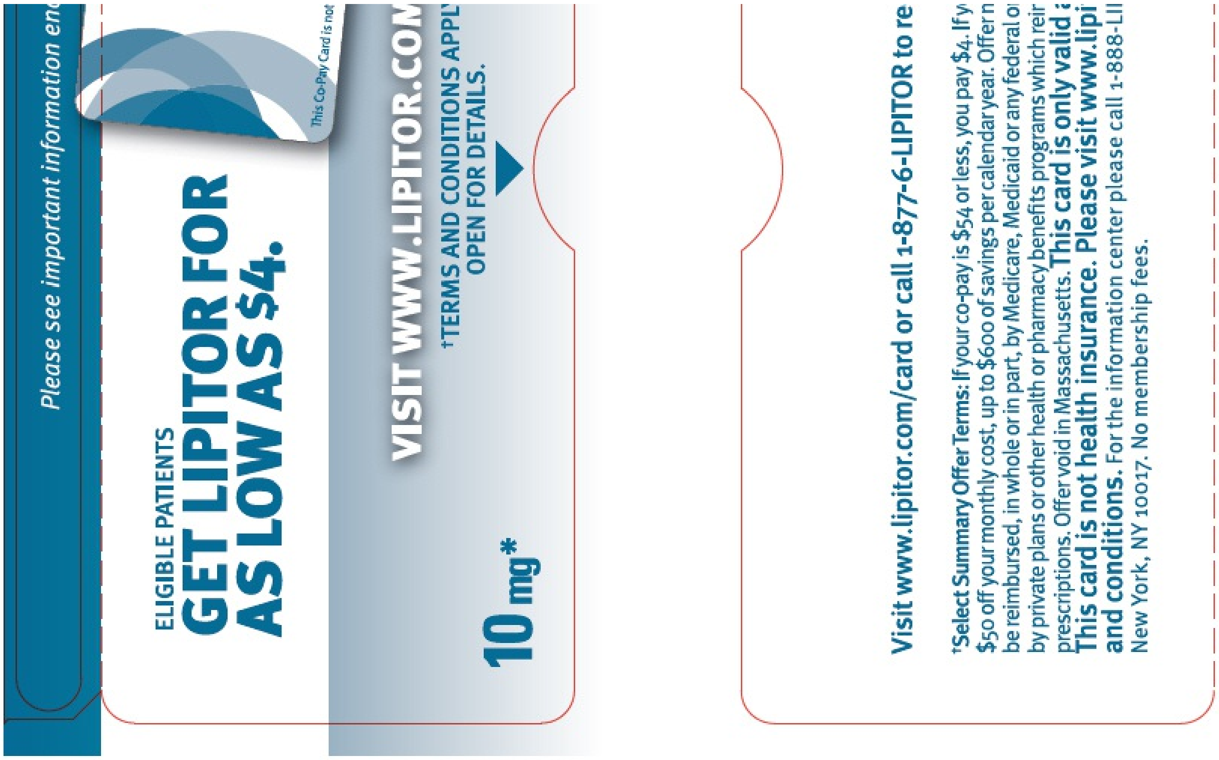

PRINCIPAL DISPLAY PANEL - 10 mg Tablet Packet

LIPITOR ®
(atorvastatin calcium) tablets

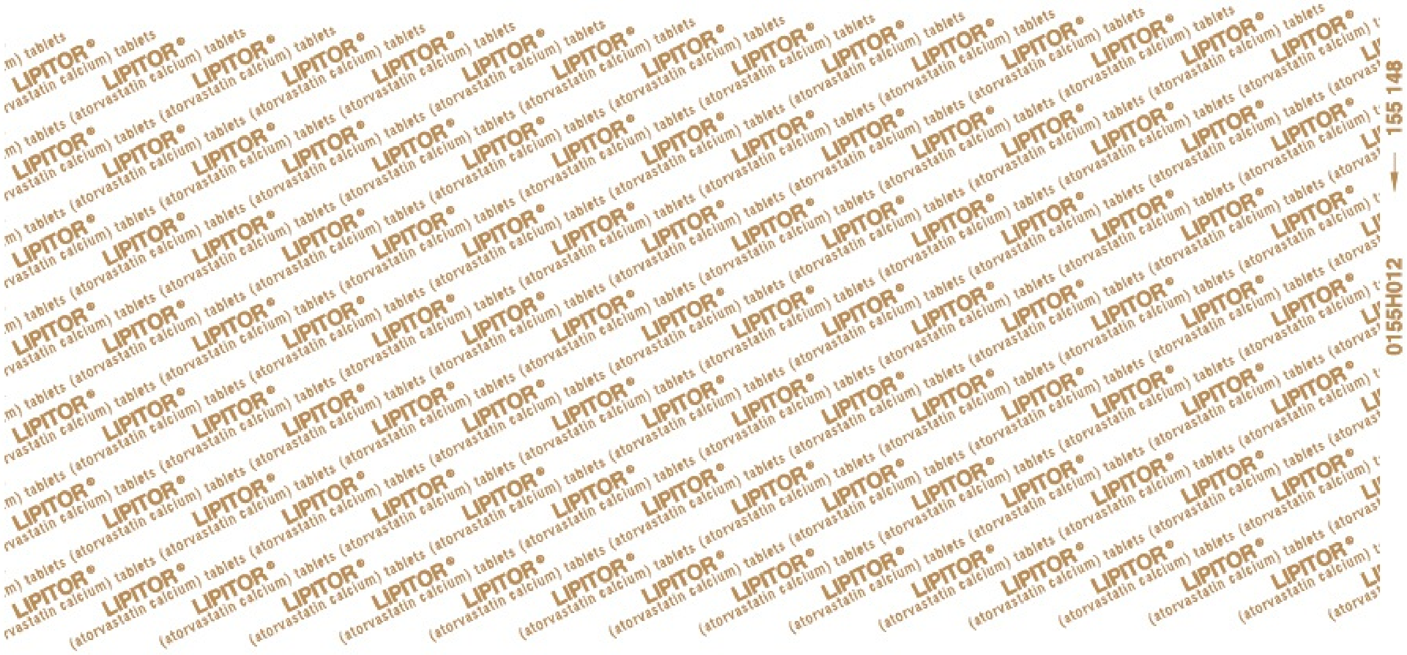

PRINCIPAL DISPLAY PANEL - 20 mg Tablet Bottle Label

NDC 0071-0156-23

Pfizer

Lipitor®
(atorvastatin calcium)

tablets

20 mg*

90 Tablets
Rx only

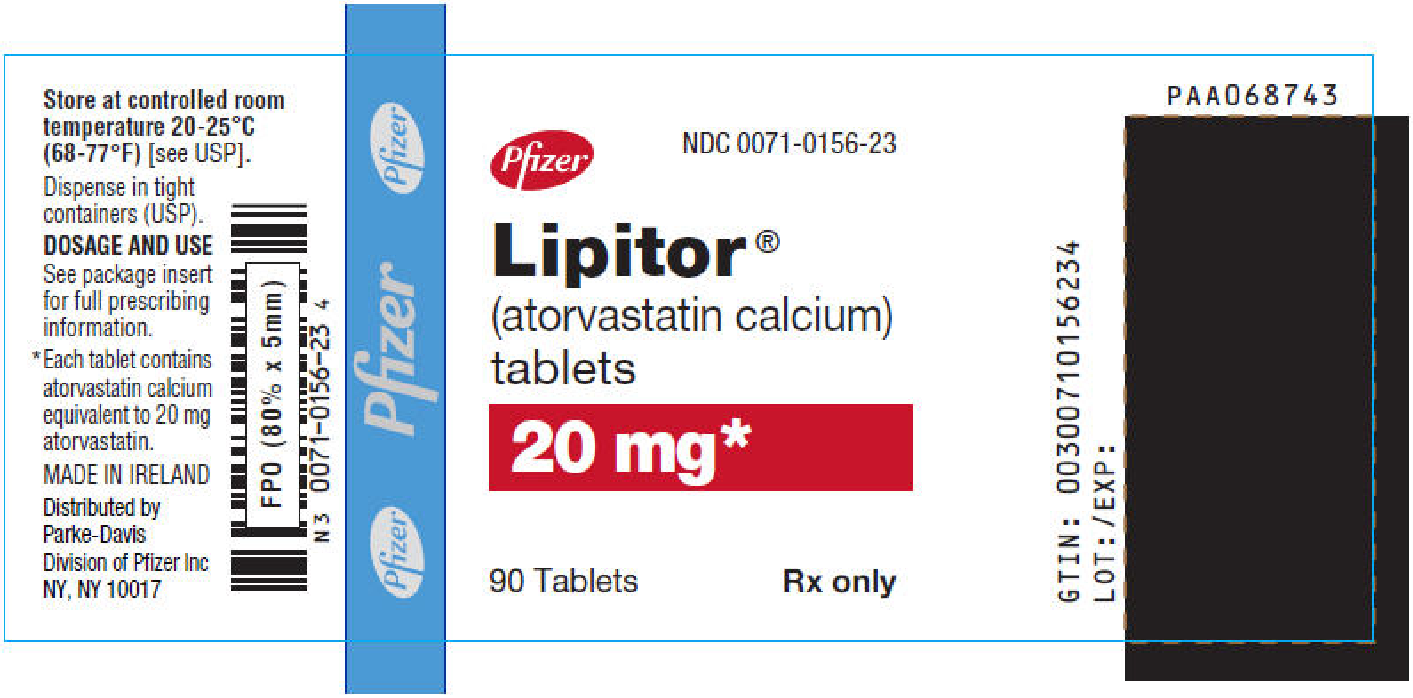

PRINCIPAL DISPLAY PANEL - 20 mg Tablet Blister Pack

Lipitor®

(Atorvastatin Calcium)
Tablet

20 mg

DISTRIBUTED BY:
PARKE-DAVIS
DIV OF PFIZER INC, NY, NY 10017
MADE IN IRELAND

EXP & LOT AREA

Lipitor®
(Atorvastatin Calcium)
Tablet
20 mg
DISTRIBUTED BY:
PARKE-DAVIS
DIV OF PFIZER INC, NY, NY 10017
MADE IN IRELAND

EXP & LOT AREA
FPO
RSS 10 mil Limited
N (01) 1 03 0071-0156-40 8

Lipitor®
(Atorvastatin Calcium)
Tablet
20 mg
DISTRIBUTED BY:
PARKE-DAVIS
DIV OF PFIZER INC, NY, NY 10017
MADE IN IRELAND
EXP & LOT AREA
FPO
RSS 10 mil Limited
N (01) 1 03 0071-0156-40 8

Lipitor®
(Atorvastatin Calcium)
Tablet
20 mg
DISTRIBUTED BY:
PARKE-DAVIS
DIV OF PFIZER INC, NY, NY 10017
MADE IN IRELAND
EXP & LOT AREA
FPO
RSS 10 mil Limited
N (01) 1 03 0071-0156-40 8

Lipitor®
(Atorvastatin Calcium)
Tablet
20 mg
DISTRIBUTED BY:
PARKE-DAVIS
DIV OF PFIZER INC, NY, NY 10017
MADE IN IRELAND
EXP & LOT AREA
FPO
RSS 10 mil Limited
N (01) 1 03 0071-0156-40 8

Lipitor®
(Atorvastatin Calcium)
Tablet
20 mg
DISTRIBUTED BY:
PARKE-DAVIS
DIV OF PFIZER INC, NY, NY 10017
MADE IN IRELAND
EXP & LOT AREA
FPO
RSS 10 mil Limited
N (01) 1 03 0071-0156-40 8

Lipitor®
(Atorvastatin Calcium)
Tablet
20 mg
DISTRIBUTED BY:
PARKE-DAVIS
DIV OF PFIZER INC, NY, NY 10017
MADE IN IRELAND
EXP & LOT AREA
FPO
RSS 10 mil Limited
N (01) 1 03 0071-0156-40 8

Lipitor®
(Atorvastatin Calcium)
Tablet
20 mg
DISTRIBUTED BY:
PARKE-DAVIS
DIV OF PFIZER INC, NY, NY 10017
MADE IN IRELAND
EXP & LOT AREA
FPO
RSS 10 mil Limited
N (01) 1 03 0071-0156-40 8

Lipitor®
(Atorvastatin Calcium)
Tablet
20 mg
DISTRIBUTED BY:
PARKE-DAVIS
DIV OF PFIZER INC, NY, NY 10017
MADE IN IRELAND
EXP & LOT AREA
FPO
RSS 10 mil Limited
N (01) 1 03 0071-0156-40 8

Lipitor®
(Atorvastatin Calcium)
Tablet
20 mg
DISTRIBUTED BY:
PARKE-DAVIS
DIV OF PFIZER INC, NY, NY 10017
MADE IN IRELAND
EXP & LOT AREA
FPO
RSS 10 mil Limited
N (01) 1 03 0071-0156-40 8

Lipitor®
(Atorvastatin Calcium)
Tablet
20 mg
MADE IN IRELAND
DIV OF PFIZER INC, NY, NY 10017
PARKE-DAVIS
DISTRIBUTED BY:
EXP & LOT
FPO
RSS 10 mil
N (01) 1 03 007

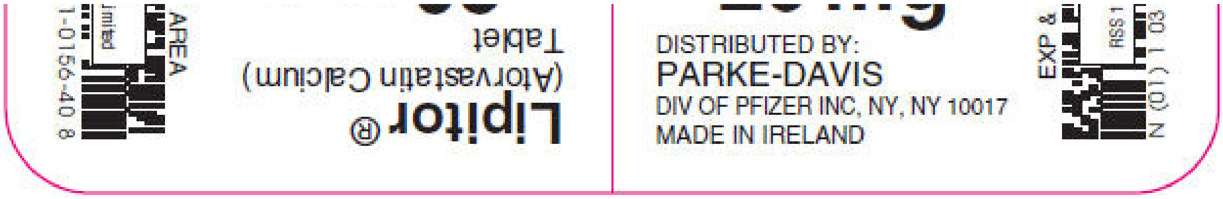

PRINCIPAL DISPLAY PANEL - 20 mg Blister Pack Carton

Pfizer

Lipitor®
(atorvastatin calcium)

20 mg*

tablets

For in-institution use only

100 Tablets

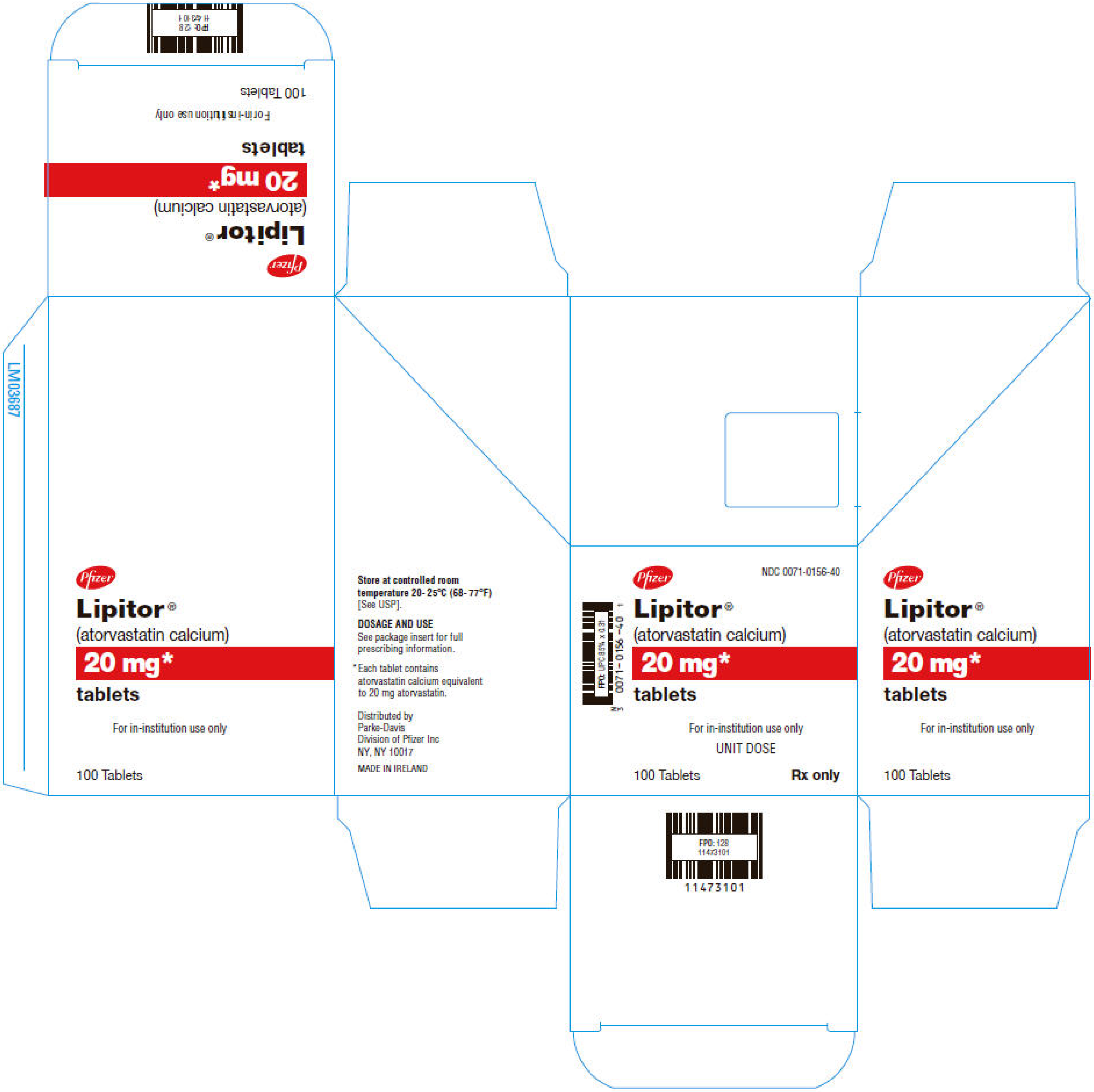

PRINCIPAL DISPLAY PANEL - 20 mg Tablet Packet Carton

NDC 0071-0156-96
PROFESSIONAL SAMPLE – NOT FOR SALE

LIPITOR®
atorvastatin calcium
tablets

20 mg*
7 TABLETS

PACKAGE NOT CHILD RESISTANT.
KEEP OUT OF REACH OF CHILDREN.

Rx only

Patient information enclosed

7 Tablets x 20 mg

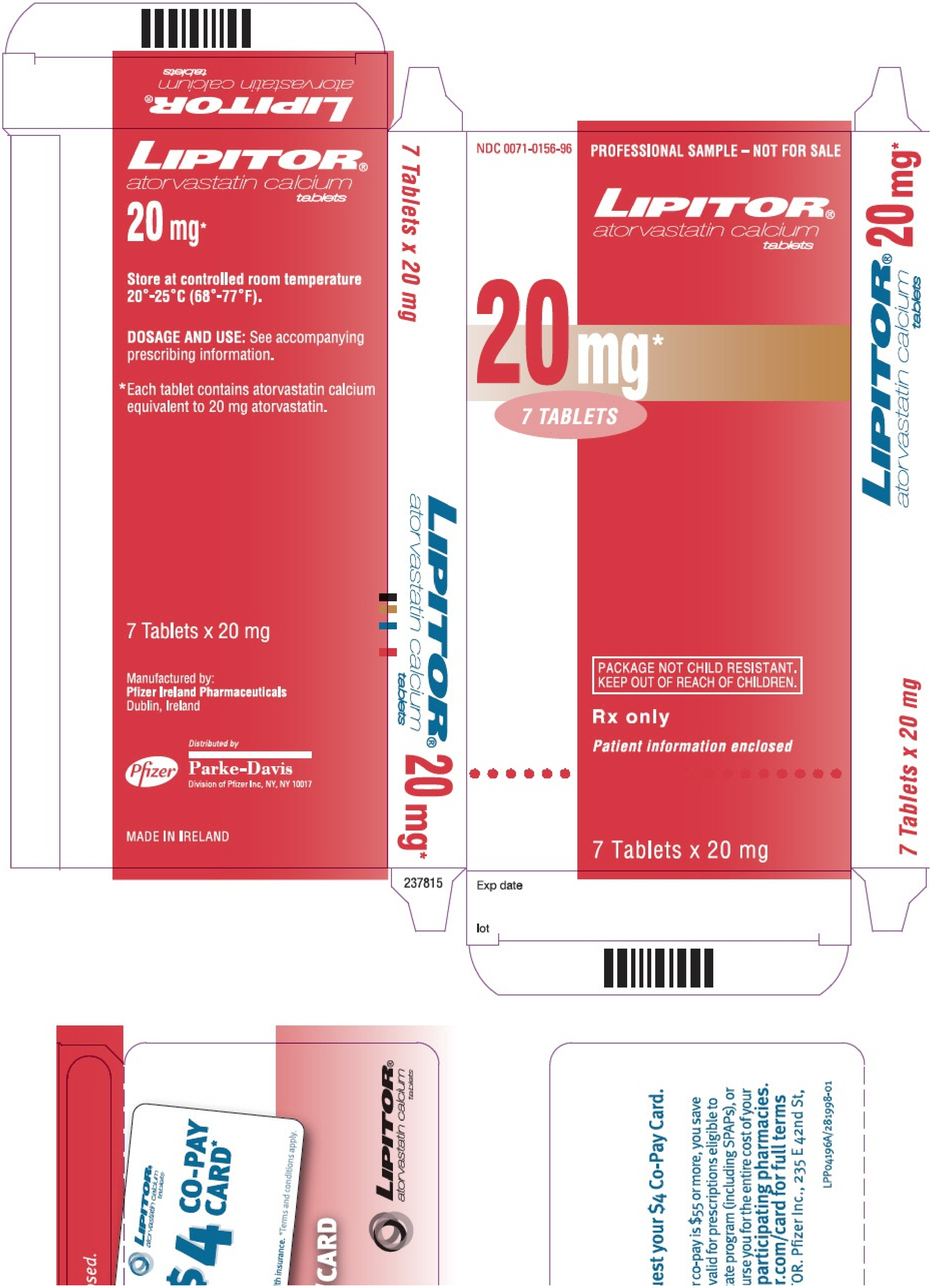
LIPITOR®
atorvastatin calcium
tablets
20 mg*
Store at controlled room temperature 20°-25°C (68°-77°F).
DOSAGE AND USE: See accompanying prescribing information.
*Each tablet contains atorvastatin calcium equivalent to 20 mg atorvastatin.
7 Tablets x 20 mg
Manufactured by:
Pfizer Ireland Pharmaceuticals
Dublin, Ireland
Distributed by
Pfizer
Parke-Davis
Division of Pfizer Inc, NY, NY 10017
MADE IN IRELAND
7 Tablets x 20 mg
LIPITOR® atorvastatin calcium tablets 20 mg*
237815
NDC 0071-0156-96
PROFESSIONAL SAMPLE – NOT FOR SALE
LIPITOR®
atorvastatin calcium
tablets
20 mg*
7 TABLETS
PACKAGE NOT CHILD RESISTANT.
KEEP OUT OF REACH OF CHILDREN.
Rx only
Patient information enclosed
7 Tablets x 20 mg
Exp date
lot
LIPITOR® atorvastatin calcium tablets 20 mg*
7 Tablets x 20 mg
$4 CO-PAY CARD*
LIPITOR atorvastatin calcium tablets
*Terms and conditions apply.
uest your $4 Co-Pay Card.
r co-pay is $55 or more, you save
valid for prescriptions eligible to
ate program (including SPAPs), or
urse you for the entire cost of your
participating pharmacies.
r.com/card for full terms
OR. Pfizer Inc., 235 E 42nd St,
LPP04196A/281998-01

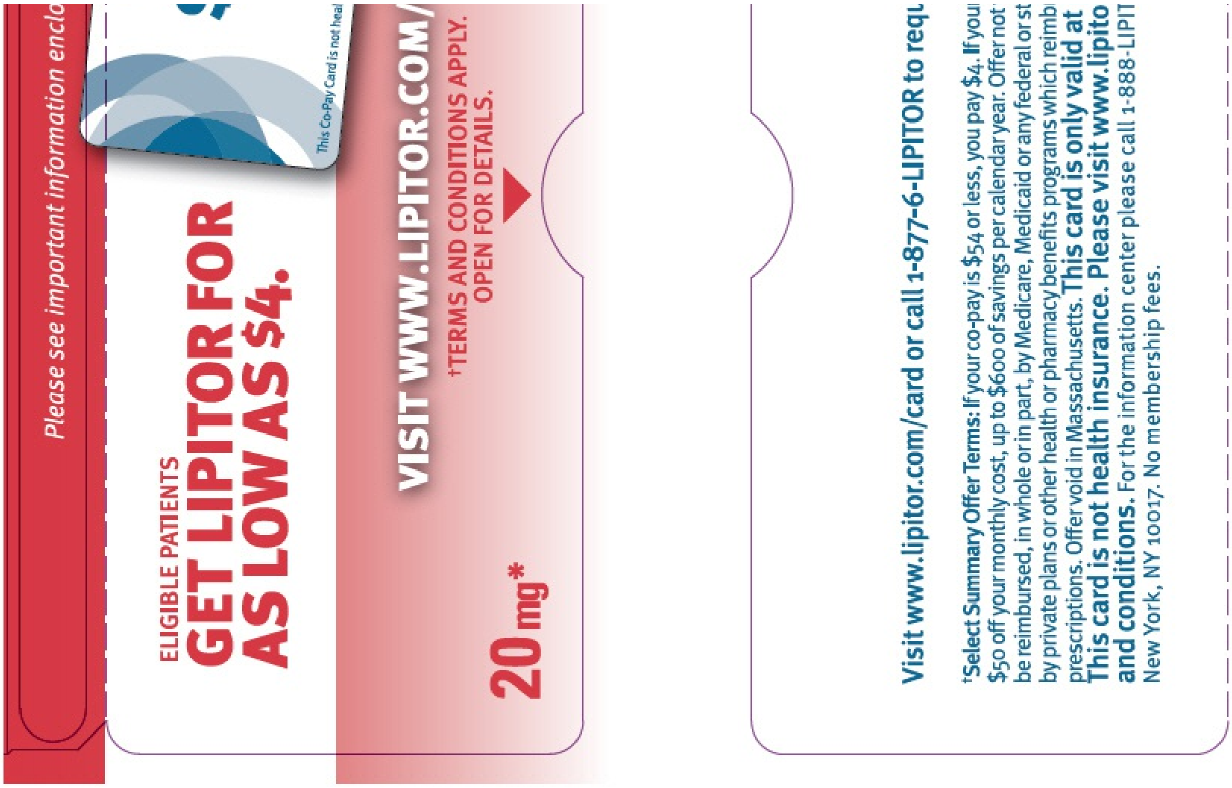

PRINCIPAL DISPLAY PANEL - 20 mg Tablet Packet

LIPITOR ®
(atorvastatin calcium) tablets

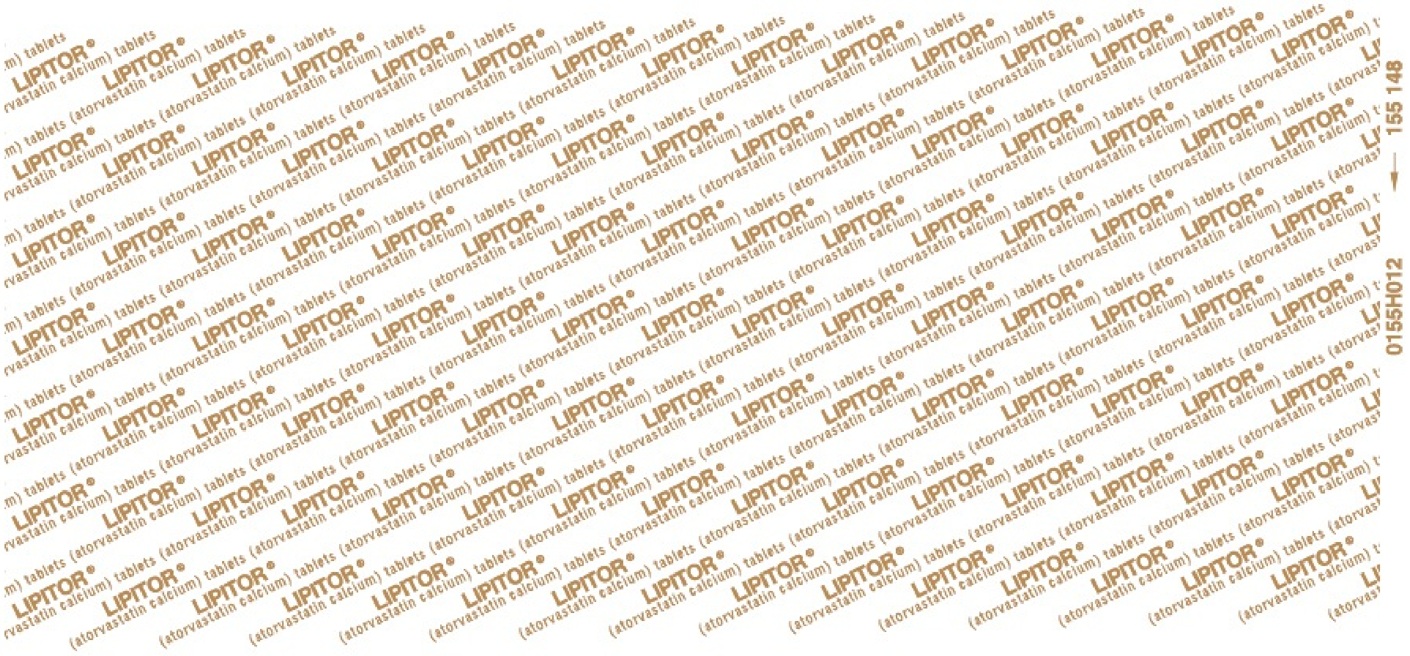

PRINCIPAL DISPLAY PANEL - 40 mg Tablet Bottle Label

NDC 0071-0157-23

Pfizer

Lipitor®

(atorvastatin calcium)

40 mg*

tablets

90 Tablets
Rx only

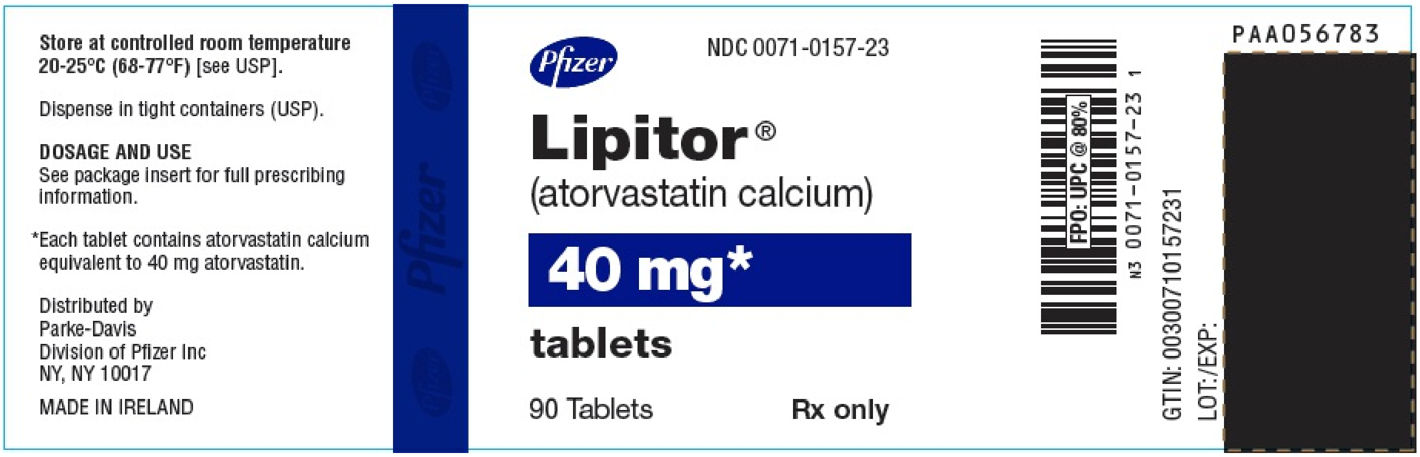

PRINCIPAL DISPLAY PANEL - 40 mg Tablet Blister Pack

Lipitor®

(Atorvastatin Calcium)
Tablet

40 mg

DISTRIBUTED BY:
PARKE-DAVIS
DIV OF PFIZER INC, NY, NY 10017
MADE IN IRELAND

EXP & LOT AREA

Lipitor®
(Atorvastatin Calcium)
Tablet
40 mg
DISTRIBUTED BY:
PARKE-DAVIS
DIV OF PFIZER INC, NY, NY 10017
MADE IN IRELAND
EXP & LOT AREA
FPO
RSS 10 mil Limited
N (01) 1 03 0071-0157-40 5

Lipitor®
(Atorvastatin Calcium)
Tablet
40 mg
DISTRIBUTED BY:
PARKE-DAVIS
DIV OF PFIZER INC, NY, NY 10017
MADE IN IRELAND
EXP & LOT AREA
FPO
RSS 10 mil Limited
N (01) 1 03 0071-0157-40 5

Lipitor®
(Atorvastatin Calcium)
Tablet
40 mg
DISTRIBUTED BY:
PARKE-DAVIS
DIV OF PFIZER INC, NY, NY 10017
MADE IN IRELAND
EXP & LOT AREA
FPO
RSS 10 mil Limited
N (01) 1 03 0071-0157-40 5

Lipitor®
(Atorvastatin Calcium)
Tablet
40 mg
DISTRIBUTED BY:
PARKE-DAVIS
DIV OF PFIZER INC, NY, NY 10017
MADE IN IRELAND
EXP & LOT AREA
FPO
RSS 10 mil Limited
N (01) 1 03 0071-0157-40 5

Lipitor®
(Atorvastatin Calcium)
Tablet
40 mg
DISTRIBUTED BY:
PARKE-DAVIS
DIV OF PFIZER INC, NY, NY 10017
MADE IN IRELAND
EXP & LOT AREA
FPO
RSS 10 mil Limited
N (01) 1 03 0071-0157-40 5

Lipitor®
(Atorvastatin Calcium)
Tablet
40 mg
DISTRIBUTED BY:
PARKE-DAVIS
DIV OF PFIZER INC, NY, NY 10017
MADE IN IRELAND
EXP & LOT AREA
FPO
RSS 10 mil Limited
N (01) 1 03 0071-0157-40 5

Lipitor®
(Atorvastatin Calcium)
Tablet
40 mg
DISTRIBUTED BY:
PARKE-DAVIS
DIV OF PFIZER INC, NY, NY 10017
MADE IN IRELAND
EXP & LOT AREA
FPO
RSS 10 mil Limited
N (01) 1 03 0071-0157-40 5

Lipitor®
(Atorvastatin Calcium)
Tablet
40 mg
DISTRIBUTED BY:
PARKE-DAVIS
DIV OF PFIZER INC, NY, NY 10017
MADE IN IRELAND
EXP & LOT AREA
FPO
RSS 10 mil Limited
N (01) 1 03 0071-0157-40 5

[illegible]
Tablet
40 mg
DISTRIBUTED BY:
PARKE-DAVIS
DIV OF PFIZER INC, NY, NY 10017
MADE IN IRELAND
EXP & LOT AREA
FPO
RSS 10 mil Limited
N (01) 1 03 0071-015[illegible]

Lipitor®
(Atorvastatin Calcium)
Tablet
40 mg
DISTRIBUTED BY:
[illegible]
EXP & LOT AREA
FPO
RSS 10 mil Limited
[illegible] 1 03 0071-0157-40 5

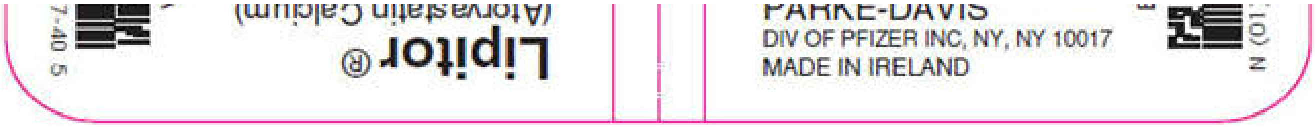

PRINCIPAL DISPLAY PANEL - 40 mg Blister Pack Carton

Pfizer

Lipitor®
(atorvastatin calcium)

40 mg*

tablets

For in-institution use only

100 Tablets

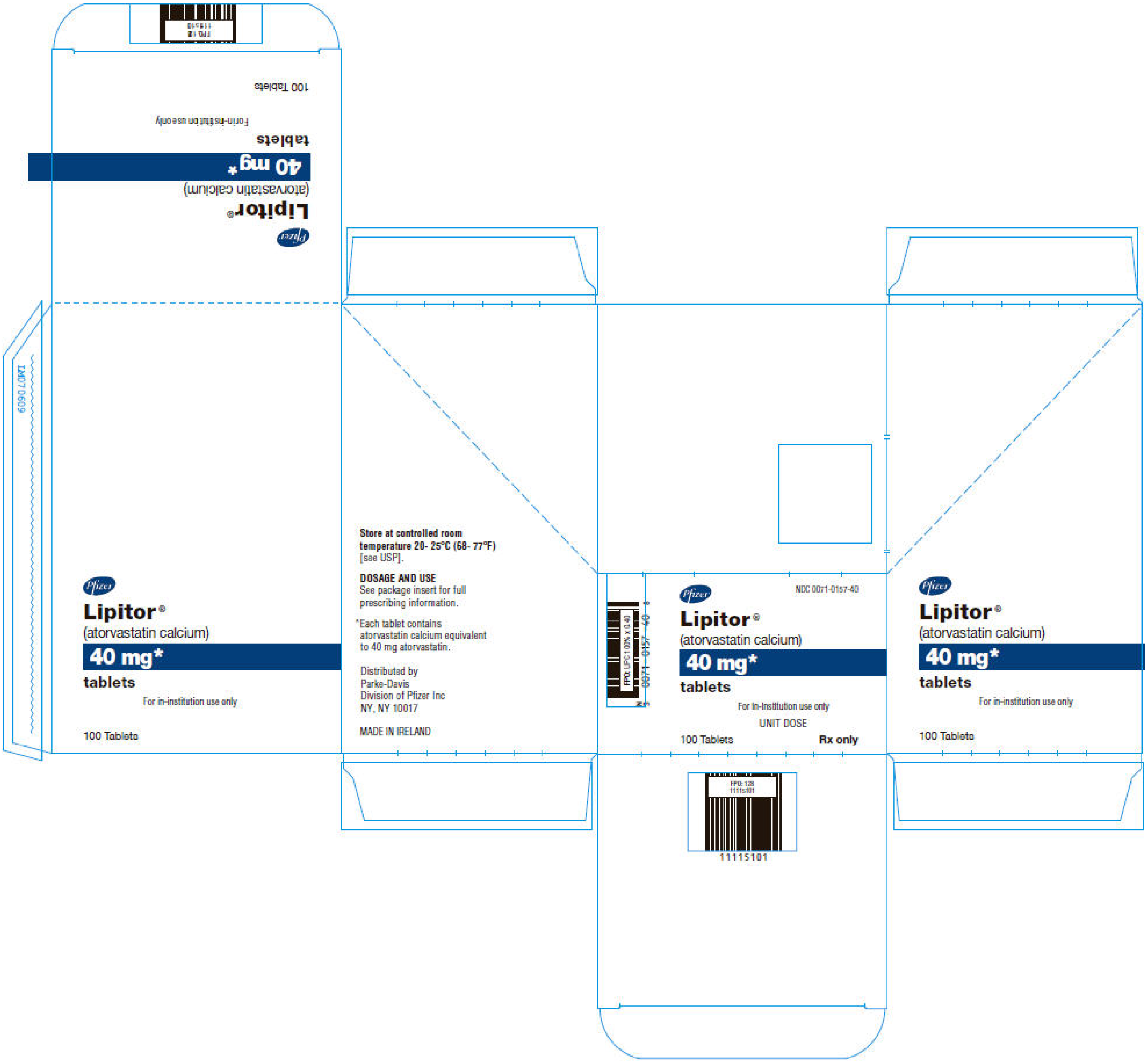

PRINCIPAL DISPLAY PANEL - 40 mg Tablet Packet Carton

NDC 0071-0157-97
PROFESSIONAL SAMPLE – NOT FOR SALE

LIPITOR®
atorvastatin calcium
tablets

40 mg*
7 TABLETS

PACKAGE NOT CHILD RESISTANT.
KEEP OUT OF REACH OF CHILDREN.

Rx only

Patient information enclosed

7 Tablets x 40 mg

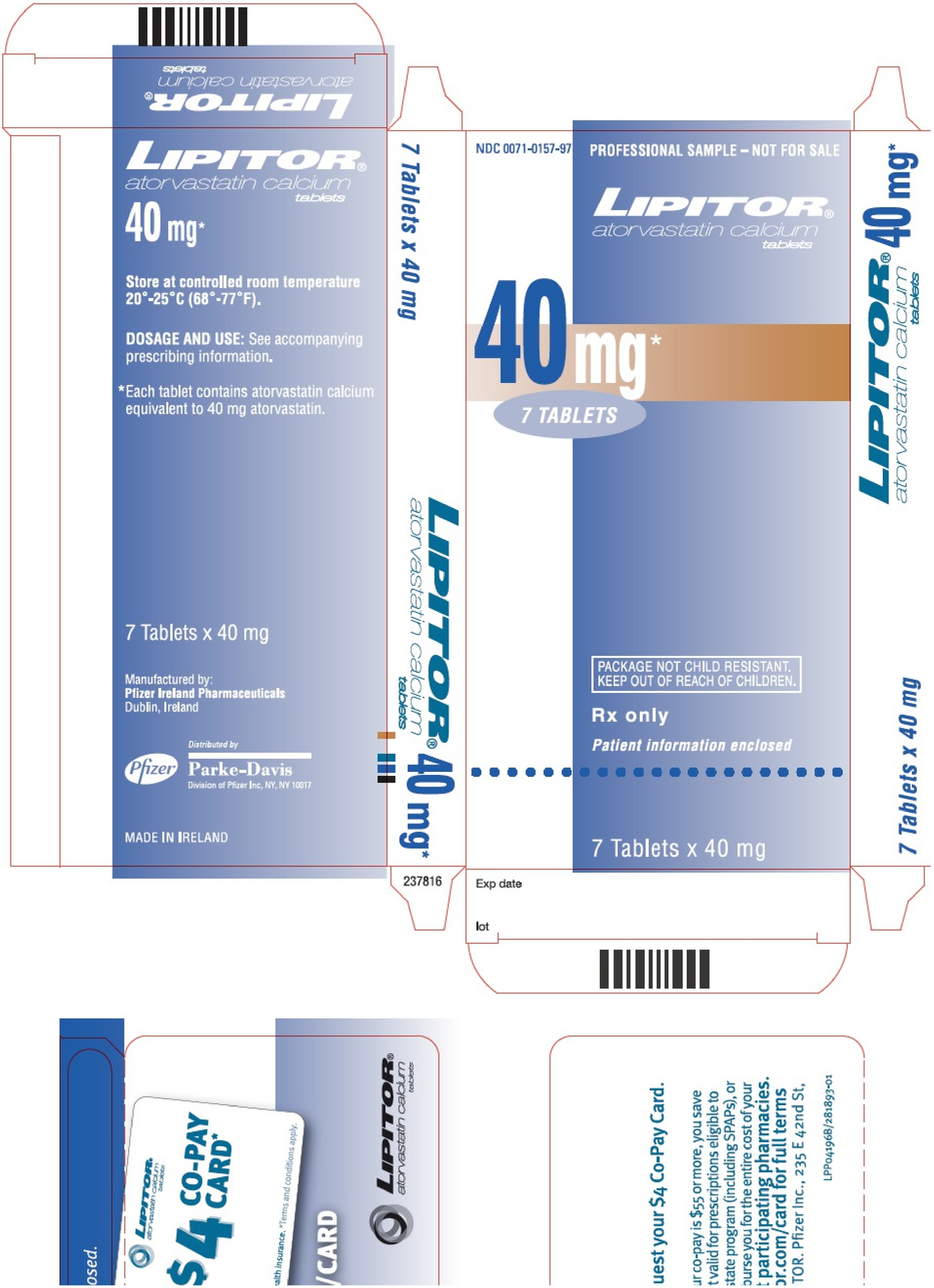
LIPITOR®
atorvastatin calcium
tablets
40 mg*
Store at controlled room temperature 20°-25°C (68°-77°F).
DOSAGE AND USE: See accompanying prescribing information.
*Each tablet contains atorvastatin calcium equivalent to 40 mg atorvastatin.
7 Tablets x 40 mg
Manufactured by:
Pfizer Ireland Pharmaceuticals
Dublin, Ireland
Distributed by
Pfizer
Parke-Davis
Division of Pfizer Inc, NY, NY 10017
MADE IN IRELAND
7 Tablets x 40 mg
LIPITOR® 40 mg*
atorvastatin calcium tablets
237816
NDC 0071-0157-97
PROFESSIONAL SAMPLE – NOT FOR SALE
LIPITOR®
atorvastatin calcium
tablets
40 mg*
7 TABLETS
PACKAGE NOT CHILD RESISTANT.
KEEP OUT OF REACH OF CHILDREN.
Rx only
Patient information enclosed
7 Tablets x 40 mg
Exp date
lot
LIPITOR® 40 mg*
atorvastatin calcium tablets
7 Tablets x 40 mg
LIPITOR atorvastatin calcium tablets
$4 CO-PAY CARD*
*Terms and conditions apply.
/CARD
LIPITOR® atorvastatin calcium tablets
uest your $4 Co-Pay Card.
ur co-pay is $55 or more, you save
t valid for prescriptions eligible to
state program (including SPAPs), or
burse you for the entire cost of your
t participating pharmacies.
or.com/card for full terms
TOR. Pfizer Inc., 235 E 42nd St,
LPP04196B/281893-01

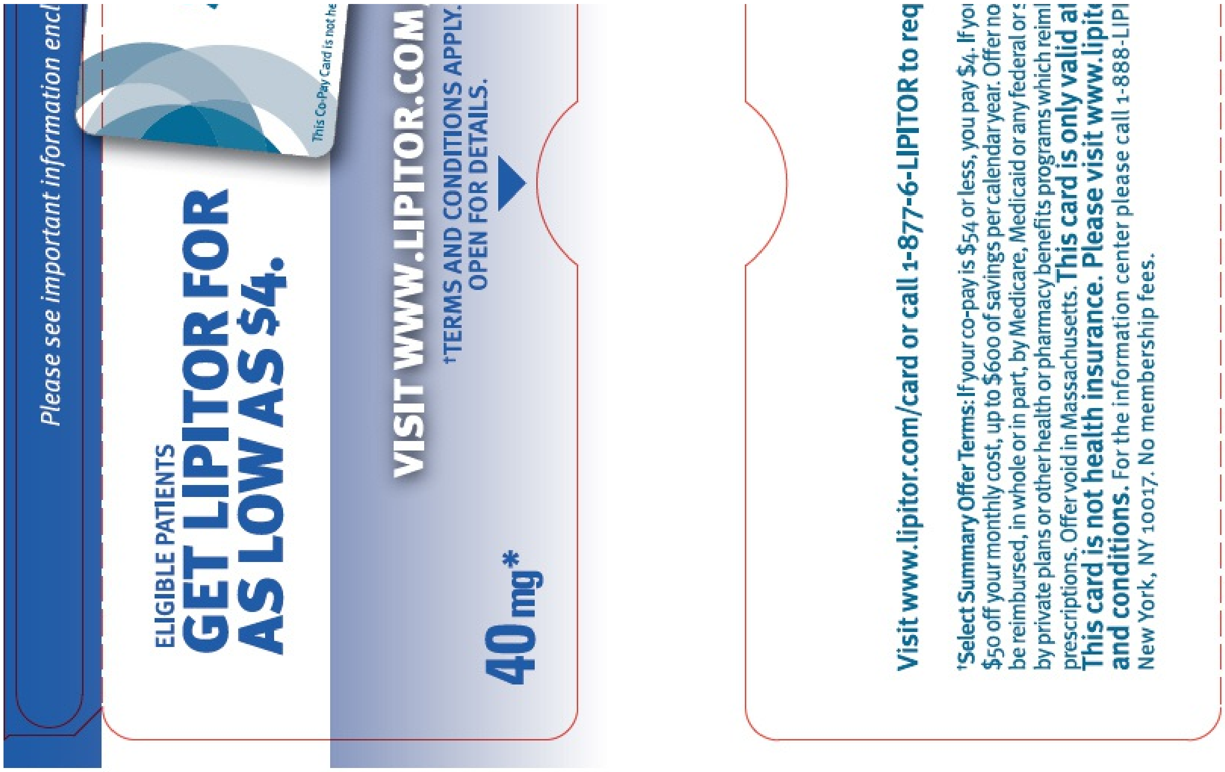

PRINCIPAL DISPLAY PANEL - 40 mg Tablet Packet

LIPITOR ®
(atorvastatin calcium) tablets

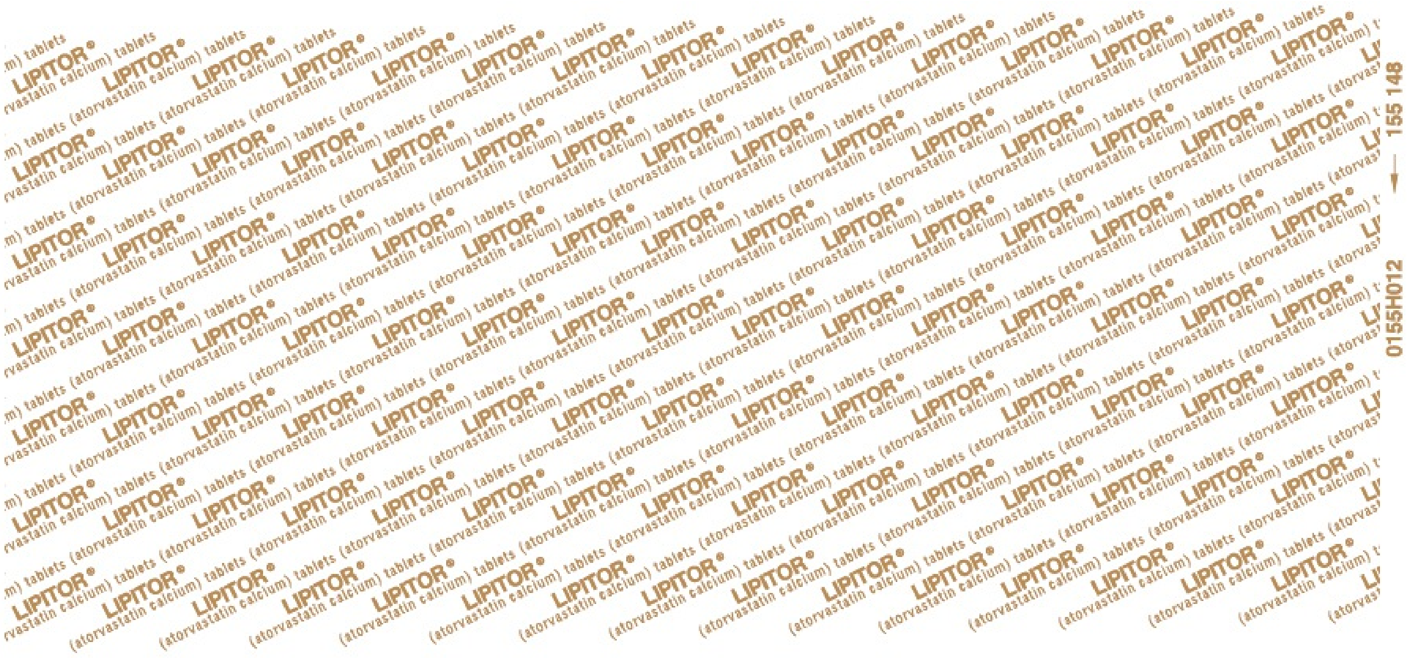

PRINCIPAL DISPLAY PANEL - 80 mg Tablet Bottle Label

NDC 0071-0158-23

Pfizer

Lipitor®

(atorvastatin calcium)

80 mg*

tablets

90 Tablets
Rx only

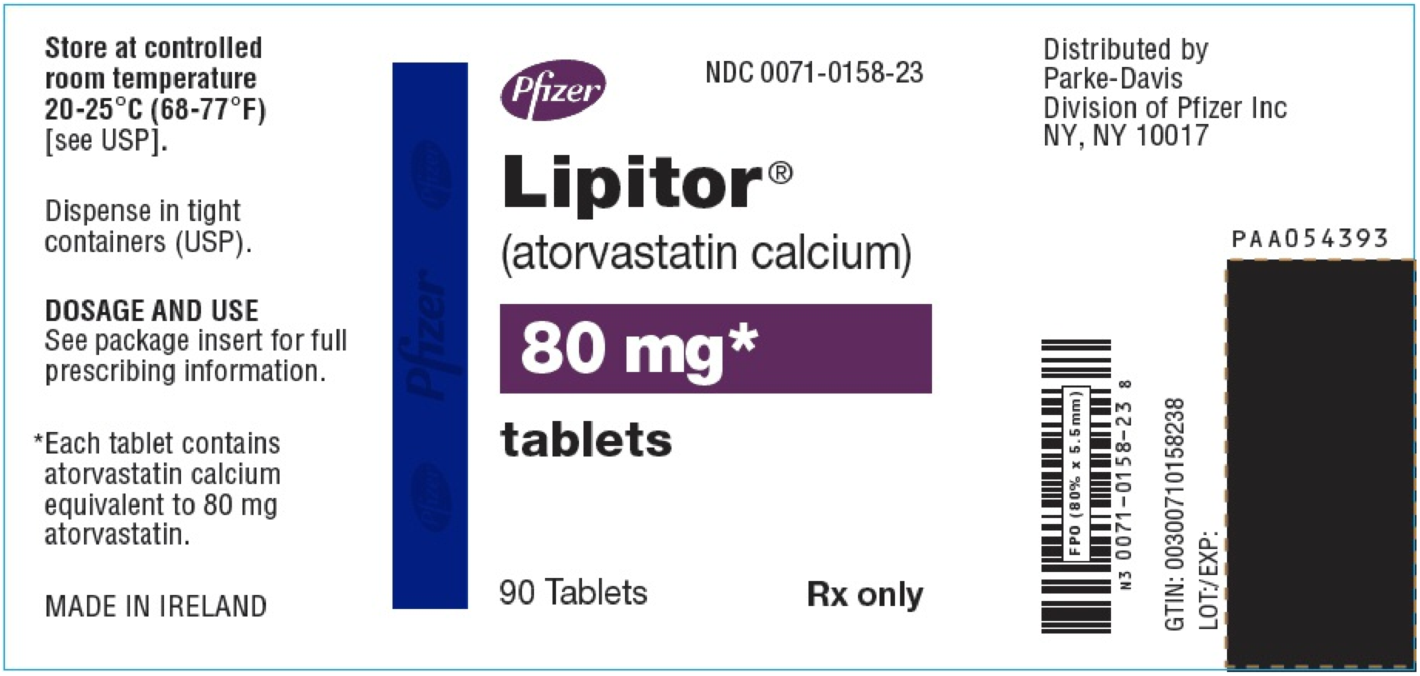

PRINCIPAL DISPLAY PANEL - 80 mg Tablet Blister Pack

Lipitor®

(Atorvastatin Calcium)
Tablet

80 mg

DISTRIBUTED BY:
PARKE-DAVIS
DIV OF PFIZER INC, NY, NY 10017
MADE IN IRELAND

EXP & LOT AREA

Lipitor®
(Atorvastatin Calcium)
Tablet
80mg
DISTRIBUTED BY:
PARKE-DAVIS
DIV OF PFIZER INC, NY, NY 10017
MADE IN IRELAND

EXP & LOT AREA
FPO
RSS 12 mil Limited
N (01) 1 03 0071-0158-92 1

Lipitor®
(Atorvastatin Calcium)
Tablet
80mg
DISTRIBUTED BY:
PARKE-DAVIS
DIV OF PFIZER INC, NY, NY 10017
MADE IN IRELAND
EXP & LOT AREA
FPO
RSS 12 mil Limited
N (01) 1 03 0071-0158-92 1

Lipitor®
(Atorvastatin Calcium)
Tablet
80mg
DISTRIBUTED BY:
PARKE-DAVIS
DIV OF PFIZER INC, NY, NY 10017
MADE IN IRELAND
EXP & LOT AREA
FPO
RSS 12 mil Limited
N (01) 1 03 0071-0158-92 1

Lipitor®
(Atorvastatin Calcium)
Tablet
80mg
DISTRIBUTED BY:
PARKE-DAVIS
DIV OF PFIZER INC, NY, NY 10017
MADE IN IRELAND
EXP & LOT AREA
FPO
RSS 12 mil Limited
N (01) 1 03 0071-0158-92 1

Lipitor®
(Atorvastatin Calcium)
Tablet
80mg
DISTRIBUTED BY:
PARKE-DAVIS
DIV OF PFIZER INC, NY, NY 10017
MADE IN IRELAND
EXP & LOT AREA
FPO
RSS 12 mil Limited
N (01) 1 03 0071-0158-92 1

Lipitor®
(Atorvastatin Calcium)
Tablet
80mg
DISTRIBUTED BY:
PARKE-DAVIS
DIV OF PFIZER INC, NY, NY 10017
MADE IN IRELAND
EXP & LOT AREA
FPO
RSS 12 mil Limited
N (01) 1 03 0071-0158-92 1

Lipitor®
(Atorvastatin Calcium)
Tablet
80mg
DISTRIBUTED BY:
PARKE-DAVIS
DIV OF PFIZER INC, NY, NY 10017
MADE IN IRELAND
EXP & LOT AREA
FPO
RSS 12 mil Limited
N (01) 1 03 0071-0158-92 1

Lipitor®
(Atorvastatin Calcium)
Tablet
80mg
DISTRIBUTED BY:
EXP & LOT AREA
FPO
RSS 12 mil Limited

Tablet
80mg
DISTRIBUTED BY:
PARKE-DAVIS
DIV OF PFIZER INC, NY, NY 10017
MADE IN IRELAND
EXP & LOT AREA
FPO
RSS 12 mil Limited
N (01) 1 03 0071-0158-[illegible]

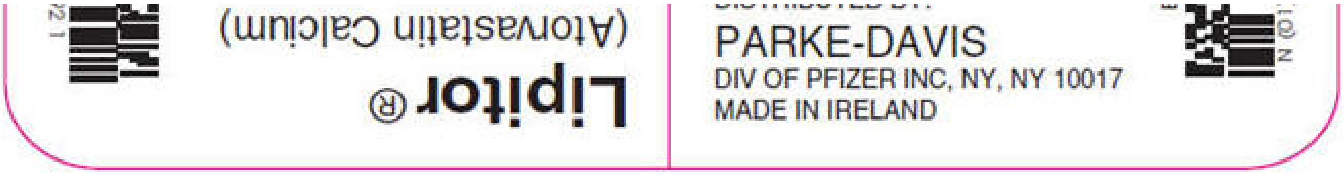

PRINCIPAL DISPLAY PANEL - 80 mg Blister Pack Carton

Pfizer

Lipitor®
(atorvastatin calcium)

80 mg*

tablets

For in-institution use only

64 Tablets

64 Tablets

For in-institution use only

tablets

80 mg*

(atorvastatin calcium)

Lipitor®

Pfizer

1M0 70 609

Pfizer

Lipitor®

(atorvastatin calcium)

80 mg*

tablets

For in-institution use only

64 Tablets

Store at controlled room temperature 20- 25°C (68- 77°F) [see USP].

DOSAGE AND USE
See package insert for full prescribing information.

*Each tablet contains atorvastatin calcium equivalent to 80 mg atorvastatin.

Distributed by
Parke-Davis
Division of Pfizer Inc
NY, NY 10017

MADE IN IRELAND

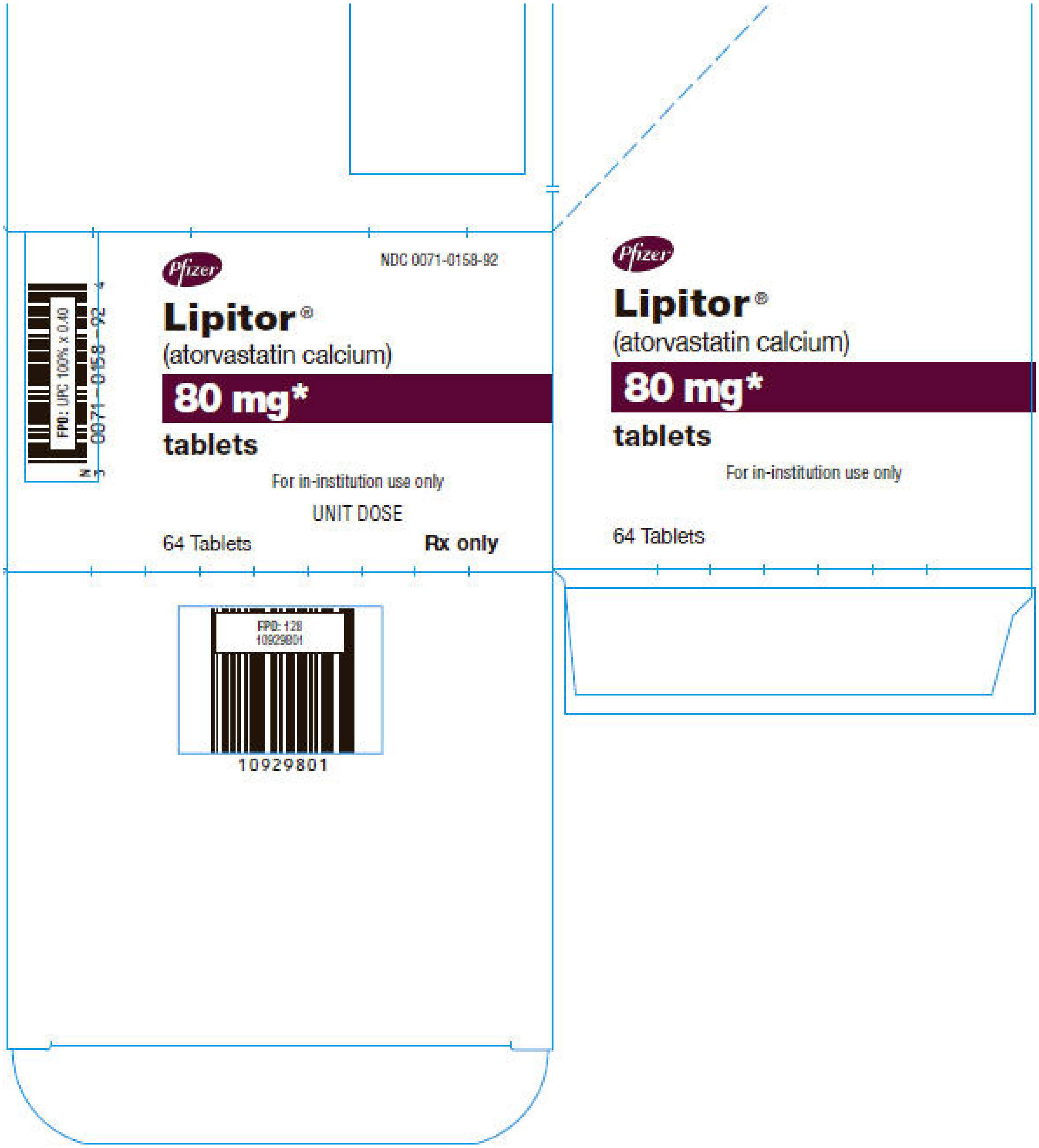

LIPITOR

atorvastatin calcium tablet, film coated

Product Information

**Product Type**	HUMAN PRESCRIPTION DRUG	**Item Code (Source)**	NDC:0071-0155
**Route of Administration**	ORAL		

Active Ingredient/Active Moiety

Ingredient Name	Basis of Strength	Strength
**ATORVASTATIN CALCIUM TRIHYDRATE** (UNII: 48A5M73Z4Q) (ATORVASTATIN - UNII:A0JWA85V8F)	ATORVASTATIN	10 mg

Inactive Ingredients	
**Ingredient Name**	**Strength**
**CALCIUM CARBONATE** (UNII: H0G9379FGK)	
**CANDELILLA WAX** (UNII: WL0328HX19)	
**CROSCARMELLOSE SODIUM** (UNII: M28OL1HH48)	
**HYDROXYPROPYL CELLULOSE (1200000 MW)** (UNII: RFW2ET671P)	
**LACTOSE MONOHYDRATE** (UNII: EWQ57Q8I5X)	
**MAGNESIUM STEARATE** (UNII: 70097M6I30)	
**MICROCRYSTALLINE CELLULOSE** (UNII: OP1R32D61U)	
**HYPROMELLOSE, UNSPECIFIED** (UNII: 3NXW29V3WO)	
**POLYETHYLENE GLYCOL, UNSPECIFIED** (UNII: 3WJQ0SDW1A)	
**TALC** (UNII: 7SEV7J4R1U)	
**TITANIUM DIOXIDE** (UNII: 15FIX9V2JP)	
**POLYSORBATE 80** (UNII: 6OZP39ZG8H)	

Product Characteristics			
**Color**	WHITE	**Score**	no score
**Shape**	OVAL (elliptical)	**Size**	10mm
**Flavor**		**Imprint Code**	PD;155;10
**Contains**			

Packaging				
**#**	**Item Code**	**Package Description**	**Marketing Start Date**	**Marketing End Date**
1	NDC:0071-0155-23	90 in 1 BOTTLE; Type 0: Not a Combination Product	12/17/1996	
2	NDC:0071-0155-34	5000 in 1 BOTTLE; Type 0: Not a Combination Product	12/17/1996	02/28/2015
3	NDC:0071-0155-40	100 in 1 CARTON	12/17/1996	
3		1 in 1 BLISTER PACK; Type 0: Not a Combination Product		
4	NDC:0071-0155-10	1000 in 1 BOTTLE; Type 0: Not a Combination Product	12/17/1996	
5	NDC:0071-0155-97	1 in 1 CARTON	12/17/1996	
5		7 in 1 PACKET; Type 0: Not a Combination Product		

Marketing Information

Marketing Category	Application Number or Monograph Citation	Marketing Start Date	Marketing End Date
NDA	NDA020702	12/17/1996	

LIPITOR

atorvastatin calcium tablet, film coated

Product Information			
**Product Type**	HUMAN PRESCRIPTION DRUG	**Item Code (Source)**	NDC:0071-0156
**Route of Administration**	ORAL		

Active Ingredient/Active Moiety		
**Ingredient Name**	**Basis of Strength**	**Strength**
**ATORVASTATIN CALCIUM TRIHYDRATE** (UNII: 48A5M73Z4Q) (ATORVASTATIN - UNII:A0JWA85V8F)	ATORVASTATIN	20 mg

Inactive Ingredients	
**Ingredient Name**	**Strength**
**CALCIUM CARBONATE** (UNII: H0G9379FGK)	
**CANDELILLA WAX** (UNII: WL0328HX19)	
**CROSCARMELLOSE SODIUM** (UNII: M28OL1HH48)	
**HYDROXYPROPYL CELLULOSE (1200000 MW)** (UNII: RFW2ET671P)	
**LACTOSE MONOHYDRATE** (UNII: EWQ57Q8I5X)	
**MAGNESIUM STEARATE** (UNII: 70097M6I30)	
**MICROCRYSTALLINE CELLULOSE** (UNII: OP1R32D61U)	
**HYPROMELLOSE, UNSPECIFIED** (UNII: 3NXW29V3WO)	
**POLYETHYLENE GLYCOL, UNSPECIFIED** (UNII: 3WJQ0SDW1A)	
**TALC** (UNII: 7SEV7J4R1U)	
**TITANIUM DIOXIDE** (UNII: 15FIX9V2JP)	
**POLYSORBATE 80** (UNII: 6OZP39ZG8H)	

Product Characteristics			
**Color**	WHITE	**Score**	no score
**Shape**	OVAL (elliptical)	**Size**	12mm
**Flavor**		**Imprint Code**	PD;156;20
**Contains**			

Packaging				
**#**	**Item Code**	**Package Description**	**Marketing Start Date**	**Marketing End Date**
1	NDC:0071-0156-23	90 in 1 BOTTLE; Type 0: Not a Combination Product	12/17/1996	
2	NDC:0071-0156-94	5000 in 1 BOTTLE; Type 0: Not a Combination Product	12/17/1996	02/26/2016
3	NDC:0071-0156-40	100 in 1 CARTON	12/17/1996	
3		1 in 1 BLISTER PACK; Type 0: Not a Combination Product		
4	NDC:0071-0156-10	1000 in 1 BOTTLE; Type 0: Not a Combination Product	12/17/1996	
5	NDC:0071-0156-96	1 in 1 CARTON	12/17/1996	
5		7 in 1 PACKET; Type 0: Not a Combination Product		

Marketing Information

Marketing Category	Application Number or Monograph Citation	Marketing Start Date	Marketing End Date
NDA	NDA020702	12/17/1996	

LIPITOR

atorvastatin calcium tablet, film coated

Product Information

**Product Type**	HUMAN PRESCRIPTION DRUG	**Item Code (Source)**	NDC:0071-0157
**Route of Administration**	ORAL		

Active Ingredient/Active Moiety

Ingredient Name	Basis of Strength	Strength
**ATORVASTATIN CALCIUM TRIHYDRATE** (UNII: 48A5M73Z4Q) (ATORVASTATIN - UNII:A0JWA85V8F)	ATORVASTATIN	40 mg

Inactive Ingredients

Ingredient Name	Strength
**CALCIUM CARBONATE** (UNII: H0G9379FGK)	
**CANDELILLA WAX** (UNII: WL0328HX19)	
**CROSCARMELLOSE SODIUM** (UNII: M28OL1HH48)	
**HYDROXYPROPYL CELLULOSE (1200000 MW)** (UNII: RFW2ET671P)	
**LACTOSE MONOHYDRATE** (UNII: EWQ57Q8I5X)	
**MAGNESIUM STEARATE** (UNII: 70097M6I30)	
**MICROCRYSTALLINE CELLULOSE** (UNII: OP1R32D61U)	
**HYPROMELLOSE, UNSPECIFIED** (UNII: 3NXW29V3WO)	
**POLYETHYLENE GLYCOL, UNSPECIFIED** (UNII: 3WJQ0SDW1A)	
**TALC** (UNII: 7SEV7J4R1U)	
**TITANIUM DIOXIDE** (UNII: 15FIX9V2JP)	
**POLYSORBATE 80** (UNII: 6OZP39ZG8H)	

Product Characteristics

**Color**	WHITE	**Score**	no score
**Shape**	OVAL (elliptical)	**Size**	15mm
**Flavor**		**Imprint Code**	PD;157;40
**Contains**			

Packaging

#	Item Code	Package Description	Marketing Start Date	Marketing End Date
1	NDC:0071-0157-23	90 in 1 BOTTLE; Type 0: Not a Combination Product	12/17/1996	
2	NDC:0071-0157-73	500 in 1 BOTTLE; Type 0: Not a Combination Product	12/17/1996	
3	NDC:0071-0157-88	2500 in 1 BOTTLE; Type 0: Not a Combination Product	12/17/1996	01/31/2016
4	NDC:0071-0157-40	100 in 1 CARTON	12/17/1996	
4		1 in 1 BLISTER PACK; Type 0: Not a Combination Product		
5	NDC:0071-0157-97	1 in 1 CARTON	12/17/1996	
5		7 in 1 PACKET; Type 0: Not a Combination Product		

Marketing Information

Marketing Category	Application Number or Monograph Citation	Marketing Start Date	Marketing End Date
NDA	NDA020702	12/17/1996	

LIPITOR

atorvastatin calcium tablet, film coated

Product Information

**Product Type**	HUMAN PRESCRIPTION DRUG	**Item Code (Source)**	NDC:0071-0158
**Route of Administration**	ORAL		

Active Ingredient/Active Moiety

Ingredient Name	Basis of Strength	Strength
**ATORVASTATIN CALCIUM TRIHYDRATE** (UNII: 48A5M73Z4Q) (ATORVASTATIN - UNII:A0JWA85V8F)	ATORVASTATIN	80 mg

Inactive Ingredients

Ingredient Name	Strength
**CALCIUM CARBONATE** (UNII: H0G9379FGK)	
**CANDELILLA WAX** (UNII: WL0328HX19)	
**CROSCARMELLOSE SODIUM** (UNII: M28OL1HH48)	
**HYDROXYPROPYL CELLULOSE (1200000 MW)** (UNII: RFW2ET671P)	
**LACTOSE MONOHYDRATE** (UNII: EWQ57Q8I5X)	
**MAGNESIUM STEARATE** (UNII: 70097M6I30)	
**MICROCRYSTALLINE CELLULOSE** (UNII: OP1R32D61U)	
**HYPROMELLOSE, UNSPECIFIED** (UNII: 3NXW29V3WO)	
**POLYETHYLENE GLYCOL, UNSPECIFIED** (UNII: 3WJQ0SDW1A)	
**TALC** (UNII: 7SEV7J4R1U)	
**TITANIUM DIOXIDE** (UNII: 15FIX9V2JP)	
**POLYSORBATE 80** (UNII: 6OZP39ZG8H)	

Product Characteristics

**Color**	WHITE	**Score**	no score
**Shape**	OVAL (elliptical)	**Size**	19mm
**Flavor**		**Imprint Code**	PD;158;80
**Contains**			

Packaging

#	Item Code	Package Description	Marketing Start Date	Marketing End Date
1	NDC:0071-0158-23	90 in 1 BOTTLE; Type 0: Not a Combination Product	04/07/2000	
2	NDC:0071-0158-73	500 in 1 BOTTLE; Type 0: Not a Combination Product	04/07/2000	

3	NDC:0071-0158-88	2500 in 1 BOTTLE; Type 0: Not a Combination Product	04/07/2000	04/30/2015
4	NDC:0071-0158-92	64 in 1 CARTON	04/07/2000	
4		1 in 1 BLISTER PACK; Type 0: Not a Combination Product		

Marketing Information

Marketing Category	Application Number or Monograph Citation	Marketing Start Date	Marketing End Date
NDA	NDA020702	04/07/2000	

Labeler - Parke-Davis Div of Pfizer Inc (829076962)

Registrant - Pfizer Inc (113480771)

Establishment

Name	Address	ID/FEI	Business Operations
Pfizer Ireland Pharmaceuticals		989811526	API MANUFACTURE(0071-0155, 0071-0156, 0071-0157, 0071-0158)

Establishment

Name	Address	ID/FEI	Business Operations
Pfizer Ireland Pharmaceuticals		896090987	ANALYSIS(0071-0155, 0071-0156, 0071-0157, 0071-0158) , API MANUFACTURE(0071-0155, 0071-0156, 0071-0157, 0071-0158) , MANUFACTURE(0071-0155, 0071-0156, 0071-0157, 0071-0158)

Establishment

Name	Address	ID/FEI	Business Operations
Pfizer Pharmaceuticals LLC		829084552	ANALYSIS(0071-0158, 0071-0157, 0071-0156, 0071-0155) , MANUFACTURE(0071-0158, 0071-0157, 0071-0156, 0071-0155) , PACK(0071-0158, 0071-0157, 0071-0156, 0071-0155)

Establishment

Name	Address	ID/FEI	Business Operations
Pfizer Asia Pacific PTE LTD		894677996	API MANUFACTURE(0071-0155, 0071-0156, 0071-0157, 0071-0158)

Establishment

Name	Address	ID/FEI	Business Operations
Pfizer Manufacturing Deutschland GmbH (Betriebsstätte Freiburg)		341970073	ANALYSIS(0071-0155, 0071-0156, 0071-0157, 0071-0158) , API MANUFACTURE(0071-0155, 0071-0156, 0071-0157, 0071-0158) , MANUFACTURE(0071-0155, 0071-0156, 0071-0157, 0071-0158) , PACK(0071-0155, 0071-0156, 0071-0157, 0071-0158)

Establishment

Name	Address	ID/FEI	Business Operations
Pfizer Inc		943955690	ANALYSIS(0071-0155, 0071-0156, 0071-0157, 0071-0158)
