# Supplementary material for: A comparison of the content and primary literature support for online medication information provided by Lexicomp and Wikipedia
Source: J Med Libr Assoc. 2018 Jul 1;106(3):352–60. doi: 10.5195/jmla.2018.256 (PMC6013145; doi:10.5195/jmla.2018.256)
Supplement: Appendix C [file jmla-106-352-s003.pdf]

LEVOTHYROXINE SODIUM- levothyroxine sodium tablet
Lannett Company, Inc.

Levothyroxine Sodium Tablets, USP

DESCRIPTION

Levothyroxine Sodium Tablets, USP contain synthetic crystalline L-3,3',5,5'-tetraiodothyronine sodium salt [levothyroxine (T_4) sodium]. Synthetic T_4 is identical to that produced in the human thyroid gland. Levothyroxine (T_4) sodium has an empirical formula of $C_{15}H_{10}I_4N\ NaO_4 \cdot H_2O$, molecular weight of 798.86 g/mol (anhydrous), and structural formula as shown:

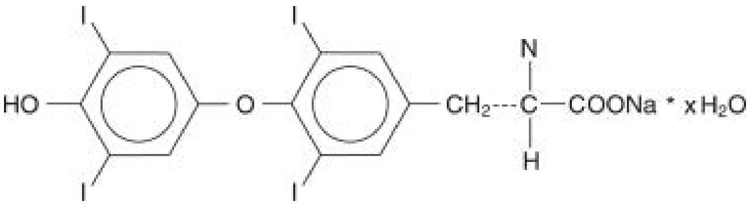

Inactive Ingredients

Colloidal silicon dioxide, lactose, magnesium stearate, microcrystalline cellulose, corn starch, acacia and sodium starch glycolate. The following are the coloring additives per tablet strength:

Strength (mcg)	Color Additive(s)
25	FD&C Yellow No. 6 Aluminum Lake
50	None
75	FD&C Red No. 40 Aluminum Lake, FD&C Blue No. 2 Aluminum Lake
88	D&C Yellow No. 10 Aluminum Lake, FD&C Yellow No. 6 Aluminum Lake, FD&C Blue No. 1 Aluminum Lake
100	D&C Yellow No. 10 Aluminum Lake, FD&C Yellow No. 6 Aluminum Lake
112	D&C Red No. 27 Aluminum Lake
125	FD&C Yellow No. 6 Aluminum Lake, FD&C Red No. 40 Aluminum Lake, FD&C Blue No. 1 Aluminum Lake
137	FD&C Blue No. 1 Aluminum Lake
150	FD&C Blue No. 2 Aluminum Lake
175	FD&C Blue No. 1 Aluminum Lake, D&C Red No. 27 Aluminum Lake
200	FD&C Red No. 40 Aluminum Lake
300	D&C Yellow No. 10 Aluminum Lake, FD&C Yellow No. 6 Aluminum Lake, FD&C Blue No. 1 Aluminum Lake

CLINICAL PHARMACOLOGY

Thyroid hormone synthesis and secretion is regulated by the hypothalamic-pituitary-thyroid axis. Thyrotropin-releasing hormone (TRH) released from the hypothalamus stimulates secretion of thyrotropin-stimulating hormone, TSH, from the anterior pituitary. TSH, in turn, is the physiologic stimulus for the synthesis and secretion of thyroid hormones, L-thyroxine (T_4) and L-triiodothyronine (T_3), by the thyroid gland. Circulating serum T_3 and T_4 levels exert a feedback effect on both TRH and

TSH secretion. When serum T_3 and T_4 levels increase, TRH and TSH secretion decrease. When thyroid hormone levels decrease, TRH and TSH secretion increase.

The mechanisms by which thyroid hormones exert their physiologic actions are not completely understood, but it is thought that their principal effects are exerted through control of DNA transcription and protein synthesis. T_3 and T_4 diffuse into the cell nucleus and bind to thyroid receptor proteins attached to DNA. This hormone nuclear receptor complex activates gene transcription and synthesis of messenger RNA and cytoplasmic proteins.

Thyroid hormones regulate multiple metabolic processes and play an essential role in normal growth and development, and normal maturation of the central nervous system and bone. The metabolic actions of thyroid hormones include augmentation of cellular respiration and thermogenesis, as well as metabolism of proteins, carbohydrates and lipids. The protein anabolic effects of thyroid hormones are essential to normal growth and development.

The physiologic actions of thyroid hormones are produced predominately by T_3, the majority of which (approximately 80%) is derived from T_4 by deiodination in peripheral tissues.

Levothyroxine, at doses individualized according to patient response, is effective as replacement or supplemental therapy in hypothyroidism of any etiology, except transient hypothyroidism during the recovery phase of subacute thyroiditis.

Levothyroxine is also effective in the suppression of pituitary TSH secretion in the treatment or prevention of various types of euthyroid goiters, including thyroid nodules, Hashimoto's thyroiditis, multinodular goiter and, as adjunctive therapy in the management of thyrotropin-dependent well-differentiated thyroid cancer (see **INDICATIONS AND USAGE, PRECAUTIONS, DOSAGE AND ADMINISTRATION**).

Pharmacokinetics

Absorption - Absorption of orally administered T_4 from the gastrointestinal (GI) tract ranges from 40% to 80%. The majority of the levothyroxine dose is absorbed from the jejunum and upper ileum. The relative bioavailability of Levothyroxine Sodium Tablets, USP, compared to an equal nominal dose of oral levothyroxine sodium solution, is approximately 99%. T_4 absorption is increased by fasting, and decreased in malabsorption syndromes and by certain foods such as soybean infant formula. Dietary fiber decreases bioavailability of T_4. Absorption may also decrease with age. In addition, many drugs and foods affect T_4 absorption (see **PRECAUTIONS, Drug Interactions** and **Drug-Food Interactions**).

Distribution - Circulating thyroid hormones are greater than 99% bound to plasma proteins, including thyroxine-binding globulin (TBG), thyroxine-binding prealbumin (TBPA), and albumin (TBA), whose capacities and affinities vary for each hormone. The higher affinity of both TBG and TBPA for T_4 partially explains the higher serum levels, slower metabolic clearance, and longer half-life of T_4 compared to T_3. Protein-bound thyroid hormones exist in reverse equilibrium with small amounts of free hormone. Only unbound hormone is metabolically active. Many drugs and physiologic conditions affect the binding of thyroid hormones to serum proteins (see **PRECAUTIONS, Drug Interactions** and **Drug-Laboratory Test Interactions**). Thyroid hormones do not readily cross the placental barrier (see **PRECAUTIONS, Pregnancy**).

Metabolism - T_4 is slowly eliminated (see **TABLE 1**). The major pathway of thyroid hormone metabolism is through sequential deiodination. Approximately eighty-percent of circulating T_3 is derived from peripheral T_4 by monodeiodination. The liver is the major site of degradation for both T_4 and T_3; with T_4 deiodination also occurring at a number of additional sites, including the kidney and other tissues. Approximately 80% of the daily dose of T_4 is deiodinated to yield equal amounts of T_3 and reverse T_3 (rT_3). T_3 and rT_3 are further deiodinated to diiodothyronine. Thyroid hormones are also metabolized via conjugation with glucuronides and sulfates and excreted directly into the bile and gut where they undergo enterohepatic recirculation.

Elimination - Thyroid hormones are primarily eliminated by the kidneys. A portion of the conjugated hormone reaches the colon unchanged and is eliminated in the feces. Approximately 20% of T_4 is eliminated in the stool. Urinary excretion of T_4 decreases with age.

Table 1: Pharmacokinetic Parameters of Thyroid Hormones in Euthyroid Patients

Hormone	Ratio in Thyroglobulin	Biologic Potency	$t_{1/2}$ (days)	Protein Binding (%)[2]
Levothyroxine (T_4)	10 - 20	1	6-7[1]	99.96
Liothyronine (T_3)	1	4	≤ 2	99.5
[1] 3 to 4 days in hyperthyroidism, 9 to 10 days in hypothyroidism; [2] Includes TBG, TBPA, and TBA				

INDICATIONS AND USAGE

Levothyroxine sodium is used for the following indications:

Hypothyroidism - As replacement or supplemental therapy in congenital or acquired hypothyroidism of any etiology, except transient hypothyroidism during the recovery phase of subacute thyroiditis. Specific indications include: primary (thyroidal), secondary (pituitary), and tertiary (hypothalamic) hypothyroidism and subclinical hypothyroidism. Primary hypothyroidism may result from functional deficiency, primary atrophy, partial or total congenital absence of the thyroid gland, or from the effects of surgery, radiation, or drugs, with or without the presence of goiter.

Pituitary TSH Suppression - In the treatment or prevention of various types of euthyroid goiters (see **WARNINGS** and **PRECAUTIONS**), including thyroid nodules (see **WARNINGS** and **PRECAUTIONS**), subacute or chronic Iymphocytic thyroiditis (Hashimoto's thyroiditis), multinodular goiter (see **WARNINGS** and **PRECAUTIONS**), and, as an adjunct to surgery and radioiodine therapy in the management of thyrotropin-dependent well-differentiated thyroid cancer.

CONTRAINDICATIONS

Levothyroxine is contraindicated in patients with untreated subclinical (suppressed serum TSH level with normal T_3 and T_4 levels) or overt thyrotoxicosis of any etiology and in patients with acute myocardial infarction. Levothyroxine is contraindicated in patients with uncorrected adrenal insufficiency since thyroid hormones may precipitate an acute adrenal crisis by increasing the metabolic clearance of glucocorticoids (see **PRECAUTIONS**). Levothyroxine Sodium Tablets, USP is contraindicated in patients with hypersensitivity to any of the inactive ingredients in Levothyroxine Sodium Tablets, USP. (See **DESCRIPTION, Inactive Ingredients**).

WARNINGS

WARNING: Thyroid hormones, including Levothyroxine Sodium Tablets, USP, either alone or with other therapeutic agents, should not be used for the treatment of obesity for weight loss. In euthyroid patients, doses within the range of daily hormonal requirements are ineffective for weight reduction. Larger doses may produce serious or even life threatening manifestations of toxicity, particularly when given in association with sympathomimetic amines such as those used for their anorectic effects.

Levothyroxine sodium should not be used in the treatment of male or female infertility unless this condition is associated with hypothyroidism.

In patients with nontoxic diffuse goiter or nodular thyroid disease, particularly the elderly or those with underlying cardiovascular disease, levothyroxine sodium therapy is contraindicated if the serum TSH level is already suppressed due to the risk of precipitating overt thyrotoxicosis (see **CONTRAINDICATIONS**). If the serum TSH level is not suppressed, Levothyroxine Sodium Tablets, USP should be used with caution in conjunction with careful monitoring of thyroid function for evidence of hyperthyroidism and clinical monitoring for potential associated adverse cardiovascular signs and symptoms of hyperthyroidism.

PRECAUTIONS

General

Levothyroxine has a narrow therapeutic index. Regardless of the indication for use, careful dosage titration is necessary to avoid the consequences of over- or under-treatment. These consequences include, among others, effects on growth and development, cardiovascular function, bone metabolism, reproductive function, cognitive function, emotional state, gastrointestinal function, and on glucose and lipid metabolism. Many drugs interact with levothyroxine sodium necessitating adjustments in dosing to maintain therapeutic response (see **Drug Interactions**).

Effects on bone mineral density - In women, long-term levothyroxine sodium therapy has been associated with increased bone resorption, thereby decreasing bone mineral density, especially in post-menopausal women on greater than replacement doses or in women who are receiving suppressive doses of levothyroxine sodium. The increased bone resorption may be associated with increased serum levels and urinary excretion of calcium and phosphorous, elevations in bone alkaline phosphatase and suppressed serum parathyroid hormone levels. Therefore, it is recommended that patients receiving levothyroxine sodium be given the minimum dose necessary to achieve the desired clinical and biochemical response.

Patients with underlying cardiovascular disease - Exercise caution when administering levothyroxine to patients with cardiovascular disorders and to the elderly in whom there is an increased risk of occult cardiac disease. In these patients, levothyroxine therapy should be initiated at lower doses than those recommended in younger individuals or in patients without cardiac disease (see **WARNINGS; PRECAUTIONS, Geriatric Use;** and **DOSAGE AND ADMINISTRATION**). If cardiac symptoms develop or worsen, the levothyroxine dose should be reduced or withheld for one week and then cautiously restarted at a lower dose. Overtreatment with levothyroxine sodium may have adverse cardiovascular effects such as an increase in heart rate, cardiac wall thickness, and cardiac contractility and may precipitate angina or arrhythmias. Patients with coronary artery disease who are receiving levothyroxine therapy should be monitored closely during surgical procedures, since the possibility of precipitating cardiac arrhythmias may be greater in those treated with levothyroxine. Concomitant administration of levothyroxine and sympathomimetic agents to patients with coronary artery disease may precipitate coronary insufficiency.

Patients with nontoxic diffuse goiter or nodular thyroid disease- Exercise caution when administering levothyroxine to patients with nontoxic diffuse goiter or nodular thyroid disease in order to prevent precipitation of thyrotoxicosis (see **WARNINGS**). If the serum TSH is already suppressed, levothyroxine sodium should not be administered (see **Contraindications**).

Associated endocrine disorders

<u>Hypothalamic/pituitary hormone deficiencies</u> - In patients with secondary or tertiary hypothyroidism, additional hypothalamic/pituitary hormone deficiencies should be considered, and, if diagnosed, treated (see **PRECAUTIONS, Autoimmune polyglandular syndrome** for adrenal insufficiency).

<u>Autoimmune polyglandular syndrome</u> - Occasionally, chronic autoimmune thyroiditis may occur in association with other autoimmune disorders such as adrenal insufficiency, pernicious anemia, and insulin-dependent diabetes mellitus. Patients with concomitant adrenal insufficiency should be treated

with replacement glucocorticoids prior to initiation of treatment with levothyroxine sodium. Failure to do so may precipitate an acute adrenal crisis when thyroid hormone therapy is initiated, due to increased metabolic clearance of glucocorticoids by thyroid hormone. Patients with diabetes mellitus may require upward adjustments of their antidiabetic therapeutic regimens when treated with levothyroxine (see **PRECAUTIONS, Drug Interactions**).

Other associated medical conditions

Infants with congenital hypothyroidism appear to be at increased risk for other congenital anomalies, with cardiovascular anomalies (pulmonary stenosis, atrial septal defect, and ventricular septal defect,) being the most common association.

Information for Patients

Patients should be informed of the following information to aid in the safe and effective use of Levothyroxine Sodium Tablets, USP:

1. Notify your physician if you are allergic to any foods or medicines, are pregnant or intend to become pregnant, are breast-feeding or are taking any other medications, including prescription and over-the-counter preparations.
2. Notify your physician of any other medical conditions you may have, particularly heart disease, diabetes, clotting disorders, and adrenal or pituitary gland problems. Your dose of medications used to control these other conditions may need to be adjusted while you are taking Levothyroxine Sodium Tablets, USP. If you have diabetes, monitor your blood and/or urinary glucose levels as directed by your physician and immediately report any changes to your physician. If you are taking anticoagulants (blood thinners), your clotting status should be checked frequently.
3. Use Levothyroxine Sodium Tablets, USP only as prescribed by your physician. Do not discontinue or change the amount you take or how often you take it, unless directed to do so by your physician.
4. The levothyroxine in Levothyroxine Sodium Tablets, USP is intended to replace a hormone that is normally produced by your thyroid gland. Generally, replacement therapy is to be taken for life, except in cases of transient hypothyroidism, which is usually associated with an inflammation of the thyroid gland (thyroiditis).
5. Take Levothyroxine Sodium Tablets, USP in the morning on an empty stomach, at least one-half hour to one hour before eating any food.
6. It may take several weeks before you notice an improvement in your symptoms.
7. Notify your physician if you experience any of the following symptoms: rapid or irregular heartbeat, chest pain, shortness of breath, leg cramps, headache, nervousness, irritability, sleeplessness, tremors, change in appetite, weight gain or loss, vomiting, diarrhea, excessive sweating, heat intolerance, fever, changes in menstrual periods, hives or skin rash, or any other unusual medical event.
8. Notify your physician if you become pregnant while taking Levothyroxine Sodium Tablets, USP. It is likely that your dose of Levothyroxine Sodium Tablets, USP will need to be increased while you are pregnant.
9. Notify your physician or dentist that you are taking Levothyroxine Sodium Tablets, USP prior to any surgery.
10. Partial hair loss may occur rarely during the first few months of Levothyroxine Sodium Tablets, USP therapy, but this is usually temporary.
11. Levothyroxine Sodium Tablets, USP should not be used as a primary or adjunctive therapy in a weight control program.
12. Keep Levothyroxine Sodium Tablets, USP out of the reach of children. Store Levothyroxine Sodium Tablets, USP away from heat, moisture, and light.
13. Agents such as iron and calcium supplements and antacids can decrease the absorption of levothyroxine sodium tablets. Therefore, levothyroxine sodium tablets should not be administered within 4 hrs of these agents.

Laboratory Tests

General

The diagnosis of hypothyroidism is confirmed by measuring TSH levels using a sensitive assay (second generation assay sensitivity ≤ 0.1 mIU/L or third generation assay sensitivity ≤ 0.01 mIU/L) and measurement of free-T_4.

The adequacy of therapy is determined by periodic assessment of appropriate laboratory tests and clinical evaluation. The choice of laboratory tests depends on various factors including the etiology of the underlying thyroid disease, the presence of concomitant medical conditions, including pregnancy, and the use of concomitant medications (see **PRECAUTIONS, Drug Interactions** and **Drug-Laboratory Test Interactions**). Persistent clinical and laboratory evidence of hypothyroidism despite an apparent adequate replacement dose of Levothyroxine Sodium Tablets, USP may be evidence of inadequate absorption, poor compliance, drug interactions, or decreased T_4 potency of the drug product.

Adults

In adult patients with primary (thyroidal) hypothyroidism, serum TSH levels (using a sensitive assay) alone may be used to monitor therapy. The frequency of TSH monitoring during levothyroxine dose titration depends on the clinical situation but it is generally recommended at 6-8 week intervals until normalization. For patients who have recently initiated levothyroxine therapy and whose serum TSH has normalized or in patients who have had their dosage of levothyroxine changed, the serum TSH concentration should be measured after 8-12 weeks. When the optimum replacement dose has been attained, clinical (physical examination) and biochemical monitoring may be performed every 6-12 months, depending on the clinical situation, and whenever there is a change in the patient's status. It is recommended that a physical examination and a serum TSH measurement be performed at least annually in patients receiving Levothyroxine Sodium Tablets, USP. (see **WARNINGS, PRECAUTIONS** and **DOSAGE AND ADMINISTRATION**).

Pediatrics

In patients with congenital hypothyroidism, the adequacy of replacement therapy should be assessed by measuring both serum TSH (using a sensitive assay) and total- or free-T_4. During the first three years of life, the serum total- or free-T_4 should be maintained at all times in the upper half of the normal range. While the aim of therapy is to also normalize the serum TSH level, this is not always possible in a small percentage of patients, particularly in the first few months of therapy. TSH may not normalize due to a resetting of the pituitary-thyroid feedback threshold as a result of *in utero* hypothyroidism. Failure of the serum T_4 to increase into the upper half of the normal range within 2 weeks of initiation of Levothyroxine Sodium Tablets, USP therapy and/or of the serum TSH to decrease below 20 mU/L within 4 weeks should alert the physician to the possibility that the child is not receiving adequate therapy. Careful inquiry should then be made regarding compliance, dose of medication administered, and method of administration prior to raising the dose of Levothyroxine Sodium Tablets, USP.

The recommended frequency of monitoring of TSH and total or free T_4 in children is as follows: at 2 and 4 weeks after the initiation of treatment; every 1-2 months during the first year of life; every 2-3 months between 1 and 3 years of age; and every 3 to 12 months thereafter until growth is completed. More frequent intervals of monitoring may be necessary if poor compliance is suspected or abnormal values are obtained. It is recommended that TSH and T_4 levels, and a physical examination, if indicated, be performed 2 weeks after any change in Levothyroxine Sodium Tablets, USP dosage. Routine clinical examination, including assessment of mental and physical growth and development, and bone maturation should be performed at regular intervals (see **PRECAUTIONS, Pediatric Use** and **DOSAGE AND ADMINISTRATION**).

Secondary (pituitary) and tertiary (hypothalamic) hypothyroidism

Adequacy of therapy should be assessed by measuring serum free-T_4 levels, which should be

maintained in the upper half of the normal range in these patients.

Drug Interactions

Many drugs affect thyroid hormone pharmacokinetics and metabolism (e.g., absorption, synthesis, secretion, catabolism, protein binding, and target tissue response) and may alter the therapeutic response to Levothyroxine Sodium Tablets, USP. In addition, thyroid hormones and thyroid status have varied effects on the pharmacokinetics and action of other drugs. A listing of drug-thyroidal axis interactions is contained in Table 2.

The list of drug-thyroidal axis interactions in Table 2 may not be comprehensive due to the introduction of new drugs that interact with the thyroidal axis or the discovery of previously unknown interactions. The prescriber should be aware of this fact and should consult appropriate reference sources (e.g., package inserts of newly approved drugs, medical literature) for additional information if a drug-drug interaction with levothyroxine is suspected.

Table 2: Drug-Thyroidal Axis Interactions

Drug or Drug Class	Effect
**Drugs that may reduce TSH secretion - the reduction is not sustained; therefore, hypothyroidism does not occur**	
Dopamine/Dopamine Agonists<br>Glucocorticoids<br>Octreotide	Use of these agents may result in a transient reduction in TSH secretion when administered at the following doses: dopamine (≥ 1 mcg/kg/min); Glucocorticoids (hydrocortisone ≥ 100 mg/day or equivalent); Octreotide (> 100 mcg/day).
**Drugs that alter thyroid hormone secretion**	
**Drugs that may decrease thyroid hormone secretion, which may result in hypothyroidism**	
Aminoglutethimide<br>Amiodarone<br>Iodide (including iodine-containing Radiographic contrast agents)<br>Lithium<br>Methimazole<br>Propylthioracil (PTU)<br>Sulfonamides<br>Tolbutamide	Long-term lithium therapy can result in goiter in up to 50% of patients, and either subclinical or overt hypothyroidism, each in up to 20% of patients. The fetus, neonate, elderly and euthyroid patients with underlying thyroid disease (e.g., Hashimoto's thyroiditis or with Grave's disease previously treated with radioiodine or surgery) are among those individuals who are particularly susceptible to iodine-induced hypothyroidism. Oral cholecystographic agents and amiodarone are slowly excreted, producing more prolonged hypothyroidism than parenterally administered iodinated contrast agents. Long-term amino-glu-tethimide therapy may minimally decrease T_4 and T_3 levels and increase TSH, although all values remain within normal limits in most patients.
**Drugs that may increase thyroid hormone secretion, which may result in hyperthyroidism**	
Amiodarone<br>Iodide (including iodine-containing Radiographic contrast agents)	Iodide and drugs that contain pharmacologic amounts of iodide may cause hyperthyroidism in euthyroid patients with Grave's disease previously treated with antithyroid drugs or in euthyroid patients with thyroid autonomy (e.g., multinodular goiter or hyper functioning thyroid adenoma). Hyperthyroidism may develop over several weeks and may persist for several months after therapy discontinuation. Amiodarone may induce hyperthyroidism by causing

	[illegible] thyroiditis.
**Drugs that may decrease T_4 absorption, which may result in hypothyroidism**	
Antacids<br>- Aluminum & Magnesium Hydroxides<br>- Simethicone<br>Bile Acid Sequestrants<br>- Cholestyramine<br>- Colestipol<br>Calcium Carbonate<br>Cation Exchange Resins<br>- Kayexalate<br>Ferrous Sulfate<br>Orlistat<br>Sucralfate	Concurrent use may reduce the efficacy of levothyroxine by binding and delaying or preventing absorption, potentially resulting in hypothyroidism. Calcium carbonate may form an insoluble chelate with levothyroxine, and ferrous sulfate likely forms a ferric-thyroxine complex. Administer levothyroxine at least 4 hours apart from these agents. Patients treated concomitantly with orlistat and levothyroxine should be monitored for changes in thyroid function.
**Drugs that may alter T_4 and T_3 serum transport - but FT_4 concentration remains normal; and, therefore, the patient remains euthyroid**	
**Drugs that may increase serum TBG concentration**	**Drugs that may decrease serum TBG concentration**
Clofibrate<br>Estrogen-containing oral contraceptives<br>Estrogens (oral)<br>Heroin / Methadone<br>5-Fluorouracil<br>Mitotane<br>Tamoxifen	Androgens / Anabolic Steroids<br>Asparaginase<br>Glucocorticoids<br>Slow-Release Nicotinic Acid
**Drugs that may cause protein-binding site displacement**	
Furosemide (> 80 mg IV)<br>Heparin<br>Hydantoins<br>Non Steroidal Anti-lnflammatory Drugs<br>- Fenamates<br>- Phenylbutazone<br>Salicylates (> 2 g/day)	Administration of these agents with levothyroxine results in an initial transient increase in FT_4. Continued administration results in a decrease in serum T_4 and normal FT_4 and TSH concentrations and, therefore, patients are clinically euthyroid. Salicylates inhibit binding of T_4 and T_3 to TBG and transthyretin. An initial increase in serum FT_4, is followed by return of FT_4 to normal levels with sustained therapeutic serum salicylate concentrations, although total-T_4 levels may decrease by as much as 30%.
**Drugs that may alter T_4 and T_3 metabolism**	
**Drugs that may increase hepatic metabolism, which may result in hypothyroidism**	
Carbamazepine<br>Hydantoins<br>Phenobarbital<br>Rifampin	Stimulation of hepatic microsomal drug-metabolizing enzyme activity may cause increased hepatic degradation of levothyroxine, resulting in increased Ievothyroxine requirements. Phenytoin and carbamazepine reduce serum protein binding of levothyroxine, and total- and free-T_4 may be reduced by 20% to 40%, but most patients have normal serum TSH levels and are clinically euthyroid.

**Drugs that may decrease T_4 5' - deiodinase activity**	
Amiodarone<br>Beta-adrenergic antagonists<br>- (e.g., Propranolol > 160 mg/day)<br>Glucocorticoids<br>-(e.g., Dexamethasone ≥ 4 mg/day)<br>Propylthiouracil (PTU)	Administration of these enzyme inhibitors decrease the peripheral conversion of T_4 to T_3, leading to decreased T_3 levels. However, serum T_4 levels are usually normal but may occasionally be slightly increased. In patients treated with large doses of propranolol (> 160 mg/day), T_3 and T_4 levels change slightly, TSH levels remain normal, and patients are clinically euthyroid. It should be noted that actions of particular beta-adrenergic antagonists may be impaired when the hypothyroid patient is converted to the euthyroid state. Short-term administration of large doses of glucocorticoids may decrease serum T_3 concentrations by 30% with minimal change in serum T_4 levels. However, long-term glucocorticoid therapy may result in slightly decreased T_3 and T_4 levels due to decreased TBG production (see above).
**Miscellaneous**	
Anticoagulants (oral)<br>- Coumarin Derivatives<br>- Indandione Derivatives	Thyroid hormones appear to increase the catabolism of vitamin K-dependent clotting factors, thereby increasing the anticoagulant activity of oral anticoagulants. Concomitant use of these agents impairs the compensatory increases in clotting factor synthesis. Prothrombin time should be carefully monitored in patients taking levothyroxine and oral anticoagulants and the dose of anticoagulant therapy adjusted accordingly.
Antidepressants<br>- Tricyclics (e.g., Amitriptyline)<br>- Tetracyclics (e.g., Maprotiline)<br>- Selective Serotonin Reuptake Inhibitors (SSRIs; e.g., Sertraline)	Concurrent use of tri/tetracyclic antidepressants and levothyroxine may increase the therapeutic and toxic effects of both drugs, possibly due to increased receptor sensitivity to catecholamines.Toxic effects may include increased risk of cardiac arrhythmias and CNS stimulation; onset of action of tricyclics may be accelerated. Administration of sertraline in patients stabilized on levothyroxine may result in increased levothyroxine requirements.
Antidiabetic Agents<br>- Biguanides<br>- Meglitinides<br>- Sulfonylureas<br>- Thiazolidediones<br>- Insulin	Addition of levothyroxine to antidiabetic or insulin therapy may result in increased antidiabetic agent or insulin requirements. Careful monitoring of diabetic control is recommended, especially when thyroid therapy is started, changed, or discontinued.
Cardiac Glycosides	Serum digitalis glycoside levels may be reduced in hyperthyroidism or when the hypothyroid patient is converted to the euthyroid state. Therapeutic effect of digitalis glycosides may be reduced.
Cytokines<br>- Interferon-α<br>- Interleukin-2	Therapy with interferon-α has been associated with the development of antithyroid microsomal antibodies in 20% of patients and some have transient hypothyroidism, hyperthyroidism, or both. Patients who have antithyroid antibodies before treatment are at higher risk for thyroid dysfunction during treatment. Interleukin-2 has been associated with transient painless thyroiditis in 20% of patients. Interferon-β and -γ have not been reported to cause thyroid dysfunction.

Growth Hormones<br>- Somatrem<br>- Somatropin	Excessive use of thyroid hormones with growth hormones may accelerate epiphyseal closure. However, untreated hypothyroidism may interfere with growth response to growth hormone.
Ketamine	Concurrent use may produce marked hypertension and tachycardia; cautious administration to patients receiving thyroid hormone therapy is recommended.
Methylxanthine Bronchodilators<br>- (e.g., Theophylline)	Decreased theophylline clearance may occur in hypothyroid patients; clearance returns to normal when the euthyroid state is achieved.
Radiographic Agents	Thyroid hormones may reduce the uptake of ^{123}I, ^{131}I, and ^{99m}Tc.
Sympathomimetics	Concurrent use may increase the effects of sympathomimetics or thyroid hormone. Thyroid hormones may increase the risk of coronary insufficiency when sympathomimetic agents are administered to patients with coronary artery disease.
Chloral Hydrate<br>Diazepam<br>Ethionamide<br>Lovastatin<br>Metoclopramide<br>6-Mercaptopurine<br>Nitroprusside<br>Para-aminosalicylate sodium<br>Perphenazine<br>Resorcinol (excessive topical use)<br>Thiazide Diuretics	These agents have been associated with thyroid hormone and/or TSH level alterations by various mechanisms.

Oral anticoagulants - Levothyroxine increases the response to oral anticoagulant therapy. Therefore, a decrease in the dose of anticoagulant may be warranted with correction of the hypothyroid state or when the Levothyroxine Sodium Tablets, USP dose is increased. Prothrombin time should be closely monitored to permit appropriate and timely dosage adjustments (see **Table 2**).

Digitalis glycosides - The therapeutic effects of digitalis glycosides may be reduced by levothyroxine. Serum digitalis glycoside levels may be decreased when a hypothyroid patient becomes euthyroid, necessitating an increase in the dose of digitalis glycosides (see **Table 2**).

Drug-Food Interactions

Consumption of certain foods may affect levothyroxine absorption thereby necessitating adjustments in dosing. Soybean flour (infant formula), cotton seed meal, walnuts, and dietary fiber may bind and decrease the absorption of levothyroxine sodium from the GI tract.

Drug-Laboratory Test Interactions

Changes in TBG concentration must be considered when interpreting T_4 and T_3 values, which necessitates measurement and evaluation of unbound (free) hormone and/or determination of the free T_4 index (FT_4I). Pregnancy, infectious hepatitis, estrogens, estrogen-containing oral contraceptives, and acute intermittent porphyria increase TBG concentrations. Decreases in TBG concentrations are observed in nephrosis, severe hypoproteinemia, severe liver disease, acromegaly, and after androgen

or corticosteroid therapy (see also **Table 2**). Familial hyper- or hypo-thyroxine binding globulinemias have been described, with the incidence of TBG deficiency approximating 1 in 9000.

Carcinogenesis, Mutagenesis, and Impairment of Fertility

Animal studies have not been performed to evaluate the carcinogenic potential, mutagenic potential or effects on fertility of levothyroxine. The synthetic T_4 in Levothyroxine Sodium Tablets, USP is identical to that produced naturally by the human thyroid gland. Although there has been a reported association between prolonged thyroid hormone therapy and breast cancer, this has not been confirmed. Patients receiving Levothyroxine Sodium Tablets, USP for appropriate clinical indications should be titrated to the lowest effective replacement dose.

Pregnancy - Category A

Studies in women taking levothyroxine sodium during pregnancy have not shown an increased risk of congenital abnormalities. Therefore, the possibility of fetal harm appears remote. Levothyroxine Sodium Tablets, USP should not be discontinued during pregnancy and hypothyroidism diagnosed during pregnancy should be promptly treated.

Hypothyroidism during pregnancy is associated with a higher rate of complications, including spontaneous abortion, pre-eclampsia, stillbirth and premature delivery. Maternal hypothyroidism may have an adverse effect on fetal and childhood growth and development. During pregnancy, serum T_4 levels may decrease and serum TSH levels increase to values outside the normal range. Since elevations in serum TSH may occur as early as 4 weeks gestation, pregnant women taking Levothyroxine Sodium Tablets, USP should have their TSH measured during each trimester. An elevated serum TSH level should be corrected by an increase in the dose of Levothyroxine Sodium Tablets, USP. Since postpartum TSH levels are similar to preconception values, the Levothyroxine Sodium Tablets, USP dosage should return to the pre-pregnancy dose immediately after delivery. A serum TSH level should be obtained 6-8 weeks postpartum.

Thyroid hormones cross the placental barrier to some extent as evidenced by levels in cord blood of athyroceotic fetuses being approximately one third maternal levels. Transfer of thyroid hormone from the mother to the fetus, however, may not be adequate to prevent *in utero,* hypothyroidism.

Nursing Mothers

Although thyroid hormones are excreted only minimally in human milk, caution should be exercised when Levothyroxine Sodium Tablets, USP is administered to a nursing woman. However, adequate replacement doses of levothyroxine are generally needed to maintain normal lactation.

Pediatric Use

General

The goal of treatment in pediatric patients with hypothyroidism is to achieve and maintain normal intellectual and physical growth and development.

The initial dose of levothyroxine varies with age and body weight (see **DOSAGE AND ADMINISTRATION**, **Table 3**). Dosing adjustments are based on an assessment of the individual patient's clinical and laboratory parameters (see **PRECAUTIONS, Laboratory Tests**).

In children in whom a diagnosis of permanent hypothyroidism has not been established, it is recommended that levothyroxine administration be discontinued for a 30-day trial period, but only after the child is at least 3 years of age. Serum T_4 and TSH levels should then be obtained. If the T_4 is low and the TSH high, the diagnosis of permanent hypothyroidism is established, and levothyroxine therapy should be reinstituted. If the T_4 and TSH levels are normal, euthyroidism may be assumed and, therefore, the hypothyroidism can be considered to have been transient. In this instance, however, the physician should carefully monitor the child and repeat the thyroid function tests if any signs or

symptoms of hypothyroidism develop. In this setting, the clinician should have a high index of suspicion of relapse. If the results of the levothyroxine withdrawal test are inconclusive, careful follow-up and subsequent testing will be necessary.

Since some more severely affected children may become clinically hypothyroid when treatment is discontinued for 30 days, an alternate approach is to reduce the replacement dose of levothyroxine by half during the 30-day trial period. If, after 30 days, the serum TSH is elevated above 20 mU/L, the diagnosis of permanent hypothyroidism is confirmed, and full replacement therapy should be resumed. However, if the serum TSH has not risen to greater than 20 mU/L, levothyroxine treatment should be discontinued for another 30-day trial period followed by repeat serum T_4 and TSH.

The presence of concomitant medical conditions should be considered in certain clinical circumstances and, if present, appropriately treated (see **PRECAUTIONS**).

Congenital Hypothyroidism (see **PRECAUTIONS, Laboratory Tests** and **DOSAGE AND ADMINISTRATION**)

Rapid restoration of normal serum T_4 concentrations is essential for preventing the adverse effects of congenital hypothyroidism on intellectual development as well as on overall physical growth and maturation. Therefore, Levothyroxine Sodium Tablets, USP therapy should be initiated immediately upon diagnosis and is generally continued for life.

During the first 2 weeks of Levothyroxine Sodium Tablets, USP therapy, infants should be closely monitored for cardiac overload, arrhythmias, and aspiration from avid suckling.

The patient should be monitored closely to avoid undertreatment or overtreatment. Undertreatment may have deleterious effects on intellectual development and linear growth. Overtreatment has been associated with craniosynostosis in infants, and may adversely affect the tempo of brain maturation and accelerate the bone age with resultant premature closure of the epiphyses and compromised adult stature.

Acquired Hypothyroidism in Pediatric Patients

The patient should be monitored closely to avoid undertreatment and overtreatment. Undertreatment may result in poor school performance due to impaired concentration and slowed mentation and in reduced adult height. Overtreatment may accelerate the bone age and result in premature epiphyseal closure and compromised adult stature.

Treated children may manifest a period of catch-up growth, which may be adequate in some cases to normalize adult height. In children with severe or prolonged hypothyroidism, catch-up growth may not be adequate to normalize adult height.

Geriatric Use

Because of the increased prevalence of cardiovascular disease among the elderly, levothyroxine therapy should not be initiated at the full replacement dose (see **WARNINGS, PRECAUTIONS** and **DOSAGE AND ADMINISTRATION**).

ADVERSE REACTIONS

Adverse reactions associated with levothyroxine therapy are primarily those of hyperthyroidism due to therapeutic overdosage (see **PRECAUTIONS** and **OVERDOSAGE**). They include the following:

General: fatigue, increased appetite, weight loss, heat intolerance, fever, excessive sweating;

Central nervous system: headache, hyperactivity, nervousness, anxiety, irritability, emotional lability, insomnia;

Musculoskeletal: tremors, muscle weakness;

Cardiovascular: palpitations, tachycardia, arrhythmias, increased pulse and blood pressure, heart failure,

angina, myocardial infarction, cardiac arrest;

Respiratory: dyspnea;

Gastrointestinal: diarrhea, vomiting, abdominal cramps and elevation in liver function tests;

Dermatologic: hair loss; flushing;

Endocrine: decreased bone mineral density;

Reproductive: menstrual irregularities, impaired fertility.

Pseudotumor cerebri and slipped capital femoral epiphysis have been reported in children receiving levothyroxine therapy. Overtreatment may result in craniosynostosis in infants and premature closure of the epiphyses in children with resultant compromised height.

Seizures have been reported rarely with the institution of levothyroxine therapy.

Inadequate levothyroxine dosage will produce or fail to ameliorate the signs and symptoms of hypothyroidism.

Hypersensitivity reactions to inactive ingredients have occurred in patients treated with thyroid hormone products. These include urticaria, pruritus, skin rash, flushing, angioedema, various Gl symptoms (abdominal pain, nausea, vomiting and diarrhea), fever, arthralgia, serum sickness and wheezing. Hypersensitivity to levothyroxine itself is not known to occur.

OVERDOSAGE

The signs and symptoms of overdosage are those of hyperthyroidism (see **PRECAUTIONS** and **ADVERSE REACTIONS**). In addition, confusion and disorientation may occur. Cerebral embolism, shock, coma, and death have been reported. Seizures have occurred in a child ingesting 18 mg of levothyroxine. Symptoms may not necessarily be evident or may not appear until several days after ingestion of levothyroxine sodium.

Treatment of Overdosage

Levothyroxine sodium should be reduced in dose or temporarily discontinued if signs or symptoms of overdosage occur.

Acute Massive Overdosage - This may be a life-threatening emergency, therefore, symptomatic and supportive therapy should be instituted immediately. If not contraindicated (e.g., by seizures, coma, or loss of the gag reflex), the stomach should be emptied by emesis or gastric lavage to decrease gastrointestinal absorption. Activated charcoal or cholestyramine may also be used to decrease absorption. Central and peripheral increased sympathetic activity may be treated by administering β-receptor antagonists, e.g., propranolol, provided there are no medical contraindications to their use. Provide respiratory support as needed; control congestive heart failure and arrhythmia; control fever, hypoglycemia, and fluid loss as necessary. Large doses of antithyroid drugs (e.g., methimazole or propylthiouracil) followed in one to two hours by large doses of iodine may be given to inhibit synthesis and release of thyroid hormones. Glucocorticoids may be given to inhibit the conversion of T_4 to T_3. Plasmapheresis, charcoal hemoperfusion and exchange transfusion have been reserved for cases in which continued clinical deterioration occurs despite conventional therapy. Because T_4 is highly protein bound, very little drug will be removed by dialysis.

DOSAGE AND ADMINISTRATION

General Principles:

The goal of replacement therapy is to achieve and maintain a clinical and biochemical euthyroid state. The goal of suppressive therapy is to inhibit growth and/or function of abnormal thyroid tissue. The dose of Levothyroxine Sodium Tablets, USP that is adequate to achieve these goals depends on a

variety of factors including the patient's age, body weight, cardiovascular status, concomitant medical conditions, including pregnancy, concomitant medications, and the specific nature of the condition being treated (see **WARNINGS** and **PRECAUTIONS**). Hence, the following recommendations serve only as dosing guidelines. Dosing must be individualized and adjustments made based on periodic assessment of the patient's clinical response and laboratory parameters (see **PRECAUTIONS, Laboratory Tests**).

Levothyroxine Sodium Tablets, USP should be taken in the morning on an empty stomach, at least one-half hour to one hour before any food is eaten. Levothyroxine Sodium Tablets, USP should be taken at least 4 hours apart from drugs that are known to interfere with its absorption (see **PRECAUTIONS, Drug Interactions**).

Due to the long half-life of levothyroxine, the peak therapeutic effect at a given dose of levothyroxine sodium may not be attained for 4-6 weeks.

Caution should be exercised when administering Levothyroxine Sodium Tablets, USP to patients with underlying cardiovascular disease, to the elderly, and to those with concomitant adrenal insufficiency (see **PRECAUTIONS**).

Specific Patient Populations:

Hypothyroidism in Adults and in Children in Whom Growth and Puberty are Complete (see **WARNINGS** and **PRECAUTIONS, Laboratory Tests**).

Therapy may begin at full replacement doses in otherwise healthy individuals less than 50 years old and in those older than 50 years who have been recently treated for hyperthyroidism or who have been hypothyroid for only a short time (such as a few months). The average full replacement dose of levothyroxine sodium is approximately 1.7 mcg/kg/day (e.g., **100-125 mcg/day** for a 70 kg adult). Older patients may require less than 1 mcg/kg/day. Levothyroxine sodium doses greater than 200 mcg/day are seldom required. An inadequate response to daily doses ≥ 300 mcg/day is rare and may indicate poor compliance, malabsorption, and/or drug interactions.

For most patients older than 50 years or for patients under 50 years of age with underlying cardiac disease, an initial starting dose of **25-50 mcg/day** of levothyroxine sodium is recommended, with gradual increments in dose at 6-8 week intervals, as needed. The recommended starting dose of levothyroxine sodium in elderly patients with cardiac disease is **12.5-25 mcg/day**, with gradual dose increments at 4-6 week intervals. The levothyroxine sodium dose is generally adjusted in 12.5-25 mcg increments until the patient with primary hypothyroidism is clinically euthyroid and the serum TSH has normalized.

In patients with severe hypothyroidism, the recommended initial levothyroxine sodium dose is **12.5-25 mcg/day** with increases of 25 mcg/day every 2-4 weeks, accompanied by clinical and laboratory assessment, until the TSH level is normalized.

In patients with secondary (pituitary) or tertiary (hypothalamic) hypothyroidism, the levothyroxine sodium dose should be titrated until the patient is clinically euthyroid and the serum free-T_4 level is restored to the upper half of the normal range.

Pediatric Dosage - Congenital or Acquired Hypothyroidism (see **PRECAUTIONS, Laboratory Tests**)

General Principles

In general, levothyroxine therapy should be instituted at full replacement doses as soon as possible. Delays in diagnosis and institution of therapy may have deleterious effects on the child's intellectual and physical growth and development.

Undertreatment and overtreatment should be avoided (see **PRECAUTIONS, Pediatric Use**).

Levothyroxine Sodium Tablets, USP may be administered to infants and children who cannot swallow intact tablets by crushing the tablet and suspending the freshly crushed tablet in a small amount (5-10 mL or 1-2 teaspoons) of water. This suspension can be administered by spoon or dropper. **DO NOT**

STORE THE SUSPENSION. Foods that decrease absorption of levothyroxine, such as soybean infant formula, should not be used for administering levothyroxine sodium tablets. (see **PRECAUTIONS, Drug-Food Interactions**).

Newborns

The recommended starting dose of levothyroxine sodium in newborn infants is **10-15 mcg/kg/day**. A lower starting dose (e.g., 25 mcg/day) should be considered in infants at risk for cardiac failure, and the dose should be increased in 4-6 weeks as needed based on clinical and laboratory response to treatment. In infants with very low (< 5 mcg/dL) or undetectable serum T_4 concentrations, the recommended initial starting dose is **50 mcg/day** of levothyroxine sodium.

Infants and Children

Levothyroxine therapy is usually initiated at full replacement doses, with the recommended dose per body weight decreasing with age (see **TABLE 3**). However, in children with chronic or severe hypothyroidism, an initial dose of **25 mcg/day** of levothyroxine sodium is recommended with increments of 25 mcg every 2-4 weeks until the desired effect is achieved.

Hyperactivity in an older child can be minimized if the starting dose is one-fourth of the recommended full replacement dose, and the dose is then increased on a weekly basis by an amount equal to one-fourth the full-recommended replacement dose until the full recommended replacement dose is reached.

Table 3: Levothyroxine Sodium Dosing Guidelines for Pediatric Hypothyroidism

AGE	Daily Dose Per Kg Body Weight[a]
**0-3 months**	**10-15 mcg/kg/day**
**3-6 months**	**8-10 mcg/kg/day**
**6-12 months**	**6-8 mcg/kg/day**
**1-5 years**	**5-6 mcg/kg/day**
**6-12 years**	**4-5 mcg/kg/day**
**>12 years but growth and puberty incomplete**	**2-3 mcg/kg/day**
**Growth and puberty complete**	**1.7 mcg/kg/day**
a. The dose should be adjusted based on clinical response and laboratory parameters (see **PRECAUTlONS, Laboratory Tests** and **Pediatric Use**).	

Pregnancy- Pregnancy may increase levothyroxine requirements (see **PREGNANCY**).

Subclinical Hypothyroidism- If this condition is treated, a lower levothyroxine sodium dose (e.g., **1 mcg/kg/day**) than that used for full replacement may be adequate to normalize the serum TSH level. Patients who are not treated should be monitored yearly for changes in clinical status and thyroid laboratory parameters.

TSH Suppression in Well-differentiated Thyroid Cancer and Thyroid Nodules- The target level for TSH suppression in these conditions has not been established with controlled studies. In addition, the efficacy of TSH suppression for benign nodular disease is controversial. Therefore, the dose of Levothyroxine Sodium Tablets, USP used for TSH suppression should be individualized based on the specific disease and the patient being treated.

In the treatment of well differentiated (papillary and follicular) thyroid cancer, levothyroxine is used as an adjunct to surgery and radioiodine therapy. Generally, TSH is suppressed to <0.1 mU/L, and this usually requires a levothyroxine sodium dose of **greater than 2 mcg/kg/day**. However, in patients with high-risk tumors, the target level for TSH suppression may be <0.01 mU/L.

In the treatment of benign nodules and nontoxic multinodular goiter, TSH is generally suppressed to a higher target (e.g., 0.1-0.5 mU/L for nodules and 0.5-1.0 mU/L for multinodular goiter) than that used for the treatment of thyroid cancer. Levothyroxine sodium is contraindicated if the serum TSH is

already suppressed due to the risk of precipitating overt thyrotoxicosis (see **CONTRAINDICATIONS, WARNINGS** and **PRECAUTIONS**).

Myxedema Coma - Myxedema coma is a life-threatening emergency characterized by poor circulation and hypometabolism, and may result in unpredictable absorption of levothyroxine sodium from the gastrointestinal tract. Therefore, oral thyroid hormone drug products are not recommended to treat this condition. Thyroid hormone products formulated for intravenous administration should be administered.

HOW SUPPLIED

Levothyroxine Sodium Tablets, USP are round, color coded, partial bisected tablets debossed with JSP and ID Number:

Strength (mcg)	Color	NDC# for bottles of 100	NDC# for bottles of 1000
25	Peach	NDC 0527-1341-01	NDC 0527-1341 -10
50	White	NDC 0527-1342-01	NDC 0527-1342 -10
75	Purple	NDC 0527-1343-01	NDC 0527-1343 -10
88	Olive	NDC 0527-1344-01	NDC 0527-1344 -10
100	Yellow	NDC 0527-1345-01	NDC 0527-1345 -10
112	Rose	NDC 0527-1346-01	NDC 0527-1346 -10
125	Tan	NDC 0527-1347-01	NDC 0527-1347 -10
137	Blue	NDC 0527-1638-01	NDC 0527-1638 -10
150	Lt. Blue	NDC 0527-1349-01	NDC 0527-1349 -10
175	Lilac	NDC 0527-1350-01	NDC 0527-1350 -10
200	Pink	NDC 0527-1351-01	NDC 0527-1351 -10
300	Green	NDC 0527-1352-01	NDC 0527-1352 -10

STORAGE CONDITIONS

20°C to 25°C (68°F to 77°F) with excursions between 15°C to 30°C (59°F to 86°F)

Rx only

Manufactured for:
Lannett Company, Inc.
Philadelphia, PA 19136

Manufactured by:
Jerome Stevens Pharmaceuticals, Inc.
Bohemia, NY 11716

Rev. 10/07

MG #18326

PRINCIPAL DISPLAY PANEL - 25 mcg (0.025 mg)

NDC 0527-1341-01

Lannett

LEVOTHYROXINE
SODIUM
TABLETS, USP

25 mcg (0.025 mg)

Rx ONLY

100 TABLETS

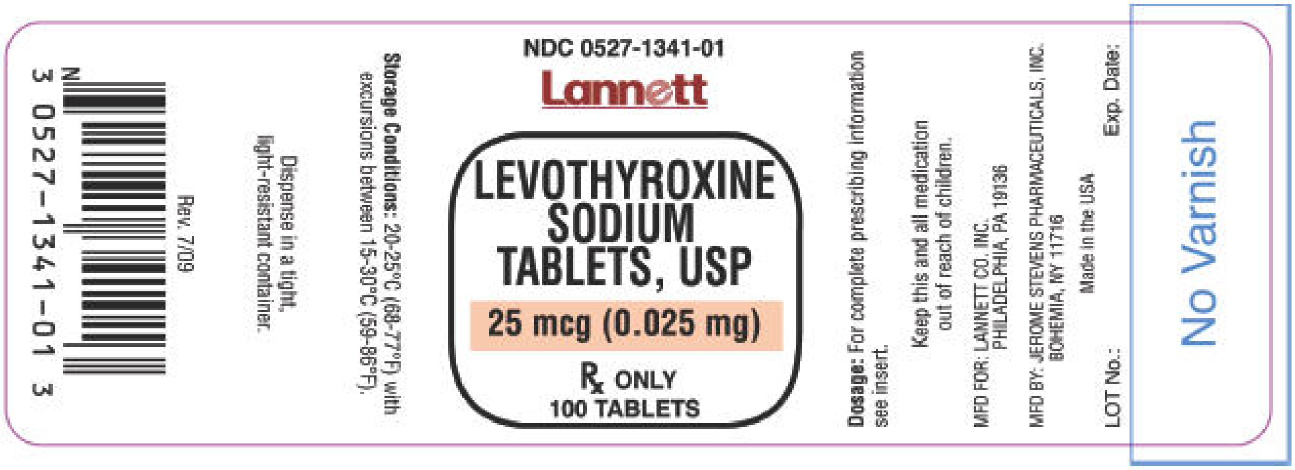

PRINCIPAL DISPLAY PANEL - 50 mcg (0.05 mg)

NDC 0527-1342-01

Lannett

**LEVOTHYROXINE
SODIUM
TABLETS, USP**

50 mcg (0.05 mg)

Rx ONLY

100 TABLETS

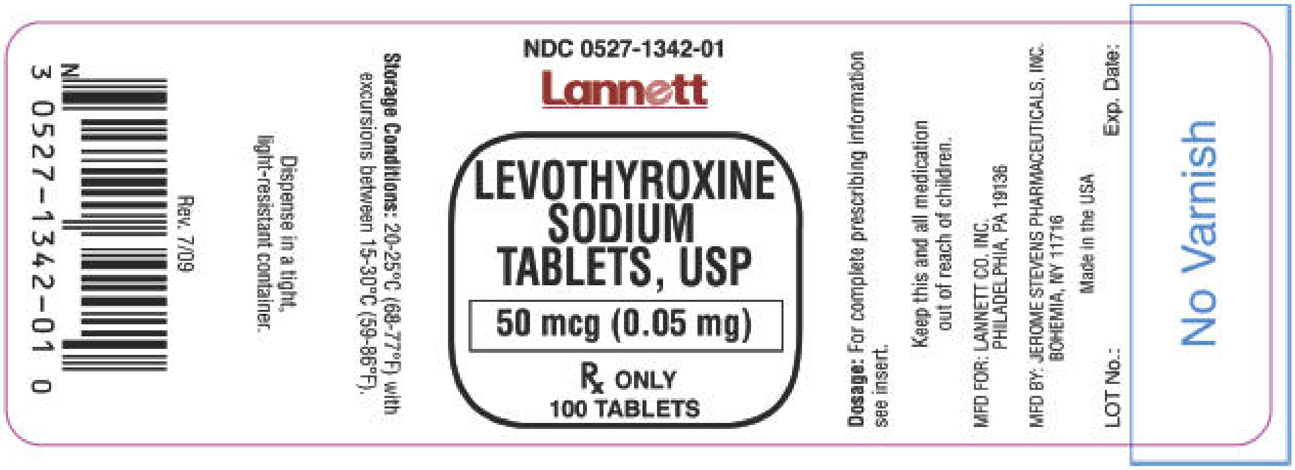

PRINCIPAL DISPLAY PANEL - 75 mcg (0.075 mg)

NDC 0527-1343-01

Lannett

**LEVOTHYROXINE
SODIUM
TABLETS, USP**

75 mcg (0.075 mg)

Rx ONLY

100 TABLETS

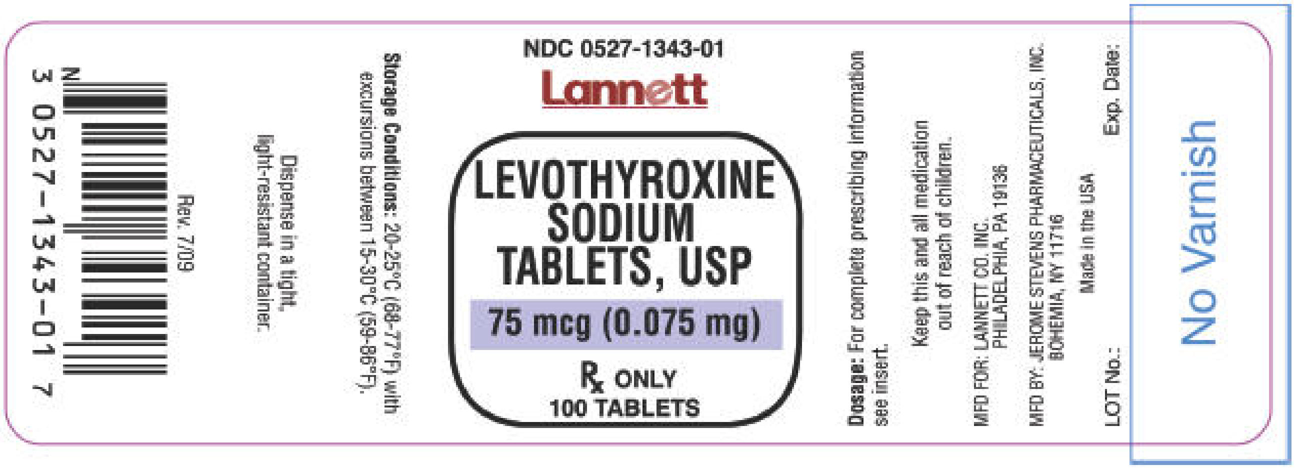

PRINCIPAL DISPLAY PANEL - 88 mcg (0.088 mg)

NDC 0527-1344-01

Lannett

LEVOTHYROXINE
SODIUM
TABLETS, USP

88 mcg (0.088 mg)

Rx ONLY

100 TABLETS

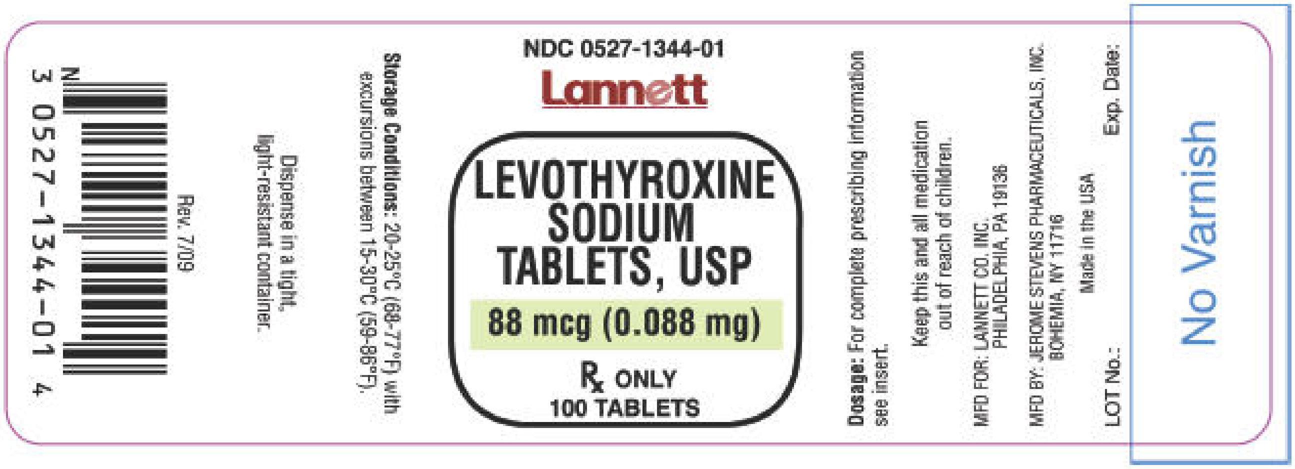

PRINCIPAL DISPLAY PANEL - 100 mcg (0.1 mg)

NDC 0527-1345-01

Lannett

LEVOTHYROXINE

SODIUM
TABLETS, USP

100 mcg (0.1 mg)

Rx ONLY

100 TABLETS

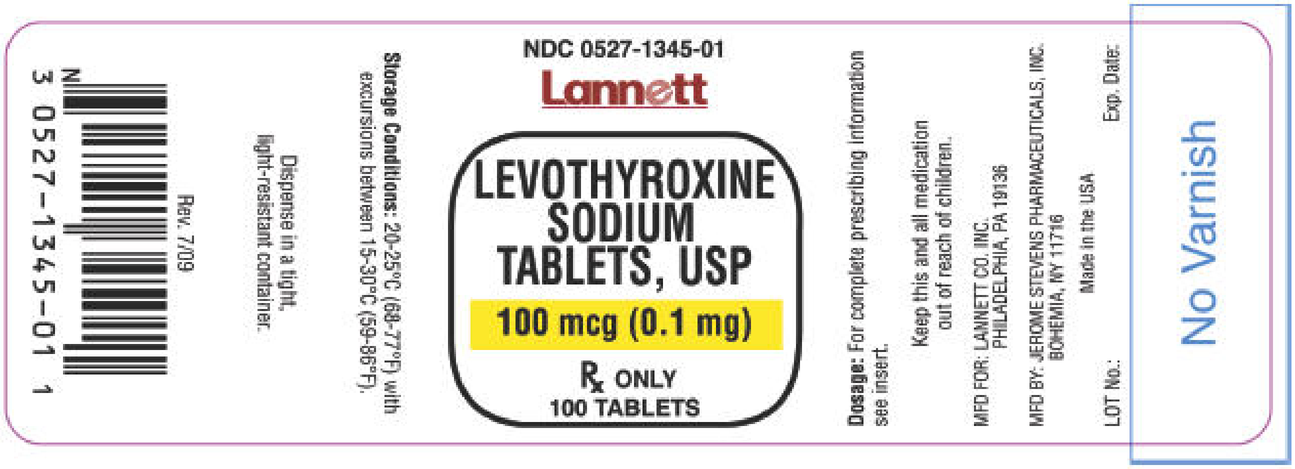

PRINCIPAL DISPLAY PANEL - 112 mcg (0.112 mg)

NDC 0527-1346-01

Lannett

LEVOTHYROXINE
SODIUM
TABLETS, USP

112 mcg (0.112 mg)

Rx ONLY

100 TABLETS

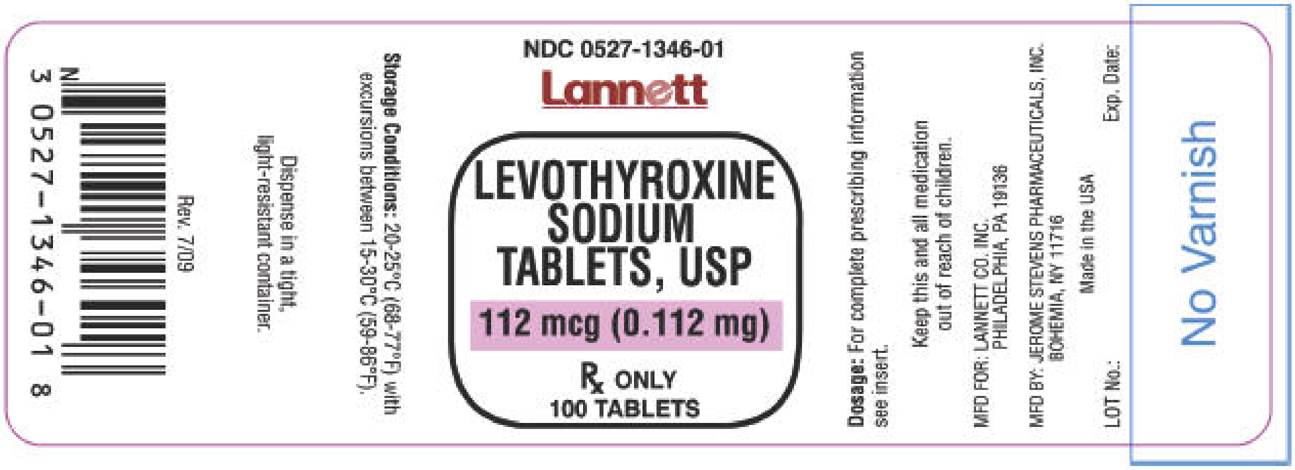

PRINCIPAL DISPLAY PANEL - 125 mcg (0.125 mg)

NDC 0527-1347-01

Lannett

LEVOTHYROXINE
SODIUM
TABLETS, USP

125 mcg (0.125 mg)

Rx ONLY

100 TABLETS

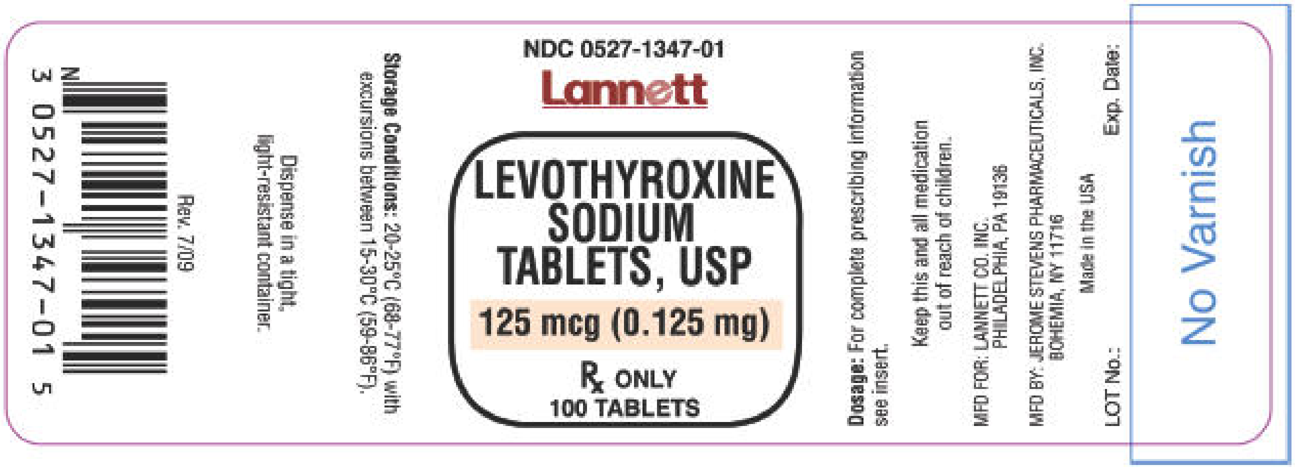

PRINCIPAL DISPLAY PANEL - 137 mcg (0.137 mg)

NDC 0527-1638-01

Lannett

LEVOTHYROXINE
SODIUM
TABLETS, USP

137 mcg (0.137 mg)

Rx ONLY

100 TABLETS

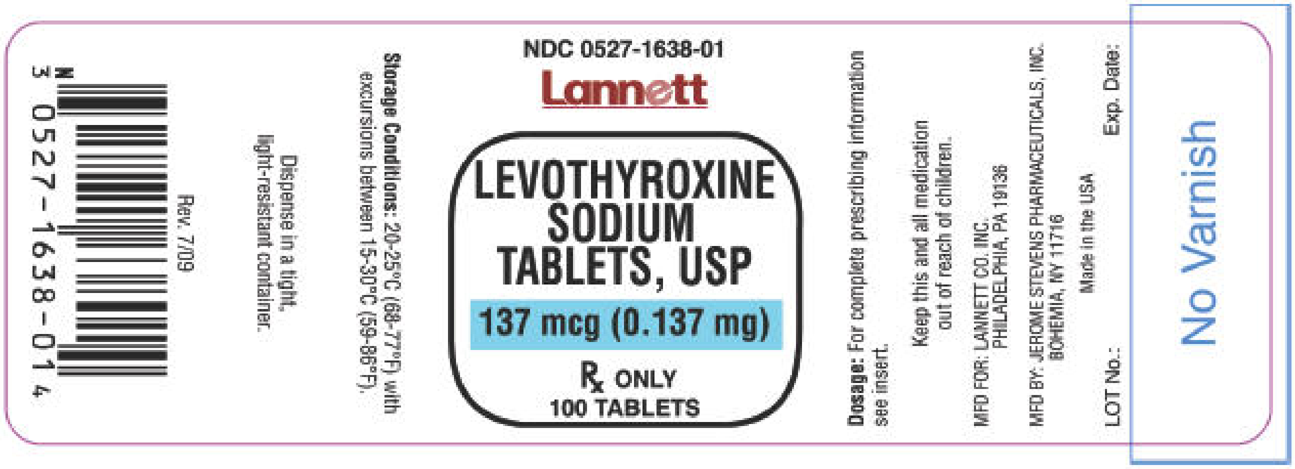

PRINCIPAL DISPLAY PANEL - 150 mcg (0.15 mg)

NDC 0527-1349-01

Lannett

LEVOTHYROXINE
SODIUM
TABLETS, USP

150 mcg (0.15 mg)

Rx ONLY

100 TABLETS

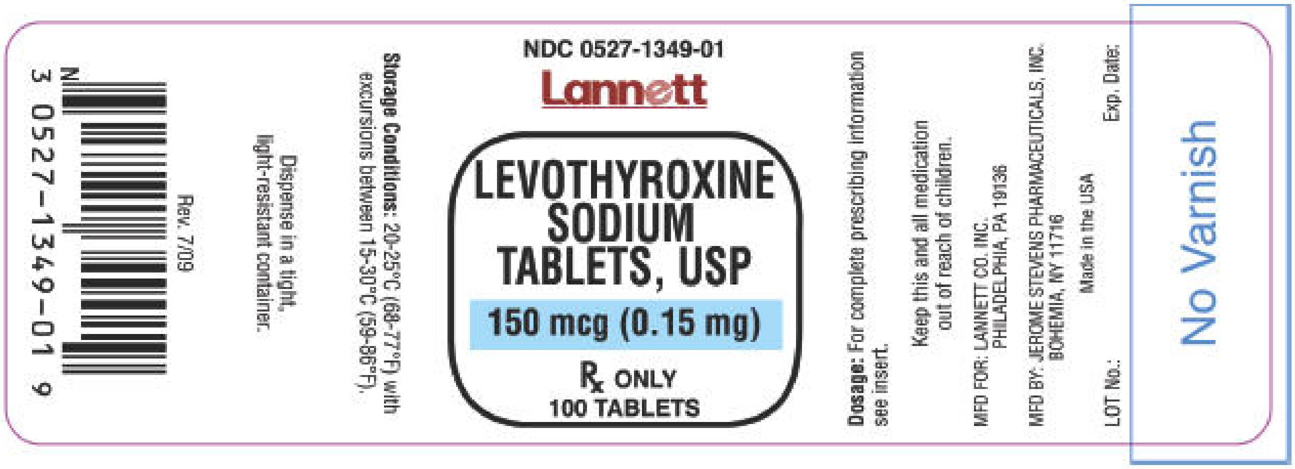

PRINCIPAL DISPLAY PANEL - 175 mcg (0.175 mg)

NDC 0527-1350-01

Lannett

LEVOTHYROXINE
SODIUM
TABLETS, USP

175 mcg (0.175 mg)

Rx ONLY

100 TABLETS

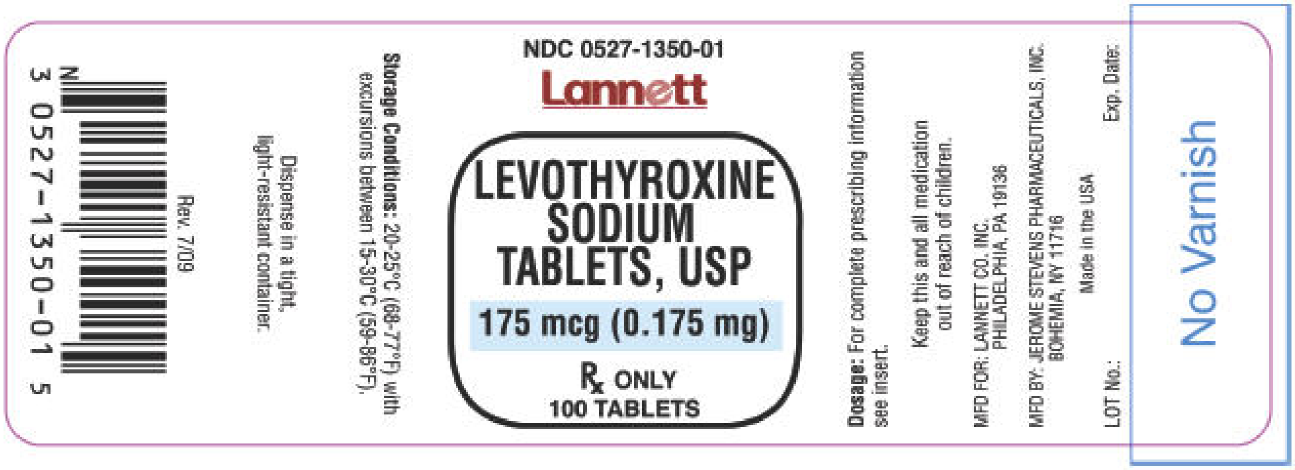

PRINCIPAL DISPLAY PANEL - 200 mcg (0.2 mg)

NDC 0527-1351-01

Lannett

LEVOTHYROXINE
SODIUM
TABLETS, USP

200 mcg (0.2 mg)

Rx ONLY

100 TABLETS

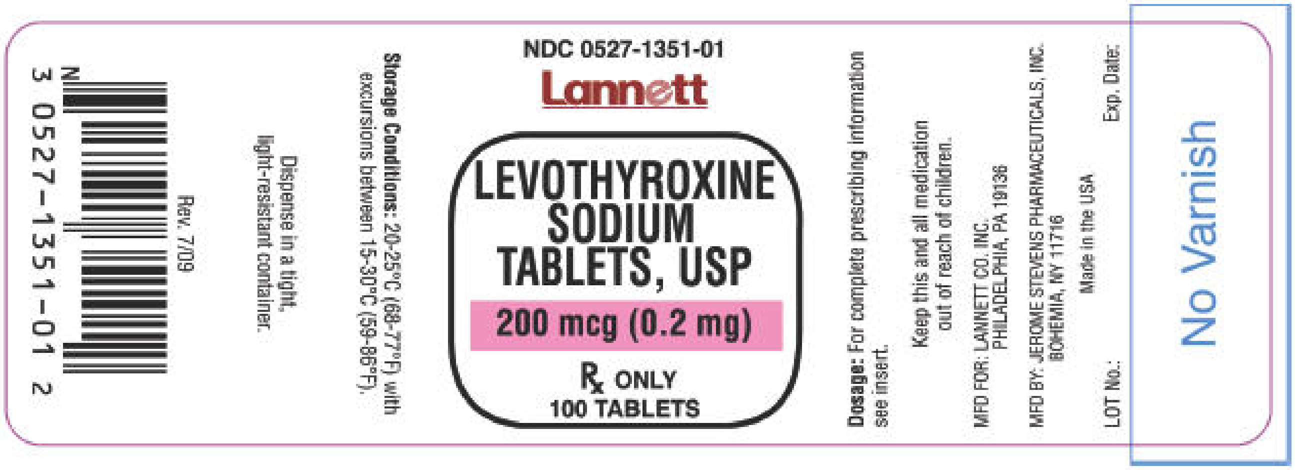

PRINCIPAL DISPLAY PANEL - 300 mcg (0.3 mg)

NDC 0527-1352-01

Lannett

LEVOTHYROXINE
SODIUM
TABLETS, USP

300 mcg (0.3 mg)

Rx ONLY

100 TABLETS

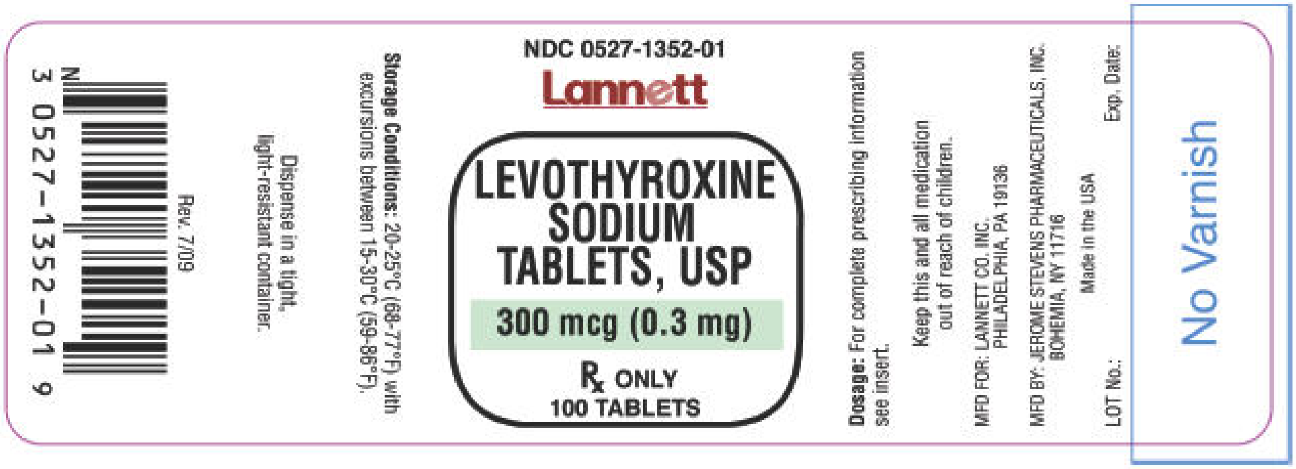

LEVOTHYROXINE SODIUM

levothyroxine sodium tablet

Product Information

**Product Type**	HUMAN PRESCRIPTION DRUG	**Item Code (Source)**	NDC:0527-1341
**Route of Administration**	ORAL		

Active Ingredient/Active Moiety

Ingredient Name	Basis of Strength	Strength
**LEVOTHYROXINE SODIUM** (UNII: 9J765S329G) (LEVOTHYROXINE - UNII:Q51BO43MG4)	LEVOTHYROXINE SODIUM	0.025 mg

Inactive Ingredients

Ingredient Name	Strength
**SILICON DIOXIDE** (UNII: ETJ7Z6XBU4)	
**LACTOSE** (UNII: J2B2A4N98G)	
**MAGNESIUM STEARATE** (UNII: 70097M6I30)	
**CELLULOSE, MICROCRYSTALLINE** (UNII: OP1R32D61U)	
**STARCH, CORN** (UNII: O8232NY3SJ)	
**ACACIA** (UNII: 5C5403N26O)	
**SODIUM STARCH GLYCOLATE TYPE A POTATO** (UNII: 5856J3G2A2)	
**FD&C YELLOW NO. 6** (UNII: H77VEI93A8)	
**ALUMINUM OXIDE** (UNII: LMI26O6933)	

Product Characteristics

**Color**	ORANGE (Peach)	**Score**	2 pieces
**Shape**	ROUND	**Size**	7mm
**Flavor**		**Imprint Code**	JSP;513
**Contains**			

Packaging

#	Item Code	Package Description	Marketing Start Date	Marketing End Date
1	NDC:0527-1341-01	100 in 1 BOTTLE, PLASTIC		
2	NDC:0527-1341-10	1000 in 1 BOTTLE, PLASTIC		

Marketing Information

Marketing Category	Application Number or Monograph Citation	Marketing Start Date	Marketing End Date
NDA	NDA021210	12/01/2003	

LEVOTHYROXINE SODIUM

levothyroxine sodium tablet

Product Information

**Product Type**	HUMAN PRESCRIPTION DRUG	**Item Code (Source)**	NDC:0527-1342
**Route of Administration**	ORAL		

Active Ingredient/Active Moiety

Ingredient Name	Basis of Strength	Strength
**LEVOTHYROXINE SODIUM** (UNII: 9J765S329G) (LEVOTHYROXINE - UNII:Q51BO43MG4)	LEVOTHYROXINE SODIUM	0.05 mg

Inactive Ingredients

Ingredient Name	Strength
**SILICON DIOXIDE** (UNII: ETJ7Z6XBU4)	
**LACTOSE** (UNII: J2B2A4N98G)	
**MAGNESIUM STEARATE** (UNII: 70097M6I30)	
**CELLULOSE, MICROCRYSTALLINE** (UNII: OP1R32D61U)	
**STARCH, CORN** (UNII: O8232NY3SJ)	
**ACACIA** (UNII: 5C5403N26O)	
**SODIUM STARCH GLYCOLATE TYPE A POTATO** (UNII: 5856J3G2A2)	

Product Characteristics

**Color**	WHITE	**Score**	2 pieces
**Shape**	ROUND	**Size**	7mm
**Flavor**		**Imprint Code**	JSP;514
**Contains**			

Packaging

#	Item Code	Package Description	Marketing Start Date	Marketing End Date
1	NDC:0527-1342-01	100 in 1 BOTTLE, PLASTIC		
2	NDC:0527-1342-10	1000 in 1 BOTTLE, PLASTIC		

Marketing Information			
**Marketing Category**	**Application Number or Monograph Citation**	**Marketing Start Date**	**Marketing End Date**
NDA	NDA021210	12/01/2003	

LEVOTHYROXINE SODIUM

levothyroxine sodium tablet

Product Information			
**Product Type**	HUMAN PRESCRIPTION DRUG	**Item Code (Source)**	NDC:0527-1343
**Route of Administration**	ORAL		

Active Ingredient/Active Moiety		
**Ingredient Name**	**Basis of Strength**	**Strength**
**LEVOTHYROXINE SODIUM** (UNII: 9J765S329G) (LEVOTHYROXINE - UNII:Q51BO43MG4)	LEVOTHYROXINE SODIUM	0.075 mg

Inactive Ingredients	
**Ingredient Name**	**Strength**
**SILICON DIOXIDE** (UNII: ETJ7Z6XBU4)	
**LACTOSE** (UNII: J2B2A4N98G)	
**MAGNESIUM STEARATE** (UNII: 70097M6I30)	
**CELLULOSE, MICROCRYSTALLINE** (UNII: OP1R32D61U)	
**STARCH, CORN** (UNII: O8232NY3SJ)	
**ACACIA** (UNII: 5C5403N26O)	
**SODIUM STARCH GLYCOLATE TYPE A POTATO** (UNII: 5856J3G2A2)	
**FD&C RED NO. 40** (UNII: WZB9127XOA)	
**FD&C BLUE NO. 2** (UNII: L06K8R7DQK)	
**ALUMINUM OXIDE** (UNII: LMI26O6933)	

Product Characteristics			
**Color**	PURPLE	**Score**	2 pieces
**Shape**	ROUND	**Size**	7mm
**Flavor**		**Imprint Code**	JSP;515
**Contains**			

Packaging				
**#**	**Item Code**	**Package Description**	**Marketing Start Date**	**Marketing End Date**
**1**	NDC:0527-1343-01	100 in 1 BOTTLE, PLASTIC		
**2**	NDC:0527-1343-10	1000 in 1 BOTTLE, PLASTIC		

Marketing Information

Marketing Category	Application Number or Monograph Citation	Marketing Start Date	Marketing End Date
NDA	NDA021210	12/01/2003	

LEVOTHYROXINE SODIUM

levothyroxine sodium tablet

Product Information

**Product Type**	HUMAN PRESCRIPTION DRUG	**Item Code (Source)**	NDC:0527-1344
**Route of Administration**	ORAL		

Active Ingredient/Active Moiety

Ingredient Name	Basis of Strength	Strength
**LEVOTHYROXINE SODIUM** (UNII: 9J765S329G) (LEVOTHYROXINE - UNII:Q51BO43MG4)	LEVOTHYROXINE SODIUM	0.088 mg

Inactive Ingredients

Ingredient Name	Strength
**SILICON DIOXIDE** (UNII: ETJ7Z6XBU4)	
**LACTOSE** (UNII: J2B2A4N98G)	
**MAGNESIUM STEARATE** (UNII: 70097M6I30)	
**CELLULOSE, MICROCRYSTALLINE** (UNII: OP1R32D61U)	
**STARCH, CORN** (UNII: O8232NY3SJ)	
**ACACIA** (UNII: 5C5403N26O)	
**SODIUM STARCH GLYCOLATE TYPE A POTATO** (UNII: 5856J3G2A2)	
**D&C YELLOW NO. 10** (UNII: 35SW5USQ3G)	
**FD&C YELLOW NO. 6** (UNII: H77VEI93A8)	
**FD&C BLUE NO. 1** (UNII: H3R47K3TBD)	
**ALUMINUM OXIDE** (UNII: LMI26O6933)	

Product Characteristics

**Color**	GREEN (Olive)	**Score**	2 pieces
**Shape**	ROUND	**Size**	7mm
**Flavor**		**Imprint Code**	JSP;561
**Contains**			

Packaging

#	Item Code	Package Description	Marketing Start Date	Marketing End Date
**1**	NDC:0527-1344-01	100 in 1 BOTTLE, PLASTIC		
**2**	NDC:0527-1344-10	1000 in 1 BOTTLE, PLASTIC		

Marketing Information

Marketing Category	Application Number or Monograph Citation	Marketing Start Date	Marketing End Date
NDA	NDA021210	12/01/2003	

LEVOTHYROXINE SODIUM

levothyroxine sodium tablet

Product Information

**Product Type**	HUMAN PRESCRIPTION DRUG	**Item Code (Source)**	NDC:0527-1345
**Route of Administration**	ORAL		

Active Ingredient/Active Moiety

Ingredient Name	Basis of Strength	Strength
**LEVOTHYROXINE SODIUM** (UNII: 9J765S329G) (LEVOTHYROXINE - UNII:Q51BO43MG4)	LEVOTHYROXINE SODIUM	0.1 mg

Inactive Ingredients

Ingredient Name	Strength
**SILICON DIOXIDE** (UNII: ETJ7Z6XBU4)	
**LACTOSE** (UNII: J2B2A4N98G)	
**MAGNESIUM STEARATE** (UNII: 70097M6I30)	
**CELLULOSE, MICROCRYSTALLINE** (UNII: OP1R32D61U)	
**STARCH, CORN** (UNII: O8232NY3SJ)	
**ACACIA** (UNII: 5C5403N26O)	
**SODIUM STARCH GLYCOLATE TYPE A POTATO** (UNII: 5856J3G2A2)	
**D&C YELLOW NO. 10** (UNII: 35SW5USQ3G)	
**FD&C YELLOW NO. 6** (UNII: H77VEI93A8)	
**ALUMINUM OXIDE** (UNII: LMI26O6933)	

Product Characteristics

**Color**	YELLOW	**Score**	2 pieces
**Shape**	ROUND	**Size**	7mm
**Flavor**		**Imprint Code**	JSP;516
**Contains**			

Packaging

#	Item Code	Package Description	Marketing Start Date	Marketing End Date
1	NDC:0527-1345-01	100 in 1 BOTTLE, PLASTIC		
2	NDC:0527-1345-10	1000 in 1 BOTTLE, PLASTIC		

Marketing Information

Marketing Category	Application Number or Monograph Citation	Marketing Start Date	Marketing End Date
NDA	NDA021210	12/01/2003	

LEVOTHYROXINE SODIUM

levothyroxine sodium tablet

Product Information

**Product Type**	HUMAN PRESCRIPTION DRUG	**Item Code (Source)**	NDC:0527-1346
**Route of Administration**	ORAL		

Active Ingredient/Active Moiety

Ingredient Name	Basis of Strength	Strength
**LEVOTHYROXINE SODIUM** (UNII: 9J765S329G) (LEVOTHYROXINE - UNII:Q51BO43MG4)	LEVOTHYROXINE SODIUM	0.112 mg

Inactive Ingredients

Ingredient Name	Strength
**SILICON DIOXIDE** (UNII: ETJ7Z6XBU4)	
**LACTOSE** (UNII: J2B2A4N98G)	
**MAGNESIUM STEARATE** (UNII: 70097M6I30)	
**CELLULOSE, MICROCRYSTALLINE** (UNII: OP1R32D61U)	
**STARCH, CORN** (UNII: O8232NY3SJ)	
**ACACIA** (UNII: 5C5403N26O)	
**SODIUM STARCH GLYCOLATE TYPE A POTATO** (UNII: 5856J3G2A2)	
**D&C RED NO. 27** (UNII: 2LRS185U6K)	
**ALUMINUM OXIDE** (UNII: LMI26O6933)	

Product Characteristics

**Color**	RED (Rose)	**Score**	2 pieces
**Shape**	ROUND	**Size**	7mm
**Flavor**		**Imprint Code**	JSP;562
**Contains**			

Packaging

#	Item Code	Package Description	Marketing Start Date	Marketing End Date
1	NDC:0527-1346-01	100 in 1 BOTTLE, PLASTIC		
2	NDC:0527-1346-10	1000 in 1 BOTTLE, PLASTIC		

Marketing Information

Marketing Category	Application Number or Monograph Citation	Marketing Start Date	Marketing End Date
NDA	NDA021210	12/01/2003	

LEVOTHYROXINE SODIUM

levothyroxine sodium tablet

Product Information			
**Product Type**	HUMAN PRESCRIPTION DRUG	**Item Code (Source)**	NDC:0527-1347
**Route of Administration**	ORAL		

Active Ingredient/Active Moiety

Ingredient Name	Basis of Strength	Strength
**LEVOTHYROXINE SODIUM** (UNII: 9J765S329G) (LEVOTHYROXINE - UNII:Q51BO43MG4)	LEVOTHYROXINE SODIUM	0.125 mg

Inactive Ingredients

Ingredient Name	Strength
**SILICON DIOXIDE** (UNII: ETJ7Z6XBU4)	
**LACTOSE** (UNII: J2B2A4N98G)	
**MAGNESIUM STEARATE** (UNII: 70097M6I30)	
**CELLULOSE, MICROCRYSTALLINE** (UNII: OP1R32D61U)	
**STARCH, CORN** (UNII: O8232NY3SJ)	
**ACACIA** (UNII: 5C5403N26O)	
**SODIUM STARCH GLYCOLATE TYPE A POTATO** (UNII: 5856J3G2A2)	
**FD&C YELLOW NO. 6** (UNII: H77VEI93A8)	
**FD&C RED NO. 40** (UNII: WZB9127XOA)	
**FD&C BLUE NO. 1** (UNII: H3R47K3TBD)	
**ALUMINUM OXIDE** (UNII: LMI26O6933)	

Product Characteristics			
**Color**	BROWN (Tan)	**Score**	2 pieces
**Shape**	ROUND	**Size**	7mm
**Flavor**		**Imprint Code**	JSP;519
**Contains**			

Packaging

#	Item Code	Package Description	Marketing Start Date	Marketing End Date
1	NDC:0527-1347-01	100 in 1 BOTTLE, PLASTIC		
2	NDC:0527-1347-10	1000 in 1 BOTTLE, PLASTIC		

Marketing Information

Marketing Category	Application Number or Monograph Citation	Marketing Start Date	Marketing End Date
NDA	NDA021210	12/01/2003	

LEVOTHYROXINE SODIUM
levothyroxine sodium tablet

Product Information			
**Product Type**	HUMAN PRESCRIPTION DRUG	**Item Code (Source)**	NDC:0527-1638
**Route of Administration**	ORAL		

Active Ingredient/Active Moiety

Ingredient Name	Basis of Strength	Strength
**LEVOTHYROXINE SODIUM** (UNII: 9J765S329G) (LEVOTHYROXINE - UNII:Q51BO43MG4)	LEVOTHYROXINE SODIUM	0.137 mg

Inactive Ingredients

Ingredient Name	Strength
**SILICON DIOXIDE** (UNII: ETJ7Z6XBU4)	
**LACTOSE** (UNII: J2B2A4N98G)	
**MAGNESIUM STEARATE** (UNII: 70097M6I30)	
**CELLULOSE, MICROCRYSTALLINE** (UNII: OP1R32D61U)	
**STARCH, CORN** (UNII: O8232NY3SJ)	
**ACACIA** (UNII: 5C5403N26O)	
**SODIUM STARCH GLYCOLATE TYPE A POTATO** (UNII: 5856J3G2A2)	
**FD&C BLUE NO. 1** (UNII: H3R47K3TBD)	
**ALUMINUM OXIDE** (UNII: LMI26O6933)	

Product Characteristics			
**Color**	BLUE	**Score**	2 pieces
**Shape**	ROUND	**Size**	7mm
**Flavor**		**Imprint Code**	JSP;564
**Contains**			

Packaging

#	Item Code	Package Description	Marketing Start Date	Marketing End Date
1	NDC:0527-1638-01	100 in 1 BOTTLE, PLASTIC		
2	NDC:0527-1638-10	1000 in 1 BOTTLE, PLASTIC		

Marketing Information

Marketing Category	Application Number or Monograph Citation	Marketing Start Date	Marketing End Date
NDA	NDA021210	10/01/2007	

LEVOTHYROXINE SODIUM

levothyroxine sodium tablet

Product Information

**Product Type**	HUMAN PRESCRIPTION DRUG	**Item Code (Source)**	NDC:0527-1349
**Route of Administration**	ORAL		

Active Ingredient/Active Moiety

Ingredient Name	Basis of Strength	Strength
**LEVOTHYROXINE SODIUM** (UNII: 9J765S329G) (LEVOTHYROXINE - UNII:Q51BO43MG4)	LEVOTHYROXINE SODIUM	0.15 mg

Inactive Ingredients

Ingredient Name	Strength
**SILICON DIOXIDE** (UNII: ETJ7Z6XBU4)	
**LACTOSE** (UNII: J2B2A4N98G)	
**MAGNESIUM STEARATE** (UNII: 70097M6I30)	
**CELLULOSE, MICROCRYSTALLINE** (UNII: OP1R32D61U)	
**STARCH, CORN** (UNII: O8232NY3SJ)	
**ACACIA** (UNII: 5C5403N26O)	
**SODIUM STARCH GLYCOLATE TYPE A POTATO** (UNII: 5856J3G2A2)	
**FD&C BLUE NO. 2** (UNII: L06K8R7DQK)	
**ALUMINUM OXIDE** (UNII: LMI26O6933)	

Product Characteristics

**Color**	BLUE (Light Blue)	**Score**	2 pieces
**Shape**	ROUND	**Size**	7mm
**Flavor**		**Imprint Code**	JSP;520
**Contains**			

Packaging

#	Item Code	Package Description	Marketing Start Date	Marketing End Date
**1**	NDC:0527-1349-01	100 in 1 BOTTLE, PLASTIC		
**2**	NDC:0527-1349-10	1000 in 1 BOTTLE, PLASTIC		

Marketing Information

Marketing Category	Application Number or Monograph Citation	Marketing Start Date	Marketing End Date
NDA	NDA021210	12/01/2003	

LEVOTHYROXINE SODIUM

levothyroxine sodium tablet

Product Information

**Product Type**	HUMAN PRESCRIPTION DRUG	**Item Code (Source)**	NDC:0527-1350
**Route of Administration**	ORAL		

Active Ingredient/Active Moiety

Ingredient Name	Basis of Strength	Strength
**LEVOTHYROXINE SODIUM** (UNII: 9J765S329G) (LEVOTHYROXINE - UNII:Q51BO43MG4)	LEVOTHYROXINE SODIUM	0.175 mg

Inactive Ingredients

Ingredient Name	Strength
**SILICON DIOXIDE** (UNII: ETJ7Z6XBU4)	
**LACTOSE** (UNII: J2B2A4N98G)	
**MAGNESIUM STEARATE** (UNII: 70097M6I30)	
**CELLULOSE, MICROCRYSTALLINE** (UNII: OP1R32D61U)	
**STARCH, CORN** (UNII: O8232NY3SJ)	
**ACACIA** (UNII: 5C5403N26O)	
**SODIUM STARCH GLYCOLATE TYPE A POTATO** (UNII: 5856J3G2A2)	
**FD&C BLUE NO. 1** (UNII: H3R47K3TBD)	
**D&C RED NO. 27** (UNII: 2LRS185U6K)	
**ALUMINUM OXIDE** (UNII: LMI26O6933)	

Product Characteristics

**Color**	PURPLE (Lilac)	**Score**	2 pieces
**Shape**	ROUND	**Size**	7mm
**Flavor**		**Imprint Code**	JSP;563
**Contains**			

Packaging

#	Item Code	Package Description	Marketing Start Date	Marketing End Date
**1**	NDC:0527-1350-01	100 in 1 BOTTLE, PLASTIC		
**2**	NDC:0527-1350-10	1000 in 1 BOTTLE, PLASTIC		

Marketing Information

Marketing Category	Application Number or Monograph Citation	Marketing Start Date	Marketing End Date
NDA	NDA021210	12/01/2003	

LEVOTHYROXINE SODIUM

levothyroxine sodium tablet

Product Information			
**Product Type**	HUMAN PRESCRIPTION DRUG	**Item Code (Source)**	NDC:0527-1351
**Route of Administration**	ORAL		

Active Ingredient/Active Moiety

Ingredient Name	Basis of Strength	Strength
**LEVOTHYROXINE SODIUM** (UNII: 9J765S329G) (LEVOTHYROXINE - UNII:Q51BO43MG4)	LEVOTHYROXINE SODIUM	0.2 mg

Inactive Ingredients

Ingredient Name	Strength
**SILICON DIOXIDE** (UNII: ETJ7Z6XBU4)	
**LACTOSE** (UNII: J2B2A4N98G)	
**MAGNESIUM STEARATE** (UNII: 70097M6I30)	
**CELLULOSE, MICROCRYSTALLINE** (UNII: OP1R32D61U)	
**STARCH, CORN** (UNII: O8232NY3SJ)	
**ACACIA** (UNII: 5C5403N26O)	
**SODIUM STARCH GLYCOLATE TYPE A POTATO** (UNII: 5856J3G2A2)	
**FD&C RED NO. 40** (UNII: WZB9127XOA)	
**ALUMINUM OXIDE** (UNII: LMI26O6933)	

Product Characteristics			
**Color**	PINK	**Score**	2 pieces
**Shape**	ROUND	**Size**	7mm
**Flavor**		**Imprint Code**	JSP;522
**Contains**			

Packaging

#	Item Code	Package Description	Marketing Start Date	Marketing End Date
**1**	NDC:0527-1351-01	100 in 1 BOTTLE, PLASTIC		
**2**	NDC:0527-1351-10	1000 in 1 BOTTLE, PLASTIC		

Marketing Information

Marketing Category	Application Number or Monograph Citation	Marketing Start Date	Marketing End Date
NDA	NDA021210	12/01/2003	

LEVOTHYROXINE SODIUM

levothyroxine sodium tablet

Product Information

**Product Type**	HUMAN PRESCRIPTION DRUG	**Item Code (Source)**	NDC:0527-1352
**Route of Administration**	ORAL		

Active Ingredient/Active Moiety

Ingredient Name	Basis of Strength	Strength
**LEVOTHYROXINE SODIUM** (UNII: 9J765S329G) (LEVOTHYROXINE - UNII:Q51BO43MG4)	LEVOTHYROXINE SODIUM	0.3 mg

Inactive Ingredients

Ingredient Name	Strength
**SILICON DIOXIDE** (UNII: ETJ7Z6XBU4)	
**LACTOSE** (UNII: J2B2A4N98G)	
**MAGNESIUM STEARATE** (UNII: 70097M6I30)	
**CELLULOSE, MICROCRYSTALLINE** (UNII: OP1R32D61U)	
**STARCH, CORN** (UNII: O8232NY3SJ)	
**ACACIA** (UNII: 5C5403N26O)	
**SODIUM STARCH GLYCOLATE TYPE A POTATO** (UNII: 5856J3G2A2)	
**D&C YELLOW NO. 10** (UNII: 35SW5USQ3G)	
**FD&C YELLOW NO. 6** (UNII: H77VEI93A8)	
**FD&C BLUE NO. 1** (UNII: H3R47K3TBD)	
**ALUMINUM OXIDE** (UNII: LMI26O6933)	

Product Characteristics

**Color**	GREEN	**Score**	2 pieces
**Shape**	ROUND	**Size**	7mm
**Flavor**		**Imprint Code**	JSP;523
**Contains**			

Packaging

#	Item Code	Package Description	Marketing Start Date	Marketing End Date
1	NDC:0527-1352-01	100 in 1 BOTTLE, PLASTIC		
2	NDC:0527-1352-10	1000 in 1 BOTTLE, PLASTIC		

Marketing Information

Marketing Category	Application Number or Monograph Citation	Marketing Start Date	Marketing End Date
NDA	NDA021210	12/01/2003	

Labeler - Lannett Company, Inc. (002277481)

Establishment			
**Name**	**Address**	**ID/FEI**	**Business Operations**
Jerome Stevens Pharmaceuticals		021130638	ANALYSIS(0527-1341, 0527-1342, 0527-1343, 0527-1344, 0527-1345, 0527-1346, 0527-1347, 0527-1638, 0527-1349, 0527-1350, 0527-1351, 0527-1352) , LABEL(0527-1341, 0527-1342, 0527-1343, 0527-1344, 0527-1345, 0527-1346, 0527-1347, 0527-1638, 0527-1349, 0527-1350, 0527-1351, 0527-1352) , MANUFACTURE(0527-1341, 0527-1342, 0527-1343, 0527-1344, 0527-1345, 0527-1346, 0527-1347, 0527-1638, 0527-1349, 0527-1350, 0527-1351, 0527-1352) , PACK(0527-1341, 0527-1342, 0527-1343, 0527-1344, 0527-1345, 0527-1346, 0527-1347, 0527-1638, 0527-1349, 0527-1350, 0527-1351, 0527-1352)

Revised: 3/2012 Lannett Company, Inc.
