# Supplementary material for: A comparison of the content and primary literature support for online medication information provided by Lexicomp and Wikipedia
Source: J Med Libr Assoc. 2018 Jul 1;106(3):352–60. doi: 10.5195/jmla.2018.256 (PMC6013145; doi:10.5195/jmla.2018.256)
Supplement: Appendix D [file jmla-106-352-s004.pdf]

**METFORMIN HCL - metformin hcl tablet**
**Ascend Laboratories, LLC**

----------

**Metformin Hydrochloride Tablets USP**

## DESCRIPTION

Metformin Hydrochloride Tablets USP are oral antihyperglycemic drugs used in the management of type 2 diabetes. Metformin hydrochloride (N,N-dimethylimidodicarbonimidic diamide hydrochloride) is not chemically or pharmacologically related to any other classes of oral antihyperglycemic agents. The structural formula is as shown:

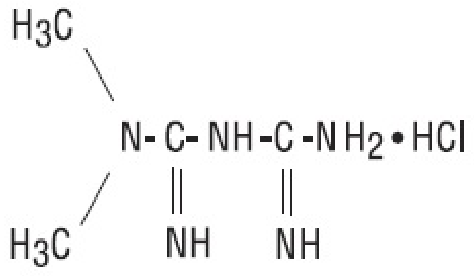


Metformin hydrochloride is a white to off-white crystalline compound with a molecular formula of $C_4H_{11}N_5$• HCl and a molecular weight of 165.63. Metformin hydrochloride is freely soluble in water and is practically insoluble in acetone, ether, and chloroform. The pKa of metformin is 12.4. The pH of a 1% aqueous solution of metformin hydrochloride is 6.68.

Metformin Hydrochloride Tablets USP contain 500 mg, 850 mg, or 1000 mg of metformin hydrochloride. Each tablet contains the inactive ingredients povidone (K-30), povidone (K-90), pregelatinized starch,and magnesium stearate. In addition, the coating for the tablets contains artificial blackberry flavor, hypromellose and polyethylene glycol.

## CLINICAL PHARMACOLOGY

### Mechanism of Action

Metformin is an antihyperglycemic agent which improves glucose tolerance in patients with type 2 diabetes, lowering both basal and postprandial plasma glucose. Its pharmacologic mechanisms of action are different from other classes of oral antihyperglycemic agents. Metformin decreases hepatic glucose production, decreases intestinal absorption of glucose, and improves insulin sensitivity by increasing peripheral glucose uptake and utilization. Unlike sulfonylureas, metformin does not produce hypoglycemia in either patients with type 2 diabetes or normal subjects (except in special circumstances, see PRECAUTIONS) and does not cause hyperinsulinemia. With metformin therapy, insulin secretion remains unchanged while fasting insulin levels and day-long plasma insulin response may actually decrease.

### Pharmacokinetics

Absorption and Bioavailability

The absolute bioavailability of a Metformin Hydrochloride 500 mg tablet given under fasting conditions is approximately 50% to 60%. Studies using single oral doses of Metformin Hydrochloride 500 mg to 1500 mg, and 850 mg to 2550 mg, indicate that there is a lack of dose proportionality with

increasing doses, which is due to decreased absorption rather than an alteration in elimination. Food decreases the extent of and slightly delays the absorption of metformin, as shown by approximately a 40% lower mean peak plasma concentration ($C_{max}$), a 25% lower area under the plasma concentration versus time curve (AUC), and a 35-minute prolongation of time to peak plasma concentration ($T_{max}$) following administration of a single 850 mg tablet of metformin with food, compared to the same tablet strength administered fasting. The clinical relevance of these decreases is unknown.

Distribution

The apparent volume of distribution (V/F) of metformin following single oral doses of Metformin Hydrochloride Tablets USP 850 mg averaged 654 ± 358 L. Metformin is negligibly bound to plasma proteins, in contrast to sulfonylureas, which are more than 90% protein bound. Metformin partitions into erythrocytes, most likely as a function of time. At usual clinical doses and dosing schedules of Metformin Hydrochloride Tablets USP, steady state plasma concentrations of metformin are reached within 24 to 48 hours and are generally <1mcg/mL. During controlled clinical trials of Metformin Hydrochloride Tablets USP, maximum metformin plasma levels did not exceed 5 mcg/mL, even at maximum doses.

Metabolism and Elimination

Intravenous single-dose studies in normal subjects demonstrate that metformin is excreted unchanged in the urine and does not undergo hepatic metabolism (no metabolites have been identified in humans) nor biliary excretion. Renal clearance (see Table 1) is approximately 3.5 times greater than creatinine clearance, which indicates that tubular secretion is the major route of metformin elimination. Following oral administration, approximately 90% of the absorbed drug is eliminated via the renal route within the first 24 hours, with a plasma elimination half-life of approximately 6.2 hours. In blood, the elimination half-life is approximately 17.6 hours, suggesting that the erythrocyte mass may be a compartment of distribution.

**Special Populations**

*Patients with Type 2 Diabetes*
In the presence of normal renal function, there are no differences between single- or multiple-dose pharmacokinetics of metformin between patients with type 2 diabetes and normal subjects (see **Table 1**), nor is there any accumulation of metformin in either group at usual clinical doses

*Renal Insufficiency*
In patients with decreased renal function (based on measured creatinine clearance), the plasma and blood half-life of metformin is prolonged and the renal clearance is decreased in proportion to the decrease in creatinine clearance (see **Table 1**; also see **WARNINGS**).

*Hepatic Insufficiency*
No pharmacokinetic studies of metformin have been conducted in patients with hepatic insufficiency.

*Geriatrics*
Limited data from controlled pharmacokinetic studies of Metformin Hydrochloride Tablets USP in healthy elderly subjects suggest that total plasma clearance of metformin is decreased, the half-life is prolonged, and $C_{max}$ is increased, compared to healthy young subjects. From these data, it appears that the change in metformin pharmacokinetics with aging is primarily accounted for by a change in renal function (see **Table 1**). Metformin Hydrochloride Tablets USP treatment should not be initiated in patients ≥80 years of age unless measurement of creatinine clearance demonstrates that renal function is not reduced (see **WARNINGS** and **DOSAGE AND ADMINISTRATION**)

**Table 1: Select Mean (±S.D.) Metformin Pharmacokinetic Parameters Following**

| Single or Multiple Oral Doses of Metformin Hydrochloride Tablets | | | |
|---|---|---|---|
| **Subject Groups: Metformin Hydrochloride Tablets dose[a] (number of subjects)** | **$C_{max}$[b] (μg/mL)** | **$T_{max}$[o] (hrs)** | **Renal Clearance (mL/min)** |
| **Healthy, nondiabetic adults:** | | | |
| 500 mg single dose (24) | 1.03 (±0.33) | 2.75 (±0.81) | 600 (±132) |
| 850 mg single dose (74)[d] | 1.60 (±0.38) | 2.64 (±0.82) | 552 (±139) |
| 850 mg three times daily for 19 doses[e] (9) | 2.01 (±0.42) | 1.79 (±0.94) | 642 (±173) |
| **Adults with type 2 diabetes:** | | | |
| 850 mg single dose (23) | 1.48 (±0.5) | 3.32 (±1.08) | 491 (±138) |
| 850 mg three times daily for 19 doses[e] (9) | 1.90 (±0.62) | 2.01 (±1.22) | 550 (±160) |
| **Elderly[f] , healthy nondiabetic adults:** | | | |
| 850 mg single dose (12) | 2.45 (±0.70) | 2.71 (±1.05) | 412 (±98) |
| **Renal-impaired adults:** | | | |
| **850 mg single dose** | | | |
| **Mild** ($CL_{cr}$[g] 61-90 mL/min) (5) | 1.86 (±0.52) | 3.20 (±0.45) | 384 (±122) |
| **Moderate** ($CL_{cr}$ 31-60 mL/ min) (4) | 4.12 (±1.83) | 3.75 (±0.50) | 108 (±57) |
| **Severe** ($CL_{cr}$ 10-30 mL/min) (6) | 3.93 (±0.92) | 4.01 (±1.10) | 130 (±90) |

[a] All doses given fasting except the first 18 doses of the multiple dose studies

[b] Peak plasma concentration

[c] Time to peak plasma concentration

[d] Combined results (average means) of five studies: mean age 32 years (range 23-59 years)

[e] Kinetic study done following dose 19, given fasting

[f] Elderly subjects, mean age 71 years (range 65-81 years)

[g] $CL_{cr}$ = creatinine clearance normalized to body surface area of 1.73 $m^2$

*Pediatrics*

After administration of a single oral Metformin Hydrochloride 500 mg tablet with food, geometric mean metformin $C_{max}$ and AUC differed less than 5% between pediatric type 2 diabetic patients (12 to 16 years of age) and gender- and weight-matched healthy adults (20 to 45 years of age), all with normal renal function.

*Gender*

Metformin pharmacokinetic parameters did not differ significantly between normal subjects and patients with type 2 diabetes when analyzed according to gender (males = 19, females = 16). Similarly, in controlled clinical studies in patients with type 2 diabetes, the antihyperglycemic effect of Metformin Hydrochloride Tablets USP was comparable in males and females.

*Race*

No studies of metformin pharmacokinetic parameters according to race have been performed. In controlled clinical studies of Metformin Hydrochloride Tablets USP in patients with type 2 diabetes, the antihyperglycemic effect was comparable in whites (n=249), blacks (n=51), and Hispanics (n=24).

**Clinical Studies**

METFORMIN HYDROCHLORIDE TABLETS USP

In a double-blind, placebo-controlled, multicenter U.S. clinical trial involving obese patients with type 2 diabetes whose hyperglycemia was not adequately controlled with dietary management alone (baseline fasting plasma glucose [FPG] of approximately 240 mg/dL), treatment with Metformin Hydrochloride Tablets USP (up to 2550 mg/day) for 29 weeks resulted in significant mean net reductions in fasting and postprandial plasma glucose (PPG) and hemoglobin $A_{1c}$ ($HbA_{1c}$ of 59 mg/dL, 83 mg/dL, and 1.8%, respectively, compared to the placebo group (see **Table 2**)

**Table 2: Metformin Hydrochloride Tablets vs Placebo Summary of Mean Changes from Baseline* in Fasting Plasma Glucose, $HbA_{1c}$, and Body Weight, at Final Visit (29-week study)**

| | **Metformin hydrochloride tablets** | **Placebo** | **p–Value** |
|---|---|---|---|
| | **(n=141)** | **(n=145)** | |
| **FPG (mg/dL)** | | | |
| Baseline | 241.5 | 237.7 | NS** |
| Change at FINAL VISIT | -53.0 | 6.3 | 0.001 |
| **Hemoglobin $A_{1c}$ (%)** | | | |
| Baseline | 8.4 | 8.2 | NS** |
| Change at FINAL VISIT | -1.4 | 0.4 | 0.001 |
| **Body Weight (lbs)** | | | |
| Baseline | 201.0 | 206.0 | NS**t |
| Change at FINAL VISIT | -1.4 | -2.4 | NS** |

*All patients on diet therapy at baseline **Not Statistically significant

A 29-week, double-blind, placebo-controlled study of Metformin Hydrochloride Tablets USP and glyburide, alone and in combination, was conducted in obese patients with type 2 diabetes who had failed to achieve adequate glycemic control while on maximum doses of glyburide (baseline FPG of approximately 250 mg/dL) (see **Table 3**). Patients randomized to the combination arm started therapy with Metformin Hydrochloride Tablets USP 500 mg and glyburide 20 mg. At the end of each week of the first four weeks of the trial, these patients had their dosages of Metformin Hydrochloride Tablets USP increased by 500 mg if they had failed to reach target fasting plasma glucose. After week four, such dosage adjustments were made monthly, although no patient was allowed to exceed Metformin Hydrochloride Tablets USP 2500 mg. Patients in the Metformin Hydrochloride Tablets USP only arm (metformin plus placebo) followed the same titration schedule. At the end of the trial, approximately 70% of the patients in the combination group were taking Metformin Hydrochloride Tablets USP 2000 mg/glyburide 20 mg or Metformin Hydrochloride Tablets USP 2500 mg/glyburide 20 mg. Patients randomized to continue on glyburide experienced worsening of glycemic control, with mean increases in FPG, PPG, and $HbA_{1c}$ of 14 mg/dL, 3 mg/dL, and 0.2%, respectively. In contrast, those randomized to Metformin Hydrochloride Tablets USP (up to 2500 mg/day) experienced a slight improvement, with mean reductions in FPG, PPG, and $HbA_{1c}$ of 1 mg/dL, 6 mg/dL, and 0.4%, respectively. The combination of Metformin Hydrochloride Tablets USP and glyburide was effective in reducing FPG, PPG, and $HbA_{1c}$ levels by 63 mg/dL, 65 mg/dL, and 1.7%, respectively. Compared to results of glyburide treatment alone, the net differences with combination treatment were -77 mg/dL, - 68 mg/dL,

and -1.9%, respectively (see **Table 3**)

| **Table 3: Combined Metformin Hydrochloride Tablets /Glyburide (Comb) vs Glyburide (Glyb) or Metformin Hydrochloride Tablets (MET) Monotherapy: Summary of Mean Changes from Baseline* in Fasting Plasma Glucose, $HbA_{1c}$, and Body Weight, at Final Visit (29-week study)** | | | | | | |
|---|---|---|---|---|---|---|
| | **Comb (n=213)** | **Glyb (n=209)** | **MET (n=210)** | **p-values** | | |
| | | | | **Glyb vs Comb** | **MET vs Comb** | **MET vs Glyb** |
| **Fasting Plasma Glucose (mg/dL)** | | | | | | |
| Baseline | 250.5 | 247.5 | 253.9 | NS** | NS** | NS** |
| Change at FINAL VISIT | -63.5 | 13.7 | -0.9 | 0.001 | 0.001 | 0.025 |
| **Hemoglobin $A_{1c}$ (%)** | | | | | | |
| Baseline | 8.8 | 8.5 | 8.9 | NS** | NS** | 0.007 |
| Change at FINAL VISIT | -1.7 | 0.2 | -0.4 | 0.001 | 0.001 | 0.001 |
| **Body Weight (lbs)** | | | | | | |
| Baseline | 202.2 | 203.0 | 204.0 | NS** | NS** | NS** |
| Change at FINAL VISIT | 0.9 | -0.7 | -8.4 | 0.011 | 0.001 | 0.001 |

*All patients on glyburide, 20 mg/day, at Baseline **Not statistically significant

The magnitude of the decline in fasting blood glucose concentration following the institution of Metformin Hydrochloride Tablets USP therapy was proportional to the level of fasting hyperglycemia. Patients with type 2 diabetes with higher fasting glucose concentrations experienced greater declines in plasma glucose and glycosylated hemoglobin.

In clinical studies, Metformin Hydrochloride Tablets USP, alone or in combination with a sulfonylurea, lowered mean fasting serum triglycerides, total cholesterol, and LDL cholesterol levels and had no adverse effects on other lipid levels (see **Table 4**).

| **Table 4: Summary of Mean Percent Change From Baseline of Major Serum Lipid Variables at Final Visit (29-week studies)** | | | | | |
|---|---|---|---|---|---|
| | **Metformin hydrochloride tablets vs Placebo** | | **Combined Metformin hydrochloride Tablets /Glyburide vs Monotherapy** | | |
| | **Metformin hydrochloride tablets (n=141)** | **Placebo (n=145)** | **Metformin hydrochloride tablets (n=210)** | **Metformin hydrochloride tablets/ Glyburide (n=213)** | **Glyburide (n=209)** |
| **Total Cholesterol (mg/dL)** | | | | | |
| Baseline | 211.0 | 212.3 | 213.1 | 215.6 | 219.6 |
| Mean % Change at | | | | | |
| FINAL VISIT | -5% | 1% | -2% | -4% | 1% |
| **Total Triglycerides** | | | | | |

| | | | | | |
|---|---|---|---|---|---|
| **(mg/dL)** | | | | | |
| Baseline | 236.1 | 203.5 | 242.5 | 215.0 | 266.1 |
| Mean % Change at | | | | | |
| FINAL VISIT | -16% | 1% | -3% | -8% | 4% |
| **LDL-Cholesterol (mg/dL)** | | | | | |
| Baseline | 135.4 | 138.5 | 134.3 | 136.0 | 137.5 |
| Mean % Change at | | | | | |
| FINAL VISIT | -8% | 1% | -4% | -6% | 3% |
| **HDL-Cholesterol (mg/dL)** | | | | | |
| Baseline | 39.0 | 40.5 | 37.2 | 39.0 | 37.0 |
| Mean % Change at | | | | | |
| FINAL VISIT | 2% | -1% | 5% | 3% | 1% |

In contrast to sulfonylureas, body weight of individuals on Metformin Hydrochloride Tablets USP tended to remain stable or even decrease somewhat (see **Tables 2** and **3**).

A 24-week, double-blind, placebo-controlled study of Metformin Hydrochloride Tablets USP plus insulin versus insulin plus placebo was conducted in patients with type 2 diabetes who failed to achieve adequate glycemic control on insulin alone (see **Table 5**). Patients randomized to receive Metformin Hydrochloride Tablets USP plus insulin achieved a reduction in $HbA_{1c}$ of 2.10%, compared to a 1.56% reduction in $HbA_{1c}$ achieved by insulin plus placebo. The improvement in glycemic control was achieved at the final study visit with 16% less insulin, 93.0 U/day vs 110.6 U/day, Metformin Hydrochloride Tablets USP plus insulin versus insulin plus placebo, respectively, p=0.04

| **Table 5: Combined Metformin Hydrochloride Tablets /Insulin vs Placebo/Insulin Summary of Mean Changes from Baseline in $HbA_{1c}$ and Daily Insulin Dose** | | | |
|---|---|---|---|
| | **Metformin Hydrochloride Tablets/ Insulin (n=26)** | **Placebo/ Insulin (n=28)** | **Treatment Difference Mean ± SE** |
| **Hemoglobin $A_{1c}$ (%)** | | | |
| Baseline | 8.95 | 9.32 | |
| Change at FINAL VISIT | -2.10 | -1.56 | -0.54 ± 0.43a |
| **Insulin Dose (U/day)** | | | |
| Baseline | 93.12 | 94.64 | |
| Change at FINAL VISIT | -0.15 | 15.93 | -16.08 ± 7.77[b] |

[a] Statistically significant using analysis of covariance with baseline as covariate (p=0.04)

Not significant using analysis of variance (values shown in table)

[b] Statistically significant for insulin (p=0.04)

A second double-blind, placebo-controlled study (n=51), with 16 weeks of randomized treatment, demonstrated that in patients with type 2 diabetes controlled on insulin for 8 weeks with an average $HbA_{1c}$ of 7.46 ± 0.97%, the addition of Metformin Hydrochloride Tablets USP maintained similar glycemic control ($HbA_{1c}$ 7.15 ± 0.61 versus 6.97 ± 0.62 for Metformin Hydrochloride Tablets USP plus insulin and placebo plus insulin, respectively) with 19% less insulin versus baseline (reduction of 23.68 ± 30.22 versus an increase of 0.43 ± 25.20 units for Metformin Hydrochloride Tablets USP plus insulin and placebo plus insulin, p<0.01). In addition, this study demonstrated that the combination of Metformin Hydrochloride Tablets USP plus insulin resulted in reduction in body weight of 3.11 ± 4.30 lbs, compared to an increase of 1.30 ± 6.08 lbs for placebo plus insulin, p=0.01.

A 24-week, double-blind, randomized study of Metformin Hydrochloride Tablets USP, taken twice daily (with breakfast and evening meal), was conducted in patients with type 2 diabetes who had been treated with Metformin Hydrochloride Tablets USP 500 mg twice daily for at least 8 weeks prior to study entry. The Metformin Hydrochloride Tablets USP dose had not necessarily been titrated to achieve a specific level of glycemic control prior to study entry. Patients qualified for the study if $HbA_{1c}$ was ≤8.5% and FPG was ≤200 mg/dL. Changes in glycemic control and body weight are shown in **Table 6**

**Table 6: Summary of Mean Changes from Baseline* in $HbA_{1c}$, Fasting Plasma Glucose, and Body Weight at Week 12 and at Final Visit (24-week study)**

| | **Metformin Hydrochloride Tablets 500 mg Twice Daily** |
|---|---|
| **Hemoglobin $A_{1c}$ (%)** | **(n=67)** |
| Baseline | 7.06 |
| Change at 12 Weeks | 0.14 |
| (95% CI) | (-0.03, 0.31) |
| Change at FINAL VISIT | 0.14[a] |
| (95% CI) | (-0.04, 0.31) |
| **FPG (mg/dL)** | **(n=69)** |
| Baseline | 127.2 |
| Change at 12 Weeks | 12.9 |
| (95% CI) | (6.5, 19.4) |
| Change at FINAL VISIT | 14.0 |
| (95% CI) | (7.0, 21.0) |
| **Body Weight (lbs)** | **(n=71)** |
| Baseline | 210.3 |
| Change at 12 Weeks | 0.4 |
| (95% CI) | (-0.4, 1.5) |
| Change at FINAL VISIT | 0.9 |
| (95% CI) | (-0.4, 2.2) |

*All patients on Metformin Hydrochloride Tablets 500mg twice daily at Baseline [a] n=68

Changes in lipid parameters in the previously described study of Metformin Hydrochloride Tablets USP are shown in **Table 7**

| Table 7: Summary of Mean Percent Changes from Baseline* in Major Lipid Variables at Final Visit (24-week study) | |
|---|---|
| | **Metformin Hydrochloride Tablets 500mg Twice Daily** |
| **Total Cholesterol (mg/dL)** | **(n=68)** |
| Baseline | 199.0 |
| Mean % Change at FINAL VISIT | 0.1% |
| **Total Triglycerides (mg/dL)** | **(n=68)** |
| Baseline | 178.0 |
| Mean % Change at FINAL VISIT | 6.3% |
| **LDL-Cholesterol (mg/dL)** | **(n=68)** |
| Baseline | 122.1 |
| Mean % Change at FINAL VISIT | -1.3% |
| **HDL-Cholesterol (mg/dL)** | **(n=68)** |
| Baseline | 41.9 |
| Mean % Change at FINAL VISIT | 4.8% |

*All patients on Metformin Hydrochloride Tablets 500mg twice daily at Baseline

**Pediatric Clinical Studies**

In a double-blind, placebo-controlled study in pediatric patients aged 10 to 16 years with type 2 diabetes (mean FPG 182.2 mg/dL), treatment with Metformin Hydrochloride Tablets USP (up to 2000 mg/day) for up to 16 weeks (mean duration of treatment 11 weeks) resulted in a significant mean net reduction in FPG of 64.3 mg/dL, compared with placebo (see **Table 8**)

| Table 8: Metformin Hydrochloride Tablets vs Placebo (Pediatrics[a]) Summary of Mean Changes from Baseline* in Plasma Glucose and Body Weight at Final Visit | | | |
|---|---|---|---|
| | **Metformin Hydrochloride Tablets** | **Placebo** | **p-Value** |
| **FPG (mg/dL)** | **(n=37)** | **(n=36)** | |
| Baseline | 162.4 | 192.3 | |
| Change at FINAL VISIT | -42.9 | 21.4 | <0.001 |
| **Body Weight (lbs)** | **(n=39)** | **(n=38)** | |
| Baseline | 205.3 | 189.0 | |
| Change at FINAL VISIT | -3.3 | -2.0 | NS** |

[a]Pediatric patients mean age 13.8 years (range 10 to 16 years)

* All patients ondiet therapy at Baseline ** Not statistically significant

## INDICATIONS & USAGE

Metformin Hydrochloride Tablets USP are indicated as an adjunct to diet and exercise to improve glycemic control in adults and children with type 2 diabetes mellitus.

**CONTRAINDICATIONS**

Metformin Hydrochloride Tablets USP are contraindicated in patients with:

1. Renal disease or renal dysfunction (e.g., as suggested by serum creatinine levels ≥1.5 mg/dL [males], ≥1.4 mg/dL [females] or abnormal creatinine clearance) which may also result from conditions such as cardiovascular collapse (shock), acute myocardial infarction, and septicemia (see **WARNINGS** and **PRECAUTIONS**).

2. Known hypersensitivity to metformin hydrochloride

3. Acute or chronic metabolic acidosis, including diabetic ketoacidosis, with or without coma. Diabetic ketoacidosis should be treated with insulin.

Metformin Hydrochloride Tablets USP should be temporarily discontinued in patients undergoing radiologic studies involving intravascular administration of iodinated contrast materials, because use of such products may result in acute alteration of renal function. (See also **PRECAUTIONS**.)

**WARNINGS**

**Lactic Acidosis:Lactic acidosis is a rare, but serious, metabolic complication that can occur due to metformin accumulation during treatment with Metformin Hydrochloride Tablets USP; when it occurs, it is fatal in approximately 50% of cases. Lactic acidosis may also occur in association with a number of pathophysiologic conditions, including diabetes mellitus, and whenever there is significant tissue hypoperfusion and hypoxemia. Lactic acidosis is characterized by elevated blood lactate levels (>5 mmol/L), decreased blood pH, electrolyte disturbances with an increased anion gap, and an increased lactate/pyruvate ratio. When metformin is implicated as the cause of lactic acidosis, metformin plasma levels >5 mcg/mL are generally found.**

**The reported incidence of lactic acidosis in patients receiving metformin hydrochloride is very low (approximately 0.03 cases/1000 patient-years, with approximately 0.015 fatal cases/1000 patient-years). In more than 20,000 patient-years exposure to metformin in clinical trials, there were no reports of lactic acidosis. Reported cases have occurred primarily in diabetic patients with significant renal insufficiency, including both intrinsic renal disease and renal hypoperfusion, often in the setting of multiple concomitant medical/surgical problems and multiple concomitant medications. Patients with congestive heart failure requiring pharmacologic management, in particular those with unstable or acute congestive heart failure who are at risk of hypoperfusion and hypoxemia, are at increased risk of lactic acidosis. The risk of lactic acidosis increases with the degree of renal dysfunction and the patient's age. The risk of lactic acidosis may, therefore, be significantly decreased by regular monitoring of renal function in patients taking Metformin Hydrochloride Tablets USP and by use of the minimum effective dose of Metformin Hydrochloride Tablets USP. In particular, treatment of the elderly should be accompanied by careful monitoring of renal function. Metformin Hydrochloride Tablets USP treatment should not be initiated in patients ≥80 years of age unless measurement of creatinine clearance demonstrates that renal function is not reduced, as these patients are more susceptible to developing lactic acidosis. In addition, Metformin Hydrochloride Tablets USP should be promptly withheld in the presence of any condition associated with hypoxemia, dehydration, or sepsis. Because impaired hepatic function may significantly limit the ability to clear lactate, Metformin Hydrochloride Tablets USP should generally be avoided in patients with clinical or laboratory evidence of hepatic disease. Patients should be cautioned against excessive alcohol**

**intake, either acute or chronic, when taking Metformin Hydrochloride Tablets USP, since alcohol potentiates the effects of metformin hydrochloride on lactate metabolism. In addition, Metformin Hydrochloride Tablets USP should be temporarily discontinued prior to any intravascular radiocontrast study and for any surgical procedure (see also PRECAUTIONS).**

**The onset of lactic acidosis often is subtle, and accompanied only by nonspecific symptoms such as malaise, myalgias, respiratory distress, increasing somnolence, and nonspecific abdominal distress. There may be associated hypothermia, hypotension, and resistant bradyarrhythmias with more marked acidosis. The patient and the patient's physician must be aware of the possible importance of such symptoms and the patient should be instructed to notify the physician immediately if they occur (see also PRECAUTIONS). Metformin Hydrochloride Tablets USP should be withdrawn until the situation is clarified. Serum electrolytes, ketones, blood glucose, and if indicated, blood pH, lactate levels, and even blood metformin levels may be useful. Once a patient is stabilized on any dose level of Metformin Hydrochloride Tablets USP, gastrointestinal symptoms, which are common during initiation of therapy, are unlikely to be drug related. Later occurrence of gastrointestinal symptoms could be due to lactic acidosis or other serious disease.**

**Levels of fasting venous plasma lactate above the upper limit of normal but less than 5 mmol/L in patients taking Metformin Hydrochloride Tablets USP do not necessarily indicate impending lactic acidosis and may be explainable by other mechanisms, such as poorly controlled diabetes or obesity, vigorous physical activity, or technical problems in sample handling. (See also PRECAUTIONS.)**

**Lactic acidosis should be suspected in any diabetic patient with metabolic acidosis lacking evidence of ketoacidosis (ketonuria and ketonemia).**

**Lactic acidosis is a medical emergency that must be treated in a hospital setting. In a patient with lactic acidosis who is taking Metformin Hydrochloride Tablets USP, the drug should be discontinued immediately and general supportive measures promptly instituted. Because metformin hydrochloride is dialyzable (with a clearance of up to 170 mL/min under good hemodynamic conditions), prompt hemodialysis is recommended to correct the acidosis and remove the accumulated metformin. Such management often results in prompt reversal of symptoms and recovery. (See also CONTRAINDICATIONS and PRECAUTIONS.)**

## PRECAUTIONS

## GENERAL PRECAUTIONS

*Macrovascular Outcomes*—There have been no clinical studies establishing conclusive evidence of macrovascular risk reduction with Metformin Hydrochloride Tablets USP for any other anti-diabetic drug.

*Monitoring of renal function*—Metformin is known to be substantially excreted by the kidney, and the risk of metformin accumulation and lactic acidosis increases with the degree of impairment of renal function. Thus, patients with serum creatinine levels above the upper limit of normal for their age should not receive Metformin Hydrochloride Tablets USP. In patients with advanced age, Metformin Hydrochloride Tablets USP should be carefully titrated to establish the minimum dose for adequate glycemic effect, because aging is associated with reduced renal function. In elderly patients, particularly those ≥80 years of age, renal function should be monitored regularly and, generally, Metformin Hydrochloride Tablets USP should not be titrated to the maximum dose (see **WARNINGS** and **DOSAGE AND ADMINISTRATION**).

Before initiation of Metformin Hydrochloride Tablets USP therapy and at least annually thereafter, renal function should be assessed and verified as normal. In patients in whom development of renal dysfunction is anticipated, renal function should be assessed more frequently and Metformin Hydrochloride Tablets USP discontinued if evidence of renal impairment is present.

*Use of concomitant medications that may affect renal function or metformin disposition*—Concomitant medication(s) that may affect renal function or result in significant hemodynamic change or may interfere with the disposition of metformin, such as cationic drugs that are eliminated by renal tubular secretion (see **PRECAUTIONS: Drug Interactions**), should be used with caution.

*Radiologic studies involving the use of intravascular iodinated contrast materials (for example, intravenous urogram, intravenous cholangiography, angiography, and computed tomography (CT) scans with intravascular contrast materials)*—Intravascular contrast studies with iodinated materials can lead to acute alteration of renal function and have been associated with lactic acidosis in patients receiving metformin (see **CONTRAINDICATIONS**). Therefore, in patients in whom any such study is planned, Metformin Hydrochloride Tablets USP should be temporarily discontinued at the time of or prior to the procedure, and withheld for 48 hours subsequent to the procedure and reinstituted only after renal function has been re-evaluated and found to be normal.

*Hypoxic states*—Cardiovascular collapse (shock) from whatever cause, acute congestive heart failure, acute myocardial infarction and other conditions characterized by hypoxemia have been associated with lactic acidosis and may also cause prerenal azotemia. When such events occur in patients on Metformin Hydrochloride Tablets USP therapy, the drug should be promptly discontinued.

*Surgical procedures*—Metformin Hydrochloride Tablets USP therapy should be temporarily suspended for any surgical procedure (except minor procedures not associated with restricted intake of food and fluids) and should not be restarted until the patient's oral intake has resumed and renal function has been evaluated as normal.

*Alcohol intake*—Alcohol is known to potentiate the effect of metformin on lactate metabolism. Patients, therefore, should be warned against excessive alcohol intake, acute or chronic, while receiving Metformin Hydrochloride Tablets USP.

*Impaired hepatic function*—Since impaired hepatic function has been associated with some cases of lactic acidosis, Metformin Hydrochloride Tablets USP should generally be avoided in patients with clinical or laboratory evidence of hepatic disease.

*Vitamin $B_{12}$ levels*—In controlled clinical trials of Metformin Hydrochloride Tablets USP of 29 weeks duration, a decrease to subnormal levels of previously normal serum vitamin $B_{12}$ levels, without clinical manifestations, was observed in approximately 7% of patients. Such decrease, possibly due to interference with $B_{12}$ absorption from the $B_{12}$-intrinsic factor complex, is, however, very rarely associated with anemia and appears to be rapidly reversible with discontinuation of Metformin Hydrochloride Tablets USP or vitamin $B_{12}$ supplementation. Measurement of hematologic parameters on an annual basis is advised in patients on Metformin Hydrochloride Tablets USP and any apparent abnormalities should be appropriately investigated and managed (see **PRECAUTIONS: Laboratory Tests**).

Certain individuals (those with inadequate vitamin $B_{12}$ or calcium intake or absorption) appear to be predisposed to developing subnormal vitamin $B_{12}$ levels. In these patients, routine serum vitamin $B_{12}$ measurements at two- to three-year intervals may be useful.

*Change in clinical status of patients with previously controlled type 2 diabetes*—A patient with type 2 diabetes previously well controlled on Metformin Hydrochloride Tablets USP who develops

laboratory abnormalities or clinical illness (especially vague and poorly defined illness) should be evaluated promptly for evidence of ketoacidosis or lactic acidosis. Evaluation should include serum electrolytes and ketones, blood glucose and, if indicated, blood pH, lactate, pyruvate, and metformin levels. If acidosis of either form occurs, Metformin Hydrochloride Tablets USP must be stopped immediately and other appropriate corrective measures initiated (see also **WARNINGS**).

*Hypoglycemia*—Hypoglycemia does not occur in patients receiving Metformin Hydrochloride Tablets USP alone under usual circumstances of use, but could occur when caloric intake is deficient, when strenuous exercise is not compensated by caloric supplementation, or during concomitant use with other glucose-lowering agents (such as sulfonylureas and insulin) or ethanol.

Elderly, debilitated, or malnourished patients, and those with adrenal or pituitary insufficiency or alcohol intoxication are particularly susceptible to hypoglycemic effects. Hypoglycemia may be difficult to recognize in the elderly, and in people who are taking beta-adrenergic blocking drugs.

*Loss of control of blood glucose*—When a patient stabilized on any diabetic regimen is exposed to stress such as fever, trauma, infection, or surgery, a temporary loss of glycemic control may occur. At such times, it may be necessary to withhold Metformin Hydrochloride Tablets USP and temporarily administer insulin. Metformin Hydrochloride Tablets USP may be reinstituted after the acute episode is resolved.

The effectiveness of oral antidiabetic drugs in lowering blood glucose to a targeted level decreases in many patients over a period of time. This phenomenon, which may be due to progression of the underlying disease or to diminished responsiveness to the drug, is known as secondary failure, to distinguish it from primary failure in which the drug is ineffective during initial therapy. Should secondary failure occur with either Metformin Hydrochloride Tablets USP or sulfonylurea monotherapy, combined therapy with Metformin Hydrochloride Tablets USP and sulfonylurea may result in a response. Should secondary failure occur with combined Metformin Hydrochloride Tablets USP/sulfonylurea therapy, it may be necessary to consider therapeutic alternatives including initiation of insulin therapy.

## INFORMATION FOR PATIENTS

Patients should be informed of the potential risks and benefits of Metformin Hydrochloride Tablets USP and of alternative modes of therapy. They should also be informed about the importance of adherence to dietary instructions, of a regular exercise program, and of regular testing of blood glucose, glycosylated hemoglobin, renal function, and hematologic parameters.

The risks of lactic acidosis, its symptoms, and conditions that predispose to its development, as noted in the **WARNINGS** and **PRECAUTIONS** sections, should be explained to patients. Patients should be advised to discontinue Metformin Hydrochloride Tablets USP immediately and to promptly notify their health practitioner if unexplained hyperventilation, myalgia, malaise, unusual somnolence, or other nonspecific symptoms occur. Once a patient is stabilized on any dose level of Metformin Hydrochloride Tablets USP, gastrointestinal symptoms, which are common during initiation of metformin therapy, are unlikely to be drug related. Later occurrence of gastrointestinal symptoms could be due to lactic acidosis or other serious disease.

Patients should be counselled against excessive alcohol intake, either acute or chronic, while receiving Metformin Hydrochloride Tablets USP.

Metformin Hydrochloride Tablets USP alone do not usually cause hypoglycemia, although it may occur when Metformin Hydrochloride Tablets USP are used in conjunction with oral sulfonylureas and insulin. When initiating combination therapy, the risks of hypoglycemia, its symptoms and treatment, and conditions that predispose to its development should be explained to patients and responsible family

members. (See **Patient Information** printed below.)

**LABORATORY TESTS**

Response to all diabetic therapies should be monitored by periodic measurements of fasting blood glucose and glycosylated hemoglobin levels, with a goal of decreasing these levels toward the normal range. During initial dose titration, fasting glucose can be used to determine the therapeutic response. Thereafter, both glucose and glycosylated hemoglobin should be monitored. Measurements of glycosylated hemoglobin may be especially useful for evaluating long-term control (see also **DOSAGE ANDADMINISTRATION**)

Initial and periodic monitoring of hematologic parameters (e.g., hemoglobin/hematocrit and red blood cell indices) and renal function (serum creatinine) should be performed, at least on an annual basis. While megaloblastic anemia has rarely been seen with Metformin Hydrochloride Tablets USP therapy, if this is suspected, vitamin $B_{12}$ deficiency should be excluded.

**DRUG INTERACTIONS**

**(Clinical Evaluation of Drug Interactions Conducted with Metformin Hydrochloride Tablets USP)**

*Glyburide*—In a single-dose interaction study in type 2 diabetes patients, coadministration of metformin and glyburide did not result in any changes in either metformin pharmacokinetics or pharmacodynamics. Decreases in glyburide AUC and $C_{max}$ were observed, but were highly variable. The single-dose nature of this study and the lack of correlation between glyburide blood levels and pharmacodynamic effects, makes the clinical significance of this interaction uncertain (see **DOSAGE AND ADMINISTRATION: Concomitant Metformin Hydrochloride Tablets USP and Oral Sulfonylurea Therapy in Adult Patients**).

*Furosemide*—A single-dose, metformin-furosemide drug interaction study in healthy subjects demonstrated that pharmacokinetic parameters of both compounds were affected by coadministration. Furosemide increased the metformin plasma and blood $C_{max}$ by 22% and blood AUC by 15%, without any significant change in metformin renal clearance. When administered with metformin, the $C_{max}$ and AUC of furosemide were 31% and 12% smaller, respectively, than when administered alone, and the terminal half-life was decreased by 32%, without any significant change in furosemide renal clearance. No information is available about the interaction of metformin and furosemide when coadministered chronically.

*Nifedipine*—A single-dose, metformin-nifedipine drug interaction study in normal healthy volunteers demonstrated that coadministration of nifedipine increased plasma metformin $C_{max}$ and AUC by 20% and 9%, respectively, and increased the amount excreted in the urine. $T_{max}$ and half-life were unaffected. Nifedipine appears to enhance the absorption of metformin. Metformin had minimal effects on nifedipine.

*Cationic drugs*—Cationic drugs (e.g., amiloride, digoxin, morphine, procainamide, quinidine, quinine, ranitidine, triamterene, trimethoprim, or vancomycin) that are eliminated by renal tubular secretion theoretically have the potential for interaction with metformin by competing for common renal tubular transport systems. Such interaction between metformin and oral cimetidine has been observed in normal healthy volunteers in both single- and multiple-dose, metformin-cimetidine drug interaction studies, with a 60% increase in peak metformin plasma and whole blood concentrations and a 40% increase in plasma and whole blood metformin AUC. There was no change in elimination half-life in the single-dose study. Metformin had no effect on cimetidine pharmacokinetics. Although such interactions remain theoretical (except for cimetidine), careful patient monitoring and dose adjustment of Metformin Hydrochloride Tablets USP and/or the interfering drug is recommended in patients who are taking cationic medications

that are excreted via the proximal renal tubular secretory system.

*Other*—Certain drugs tend to produce hyperglycemia and may lead to loss of glycemic control. These drugs include the thiazides and other diuretics, corticosteroids, phenothiazines, thyroid products, estrogens, oral contraceptives, phenytoin, nicotinic acid, sympathomimetics, calcium channel blocking drugs, and isoniazid. When such drugs are administered to a patient receiving Metformin Hydrochloride Tablets USP, the patient should be closely observed for loss of blood glucose control. When such drugs are withdrawn from a patient receiving Metformin Hydrochloride Tablets USP, the patient should be observed closely for hypoglycemia.

In healthy volunteers, the pharmacokinetics of metformin and propranolol, and metformin and ibuprofen were not affected when coadministered in single-dose interaction studies.

Metformin is negligibly bound to plasma proteins and is, therefore, less likely to interact with highly protein-bound drugs such as salicylates, sulfonamides, chloramphenicol, and probenecid, as compared to the sulfonylureas, which are extensively bound to serum proteins.

**DRUG & OR LABORATORY TEST INTERACTIONS**

**CARCINOGENESIS & MUTAGENESIS & IMPAIRMENT OF FERTILITY**

Long-term carcinogenicity studies have been performed in rats (dosing duration of 104 weeks) and mice (dosing duration of 91 weeks) at doses up to and including 900 mg/kg/day and 1500 mg/kg/day, respectively. These doses are both approximately four times the maximum recommended human daily dose of 2000 mg based on body surface area comparisons. No evidence of carcinogenicity with metformin was found in either male or female mice. Similarly, there was no tumorigenic potential observed with metformin in male rats. There was, however, an increased incidence of benign stromal uterine polyps in female rats treated with 900 mg/kg/day.

There was no evidence of a mutagenic potential of metformin in the following in vitro tests: Ames test (S. typhimurium), gene mutation test (mouse lymphoma cells), or chromosomal aberrations test (human lymphocytes). Results in the in vivo mouse micronucleus test were also negative.

Fertility of male or female rats was unaffected by metformin when administered at doses as high as 600 mg/kg/day, which is approximately three times the maximum recommended human daily dose based on body surface area comparisons.

**PREGNANCY**

*Teratogenic Effects: Pregnancy Category B*

Recent information strongly suggests that abnormal blood glucose levels during pregnancy are associated with a higher incidence of congenital abnormalities. Most experts recommend that insulin be used during pregnancy to maintain blood glucose levels as close to normal as possible. Because animal reproduction studies are not always predictive of human response, Metformin Hydrochloride Tablets USP should not be used during pregnancy unless clearly needed.

There are no adequate and well-controlled studies in pregnant women with Metformin Hydrochloride Tablets USP. Metformin was not teratogenic in rats and rabbits at doses up to 600 mg/kg/day. This represents an exposure of about two and six times the maximum recommended human daily dose of 2000 mg based on body surface area comparisons for rats and rabbits, respectively. Determination of fetal concentrations demonstrated a partial placental barrier to metformin.

**LABOR & DELIVERY**

## NURSING MOTHERS

Studies in lactatingrats show that metformin is excreted into milk and reaches levels comparable tothose in plasma. Similar studies have not been conducted in nursing mothers.Because the potential for hypoglycemia in nursing infants may exist, a decisionshould be made whether to discontinue nursing or to discontinue the drug,taking into account the importance of the drug to the mother. If Metformin HydrochlorideTablets USP are discontinued, and if diet alone is inadequate for controllingblood glucose, insulin therapy should be considered.

## PEDIATRIC USE

The safety and effectiveness of Metformin Hydrochloride Tablets USP for the treatment of type 2 diabetes have been established in pediatric patients ages 10 to 16 years (studies have not been conducted in pediatric patients below the age of 10 years). Use of Metformin Hydrochloride Tablets USP in this age group is supported by evidence from adequate and well-controlled studies of Metformin Hydrochloride Tablets USP in adults with additional data from a controlled clinical study in pediatric patients ages 10 to 16 years with type 2 diabetes, which demonstrated a similar response in glycemic control to that seen in adults. (See **CLINICAL PHARMACOLOGY: Pediatric Clinical Studies**.) In this study, adverse effects were similar to those described in adults. (See **ADVERSE REACTIONS: Pediatric Patients.**) A maximum daily dose of 2000 mg is recommended. (See **DOSAGE AND ADMINISTRATION: Recommended Dosing Schedule: Pediatrics.**)

## GERIATRIC USE

Controlled clinical studies of Metformin Hydrochloride Tablets USP did not include sufficient numbers of elderly patients to determine whether they respond differently from younger patients, although other reported clinical experience has not identified differences in responses between the elderly and younger patients. Metformin is known to be substantially excreted by the kidney and because the risk of serious adverse reactions to the drug is greater in patients with impaired renal function, Metformin Hydrochloride Tablets USP should only be used in patients with normal renal function (see **CONTRAINDICATIONS, WARNINGS**, and **CLINICAL PHARMACOLOGY: Pharmacokinetics**). Because aging is associated with reduced renal function, Metformin Hydrochloride Tablets USP should be used with caution as age increases. Care should be taken in dose selection and should be based on careful and regular monitoring of renal function. Generally, elderly patients should not be titrated to the maximum dose of Metformin Hydrochloride Tablets USP (see also **WARNINGS and DOSAGE AND ADMINISTRATION**).

## ADVERSE REACTIONS

In a US double-blind clinical study of Metformin Hydrochloride Tablets USP in patients with type 2 diabetes, a total of 141 patients received Metformin Hydrochloride Tablets USP therapy (up to 2550 mg per day) and 145 patients received placebo. Adverse reactions reported in greater than 5% of the Metformin Hydrochloride Tablets USP patients, and that were more common in Metformin Hydrochloride Tablets USP- than placebo-treated patients, are listed in **Table 9**.

**Table 9: Most Common Adverse Reactions (>5.0 Percent) in a Placebo-Controlled Clinical Study of Metformin Hydrochloride Tablets Monotherapy***

| Adverse Reaction | Metformin Hydrochloride Tablets Monotherapy (n=141) | Placebo (n=145) |
|---|---|---|
| | % of Patients | |
| Diarrhea | 53.2 | 11.7 |
| Nausea/Vomiting | 25.5 | 8.3 |
| Flatulence | 12.1 | 5.5 |

| | | |
|---|---|---|
| **Asthenia** | **9.2** | **5.5** |
| **Indigestion** | **7.1** | **4.1** |
| **Abdominal Discomfort** | **6.4** | **4.8** |
| **Headache** | **5.7** | **4.8** |

* Reactions that were more common in metformin hydrochloride tablets than placebo-treated patients.

Diarrhea led to discontinuation of study medication in 6% of patients treated with Metformin Hydrochloride Tablets USP. Additionally, the following adverse reactions were reported in ≥1.0 to ≤5.0% of Metformin Hydrochloride Tablets USP patients and were more commonly reported with Metformin Hydrochloride Tablets USP than placebo: abnormal stools, hypoglycemia, myalgia, lightheaded, dyspnea, nail disorder, rash, sweating increased, taste disorder, chest discomfort, chills, flu syndrome, flushing, palpitation.

**Pediatric Patients**
In clinical trials with Metformin Hydrochloride Tablets USP in pediatric patients with type 2 diabetes, the profile of adverse reactions was similar to that observed in adults.

## DRUG ABUSE AND DEPENDENCE

## OVERDOSAGE

Overdose of metformin hydrochloride has occurred, including ingestion of amounts greater than 50 grams. Hypoglycemia was reported in approximately 10% of cases, but no causal association with metformin hydrochloride has been established. Lactic acidosis has been reported in approximately 32% of metformin overdose cases (see **WARNINGS**). Metformin is dialyzable with a clearance of up to 170 mL/min under good hemodynamic conditions. Therefore, hemodialysis may be useful for removal of accumulated drug from patients in whom metformin overdosage is suspected.

## DOSAGE & ADMINISTRATION

There is no fixed dosage regimen for the management of hyperglycemia in patients with type 2 diabetes with metformin hydrochloride or any other pharmacologic agent. Dosage of metformin hydrochloride must be individualized on the basis of both effectiveness and tolerance, while not exceeding the maximum recommended daily doses. The maximum recommended daily dose of metformin hydrochloride is 2550 mg in adults and 2000 mg in pediatric patients (10 to 16 years of age).

Metformin hydrochloride should be given in divided doses with meals. Metformin hydrochloride should be started at a low dose, with gradual dose escalation, both to reduce gastrointestinal side effects and to permit identification of the minimum dose required for adequate glycemic control of the patient.

During treatment initiation and dose titration (see **Recommended Dosing Schedule**), fasting plasma glucose should be used to determine the therapeutic response to metformin hydrochloride and identify the minimum effective dose for the patient. Thereafter, glycosylated hemoglobin should be measured at intervals of approximately three months. **The therapeutic goal should be to decrease both fasting plasma glucose and glycosylated hemoglobin levels to normal or near normal by using the lowest effective dose of metformin hydrochloride, either when used as monotherapy or in combination with sulfonylurea or insulin.**

Monitoring of blood glucose and glycosylated hemoglobin will also permit detection of primary

failure, i.e., inadequate lowering of blood glucose at the maximum recommended dose of medication, and secondary failure, i.e., loss of an adequate blood glucose lowering response after an initial period of effectiveness.

Short-term administration of metformin hydrochloride may be sufficient during periods of transient loss of control in patients usually well-controlled on diet alone.

**Recommended Dosing Schedule**

**Adults** – In general, clinically significant responses are not seen at doses below 1500 mg per day. However, a lower recommended starting dose and gradually increased dosage is advised to minimize gastrointestinal symptoms.

The usual starting dose of metformin hydrochloride is 500 mg twice a day or 850 mg once a day, given with meals. Dosage increases should be made in increments of 500 mg weekly or 850 mg every 2 weeks, up to a total of 2000 mg per day, given in divided doses. Patients can also be titrated from 500 mg twice a day to 850 mg twice a day after 2 weeks. For those patients requiring additional glycemic control, metformin hydrochloride may be given to a maximum daily dose of 2550 mg per day. Doses above 2000 mg may be better tolerated given three times a day with meals.

If higher doses of metformin are required, metformin hydrochloride should be used at total daily doses up to 2550 mg administered in divided daily doses, as described above. (See **CLINICAL PHARMACOLOGY: Clinical Studies**.)

**Pediatrics** – The usual starting dose of metformin hydrochloride is 500 mg twice a day, given with meals. Dosage increases should be made in increments of 500 mg weekly up to a maximum of 2000 mg per day, given in divided doses.

**Transfer From Other Antidiabetic Therapy**

When transferring patients from standard oral hypoglycemic agents other than chlorpropamide to metformin hydrochloride, no transition period generally is necessary. When transferring patients from chlorpropamide, care should be exercised during the first two weeks because of the prolonged retention of chlorpropamide in the body, leading to overlapping drug effects and possible hypoglycemia.

**Concomitant Metformin Hydrochloride and Oral Sulfonylurea Therapy in Adult Patients**

If patients have not responded to four weeks of the maximum dose of metformin hydrochloride monotherapy, consideration should be given to gradual addition of an oral sulfonylurea while continuing metformin hydrochloride at the maximum dose, even if prior primary or secondary failure to a sulfonylurea has occurred. Clinical and pharmacokinetic drug-drug interaction data are currently available only for metformin plus glyburide (glibenclamide).

With concomitant metformin hydrochloride and sulfonylurea therapy, the desired control of blood glucose may be obtained by adjusting the dose of each drug. In a clinical trial of patients with type 2 diabetes and prior failure on glyburide, patients started on metformin hydrochloride 500 mg and glyburide 20 mg were titrated to 1000/20 mg, 1500/20 mg, 2000/20 mg or 2500/20 mg of metformin hydrochloride and glyburide, respectively, to reach the goal of glycemic control as measured by FPG, $HbA_{1c}$ and plasma glucose response (see **CLINICAL PHARMACOLOGY: Clinical Studies**). However, attempts should be made to identify the minimum effective dose of each drug to achieve this

goal. With concomitant metformin hydrochloride and sulfonylurea therapy, the risk of hypoglycemia associated with sulfonylurea therapy continues and may be increased. Appropriate precautions should be taken. (See Package Insert of the respective sulfonylurea.)

If patients have not satisfactorily responded to one to three months of concomitant therapy with the maximum dose of metformin hydrochloride and the maximum dose of an oral sulfonylurea, consider therapeutic alternatives including switching to insulin with or without metformin hydrochloride.

**Concomitant Metformin Hydrochloride and Insulin Therapy in Adult Patients**

The current insulin dose should be continued upon initiation of metformin hydrochloride therapy. Metformin hydrochloride therapy should be initiated at 500 mg once daily in patients on insulin therapy. For patients not responding adequately, the dose of metformin hydrochloride should be increased by 500 mg after approximately 1 week and by 500 mg every week thereafter until adequate glycemic control is achieved. The maximum recommended daily dose is 2500 mg for metformin hydrochloride. It is recommended that the insulin dose be decreased by 10% to 25% when fasting plasma glucose concentrations decrease to less than 120 mg/dL in patients receiving concomitant insulin and metformin hydrochloride. Further adjustment should be individualized based on glucose-lowering response.

**Specific Patient Populations**

Metformin hydrochloride is not recommended for use in pregnancy. Metformin hydrochloride is not recommended in patients below the age of 10 years.

The initial and maintenance dosing of metformin hydrochloride should be conservative in patients with advanced age, due to the potential for decreased renal function in this population. Any dosage adjustment should be based on a careful assessment of renal function. Generally, elderly, debilitated, and malnourished patients should not be titrated to the maximum dose of metformin hydrochloride.

Monitoring of renal function is necessary to aid in prevention of lactic acidosis, particularly in the elderly. (See **WARNINGS**.)

**HOW SUPPLIED**

Metformin Hydrochloride Tablets, USP

500 mg - White to off-white, round, biconvex, film coated tablets debossed with G;10 on one side and plain on the other side.

| | |
|---|---|
| Bottles of 100 | **NDC** 67877-561-01 |
| Bottles of 500 | **NDC** 67877-561-05 |
| Bottles of 1000 | **NDC** 67877-561-10 |

850 mg -White to off-white, round, biconvex, film coated tablets debossed with G;11 on one side and plain on the other side.

| | |
|---|---|
| Bottles of 100 | **NDC** 67877-562-01 |
| Bottles of 500 | **NDC** 67877-562-05 |
| Bottles of 1000 | **NDC** 67877-562-10 |

1000 mg - White to off-white, oval, biconvex, scored, film coated tablets debossed with G and 12 on either side of the scoreline on one side and plain on the other side.

| | |
|---|---|
| Bottles of 100 | **NDC** 67877-563-01 |
| Bottles of 500 | **NDC** 67877-563-05 |
| Bottles of 1000 | **NDC** 67877-563-10 |

**Storage**

Store at 20°–25° C (68°–77° F); excursions permitted to 15°–30° C (59°–86° F). [See USP Controlled Room Temperature.]

Dispense in tight, light-resistant containers with child-resistant closure.

Manufactured by:

Granules India Limited,

Hyderabad-500081, INDIA

**MADE IN INDIA**

Distributed by:

**Ascend Laboratories, LLC**

Parsippany, NJ 07054

Toll Free No.: 1-877-272-7901
Nov 2016

**PATIENT INFORMATION**

**Metformin Hydrochloride Tablets USP**

Read this information carefully before you start taking this medicine and each time you refill your prescription. There may be new information. This information does not take the place of your doctor's advice. Ask your doctor or pharmacist if you do not understand some of this information or if you want to know more about this medicine.

**What are Metformin Hydrochloride Tablets USP?**

Metformin Hydrochloride Tablets USP are used to treat type 2 diabetes. This is also known as non-insulin-dependent diabetes mellitus. People with type 2 diabetes are not able to make enough insulin or respond normally to the insulin their bodies make. When this happens, sugar (glucose) builds up in the blood. This can lead to serious medical problems including kidney damage, amputations, and blindness. Diabetes is also closely linked to heart disease. The main goal of treating diabetes is to lower your blood sugar to a normal level.

High blood sugar can be lowered by diet and exercise, by a number of medicines taken by mouth, and by insulin shots. Before you take Metformin Hydrochloride Tablets USP, try to control your diabetes by exercise and weight loss. While you take your diabetes medicine, continue to exercise and follow the diet advised for your diabetes. No matter what your recommended diabetes management plan is, studies have shown that maintaining good blood sugar control can prevent or delay complications of diabetes, such as blindness.

Metformin Hydrochloride Tablets USP can help control your blood sugar in a number of ways. These include helping your body respond better to the insulin it makes naturally, decreasing the amount of sugar your liver makes, and decreasing the amount of sugar your intestines absorb. Metformin Hydrochloride Tablets USP do not cause your body to make more insulin. Because of this, when taken alone, it rarely cause hypoglycemia (low blood sugar), and usually do not cause weight gain. However, when it is taken with a sulfonylurea or with insulin, hypoglycemia is more likely to occur, as is weight gain.

**WARNING: A small number of people who have taken Metformin Hydrochloride Tablets USP have developed a serious condition called lactic acidosis. Lactic acidosis is caused by a buildup of lactic acid in the blood. This happens more often in people with kidney problems. Most people with kidney problems should not take Metformin Hydrochloride Tablets USP. (See "What are the side effects of Metformin Hydrochloride Tablets USP?")**

### Who should not take Metformin Hydrochloride Tablets USP?

Some conditions increase your chance of getting lactic acidosis, or cause other problems if you take either of these medicines. Most of the conditions listed below canincrease your chance of getting lactic acidosis.

**Do not take Metformin Hydrochloride Tablets USP if you:**

• have kidney problems

• have liver problems

• have heart failure that is treated with medicines, such as Lanoxin® (digoxin) or Lasix® (furosemide)

• drink a lot of alcohol. This means you binge drink for short periods or drink all the time

• are seriously dehydrated (have lost a lot of water from your body)

• are going to have an x-ray procedure with injection of dyes (contrast agents)

• are going to have surgery

• develop a serious condition, such as heart attack, severe infection, or a stroke

• are 80 years or older and you have NOT had your kidney function tested

Tell your doctor if you are pregnant or plan to become pregnant. Metformin Hydrochloride Tablets USP may not be right for you. Talk with your doctor about your choices. You should also discuss your choices with your doctor if you are nursing a child.

### Can Metformin Hydrochloride Tablets USP be used in children?

Metformin Hydrochloride Tablets USP have been shown to effectively lower glucose levels in children (ages 10 to 16 years) with type 2 diabetes. Metformin Hydrochloride Tablets USP have not been studied in children younger than 10 years old. Metformin Hydrochloride Tablets USP have not been studied in combination with other oral glucose-control medicines or insulin in children. If you have any questions about the use of Metformin Hydrochloride Tablets USP in children, talk with your doctor or other healthcare provider.

### How should I take Metformin Hydrochloride Tablets USP?

Your doctor will tell you how much medicine to take and when to take it. You will probably start out with a low dose of the medicine. Your doctor may slowly increase your dose until your blood sugar is better controlled. You should take Metformin Hydrochloride Tablets USP with meals.

Your doctor may have you take other medicines along with Metformin Hydrochloride Tablets USP to

control your blood sugar. These medicines may include insulin shots. Taking Metformin Hydrochloride Tablets USP with insulin may help you better control your blood sugar while reducing the insulin dose.

Continue your exercise and diet program and test your blood sugar regularly while taking Metformin Hydrochloride Tablets USP. Your doctor will monitor your diabetes and may perform blood tests on you from time to time to make sure your kidneys and your liver are functioning normally. There is no evidence that Metformin Hydrochloride Tablets USP causes harm to the liver or kidneys.

Tell your doctor if you:

• have an illness that causes severe vomiting, diarrhea or fever, or if you drink a much lower amount of liquid than normal. These conditions can lead to severe dehydration (loss of water in your body). You may need to stop taking Metformin Hydrochloride Tablets USP for a short time.

• plan to have surgery or an x-ray procedure with injection of dye (contrast agent). You may need to stop taking Metformin Hydrochloride Tablets USP for a short time.

• start to take other medicines or change how you take a medicine. Metformin Hydrochloride Tablets USP can affect how well other drugs work, and some drugs can affect how well Metformin Hydrochloride Tablets USP work. Some medicines may cause high blood sugar.

**What should I avoid while taking Metformin Hydrochloride Tablets USP?**

Do not drink a lot of alcoholic drinks while taking Metformin Hydrochloride Tablets USP. This means you should not binge drink for short periods, and you should not drink a lot of alcohol on a regular basis. Alcohol can increase the chance of getting lactic acidosis.

**What are the side effects of Metformin Hydrochloride Tablets USP?**

***Lactic Acidosis*. In rare cases, Metformin Hydrochloride Tablets USP can cause a serious side effect called lactic acidosis. This is caused by a buildup of lactic acid in your blood. This buildup can cause serious damage.** Lactic acidosis caused by Metformin Hydrochloride Tablets USP is rare and has occurred mostly in people whose kidneys were not working normally. Lactic acidosis has been reported in about one in 33,000 patients taking Metformin Hydrochloride Tablets USP over the course of a year. Although rare, if lactic acidosis does occur, it can be fatal in up to half the people who develop it.

It is also important for your liver to be working normally when you take Metformin Hydrochloride Tablets USP. Your liver helps remove lactic acid from your blood.

Make sure you tell your doctor before you use Metformin Hydrochloride Tablets USP if you have kidney or liver problems. You should also **stop using Metformin Hydrochloride Tablets USP and call your doctor right away if you have signs of lactic acidosis. Lactic acidosis is a medical emergency that must be treated in a hospital.**

**Signs of lactic acidosis are:**

• feeling very weak, tired, or uncomfortable

• unusual muscle pain

• trouble breathing

• unusual or unexpected stomach discomfort

• feeling cold

• feeling dizzy or lightheaded

• suddenly developing a slow or irregular heartbeat

If your medical condition suddenly changes, stop taking Metformin Hydrochloride Tablets USP and call your doctor right away. This may be a sign of lactic acidosis or another serious side effect.

*Other Side Effects.* Common side effects of Metformin Hydrochloride Tablets USP include diarrhea, nausea, and upset stomach. These side effects generally go away after you take the medicine for a while. Taking your medicine with meals can help reduce these side effects. Tell your doctor if the side effects bother you a lot, last for more than a few weeks, come back after they've gone away, or start later in therapy. You may need a lower dose or need to stop taking the medicine for a short period or for good.

About 3 out of every 100 people who take Metformin Hydrochloride Tablets USP have an unpleasant metallic taste when they start taking the medicine. It lasts for a short time.

Metformin Hydrochloride Tablets USP rarely cause hypoglycemia (low blood sugar) by themselves. However, hypoglycemia can happen if you do not eat enough, if you drink alcohol, or if you take other medicines to lower blood sugar.

**General advice about prescription medicines**

If you have questions or problems, talk with your doctor or other healthcare provider. You can ask your doctor or pharmacist for the information about Metformin Hydrochloride Tablets USP that is written for healthcare professionals. Medicines are sometimes prescribed for purposes other than those listed in a patient information leaflet. Do not use Metformin Hydrochloride Tablets USP for a condition for which it was not prescribed. Do not share your medicine with other people.

Call your doctor for medical advice about side effects. You may report side effects to FDA at 1-800-FDA-1088.

Manufactured by:

Granules India Limited,

Hyderabad-500081, INDIA

**MADE IN INDIA**

Distributed by:

**Ascend Laboratories, LLC**

Parsippany, NJ 07054

Toll Free No.: 1-877-272-7901

M.L.No.: 37/RR/AP/2003/F/R

P0820016

**PACKAGE LABEL.PRINCIPAL DISPLAY PANEL**

NDC -67877-561-01

Bottle of 100 tablets

Rx Only

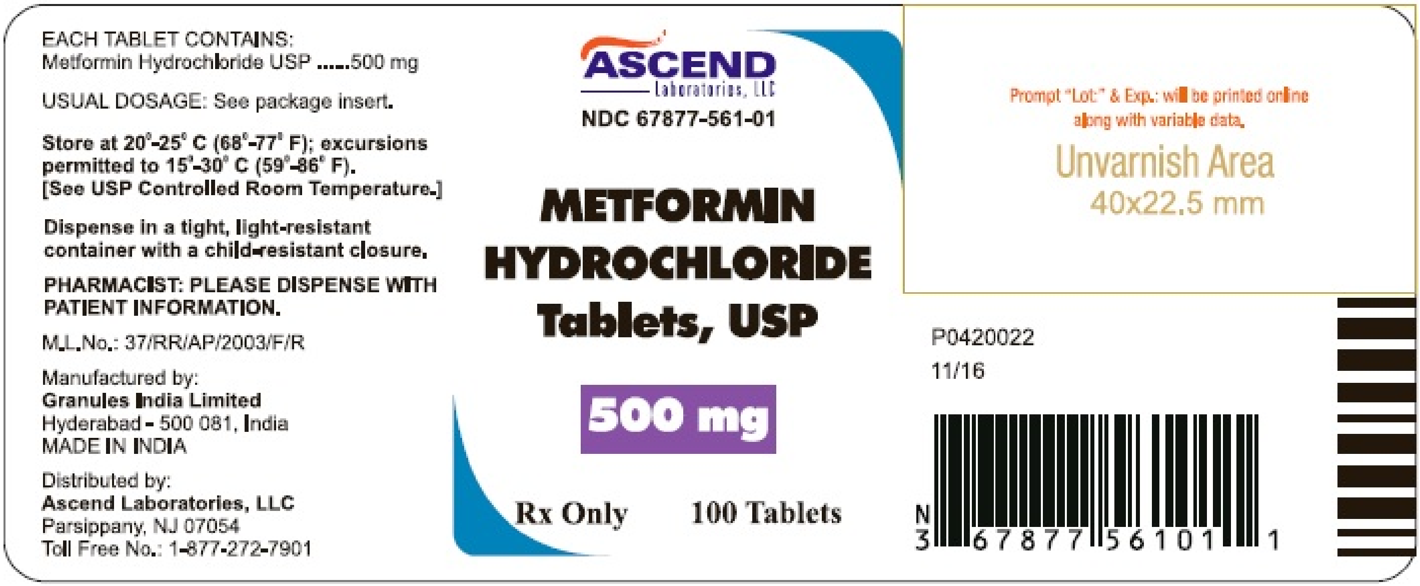


NDC -67877-562-01

Bottle of 100 tablets

Rx Only

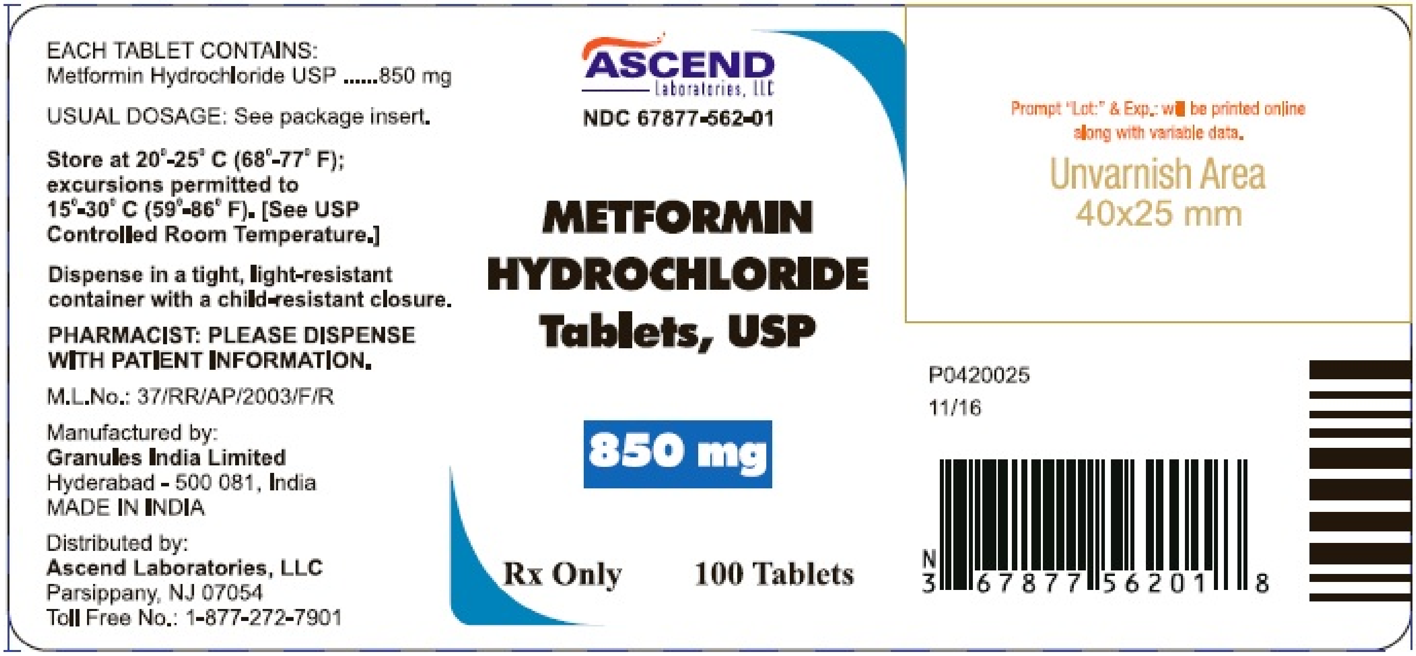


NDC -67877-563-01

Bottle of 100 tablets

Rx Only

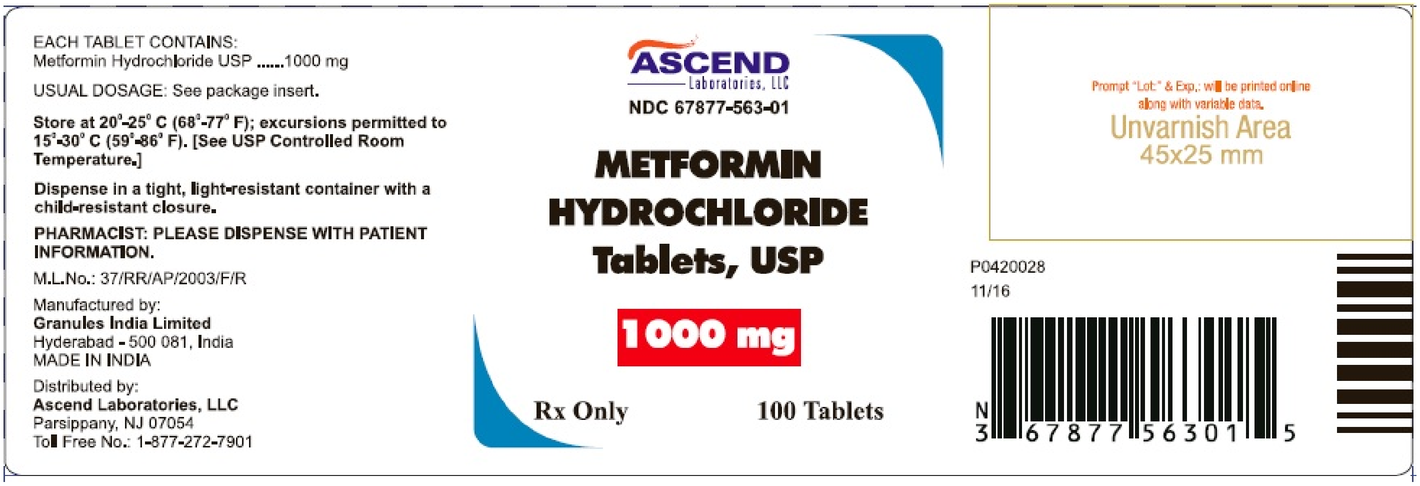


# METFORMIN HCL

metformin hcl tablet

## Product Information

| | | | |
|---|---|---|---|
| **Product Type** | HUMAN PRESCRIPTION DRUG | **Item Code (Source)** | NDC:67877-561 |
| **Route of Administration** | ORAL | | |

## Active Ingredient/Active Moiety

| Ingredient Name | Basis of Strength | Strength |
|---|---|---|
| **METFORMIN HYDROCHLORIDE** (UNII: 786Z46389E) (METFORMIN - UNII:9100L32L2N) | METFORMIN HYDROCHLORIDE | 500 mg |

## Inactive Ingredients

| Ingredient Name | Strength |
|---|---|
| **POVIDONE K30** (UNII: U725QWY32X) | |
| **POVIDONE K90** (UNII: RDH86HJV5Z) | |
| **STARCH, CORN** (UNII: O8232NY3SJ) | |
| **MAGNESIUM STEARATE** (UNII: 70097M6I30) | |
| **HYPROMELLOSE 2910 (6 MPA.S)** (UNII: 0WZ8WG20P6) | |
| **POLYETHYLENE GLYCOL 400** (UNII: B697894SGQ) | |
| **MALTODEXTRIN** (UNII: 7CVR7L4A2D) | |
| **TRIACETIN** (UNII: XHX3C3X673) | |

## Product Characteristics

| | | | |
|---|---|---|---|
| **Color** | WHITE | **Score** | no score |
| **Shape** | ROUND | **Size** | 11mm |
| **Flavor** | BLACKBERRY | **Imprint Code** | G;10 |
| **Contains** | | | |

## Packaging

| # | Item Code | Package Description | Marketing Start Date | Marketing End Date |
|---|---|---|---|---|
| 1 | NDC:67877-561-01 | 100 in 1 BOTTLE; Type 0: Not a Combination Product | 02/06/2017 | |
| 2 | NDC:67877-561-05 | 500 in 1 BOTTLE; Type 0: Not a Combination Product | 02/06/2017 | |
| 3 | NDC:67877-561-10 | 1000 in 1 BOTTLE; Type 0: Not a Combination Product | 02/06/2017 | |

## Marketing Information

| Marketing Category | Application Number or Monograph Citation | Marketing Start Date | Marketing End Date |
|---|---|---|---|
| ANDA | ANDA090564 | 02/06/2017 | |

# METFORMIN HCL

metformin hcl tablet

## Product Information

| | | | |
|---|---|---|---|
| **Product Type** | HUMAN PRESCRIPTION DRUG | **Item Code (Source)** | NDC:67877-562 |
| **Route of Administration** | ORAL | | |

## Active Ingredient/Active Moiety

| Ingredient Name | Basis of Strength | Strength |
|---|---|---|
| **METFORMIN HYDROCHLORIDE** (UNII: 786Z46389E) (METFORMIN - UNII:9100L32L2N) | METFORMIN HYDROCHLORIDE | 850 mg |

## Inactive Ingredients

| Ingredient Name | Strength |
|---|---|
| **POVIDONE K30** (UNII: U725QWY32X) | |
| **POVIDONE K90** (UNII: RDH86HJV5Z) | |
| **STARCH, CORN** (UNII: O8232NY3SJ) | |
| **MAGNESIUM STEARATE** (UNII: 70097M6I30) | |
| **HYPROMELLOSE 2910 (6 MPA.S)** (UNII: 0WZ8WG20P6) | |
| **POLYETHYLENE GLYCOL 400** (UNII: B697894SGQ) | |
| **MALTODEXTRIN** (UNII: 7CVR7L4A2D) | |
| **TRIACETIN** (UNII: XHX3C3X673) | |

## Product Characteristics

| | | | |
|---|---|---|---|
| **Color** | WHITE | **Score** | no score |
| **Shape** | ROUND | **Size** | 13mm |
| **Flavor** | BLACKBERRY | **Imprint Code** | G;11 |
| **Contains** | | | |

## Packaging

| # | Item Code | Package Description | Marketing Start Date | Marketing End Date |
|---|---|---|---|---|
| 1 | NDC:67877-562-01 | 100 in 1 BOTTLE; Type 0: Not a Combination Product | 02/06/2017 | |

| | | | | |
|---|---|---|---|---|
| 2 | NDC:67877-562-05 | 500 in 1 BOTTLE; Type 0: Not a Combination Product | 02/06/2017 | |
| 3 | NDC:67877-562-10 | 1000 in 1 BOTTLE; Type 0: Not a Combination Product | 02/06/2017 | |

## Marketing Information

| Marketing Category | Application Number or Monograph Citation | Marketing Start Date | Marketing End Date |
|---|---|---|---|
| ANDA | ANDA090564 | 02/06/2017 | |

# METFORMIN HCL

metformin hcl tablet

## Product Information

| | | | |
|---|---|---|---|
| **Product Type** | HUMAN PRESCRIPTION DRUG | **Item Code (Source)** | NDC:67877-563 |
| **Route of Administration** | ORAL | | |

## Active Ingredient/Active Moiety

| Ingredient Name | Basis of Strength | Strength |
|---|---|---|
| **METFORMIN HYDROCHLORIDE** (UNII: 786Z46389E) (METFORMIN - UNII:9100L32L2N) | METFORMIN HYDROCHLORIDE | 1000 mg |

## Inactive Ingredients

| Ingredient Name | Strength |
|---|---|
| **POVIDONE K30** (UNII: U725QWY32X) | |
| **POVIDONE K90** (UNII: RDH86HJV5Z) | |
| **STARCH, CORN** (UNII: O8232NY3SJ) | |
| **MAGNESIUM STEARATE** (UNII: 70097M6I30) | |
| **HYPROMELLOSE 2910 (6 MPA.S)** (UNII: 0WZ8WG20P6) | |
| **POLYETHYLENE GLYCOL 400** (UNII: B697894SGQ) | |
| **MALTODEXTRIN** (UNII: 7CVR7L4A2D) | |
| **TRIACETIN** (UNII: XHX3C3X673) | |

## Product Characteristics

| | | | |
|---|---|---|---|
| **Color** | WHITE | **Score** | 2 pieces |
| **Shape** | ROUND | **Size** | 19mm |
| **Flavor** | BLACKBERRY | **Imprint Code** | G;12 |
| **Contains** | | | |

## Packaging

| # | Item Code | Package Description | Marketing Start Date | Marketing End Date |
|---|---|---|---|---|
| 1 | NDC:67877-563-01 | 100 in 1 BOTTLE; Type 0: Not a Combination Product | 02/06/2017 | |
| 2 | NDC:67877-563-05 | 500 in 1 BOTTLE; Type 0: Not a Combination Product | 02/06/2017 | |
| 3 | NDC:67877-563-10 | 1000 in 1 BOTTLE; Type 0: Not a Combination Product | 02/06/2017 | |

## Marketing Information

| Marketing Category | Application Number or Monograph Citation | Marketing Start Date | Marketing End Date |
|---|---|---|---|
| ANDA | ANDA090564 | 02/06/2017 | |

## Labeler - Ascend Laboratories, LLC (141250469)

## Establishment

| Name | Address | ID/FEI | Business Operations |
|---|---|---|---|
| Granules India Limited | | 918609236 | MANUFACTURE(67877-561, 67877-562, 67877-563) |

Revised: 2/2017 Ascend Laboratories, LLC
