# Supplementary material for: A comparison of the content and primary literature support for online medication information provided by Lexicomp and Wikipedia
Source: J Med Libr Assoc. 2018 Jul 1;106(3):352–60. doi: 10.5195/jmla.2018.256 (PMC6013145; doi:10.5195/jmla.2018.256)
Supplement: Appendix E [file jmla-106-352-s005.pdf]

PANTOPRAZOLE SODIUM- pantoprazole tablet, delayed release
Amneal Pharmaceuticals of New York, LLC

HIGHLIGHTS OF PRESCRIBING INFORMATION
These highlights do not include all the information needed to use PANTOPRAZOLE SODIUM DELAYED-RELEASE TABLETS safely and effectively. See full prescribing information for PANTOPRAZOLE SODIUM DELAYED-RELEASE TABLETS.

PANTOPRAZOLE sodium delayed-release tablets USP, for oral use
Initial U.S. Approval: 2000

RECENT MAJOR CHANGES

Dosage and Administration, Recommended Dosing Schedule (2.1) 12/2014
Contraindications (4) 12/2014
Warnings and Precautions, Acute Interstitial Nephritis (5.3) 12/2014

INDICATIONS AND USAGE

Pantoprazole sodium delayed-release tablets, USP are a proton pump inhibitor indicated for the following:

- Short-Term Treatment of Erosive Esophagitis Associated with Gastroesophageal Reflux Disease (GERD) (1.1)
- Maintenance of Healing of Erosive Esophagitis (1.2)
- Pathological Hypersecretory Conditions Including Zollinger-Ellison Syndrome (1.3)

DOSAGE AND ADMINISTRATION

**Indication**	**Dose**	**Frequency**
**Short-Term Treatment of Erosive Esophagitis Associated With GERD (2.1)**		
Adults	40 mg	Once Daily for up to 8 wks
Children (5 years and older)		
≥ 15 kg to < 40 kg	20 mg	Once Daily for up to 8 wks
≥ 40 kg	40 mg	
**Maintenance of Healing of Erosive Esophagitis (2.1)**		
Adults	40 mg	Once Daily*
**Pathological Hypersecretory Conditions Including Zollinger-Ellison Syndrome (2.1)**		
Adults	40 mg	Twice Daily

* Controlled studies did not extend beyond 12 months
See full prescribing information for administration instructions

DOSAGE FORMS AND STRENGTHS

- Delayed-release Tablets, 20 mg and 40 mg (3)

CONTRAINDICATIONS

Known hypersensitivity to any component of the formulation or to substituted benzimidazoles (4)

WARNINGS AND PRECAUTIONS

- Symptomatic response does not preclude presence of gastric malignancy (5.1)
- Atrophic gastritis has been noted with long-term therapy (5.2)
- Acute interstitial nephritis has been observed in patients taking PPIs. (5.3)
- Cyanocobalamin (vitamin B-12) Deficiency: Daily long-term use (e.g., longer than 3 years) may lead to malabsorption or a deficiency of cyanocobalamin. (5.4)
- PPI therapy may be associated with increased risk of *Clostridium difficile* associated diarrhea. (5.5)
- Bone Fracture: Long-term and multiple daily dose PPI therapy may be associated with an increased risk for osteoporosis-related fractures of the hip, wrist or spine. (5.6)
- Hypomagnesemia has been reported rarely with prolonged treatment with PPIs (5.7)

ADVERSE REACTIONS

The most frequently occurring adverse reactions are as follows:

- For adult use (>2%) are headache, diarrhea, nausea, abdominal pain, vomiting, flatulence, dizziness, and arthralgia. (6)
- For pediatric use (>4%) are URI, headache, fever, diarrhea, vomiting, rash, and abdominal pain. (6)

To report SUSPECTED ADVERSE REACTIONS, contact Amneal Pharmaceuticals at 1-877-835-5472 and

www.amneal.com or FDA at 1-800-FDA-1088 or *www.fda.gov/medwatch*.

DRUG INTERACTIONS

- Do not co-administer with atazanavir or nelfinavir (7.1)
- Concomitant warfarin use may require monitoring (7.2)
- May interfere with the absorption of drugs where gastric pH is important for bioavailability (e.g. ketoconazole, ampicillin esters, atazanavir, iron salts, erlotinib and mycophenolate mofetil) (7.4)
- May produce false-positive urine screen for THC (7.5)
- Methotrexate: Pantoprazole sodium may increase serum level of methotrexate (7.6)

See 17 for PATIENT COUNSELING INFORMATION and Medication Guide.

Revised: 11/2015

FULL PRESCRIBING INFORMATION: CONTENTS*

1 INDICATIONS AND USAGE
1.1 Short-Term Treatment of Erosive Esophagitis Associated With Gastroesophageal Reflux Disease (GERD)
1.2 Maintenance of Healing of Erosive Esophagitis
1.3 Pathological Hypersecretory Conditions Including Zollinger-Ellison Syndrome
2 DOSAGE AND ADMINISTRATION
2.1 Recommended Dosing Schedule
2.2 Administration Instructions
3 DOSAGE FORMS AND STRENGTHS
4 CONTRAINDICATIONS
5 WARNINGS AND PRECAUTIONS
5.1 Concurrent Gastric Malignancy
5.2 Atrophic Gastritis
5.3 Acute Interstitial Nephritis
5.4 Cyanocobalamin (Vitamin B-12) Deficiency
5.5 Clostridium difficile associated diarrhea
5.6 Bone Fracture
5.7 Hypomagnesemia
5.8 Tumorigenicity
5.9 Interference with Urine Screen for THC
5.10 Concomitant use of Pantoprazole Sodium with Methotrexate
6 ADVERSE REACTIONS
6.1 Clinical Trial Experience
6.2 Postmarketing Experience
7 DRUG INTERACTIONS
7.1 Interference with Antiretroviral Therapy
7.2 Coumarin Anticoagulants
7.3 Clopidogrel
7.4 Drugs for Which Gastric pH Can Affect Bioavailability
7.5 False Positive Urine Tests for THC
7.6 Methotrexate
8 USE IN SPECIFIC POPULATIONS
8.1 Pregnancy
8.3 Nursing Mothers
8.4 Pediatric Use
8.5 Geriatric Use
8.6 Gender
8.7 Patients with Hepatic Impairment

10 OVERDOSAGE
11 DESCRIPTION
12 CLINICAL PHARMACOLOGY
12.1 Mechanism of Action
12.2 Pharmacodynamics
12.3 Pharmacokinetics
12.4 Pharmacogenomics
13 NONCLINICAL TOXICOLOGY
13.1 Carcinogenesis, Mutagenesis, Impairment of Fertility
13.2 Animal Toxicology and/or Pharmacology
14 CLINICAL STUDIES
14.1 Erosive Esophagitis (EE) Associated with Gastroesophageal Reflux Disease (GERD)
14.2 Long-Term Maintenance of Healing of Erosive Esophagitis
14.3 Pathological Hypersecretory Conditions Including Zollinger-Ellison Syndrome
16 HOW SUPPLIED/STORAGE AND HANDLING
17 PATIENT COUNSELING INFORMATION
* Sections or subsections omitted from the full prescribing information are not listed.

FULL PRESCRIBING INFORMATION

1 INDICATIONS AND USAGE

Pantoprazole sodium delayed-release tablets, USP are indicated for:

1.1 Short-Term Treatment of Erosive Esophagitis Associated With Gastroesophageal Reflux Disease (GERD)

Pantoprazole sodium delayed-release tablets, USP are indicated in adults and pediatric patients five years of age and older for the short-term treatment (up to 8 weeks) in the healing and symptomatic relief of erosive esophagitis. For those adult patients who have not healed after 8 weeks of treatment, an additional 8-week course of pantoprazole sodium delayed-release tablets, USP may be considered. Safety of treatment beyond 8 weeks in pediatric patients has not been established.

1.2 Maintenance of Healing of Erosive Esophagitis

Pantoprazole sodium delayed-release tablets, USP are indicated for maintenance of healing of erosive esophagitis and reduction in relapse rates of daytime and nighttime heartburn symptoms in adult patients with GERD. Controlled studies did not extend beyond 12 months.

1.3 Pathological Hypersecretory Conditions Including Zollinger-Ellison Syndrome

Pantoprazole sodium delayed-release tablets, USP are indicated for the long-term treatment of pathological hypersecretory conditions, including Zollinger-Ellison syndrome.

2 DOSAGE AND ADMINISTRATION

2.1 Recommended Dosing Schedule

Pantoprazole sodium delayed-release tablets, USP are supplied as delayed-release tablets. The recommended dosages are outlined in Table 1.

Table 1: Recommended Dosing Schedule for Pantoprazole Sodium Delayed-Release Tablets,

USP

Indication	Dose	Frequency
**Short-Term Treatment of Erosive Esophagitis Associated With GERD**		
Adults	40 mg	Once daily for up to 8 weeks*
Children (5 years and older)		
≥ 15 kg to < 40 kg	20 mg	Once daily for up to 8 weeks
≥ 40 kg	40 mg	
**Maintenance of Healing of Erosive Esophagitis**		
Adults	40 mg	Once daily***
**Pathological Hypersecretory Conditions Including Zollinger-Ellison Syndrome**		
Adults	40 mg	Twice daily**
* For adult patients who have not healed after 8 weeks of treatment, an additional 8-week course of pantoprazole sodium delayed-release tablets, USP may be considered.		
** Dosage regimens should be adjusted to individual patient needs and should continue for as long as clinically indicated. Doses up to 240 mg daily have been administered.		
*** Controlled studies did not extend beyond 12 months.		

2.2 Administration Instructions

Directions for method of administration are presented in Table 2.

Table 2: Administration Instructions

Formulation	Route	Instructions*
**Delayed-release Tablets**	Oral	Swallowed whole, with or without food
* Patients should be cautioned that pantoprazole sodium delayed-release tablets, USP should not be split, chewed, or crushed.		

Pantoprazole sodium delayed-release tablets, USP

Pantoprazole sodium delayed-release tablets, USP should be swallowed whole, with or without food in the stomach. If patients are unable to swallow a 40 mg tablet, two 20 mg tablets may be taken. Concomitant administration of antacids does not affect the absorption of pantoprazole sodium delayed-release tablets, USP.

3 DOSAGE FORMS AND STRENGTHS

Delayed-release Tablets:

- 20 mg, light yellow to yellow color, oval shaped, biconvex, delayed-release tablets imprinted “A6” with black ink on one side and plain on the other side.
- 40 mg, light yellow to yellow color, oval shaped, biconvex, delayed-release tablets imprinted “A37” with black ink on one side and plain on the other side.

4 CONTRAINDICATIONS

Pantoprazole sodium delayed-release tablets, USP are contraindicated in patients with known hypersensitivity to any component of the formulation or any substituted benzimidazole. Hypersensitivity reactions may include anaphylaxis, anaphylactic shock, angioedema, bronchospasm, acute interstitial nephritis, and urticaria *[see Adverse Reactions (6)]*.

5 WARNINGS AND PRECAUTIONS

5.1 Concurrent Gastric Malignancy

Symptomatic response to therapy with pantoprazole sodium does not preclude the presence of gastric malignancy.

5.2 Atrophic Gastritis

Atrophic gastritis has been noted occasionally in gastric corpus biopsies from patients treated long-term with pantoprazole sodium, particularly in patients who were *H. pylori* positive.

5.3 Acute Interstitial Nephritis

Acute interstitial nephritis has been observed in patients taking PPIs including pantoprazole sodium. Acute interstitial nephritis may occur at any point during PPI therapy and is generally attributed to an idiopathic hypersensitivity reaction. Discontinue pantoprazole sodium if acute interstitial nephritis develops *[see Contraindications (4)]*.

5.4 Cyanocobalamin (Vitamin B-12) Deficiency

Generally, daily treatment with any acid-suppressing medications over a long period of time (e.g., longer than 3 years) may lead to malabsorption of cyanocobalamin (Vitamin B-12) caused by hypo- or achlorhydria. Rare reports of cyanocobalamin deficiency occurring with acid-suppressing therapy have been reported in the literature. This diagnosis should be considered if clinical symptoms consistent with cyanocobalamin deficiency are observed.

5.5 Clostridium difficile associated diarrhea

Published observational studies suggest that PPI therapy like pantoprazole sodium may be associated with an increased risk of *Clostridium difficile* associated diarrhea, especially in hospitalized patients. This diagnosis should be considered for diarrhea that does not improve *[see Adverse Reactions (6.2)]*

Patients should use the lowest dose and shortest duration of PPI therapy appropriate to the condition being treated.

5.6 Bone Fracture

Several published observational studies suggest that proton pump inhibitor (PPI) therapy may be associated with an increased risk for osteoporosis-related fractures of the hip, wrist, or spine. The risk of fracture was increased in patients who received high-dose, defined as multiple daily doses, and long-term PPI therapy (a year or longer). Patients should use the lowest dose and shortest duration of PPI therapy appropriate to the condition being treated. Patients at risk for osteoporosis-related fractures should be managed according to established treatment guidelines *[see Dosage and Administration (2) and Adverse Reactions (6.2)]*.

5.7 Hypomagnesemia

Hypomagnesemia, symptomatic and asymptomatic, has been reported rarely in patients treated with PPIs for at least three months, in most cases after a year of therapy. Serious adverse events include tetany, arrhythmias, and seizures. In most patients, treatment of hypomagnesemia required magnesium replacement and discontinuation of the PPI.

For patients expected to be on prolonged treatment or who take PPIs with medications such as digoxin or drugs that may cause hypomagnesemia (e.g., diuretics), health care professionals may consider monitoring magnesium levels prior to initiation of PPI treatment and periodically *[see Adverse Reactions (6.2)]*.

5.8 Tumorigenicity

Due to the chronic nature of GERD, there may be a potential for prolonged administration of

pantoprazole sodium. In long-term rodent studies, pantoprazole was carcinogenic and caused rare types of gastrointestinal tumors. The relevance of these findings to tumor development in humans is unknown *[see Nonclinical Toxicology (13.1)].*

5.9 Interference with Urine Screen for THC

See Drug Interactions (7.5).

5.10 Concomitant use of Pantoprazole Sodium with Methotrexate

Literature suggests that concomitant use of PPIs with methotrexate (primarily at high dose; see methotrexate prescribing information) may elevate and prolong serum levels of methotrexate and/or its metabolite, possibly leading to methotrexate toxicities. In high-dose methotrexate administration, a temporary withdrawal of the PPI may be considered in some patients *[see Drug Interactions (7.6)].*

6 ADVERSE REACTIONS

6.1 Clinical Trial Experience

Because clinical trials are conducted under widely varying conditions, adverse reaction rates observed in the clinical trials of a drug cannot be directly compared to rates in the clinical trials of another drug and may not reflect the rates observed in clinical practice.

Adults

Safety in nine randomized comparative US clinical trials in patients with GERD included 1,473 patients on oral pantoprazole sodium (20 mg or 40 mg), 299 patients on an H_2-receptor antagonist, 46 patients on another proton pump inhibitor, and 82 patients on placebo. The most frequently occurring adverse reactions are listed in Table 3.

Table 3: Adverse Reactions Reported in Clinical Trials of Adult Patients with GERD at a Frequency of > 2%

	Pantoprazole Sodium (n=1473) %	Comparators (n=345) %	Placebo (n=82) %
Headache	12.2	12.8	8.5
Diarrhea	8.8	9.6	4.9
Nausea	7	5.2	9.8
Abdominal pain	6.2	4.1	6.1
Vomiting	4.3	3.5	2.4
Flatulence	3.9	2.9	3.7
Dizziness	3	2.9	1.2
Arthralgia	2.8	1.4	1.2

Additional adverse reactions that were reported for pantoprazole sodium in clinical trials with a frequency of ≤ 2% are listed below by body system:

<u>Body as a Whole:</u> allergic reaction, pyrexia, photosensitivity reaction, facial edema

<u>Gastrointestinal:</u> constipation, dry mouth, hepatitis

<u>Hematologic:</u> leukopenia, thrombocytopenia

<u>Metabolic/Nutritional:</u> elevated CK (creatine kinase), generalized edema, elevated triglycerides, liver enzymes elevated

Musculoskeletal: myalgia

Nervous: depression, vertigo

Skin and Appendages: urticaria, rash, pruritus

Special Senses: blurred vision

Pediatric Patients

Safety of pantoprazole sodium in the treatment of Erosive Esophagitis (EE) associated with GERD was evaluated in pediatric patients ages 1 year through 16 years in three clinical trials. Safety trials involved pediatric patients with EE; however, as EE is uncommon in the pediatric population, 249 pediatric patients with endoscopically-proven or symptomatic GERD were also evaluated. All adult adverse reactions to pantoprazole sodium are considered relevant to pediatric patients. In patients ages 1 year through 16 years, the most commonly reported (> 4%) adverse reactions include: URI, headache, fever, diarrhea, vomiting, rash, and abdominal pain.

For safety information in patients less than 1 year of age see *Use in Specific Populations (8.4)*.

Additional adverse reactions that were reported for pantoprazole sodium in pediatric patients in clinical trials with a frequency of ≤ 4% are listed below by body system:

Body as a Whole: allergic reaction, facial edema

Gastrointestinal: constipation, flatulence, nausea

Metabolic/Nutritional: elevated triglycerides, elevated liver enzymes, elevated CK (creatine

kinase)

Musculoskeletal: arthralgia, myalgia

Nervous: dizziness, vertigo

Skin and Appendages: urticaria

The following adverse reactions seen in adults in clinical trials were not reported in pediatric patients in clinical trials, but are considered relevant to pediatric patients: photosensitivity reaction, dry mouth, hepatitis, thrombocytopenia, generalized edema, depression, pruritus, leukopenia, and blurred vision.

Zollinger-Ellison Syndrome

In clinical studies of Zollinger-Ellison Syndrome, adverse reactions reported in 35 patients taking pantoprazole sodium 80 mg/day to 240 mg/day for up to 2 years were similar to those reported in adult patients with GERD.

6.2 Postmarketing Experience

The following adverse reactions have been identified during postapproval use of pantoprazole sodium. Because these reactions are reported voluntarily from a population of uncertain size, it is not always possible to reliably estimate their frequency or establish a causal relationship to drug exposure.

These adverse reactions are listed below by body system:

General Disorders and Administration Conditions: asthenia, fatigue, malaise

Hematologic: pancytopenia, agranulocytosis

Hepatobiliary Disorders: hepatocellular damage leading to jaundice and hepatic failure

Immune System Disorders: anaphylaxis (including anaphylactic shock)

Infections and Infestations: *Clostridium difficile* associated diarrhea

Investigations: weight changes

Metabolism and Nutritional Disorders: hyponatremia, hypomagnesemia

Musculoskeletal Disorders: rhabdomyolysis, bone fracture

Nervous: ageusia, dysgeusia

Psychiatric Disorders: hallucination, confusion, insomnia, somnolence

Renal and Urinary Disorders: interstitial nephritis

Skin and Subcutaneous Tissue Disorders: severe dermatologic reactions (some fatal), including erythema multiforme, Stevens-Johnson syndrome, and toxic epidermal necrolysis (TEN, some fatal), and angioedema (Quincke's edema)

7 DRUG INTERACTIONS

7.1 Interference with Antiretroviral Therapy

Concomitant use of atazanavir or nelfinavir with proton pump inhibitors is not recommended. Co-administration of atazanavir or nelfinavir with proton pump inhibitors is expected to substantially decrease atazanavir or nelfinavir plasma concentrations and may result in a loss of therapeutic effect and development of drug resistance.

7.2 Coumarin Anticoagulants

There have been postmarketing reports of increased INR and prothrombin time in patients receiving proton pump inhibitors, including pantoprazole sodium, and warfarin concomitantly. Increases in INR and prothrombin time may lead to abnormal bleeding and even death. Patients treated with proton pump inhibitors and warfarin concomitantly should be monitored for increases in INR and prothrombin time.

7.3 Clopidogrel

Concomitant administration of pantoprazole and clopidogrel in healthy subjects had no clinically important effect on exposure to the active metabolite of clopidogrel or clopidogrel-induced platelet inhibition *[see Clinical Pharmacology (12.3)]*. No dose adjustment of clopidogrel is necessary when administered with an approved dose of pantoprazole sodium.

7.4 Drugs for Which Gastric pH Can Affect Bioavailability

Due to its effects on gastric acid secretion, pantoprazole can reduce the absorption of drugs where gastric pH is an important determinant of their bioavailability. Like with other drugs that decrease the intragastric acidity, the absorption of drugs such as ketoconazole, ampicillin esters, atazanavir, iron salts, erlotinib, and mycophenolate mofetil (MMF) can decrease.

Co-administration of pantoprazole in healthy subjects and in transplant patients receiving MMF has been reported to reduce the exposure to the active metabolite, mycophenolic acid (MPA), possibly due to a decrease in MMF solubility at an increased gastric pH. The clinical relevance of reduced MPA exposure on organ rejection has not been established in transplant patients receiving pantoprazole sodium and MMF. Use pantoprazole sodium with caution in transplant patients receiving MMF *[see Clinical Pharmacology (12.3)]*.

7.5 False Positive Urine Tests for THC

There have been reports of false positive urine screening tests for tetrahydrocannabinol (THC) in patients receiving proton pump inhibitors. An alternative confirmatory method should be considered to verify positive results.

7.6 Methotrexate

Case reports, published population pharmacokinetic studies, and retrospective analyses suggest that

concomitant administration of PPIs and methotrexate (primarily at high dose; see methotrexate prescribing information) may elevate and prolong serum levels of methotrexate and/or its metabolite hydroxymethotrexate. However, no formal drug interaction studies of Methotrexate with PPIs have been conducted *[see Warnings and Precautions (5.10)]*.

8 USE IN SPECIFIC POPULATIONS

8.1 Pregnancy

Teratogenic Effects

Pregnancy Category B

Reproduction studies have been performed in rats at oral doses up to 88 times the recommended human dose and in rabbits at oral doses up to 16 times the recommended human dose and have revealed no evidence of impaired fertility or harm to the fetus due to pantoprazole. There are, however, no adequate and well-controlled studies in pregnant women. Because animal reproduction studies are not always predictive of human response, this drug should be used during pregnancy only if clearly needed *[see Nonclinical Toxicology (13.2)]*.

8.3 Nursing Mothers

Pantoprazole and its metabolites are excreted in the milk of rats. Pantoprazole excretion in human milk has been detected in a study of a single nursing mother after a single 40 mg oral dose. The clinical relevance of this finding is not known. Many drugs which are excreted in human milk have a potential for serious adverse reactions in nursing infants. Based on the potential for tumorigenicity shown for pantoprazole in rodent carcinogenicity studies, a decision should be made whether to discontinue nursing or to discontinue the drug, taking into account the benefit of the drug to the mother.

8.4 Pediatric Use

The safety and effectiveness of pantoprazole sodium for short-term treatment (up to eight weeks) of erosive esophagitis (EE) associated with GERD have been established in pediatric patients 1 year through 16 years of age. Effectiveness for EE has not been demonstrated in patients less than 1 year of age. In addition, for patients less than 5 years of age, there is no appropriate dosage strength in an age-appropriate formulation available. Therefore, pantoprazole sodium is indicated for the short-term treatment of EE associated with GERD for patients 5 years and older. The safety and effectiveness of pantoprazole sodium for pediatric uses other than EE have not been established.

1 year through 16 years of age

Use of pantoprazole sodium in pediatric patients 1 year through 16 years of age for short-term treatment (up to eight weeks) of EE associated with GERD is supported by: a) extrapolation of results from adequate and well-controlled studies that supported the approval of pantoprazole sodium for treatment of EE associated with GERD in adults, and b) safety, effectiveness, and pharmacokinetic studies performed in pediatric patients *[see Clinical Studies (14.1), and Clinical Pharmacology (12.3)]*.

Safety of pantoprazole sodium in the treatment of EE associated with GERD in pediatric patients 1 through 16 years of age was evaluated in three multicenter, randomized, double-blind, parallel-treatment studies, involving 249 pediatric patients, including 8 with EE (4 patients ages 1 year to 5 years and 4 patients 5 years to 11 years). The children ages 1 year to 5 years with endoscopically diagnosed EE (defined as an endoscopic Hetzel-Dent score ≥ 2) were treated once daily for 8 weeks with one of two dose levels of pantoprazole sodium (approximating 0.6 mg/kg or 1.2 mg/kg). All 4 of these patients with EE were healed (Hetzel-Dent score of 0 or 1) at 8 weeks. Because EE is uncommon in the pediatric population, predominantly pediatric patients with endoscopically-proven or symptomatic GERD were also included in these studies. Patients were treated with a range of doses of pantoprazole sodium once daily for 8 weeks. For safety findings see *Adverse Reactions (6.1)*. Because these pediatric trials had no

placebo, active comparator, or evidence of a dose response, the trials were inconclusive regarding the clinical benefit of pantoprazole sodium for symptomatic GERD in the pediatric population. The effectiveness of pantoprazole sodium for treating symptomatic GERD in pediatric patients has not been established.

Although the data from the clinical trials support use of pantoprazole sodium for the short-term treatment of EE associated with GERD in pediatric patients 1 year through 5 years, there is no commercially available dosage formulation appropriate for patients less than 5 years of age *[see Dosage and Administration (2)]*.

In a population pharmacokinetic analysis, clearance values in the children 1 to 5 years old with endoscopically proven GERD had a median value of 2.4 L/h. Following a 1.2 mg/kg equivalent dose (15 mg for ≤ 12.5 kg and 20 mg for > 12.5 to < 25 kg), the plasma concentrations of pantoprazole were highly variable and the median time to peak plasma concentration was 3 to 6 hours. The estimated AUC for patients 1 to 5 years old was 37% higher than for adults receiving a single 40 mg tablet, with a geometric mean AUC value of 6.8 mcg•hr/mL.

Neonates to less than one year of age

Pantoprazole sodium was not found to be effective in a multicenter, randomized, double-blind, placebo-controlled, treatment-withdrawal study of 129 pediatric patients 1 through 11 months of age. Patients were enrolled if they had symptomatic GERD based on medical history and had not responded to non-pharmacologic interventions for GERD for two weeks. Patients received pantoprazole sodium daily for four weeks in an open-label phase, then patients were randomized in equal proportion to receive pantoprazole sodium treatment or placebo for the subsequent four weeks in a double-blind manner. Efficacy was assessed by observing the time from randomization to study discontinuation due to symptom worsening during the four-week treatment-withdrawal phase. There was no statistically significant difference between pantoprazole sodium and placebo in the rate of discontinuation.

In this trial, the adverse reactions that were reported more commonly (difference of ≥ 4%) in the treated population compared to the placebo population were elevated CK, otitis media, rhinitis, and laryngitis.

In a population pharmacokinetic analysis, the systemic exposure was higher in patients less than 1 year of age with GERD compared to adults who received a single 40 mg dose (geometric mean AUC was 103% higher in preterm infants and neonates receiving single dose of 2.5 mg of pantoprazole sodium, and 23% higher in infants 1 through 11 months of age receiving a single dose of approximately 1.2 mg/kg). In these patients, the apparent clearance (CL/F) increased with age (median clearance: 0.6 L/hr, range: 0.03 to 3.2 L/hr).

These doses resulted in pharmacodynamic effects on gastric but not esophageal pH. Following once daily dosing of 2.5 mg of pantoprazole sodium in preterm infants and neonates, there was an increase in the mean gastric pH (from 4.3 at baseline to 5.2 at steady-state) and in the mean % time that gastric pH was > 4 (from 60% at baseline to 80% at steady-state). Following once daily dosing of approximately 1.2 mg/kg of pantoprazole sodium in infants 1 through 11 months of age, there was an increase in the mean gastric pH (from 3.1 at baseline to 4.2 at steady-state) and in the mean % time that gastric pH was > 4 (from 32% at baseline to 60% at steady-state). However, no significant changes were observed in mean intraesophageal pH or % time that esophageal pH was < 4 in either age group.

Because pantoprazole sodium was not shown to be effective in the randomized, placebo-controlled study in this age group, the use of pantoprazole sodium for treatment of symptomatic GERD in infants less than 1 year of age is not indicated.

8.5 Geriatric Use

In short-term US clinical trials, erosive esophagitis healing rates in the 107 elderly patients (≥ 65 years old) treated with pantoprazole sodium were similar to those found in patients under the age of 65. The incidence rates of adverse reactions and laboratory abnormalities in patients aged 65 years and older were similar to those associated with patients younger than 65 years of age.

8.6 Gender

Erosive esophagitis healing rates in the 221 women treated with pantoprazole sodium delayed-release tablets in US clinical trials were similar to those found in men. In the 122 women treated long-term with pantoprazole sodium 40 mg or 20 mg, healing was maintained at a rate similar to that in men. The incidence rates of adverse reactions were also similar for men and women.

8.7 Patients with Hepatic Impairment

Doses higher than 40 mg/day have not been studied in patients with hepatic impairment *[see Clinical Pharmacology (12.3)]*.

10 OVERDOSAGE

Experience in patients taking very high doses of pantoprazole sodium (> 240 mg) is limited. Spontaneous post-marketing reports of overdose are generally within the known safety profile of pantoprazole sodium.

Pantoprazole is not removed by hemodialysis. In case of overdosage, treatment should be symptomatic and supportive.

Single oral doses of pantoprazole at 709 mg/kg, 798 mg/kg, and 887 mg/kg were lethal to mice, rats, and dogs, respectively. The symptoms of acute toxicity were hypoactivity, ataxia, hunched sitting, limb-splay, lateral position, segregation, absence of ear reflex, and tremor.

11 DESCRIPTION

The active ingredient in pantoprazole sodium delayed-release tablets, USP is a substituted benzimidazole, sodium 5-(difluoromethoxy)-2-[[(3,4-dimethoxy-2-pyridinyl)methyl] sulfinyl]-1*H*-benzimidazole sesquihydrate, a compound that inhibits gastric acid secretion. Its empirical formula is $C_{16}H_{14}F_2N_3NaO_4S$ x 1.5 H_2O, with a molecular weight of 432.4. The structural formula is:

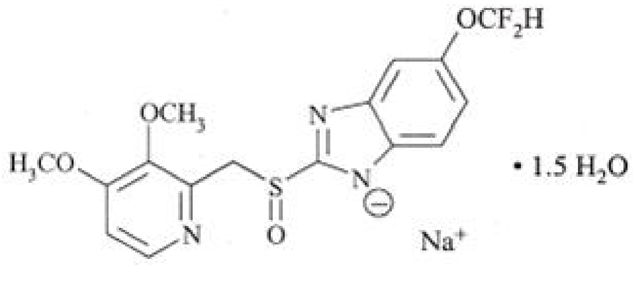

Pantoprazole sodium, USP sesquihydrate is a white to off-white crystalline powder and is racemic. Pantoprazole has weakly basic and acidic properties. Pantoprazole sodium, USP sesquihydrate is freely soluble in water, very slightly soluble in phosphate buffer at pH 7.4, and practically insoluble in n-hexane.

The stability of the compound in aqueous solution is pH-dependent. The rate of degradation increases with decreasing pH. At ambient temperature, the degradation half-life is approximately 2.8 hours at pH 5 and approximately 220 hours at pH 7.8.

Pantoprazole sodium, USP is supplied as a delayed-release tablet, available in two strengths (20 mg and 40 mg).

Each pantoprazole sodium delayed-release tablet, USP contains 45.1 mg or 22.56 mg of pantoprazole sodium, USP sesquihydrate (equivalent to 40 mg or 20 mg pantoprazole, respectively) with the following inactive ingredients: ammonium hydroxide, crospovidone, hydroxyl propyl cellulose,

hypromellose, iron oxide black, iron oxide yellow, mannitol, methacrylic acid copolymer dispersion, polyethylene glycol, polyvinyl alcohol, povidone, propylene glycol, shellac, sodium carbonate, sodium hydroxide, sodium stearyl fumarate, talc, titanium dioxide and triethyl citrate. Pantoprazole sodium delayed-release tablets, USP (40 mg and 20 mg) complies with USP dissolution test 2.

12 CLINICAL PHARMACOLOGY

12.1 Mechanism of Action

Pantoprazole is a proton pump inhibitor (PPI) that suppresses the final step in gastric acid production by covalently binding to the (H^+, K^+)-ATPase enzyme system at the secretory surface of the gastric parietal cell. This effect leads to inhibition of both basal and stimulated gastric acid secretion, irrespective of the stimulus. The binding to the (H^+, K^+)-ATPase results in a duration of antisecretory effect that persists longer than 24 hours for all doses tested (20 mg to 120 mg).

12.2 Pharmacodynamics

Pantoprazole sodium for delayed-release oral suspension, 40 mg has been shown to be comparable to pantoprazole sodium delayed-release tablets in suppressing pentagastrin-stimulated MAO in patients (n = 49) with GERD and a history of EE. In this multicenter, pharmacodynamic crossover study, a 40 mg oral dose of pantoprazole sodium for delayed-release oral suspension administered in a teaspoonful of applesauce was compared with a 40 mg oral dose of pantoprazole sodium delayed-release tablets after administration of each formulation once daily for 7 days. Both medications were administered thirty minutes before breakfast. Pentagastrin-stimulated (MAO) was assessed from hour 23 to 24 at steady-state.

Antisecretory Activity

Under maximal acid stimulatory conditions using pentagastrin, a dose-dependent decrease in gastric acid output occurs after a single dose of oral (20 to 80 mg) or a single dose of intravenous (20 to 120 mg) pantoprazole in healthy volunteers. Pantoprazole given once daily results in increasing inhibition of gastric acid secretion. Following the initial oral dose of 40 mg pantoprazole, a 51% mean inhibition was achieved by 2.5 hours. With once-a-day dosing for 7 days, the mean inhibition was increased to 85%. Pantoprazole suppressed acid secretion in excess of 95% in half of the subjects. Acid secretion had returned to normal within a week after the last dose of pantoprazole; there was no evidence of rebound hypersecretion.

In a series of dose-response studies, pantoprazole, at oral doses ranging from 20 to 120 mg, caused dose-related increases in median basal gastric pH and in the percent of time gastric pH was > 3 and > 4. Treatment with 40 mg of pantoprazole produced significantly greater increases in gastric pH than the 20 mg dose. Doses higher than 40 mg (60, 80, 120 mg) did not result in further significant increases in median gastric pH. The effects of pantoprazole on median pH from one double-blind crossover study are shown in Table 4.

Table 4: Effect of Single Daily Doses of Oral Pantoprazole on Intragastric pH

	————————Median pH on day 7 ———————			
Time	Placebo	20 mg	40 mg	80 mg
8 a.m. to 8 a.m.				
(24 hours)	1.3	2.9*	3.8*#	3.9*#
8 a.m. to 10 p.m.				
(Daytime)	1.6	3.2*	4.4*#	4.8*#

10 p.m. to 8 a.m.				
(Nighttime)	1.2	2.1*	3*	2.6*
* Significantly different from placebo				
# Significantly different from 20 mg				

Serum Gastrin Effects

Fasting serum gastrin levels were assessed in two double-blind studies of the acute healing of erosive esophagitis (EE) in which 682 patients with gastroesophageal reflux disease (GERD) received 10, 20, or 40 mg of pantoprazole sodium for up to 8 weeks. At 4 weeks of treatment there was an increase in mean gastrin levels of 7%, 35%, and 72% over pretreatment values in the 10, 20, and 40 mg treatment groups, respectively. A similar increase in serum gastrin levels was noted at the 8-week visit with mean increases of 3%, 26%, and 84% for the three pantoprazole dose groups. Median serum gastrin levels remained within normal limits during maintenance therapy with pantoprazole sodium delayed-release tablets.

In long-term international studies involving over 800 patients, a 2- to 3-fold mean increase from the pretreatment fasting serum gastrin level was observed in the initial months of treatment with pantoprazole at doses of 40 mg per day during GERD maintenance studies and 40 mg or higher per day in patients with refractory GERD. Fasting serum gastrin levels generally remained at approximately 2 to 3 times baseline for up to 4 years of periodic follow-up in clinical trials.

Following short-term treatment with pantoprazole sodium, elevated gastrin levels return to normal by at least 3 months.

Enterochromaffin-Like (ECL) Cell Effects

In 39 patients treated with oral pantoprazole 40 mg to 240 mg daily (majority receiving 40 mg to 80 mg) for up to 5 years, there was a moderate increase in ECL-cell density, starting after the first year of use, which appeared to plateau after 4 years.

In a nonclinical study in Sprague-Dawley rats, lifetime exposure (24 months) to pantoprazole at doses of 0.5 to 200 mg/kg/day resulted in dose-related increases in gastric ECL cell proliferation and gastric neuroendocrine (NE)-cell tumors. Gastric NE-cell tumors in rats may result from chronic elevation of serum gastrin concentrations. The high density of ECL cells in the rat stomach makes this species highly susceptible to the proliferative effects of elevated gastrin concentrations produced by proton pump inhibitors. However, there were no observed elevations in serum gastrin following the administration of pantoprazole at a dose of 0.5 mg/kg/day. In a separate study, a gastric NE-cell tumor without concomitant ECL-cell proliferative changes was observed in 1 female rat following 12 months of dosing with pantoprazole at 5 mg/kg/day and a 9 month off-dose recovery *[see Nonclinical Toxicology (13.1)]*.

12.3 Pharmacokinetics

Pantoprazole sodium delayed-release tablets are prepared as enteric-coated tablets so that absorption of pantoprazole begins only after the tablet leaves the stomach. Peak serum concentration (C_{max}) and area under the serum concentration time curve (AUC) increase in a manner proportional to oral and intravenous doses from 10 mg to 80 mg. Pantoprazole does not accumulate, and its pharmacokinetics are unaltered with multiple daily dosing. Following oral or intravenous administration, the serum concentration of pantoprazole declines biexponentially, with a terminal elimination half-life of approximately one hour.

In extensive metabolizers with normal liver function receiving an oral dose of the enteric-coated 40 mg pantoprazole tablet, the peak concentration (C_{max}) is 2.5 mcg/mL; the time to reach the peak concentration (t_{max}) is 2.5 h, and the mean total area under the plasma concentration versus time curve (AUC) is 4.8 mcg•h/mL (range 1.4 to 13.3 mcg•h/mL). Following intravenous administration of pantoprazole to extensive metabolizers, its total clearance is 7.6 to 14 L/h, and its apparent volume of

distribution is 11 to 23.6 L.

Absorption

After administration of a single or multiple oral 40 mg doses of pantoprazole sodium delayed-release tablets, the peak plasma concentration of pantoprazole was achieved in approximately 2.5 hours, and C_{max} was 2.5 mcg/mL. Pantoprazole undergoes little first-pass metabolism, resulting in an absolute bioavailability of approximately 77%. Pantoprazole absorption is not affected by concomitant administration of antacids.

Administration of pantoprazole sodium delayed-release tablets with food may delay its absorption up to 2 hours or longer; however, the C_{max} and the extent of pantoprazole absorption (AUC) are not altered. Thus, pantoprazole sodium delayed-release tablets may be taken without regard to timing of meals.

Distribution

The apparent volume of distribution of pantoprazole is approximately 11 to 23.6 L, distributing mainly in extracellular fluid. The serum protein binding of pantoprazole is about 98%, primarily to albumin.

Metabolism

Pantoprazole is extensively metabolized in the liver through the cytochrome P450 (CYP) system. Pantoprazole metabolism is independent of the route of administration (intravenous or oral). The main metabolic pathway is demethylation, by CYP2C19, with subsequent sulfation; other metabolic pathways include oxidation by CYP3A4. There is no evidence that any of the pantoprazole metabolites have significant pharmacologic activity.

Elimination

After a single oral or intravenous dose of ^{14}C-labeled pantoprazole to healthy, normal metabolizer volunteers, approximately 71% of the dose was excreted in the urine, with 18% excreted in the feces through biliary excretion. There was no renal excretion of unchanged pantoprazole.

Geriatric

Only slight to moderate increases in pantoprazole AUC (43%) and C_{max} (26%) were found in elderly volunteers (64 to 76 years of age) after repeated oral administration, compared with younger subjects. No dosage adjustment is recommended based on age.

Pediatric

The pharmacokinetics of pantoprazole were studied in children less than 16 years of age in four randomized, open-label clinical trials in pediatric patients with presumed/proven GERD. A pediatric granule formulation was studied in children through 5 years of age, and pantoprazole sodium delayed-release tablets were studied in children older than 5 years.

In a population PK analysis, total clearance increased with increasing bodyweight in a non-linear fashion. The total clearance also increased with increasing age only in children under 3 years of age.

Neonate through 5 years of age

See Use in Specific Populations (8.4).

Children and Adolescents 6 through 16 Years of Age

The pharmacokinetics of pantoprazole sodium delayed-release tablets were evaluated in children ages 6 through 16 years with a clinical diagnosis of GERD. The PK parameters following a single oral dose of 20 mg or 40 mg of pantoprazole sodium delayed-release tablets in children ages 6 through 16 years were highly variable (%CV ranges 40% to 80%). The geometric mean AUC estimated from population PK analysis after a 40 mg pantoprazole sodium delayed-release tablet in pediatric patients was about 39% and 10% higher respectively in 6 to 11 and 12 to 16 year-old children, compared to that of adults (Table 6).

Table 6: PK Parameters in Children and Adolescents 6 through 16 years with GERD receiving 40 mg Pantoprazole Sodium Delayed-release Tablets

	6 to 11 years (n=12)	12 to 16 years (n=11)
C_{max} (mcg/mL)[a]	1.8	1.8
t_{max} (h)[b]	2	2
AUC (mcg•h/mL)[a]	6.9	5.5
CL/F (L/h)[b]	6.6	6.8
[a] Geometric mean values		
[b] Median values		

Gender

There is a modest increase in pantoprazole AUC and C_{max} in women compared to men. However, weight-normalized clearance values are similar in women and men. No dosage adjustment is recommended based on gender. In pediatric patients ages 1 through 16 years there were no clinically relevant effects of gender on clearance of pantoprazole, as shown by population pharmacokinetic analysis.

Renal Impairment

In patients with severe renal impairment, pharmacokinetic parameters for pantoprazole were similar to those of healthy subjects. No dosage adjustment is necessary in patients with renal impairment or in patients undergoing hemodialysis.

Hepatic Impairment

In patients with mild to severe hepatic impairment (Child-Pugh A to C cirrhosis), maximum pantoprazole concentrations increased only slightly (1.5-fold) relative to healthy subjects. Although serum half-life values increased to 7 to 9 hours and AUC values increased by 5- to 7-fold in hepatic-impaired patients, these increases were no greater than those observed in CYP2C19 poor metabolizers, where no dosage adjustment is warranted. These pharmacokinetic changes in hepatic-impaired patients result in minimal drug accumulation following once-daily, multiple-dose administration. No dosage adjustment is needed in patients with mild to severe hepatic impairment. Doses higher than 40 mg/day have not been studied in hepatically impaired patients.

Drug-Drug Interactions

Pantoprazole is metabolized mainly by CYP2C19 and to minor extents by CYPs 3A4, 2D6, and 2C9. In *in vivo* drug-drug interaction studies with CYP2C19 substrates (diazepam [also a CYP3A4 substrate] and phenytoin [also a CYP3A4 inducer] and clopidogrel), nifedipine, midazolam, and clarithromycin (CYP3A4 substrates), metoprolol (a CYP2D6 substrate), diclofenac, naproxen and piroxicam (CYP2C9 substrates), and theophylline (a CYP1A2 substrate) in healthy subjects, the pharmacokinetics of pantoprazole were not significantly altered.

Clopidogrel: Clopidogrel is metabolized to its active metabolite in part by CYP2C19. In a crossover clinical study, 66 healthy subjects were administered clopidogrel (300 mg loading dose followed by 75 mg per day) alone and with pantoprazole (80 mg at the same time as clopidogrel) for 5 days. On Day 5, the mean AUC of the active metabolite of clopidogrel was reduced by approximately 14% (geometric mean ratio was 86%, with 90% CI of 79 to 93%) when pantoprazole was co-administered with clopidogrel as compared to clopidogrel administered alone. Pharmacodynamic parameters were also measured and demonstrated that the change in inhibition of platelet aggregation (induced by 5 µM ADP) was correlated with the change in the exposure to clopidogrel active metabolite. The clinical significance of this finding is not clear.

Mycophenolate Mofetil (MMF): Administration of pantoprazole 40 mg twice daily for 4 days and a

single 1000 mg dose of MMF approximately one hour after the last dose of pantoprazole to 12 healthy subjects in a cross-over study resulted in a 57% reduction in the C_{max} and 27% reduction in the AUC of MPA. Transplant patients receiving approximately 2000 mg per day of MMF (n=12) were compared to transplant patients receiving approximately the same dose of MMF and pantoprazole 40 mg per day (n=21). There was a 78% reduction in the C_{max} and a 45% reduction in the AUC of MPA in patients receiving both pantoprazole and MMF.

In vivo studies also suggest that pantoprazole does not significantly affect the kinetics of the following drugs (cisapride, theophylline, diazepam [and its active metabolite, desmethyldiazepam], phenytoin, warfarin, metoprolol, nifedipine, carbamazepine, midazolam, clarithromycin, naproxen, piroxicam, and oral contraceptives [levonorgestrel/ethinyl estradiol]). Dosage adjustment of these drugs is not necessary when they are co-administered with pantoprazole. In other *in vivo* studies, digoxin, ethanol, glyburide, antipyrine, caffeine, metronidazole, and amoxicillin had no clinically relevant interactions with pantoprazole.

Based on studies evaluating possible interactions of pantoprazole with other drugs, no dosage adjustment is needed with concomitant use of the following: theophylline, cisapride, antipyrine, caffeine, carbamazepine, diazepam (and its active metabolite, desmethyldiazepam), diclofenac, naproxen, piroxicam, digoxin, ethanol, glyburide, an oral contraceptive (levonorgestrel/ethinyl estradiol), metoprolol, nifedipine, phenytoin, warfarin, midazolam, clarithromycin, metronidazole, or amoxicillin.

There was also no interaction with concomitantly administered antacids.

There have been postmarketing reports of increased INR and prothrombin time in patients receiving proton pump inhibitors, including pantoprazole sodium, and warfarin concomitantly *[see Drug Interactions (7.2)]*.

Although no significant drug-drug interactions have been observed in clinical studies, the potential for significant drug-drug interactions with more than once-daily dosing with high doses of pantoprazole has not been studied in poor metabolizers or individuals who are hepatically impaired.

Other Effects

In a clinical pharmacology study, pantoprazole sodium 40 mg given once daily for 2 weeks had no effect on the levels of the following hormones: cortisol, testosterone, triiodothyronine (T_3), thyroxine (T_4), thyroid-stimulating hormone (TSH), thyronine-binding protein, parathyroid hormone, insulin, glucagon, renin, aldosterone, follicle-stimulating hormone, luteinizing hormone, prolactin, and growth hormone.

In a 1-year study of GERD patients treated with pantoprazole sodium 40 mg or 20 mg, there were no changes from baseline in overall levels of T_3, T_4, and TSH.

12.4 Pharmacogenomics

CYP2C19 displays a known genetic polymorphism due to its deficiency in some subpopulations (e.g., approximately 3% of Caucasians and African-Americans and 17% to 23% of Asians are poor metabolizers). Although these subpopulations of pantoprazole poor metabolizers have elimination half-life values of 3.5 to 10 hours in adults, they still have minimal accumulation (≤ 23%) with once-daily dosing. For adult patients who are CYP2C19 poor metabolizers, no dosage adjustment is needed.

Similar to adults, pediatric patients who have the poor metabolizer genotype of CYP2C19 (CYP2C19 *2/*2) exhibited greater than a 6-fold increase in AUC compared to pediatric extensive (CYP2C19 *1/*1) and intermediate (CYP2C19 *1/*x) metabolizers. Poor metabolizers exhibited approximately 10-fold lower apparent oral clearance compared to extensive metabolizers.

For known pediatric poor metabolizers, a dose reduction should be considered.

13 NONCLINICAL TOXICOLOGY

13.1 Carcinogenesis, Mutagenesis, Impairment of Fertility

In a 24-month carcinogenicity study, Sprague-Dawley rats were treated orally with doses of 0.5 to 200 mg/kg/day, about 0.1 to 40 times the exposure on a body surface area basis of a 50 kg person dosed at 40 mg/day. In the gastric fundus, treatment at 0.5 to 200 mg/kg/day produced enterochromaffin-like (ECL) cell hyperplasia and benign and malignant neuroendocrine cell tumors in a dose-related manner. In the forestomach, treatment at 50 and 200 mg/kg/day (about 10 and 40 times the recommended human dose on a body surface area basis) produced benign squamous cell papillomas and malignant squamous cell carcinomas. Rare gastrointestinal tumors associated with pantoprazole treatment included an adenocarcinoma of the duodenum at 50 mg/kg/day and benign polyps and adenocarcinomas of the gastric fundus at 200 mg/kg/day. In the liver, treatment at 0.5 to 200 mg/kg/day produced dose-related increases in the incidences of hepatocellular adenomas and carcinomas. In the thyroid gland, treatment at 200 mg/kg/day produced increased incidences of follicular cell adenomas and carcinomas for both male and female rats.

In a 24-month carcinogenicity study, Fischer 344 rats were treated orally with doses of 5 to 50 mg/kg/day, approximately 1 to 10 times the recommended human dose based on body surface area. In the gastric fundus, treatment at 5 to 50 mg/kg/day produced enterochromaffin-like (ECL) cell hyperplasia and benign and malignant neuroendocrine cell tumors. Dose selection for this study may not have been adequate to comprehensively evaluate the carcinogenic potential of pantoprazole.

In a 24-month carcinogenicity study, B6C3F1 mice were treated orally with doses of 5 to 150 mg/kg/day, 0.5 to 15 times the recommended human dose based on body surface area. In the liver, treatment at 150 mg/kg/day produced increased incidences of hepatocellular adenomas and carcinomas in female mice. Treatment at 5 to 150 mg/kg/day also produced gastric-fundic ECL cell hyperplasia.

A 26-week p53 +/- transgenic mouse carcinogenicity study was not positive.

Pantoprazole was positive in the *in vitro* human lymphocyte chromosomal aberration assays, in one of two mouse micronucleus tests for clastogenic effects, and in the *in vitro* Chinese hamster ovarian cell/HGPRT forward mutation assay for mutagenic effects. Equivocal results were observed in the *in vivo* rat liver DNA covalent binding assay. Pantoprazole was negative in the *in vitro* Ames mutation assay, the *in vitro* unscheduled DNA synthesis (UDS) assay with rat hepatocytes, the *in vitro* AS52/GPT mammalian cell-forward gene mutation assay, the *in vitro* thymidine kinase mutation test with mouse lymphoma L5178Y cells, and the *in vivo* rat bone marrow cell chromosomal aberration assay.

There were no effects on fertility or reproductive performance when pantoprazole was given at oral doses up to 500 mg/kg/day in male rats (98 times the recommended human dose based on body surface area) and 450 mg/kg/day in female rats (88 times the recommended human dose based on body surface area).

13.2 Animal Toxicology and/or Pharmacology

Studies in neonatal/juvenile and adult rats and dogs were performed. The data from these studies revealed that animals in both age groups respond to pantoprazole in a similar manner. Gastric alterations, including increased stomach weights, increased incidence of eosinophilic chief cells in adult and neonatal/juvenile rats, and atrophy of chief cells in adult rats and in neonatal/juvenile dogs, were observed in the fundic mucosa of stomachs in repeated-dose studies. Decreases in red cell mass parameters, increases in cholesterol and triglycerides, increased liver weight, enzyme induction, and hepatocellular hypertrophy were also seen in repeated-dose studies in rats and/or dogs. Full to partial recovery of these effects were noted in animals of both age groups following a recovery period.

Reproductive Toxicology Studies

Reproduction studies have been performed in rats at oral doses up to 450 mg/kg/day (88 times the recommended human dose based on body surface area) and rabbits at oral doses up to 40 mg/kg/day (16 times the recommended human dose based on body surface area) and have revealed no evidence of impaired fertility or harm to the fetus due to pantoprazole.

14 CLINICAL STUDIES

Pantoprazole sodium delayed-release tablets were used in the following clinical trials.

14.1 Erosive Esophagitis (EE) Associated with Gastroesophageal Reflux Disease (GERD)

Adult Patients

A US multicenter, double-blind, placebo-controlled study of pantoprazole sodium 10 mg, 20 mg, or 40 mg once daily was conducted in 603 patients with reflux symptoms and endoscopically diagnosed EE of grade 2 or above (Hetzel-Dent scale). In this study, approximately 25% of enrolled patients had severe EE of grade 3, and 10% had grade 4. The percentages of patients healed (per protocol, n = 541) in this study are shown in Table 7.

Table 7: Erosive Esophagitis Healing Rates (Per Protocol)

	————Pantoprazole Sodium————			Placebo
	10 mg daily	20 mg daily	40 mg daily	
Week	(n = 153)	(n = 158)	(n = 162)	(n = 68)
4	45.6%+	58.4%+#	75%+*	14.3%
8	66%+	83.5%+#	92.6%+*	39.7%
+ ($p < 0.001$) pantoprazole sodium versus placebo				
* ($p < 0.05$) versus 10 mg or 20 mg pantoprazole sodium				
# ($p < 0.05$) versus 10 mg pantoprazole sodium				

In this study, all pantoprazole sodium treatment groups had significantly greater healing rates than the placebo group. This was true regardless of *H. pylori* status for the 40 mg and 20 mg pantoprazole sodium treatment groups. The 40 mg dose of pantoprazole sodium resulted in healing rates significantly greater than those found with either the 20 mg or 10 mg dose.

A significantly greater proportion of patients taking pantoprazole sodium 40 mg experienced complete relief of daytime and nighttime heartburn and the absence of regurgitation, starting from the first day of treatment, compared with placebo. Patients taking pantoprazole sodium consumed significantly fewer antacid tablets per day than those taking placebo.

Pantoprazole sodium 40 mg and 20 mg once daily were also compared with nizatidine 150 mg twice daily in a US multicenter, double-blind study of 243 patients with reflux symptoms and endoscopically diagnosed EE of grade 2 or above. The percentages of patients healed (per protocol, n = 212) are shown in Table 8.

Table 8: Erosive Esophagitis Healing Rates (Per Protocol)

	————Pantoprazole Sodium————		Nizatidine
	20 mg daily	40 mg daily	150 mg twice daily
Week	(n = 72)	(n = 70)	(n = 70)
4	61.4%+	64%+	22.2%
8	79.2%+	82.9%+	41.4%
+ ($p < 0.001$) pantoprazole sodium versus nizatidine			

Once-daily treatment with pantoprazole sodium 40 mg or 20 mg resulted in significantly superior rates of healing at both 4 and 8 weeks compared with twice-daily treatment with 150 mg of nizatidine. For the 40 mg treatment group, significantly greater healing rates compared to nizatidine were achieved

regardless of the *H. pylori* status.

A significantly greater proportion of the patients in the pantoprazole sodium treatment groups experienced complete relief of nighttime heartburn and regurgitation, starting on the first day and of daytime heartburn on the second day, compared with those taking nizatidine 150 mg twice daily. Patients taking pantoprazole sodium consumed significantly fewer antacid tablets per day than those taking nizatidine.

<u>Pediatric Patients Ages 5 Years through 16 Years</u>

The efficacy of pantoprazole sodium in the treatment of EE associated with GERD in pediatric patients ages 5 years through 16 years is extrapolated from adequate and well-conducted trials in adults, as the pathophysiology is thought to be the same. Four pediatric patients with endoscopically diagnosed EE were studied in multicenter, randomized, double-blind, parallel-treatment trials. Children with endoscopically diagnosed EE (defined as an endoscopic Hetzel-Dent score ≥ 2) were treated once daily for 8 weeks with one of two dose levels of pantoprazole sodium (20 mg or 40 mg). All 4 patients with EE were healed (Hetzel-Dent score of 0 or 1) at 8 weeks.

14.2 Long-Term Maintenance of Healing of Erosive Esophagitis

Two independent, multicenter, randomized, double-blind, comparator-controlled trials of identical design were conducted in adult GERD patients with endoscopically confirmed healed erosive esophagitis to demonstrate efficacy of pantoprazole sodium in long-term maintenance of healing. The two US studies enrolled 386 and 404 patients, respectively, to receive either 10 mg, 20 mg, or 40 mg of pantoprazole sodium delayed-release tablets once daily or 150 mg of ranitidine twice daily. As demonstrated in Table 9, pantoprazole sodium 40 mg and 20 mg were significantly superior to ranitidine at every timepoint with respect to the maintenance of healing. In addition, pantoprazole sodium 40 mg was superior to all other treatments studied.

Table 9: Long-Term Maintenance of Healing of Erosive Gastroesophageal Reflux Disease (GERD Maintenance): Percentage of Patients Who Remained Healed

	Pantoprazole sodium 20 mg daily	Pantoprazole sodium 40 mg daily	Ranitidine 150 mg twice daily
**Study 1**	n = 75	n = 74	n = 75
Month 1	91*	99*	68
Month 3	82*	93*#	54
Month 6	76*	90*#	44
Month 12	70*	86*#	35
**Study 2**	n = 74	n = 88	n = 84
Month 1	89*	92*#	62
Month 3	78*	91*#	47
Month 6	72*	88*#	39
Month 12	72*	83*	37
* ($p < 0.05$ vs. ranitidine)			
# ($p < 0.05$ vs. pantoprazole sodium 20 mg)			
Note: pantoprazole sodium 10 mg was superior ($p < 0.05$) to ranitidine in Study 2, but not Study 1.			

Pantoprazole sodium 40 mg was superior to ranitidine in reducing the number of daytime and nighttime heartburn episodes from the first through the twelfth month of treatment. Pantoprazole sodium 20 mg, administered once daily, was also effective in reducing episodes of daytime and nighttime heartburn in one trial, as presented in Table 10.

Table 10: Number of Episodes of Heartburn (mean ± SD)

		Pantoprazole sodium 40 mg daily	Ranitidine 150 mg twice daily
Month 1	Daytime	5.1 ± 1.6*	18.3 ± 1.6
	Nighttime	3.9 ± 1.1*	11.9 ± 1.1
Month 12	Daytime	2.9 ± 1.5*	17.5 ± 1.5
	Nighttime	2.5 ± 1.2*	13.8 ± 1.3
* (p < 0.001 vs. ranitidine, combined data from the two US studies)			

14.3 Pathological Hypersecretory Conditions Including Zollinger-Ellison Syndrome

In a multicenter, open-label trial of 35 patients with pathological hypersecretory conditions, such as Zollinger-Ellison syndrome, with or without multiple endocrine neoplasia-type I, pantoprazole sodium successfully controlled gastric acid secretion. Doses ranging from 80 mg daily to 240 mg daily maintained gastric acid output below 10 mEq/h in patients without prior acid-reducing surgery and below 5 mEq/h in patients with prior acid-reducing surgery.

Doses were initially titrated to the individual patient needs, and adjusted in some patients based on the clinical response with time *[see Dosage and Administration (2)]*. Pantoprazole sodium was well tolerated at these dose levels for prolonged periods (greater than 2 years in some patients).

16 HOW SUPPLIED/STORAGE AND HANDLING

How Supplied

Pantoprazole sodium delayed-release tablets, USP, **20 mg**, are supplied as light yellow to yellow color, oval shaped, biconvex, delayed-release tablets imprinted “A6” with black ink on one side and plain on the other side.
They are available as follows:

Bottles of 30: NDC 65162-636-03
Bottles of 90: NDC 65162-636-09

Pantoprazole sodium delayed-release tablets, USP, **40 mg**, are supplied as light yellow to yellow color, oval shaped, biconvex, delayed-release tablets imprinted “A37” with black ink on one side and plain on the other side.
They are available as follows:

Bottles of 30: NDC 65162-637-03
Bottles of 90: NDC 65162-637-09
Bottles of 500: NDC 65162-637-50
Bottles of 1000: NDC 65162-637-11

Storage

Store pantoprazole sodium delayed-release tablets, USP at 20° to 25°C (68° to 77°F); excursions permitted to 15° to 30°C (59° to 86°F) [See USP Controlled Room Temperature].

17 PATIENT COUNSELING INFORMATION

See FDA-Approved Medication Guide

- Caution patients that pantoprazole sodium delayed-release tablets should not be split, crushed, or chewed.
- Tell patients that pantoprazole sodium delayed-release tablets should be swallowed whole, with or without food in the stomach.
- Let patients know that concomitant administration of antacids does not affect the absorption of

pantoprazole sodium delayed-release tablets.

- Advise patients to immediately report and seek care for any cardiovascular or neurological symptoms including palpitation, dizziness, seizures, and tetany as these may be signs of hypomagnesemia *[see Warnings and Precautions (5.7)]*.
- Advise patients to immediately report and seek care for diarrhea that does not improve. This may be a sign of *Clostridium difficile* associated diarrhea *[see Warnings and Precautions (5.5)]*.

This product's label may have been updated. For current full prescribing information, please visit www.amneal.com.

Manufactured by:
Amneal Pharmaceuticals Pvt. Ltd.
Ahmedabad, INDIA 382220

Distributed by:
Amneal Pharmaceuticals
Bridgewater, NJ 08807

Rev. 11-2015-00

MEDICATION GUIDE

Pantoprazole (pan-TOE-pra-zole) Sodium Delayed-release Tablets

Read this Medication Guide before you start taking pantoprazole sodium delayed-release tablets and each time you get a refill. There may be new information. This information does not take the place of talking with your doctor about your medical condition or your treatment.

What is the most important information I should know about pantoprazole sodium delayed-release tablets?

Pantoprazole sodium delayed-release tabletsmay help your acid-related symptoms, but you could still have serious stomach problems. Talk with your doctor.

Pantoprazole sodium delayed-release tabletscan cause serious side effects, including:

- **Diarrhea.** Pantoprazole sodium delayed-release tablets may increase your risk of getting severe diarrhea. This diarrhea may be caused by an infection (*Clostridium difficile*) in your intestines.

Call your doctor right away if you have watery stool, stomach pain, and fever that does not go away.

- **Bone fractures.** People who take multiple daily doses of proton pump inhibitor medicines for a long period of time (a year or longer) may have an increased risk of fractures of the hip, wrist or spine. You should take pantoprazole sodium delayed-release tablets exactly as prescribed, at the lowest dose possible for your treatment and for the shortest time needed. Talk to your doctor about your risk of bone fracture if you take pantoprazole sodium delayed-release tablets.

Pantoprazole sodium delayed-release tablets can have other serious side effects. See **"What are the possible side effects of pantoprazole sodium delayed-release tablets?"**

What are pantoprazole sodiumdelayed-release tablets?

Pantoprazole sodium delayed-release tablets are a prescription medicine called a proton pump inhibitor (PPI).

Pantoprazole sodium delayed-release tablets reduce the amount of acid in your stomach.

Pantoprazole sodium delayed-release tablets are used in adults:

- for up to 8 weeks to heal acid-related damage to the lining of the esophagus (erosive esophagitis or EE) and to relieve symptoms caused by gastroesophageal reflux disease (GERD). If needed, your

doctor may decide to prescribe another 8 weeks of pantoprazole sodium delayed-release tablets.

- to maintain the healing of acid-related damage to the lining of the esophagus and help prevent return of heartburn symptoms caused by GERD. It is not known if pantoprazole sodium delayed-release tablets are safe and effective if used longer than 12 months (1 year).

GERD happens when acid in your stomach backs up into the tube (esophagus) that connects your mouth to your stomach. This may cause a burning feeling in your chest or throat, sour taste, or burping.

- for the long-term treatment of conditions where your stomach makes too much acid. This includes a rare condition called Zollinger-Ellison syndrome.

Pantoprazole sodium delayed-release tablets are used in children 5 years of age and older for up to 8 weeks to heal acid-related damage to the lining of the esophagus (erosive esophagitis or EE) caused by GERD.

It is not known if pantoprazole sodium delayed-release tablets are safe if used longer than 8 weeks in children. Pantoprazole sodium delayed-release tablets are not for use in children under 5 years of age.

Who should not take pantoprazole sodium delayed-release tablets?

Do not take pantoprazole sodium delayed-release tablets if you are:

- allergic to pantoprazole sodium or any of the other ingredients in pantoprazole sodium delayed-release tablets. See the end of this Medication Guide for a complete list of ingredients in pantoprazole sodium delayed-release tablets.
- allergic to any proton pump inhibitor (PPI) medicine.

What should I tell my doctor before taking pantoprazole sodium delayed-release tablets?

Before taking pantoprazole sodium delayed-release tablets, tell your doctor if you:

- have been told that you have low magnesium levels in your blood
- have liver problems
- have any other medical conditions
- are pregnant or plan to become pregnant. It is not known if pantoprazole sodium delayed-release tablets will harm your unborn baby.
- are breastfeeding or plan to breastfeed. Pantoprazole sodium may pass into your milk. You and your doctor should decide if you will take pantoprazole sodium delayed-release tablets or breastfeed. You should not do both. Talk with your doctor about the best way to feed your baby if you take pantoprazole sodium delayed-release tablets.

Tell your doctor about all of the medicines you take, including prescription and non-prescription drugs, vitamins and herbal supplements. Pantoprazole sodium delayed-release tablets may affect how other medicines work, and other medicines may affect how pantoprazole sodium delayed-release tablets works.

Especially tell your doctor if you take:

- atazanavir (Reyataz)
- nelfinavir (Viracept)
- warfarin (Coumadin, Jantoven)
- ketoconazole (Nizoral)
- a product that contains iron
- an antibiotic that contains ampicillin
- methotrexate
- mycophenolate mofetil (Cellcept)

Ask your doctor or pharmacist for a list of these medicines, if you are not sure.

Know the medicines that you take. Keep a list of them to show your doctor and pharmacist when you get a new medicine.

How should I take pantoprazole sodium delayed-release tablets?

- Take pantoprazole sodium delayed-release tablets exactly as prescribed by your doctor.
- Do not change your dose or stop pantoprazole sodium delayed-release tablets without talking to your doctor.
- If you forget to take a dose of pantoprazole sodium delayed-release tablets, take it as soon as you remember. If it is almost time for your next dose, do not take the missed dose. Take the next dose at your regular time. Do not take two doses to try to make up for a missed dose.
- If you take too much pantoprazole sodium delayed-release tablets, call your doctor right away or go to the nearest hospital emergency room.
- See the Instructions for Use at the end of this Medication Guide for detailed instructions about:

- how to take pantoprazole sodium delayed-release tablets

What are the possible side effects of pantoprazole sodium delayed-release tablets?

Pantoprazole sodium delayed-release tablets may cause serious side effects, including:

See "What is the most important information I should know about pantoprazole sodium delayed-release tablets?"

- **Chronic (lasting a long time) inflammation of the lining of the stomach (Atrophic Gastritis).** Taking pantoprazole sodium delayed-release tablets for a long period of time may increase the risk of inflammation to your stomach lining. You may or may not have symptoms. Tell your doctor if you have stomach pain, nausea, vomiting or weight loss.
- **Vitamin B-12 deficiency.** Pantoprazole sodium delayed-release tablets reduce the amount of acid in your stomach. Stomach acid is needed to absorb vitamin B-12 properly. Talk with your doctor about the possibility of vitamin B-12 deficiency if you have been on pantoprazole sodium delayed-release tablets for a long time (more than 3 years).
- **Low magnesium levels in your body.** This problem can be serious. Low magnesium can happen in some people who take a proton pump inhibitor medicine for at least 3 months. If low magnesium levels happen, it is usually after a year of treatment. You may or may not have symptoms of low magnesium.

Tell your doctor right away if you have any of these symptoms:

- seizures
- dizziness
- abnormal or fast heartbeat
- jitteriness
- jerking movements or shaking (tremors)
- muscle weakness
- spasms of the hands and feet
- cramps or muscle aches
- spasm of the voice box

Your doctor may check the level of magnesium in your body before you start taking pantoprazole sodium delayed-release tablets or during treatment, if you will be taking pantoprazole sodium delayed-release tablets for a long period of time.

The most common side effects with pantoprazole sodium delayed-release tablets in adults include:

• Headache	• Vomiting

• Diarrhea	• Gas
• Nausea	• Dizziness
• Stomach pain	• Pain in your joints

The most common side effects with pantoprazole sodium delayed-release tablets in children include:

• Upper respiratory infection	• Vomiting
• Headache	• Rash
• Fever	• Stomach pain
• Diarrhea	

Other side effects:

- **Serious allergic reactions.** Tell your doctor if you get any of the following symptoms with pantoprazole sodium delayed-release tablets:

- rash
- face swelling
- throat tightness
- difficult breathing

Your doctor may stop pantoprazole sodium delayed-release tablets if these symptoms happen.

Tell your doctor about any side effects that bother you or that do not go away.

These are not all the possible side effects with pantoprazole sodium delayed-release tablets. For more information, ask your doctor or pharmacist.

Call your doctor for medical advice about side effects. You may report side effects to FDA at 1-800-FDA-1088.

How should I store pantoprazole sodium delayed-release tablets?

- Store pantoprazole sodium delayed-release tablets at room temperature between 68° to 77°F (20° to 25°C).

Keep pantoprazole sodium delayed-release tablets and all medicines out of the reach of children.

General information about pantoprazole sodium delayed-release tablets

Medicines are sometimes prescribed for purposes other than those listed in a Medication Guide. Do not use pantoprazole sodium delayed-release tablets for a condition for which it was not prescribed. Do not give pantoprazole sodium delayed-release tablets to other people, even if they have the same symptoms you have. It may harm them.

This Medication Guide summarizes the most important information about pantoprazole sodium delayed-release tablets. For more information, ask your doctor. You can ask your doctor or pharmacist for information that is written for healthcare professionals.

For more information, go to www.amneal.com or call toll-free 1-877-835-5472.

What are the ingredients in pantoprazole sodium delayed-release tablets?

Active ingredient: pantoprazole sodium sesquihydrate

Inactive ingredients in pantoprazole sodium delayed-release tablets: ammonium hydroxide, crospovidone, hydroxyl propyl cellulose, hypromellose, iron oxide black, iron oxide yellow, mannitol, methacrylic acid copolymer dispersion, polyethylene glycol, polyvinyl alcohol, povidone, propylene glycol, shellac, sodium carbonate, sodium hydroxide, sodium stearyl fumarate, talc, titanium dioxide and triethyl citrate.

Instructions for Use

<u>Pantoprazole sodium delayed-release tablets:</u>

- You can take pantoprazole sodium delayed-release tablets with food or on an empty stomach.
- Swallow pantoprazole sodium delayed-release tablets whole.
- If you have trouble swallowing a pantoprazole sodium delayed-release 40 mg tablet, you can take two 20 mg tablets instead.
- Do not split, chew, or crush pantoprazole sodium delayed-release tablets.

This Medication Guide has been approved by the U.S. Food and Drug Administration.

This product's label may have been updated. For current full prescribing information, please visit www.amneal.com.

Manufactured by:

Amneal Pharmaceuticals Pvt. Ltd.

Ahmedabad, INDIA 382220

Distributed by:

Amneal Pharmaceuticals

Bridgewater, NJ 08807

Rev. 11-2015-00

PACKAGE LABEL.PRINCIPAL DISPLAY PANEL

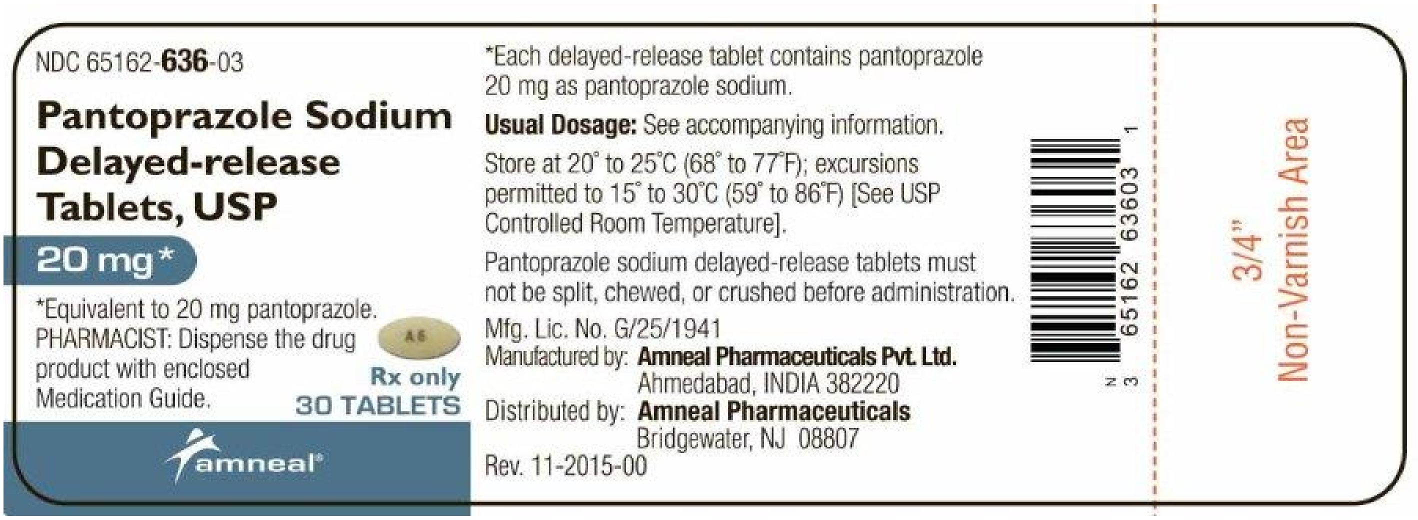

PACKAGE LABEL.PRINCIPAL DISPLAY PANEL

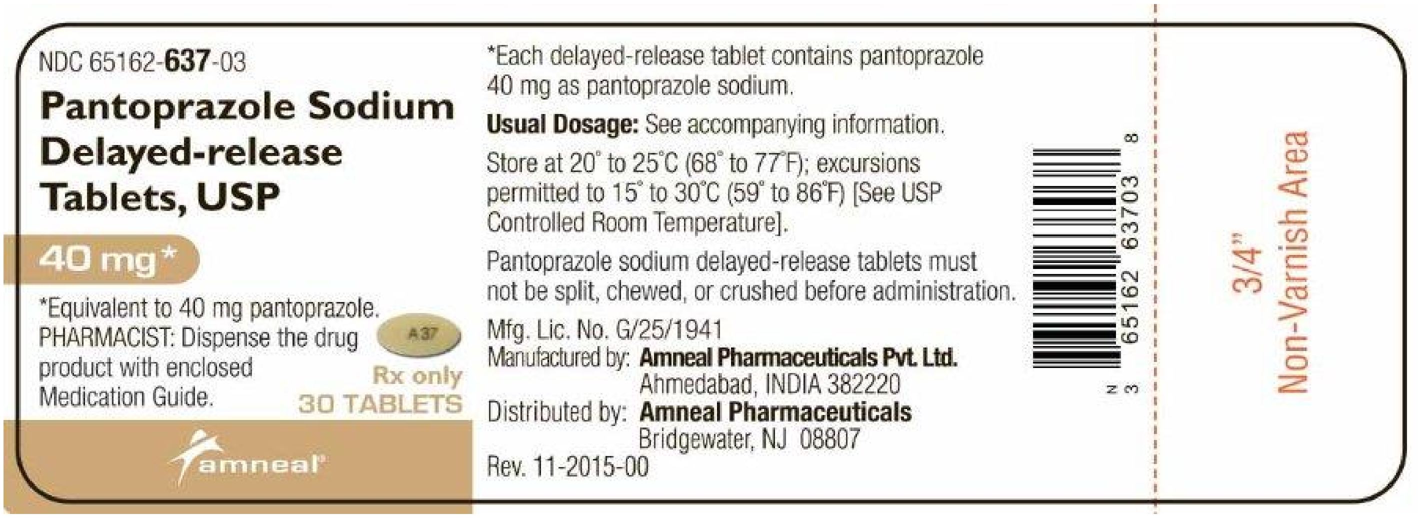

PANTOPRAZOLE SODIUM

pantoprazole tablet, delayed release

Product Information			
**Product Type**	HUMAN PRESCRIPTION DRUG	**Item Code (Source)**	NDC:65162-636
**Route of Administration**	ORAL		

Active Ingredient/Active Moiety		
**Ingredient Name**	**Basis of Strength**	**Strength**
**PANTOPRAZOLE SODIUM** (UNII: 6871619Q5X) (PANTOPRAZOLE - UNII:D8TST4O562)	PANTOPRAZOLE	20 mg

Inactive Ingredients	
**Ingredient Name**	**Strength**
**AMMONIA** (UNII: 5138Q19F1X)	
**CROSPOVIDONE (12 MPA.S AT 5%)** (UNII: 40UAA97IT9)	
**HYDROXYPROPYL CELLULOSE, LOW SUBSTITUTED** (UNII: 2165RE0K14)	
**HYPROMELLOSES** (UNII: 3NXW29V3WO)	
**FERROSOFERRIC OXIDE** (UNII: XM0M87F357)	
**FERRIC OXIDE YELLOW** (UNII: EX438O2MRT)	
**MANNITOL** (UNII: 3OWL53L36A)	
**METHACRYLIC ACID - METHYL METHACRYLATE COPOLYMER (1:1)** (UNII: 74G4R6TH13)	
**POLYETHYLENE GLYCOLS** (UNII: 3WJQ0SDW1A)	
**POVIDONE** (UNII: FZ989GH94E)	
**PROPYLENE GLYCOL** (UNII: 6DC9Q167V3)	
**POLYVINYL ALCOHOL, UNSPECIFIED** (UNII: 532B59J990)	
**SHELLAC** (UNII: 46N107B71O)	
**SODIUM CARBONATE** (UNII: 45P3261C7T)	
**SODIUM HYDROXIDE** (UNII: 55X04QC32I)	
**SODIUM STEARYL FUMARATE** (UNII: 7CV7WJK4UI)	
**TALC** (UNII: 7SEV7J4R1U)	
**TITANIUM DIOXIDE** (UNII: 15FIX9V2JP)	

**TRIETHYL CITRATE** (UNII: 8Z96QXD6UM)	

Product Characteristics

**Color**	YELLOW (light yellow)	**Score**	no score
**Shape**	OVAL	**Size**	8mm
**Flavor**		**Imprint Code**	A6
**Contains**			

Packaging

#	Item Code	Package Description	Marketing Start Date	Marketing End Date
1	NDC:65162-636-03	30 in 1 BOTTLE; Type 0: Not a Combination Product	06/20/2016	
2	NDC:65162-636-09	90 in 1 BOTTLE; Type 0: Not a Combination Product	06/20/2016	

Marketing Information

Marketing Category	Application Number or Monograph Citation	Marketing Start Date	Marketing End Date
ANDA	ANDA205119	06/20/2016	

PANTOPRAZOLE SODIUM

pantoprazole tablet, delayed release

Product Information

**Product Type**	HUMAN PRESCRIPTION DRUG	**Item Code (Source)**	NDC:65162-637
**Route of Administration**	ORAL		

Active Ingredient/Active Moiety

Ingredient Name	Basis of Strength	Strength
**PANTOPRAZOLE SODIUM** (UNII: 6871619Q5X) (PANTOPRAZOLE - UNII:D8TST4O562)	PANTOPRAZOLE	40 mg

Inactive Ingredients

Ingredient Name	Strength
**AMMONIA** (UNII: 5138Q19F1X)	
**CROSPOVIDONE (12 MPA.S AT 5%)** (UNII: 40UAA97IT9)	
**HYDROXYPROPYL CELLULOSE, LOW SUBSTITUTED** (UNII: 2165RE0K14)	
**HYPROMELLOSES** (UNII: 3NXW29V3WO)	
**FERROSOFERRIC OXIDE** (UNII: XM0M87F357)	
**FERRIC OXIDE YELLOW** (UNII: EX438O2MRT)	
**MANNITOL** (UNII: 3OWL53L36A)	
**METHACRYLIC ACID - METHYL METHACRYLATE COPOLYMER (1:1)** (UNII: 74G4R6TH13)	
**POLYETHYLENE GLYCOLS** (UNII: 3WJQ0SDW1A)	
**POVIDONE** (UNII: FZ989GH94E)	
**PROPYLENE GLYCOL** (UNII: 6DC9Q167V3)	

**POLYVINYL ALCOHOL, UNSPECIFIED** (UNII: 532B59J990)	
**SHELLAC** (UNII: 46N107B71O)	
**SODIUM CARBONATE** (UNII: 45P3261C7T)	
**SODIUM HYDROXIDE** (UNII: 55X04QC32I)	
**SODIUM STEARYL FUMARATE** (UNII: 7CV7WJK4UI)	
**TALC** (UNII: 7SEV7J4R1U)	
**TITANIUM DIOXIDE** (UNII: 15FIX9V2JP)	
**TRIETHYL CITRATE** (UNII: 8Z96QXD6UM)	

Product Characteristics

**Color**	YELLOW (light yellow)	**Score**	no score
**Shape**	OVAL	**Size**	11mm
**Flavor**		**Imprint Code**	A37
**Contains**			

Packaging

#	Item Code	Package Description	Marketing Start Date	Marketing End Date
**1**	NDC:65162-637-03	30 in 1 BOTTLE; Type 0: Not a Combination Product	06/20/2016	
**2**	NDC:65162-637-09	90 in 1 BOTTLE; Type 0: Not a Combination Product	06/20/2016	
**3**	NDC:65162-637-11	1000 in 1 BOTTLE; Type 0: Not a Combination Product	06/20/2016	
**4**	NDC:65162-637-50	500 in 1 BOTTLE; Type 0: Not a Combination Product	06/20/2016	

Marketing Information

Marketing Category	Application Number or Monograph Citation	Marketing Start Date	Marketing End Date
ANDA	ANDA205119	06/20/2016	

Labeler - Amneal Pharmaceuticals of New York, LLC (123797875)

Establishment

Name	Address	ID/FEI	Business Operations
Amneal Pharmaceuticals Pvt. Ltd.		915076126	ANALYSIS(65162-636, 65162-637) , LABEL(65162-636, 65162-637) , MANUFACTURE(65162-636, 65162-637) , PACK(65162-636, 65162-637)

Revised: 2/2016 Amneal Pharmaceuticals of New York, LLC
